# A triboelectric radical generation route to chlorine disinfectants from brine

**DOI:** 10.1038/s41467-026-75108-3

**Published:** 2026-07-07

**Authors:** Han Qian, Jianming Liu, Ning Wu, Shaoxin Li, Xinhong Song, Feiyao Yang, Morten Willatzen, Jiajia Shao, Vishnu Shankar, Daping Chu, Richard N. Zare, Zhong Lin Wang, Di Wei

**Affiliations:** 1 Beijing Institute of Nanoenergy and Nanosystems, Chinese Academy of Sciences, Beijing, China; 2 School of Nanoscience and Engineering, University of Chinese Academy of Sciences, Beijing, China; 3 Department of Chemistry, Stanford University, Stanford, CA USA; 4 Program in Immunology, Stanford University School of Medicine, Stanford, CA USA; 5 Centre for Photonic Devices and Sensors, University of Cambridge, Cambridge, UK

**Keywords:** Sustainability, Electron transfer, Nanoparticles

## Abstract

Contact electrification (CE), one of the earliest documented physical phenomena, has traditionally been associated with triboelectric charging but rarely considered as a driver of chemical transformations. Here we show that CE generates radicals at solid-liquid interfaces, establishing contact-electro-chemistry (CE-Chemistry) as a metal-catalyst-free route that uses ambient mechanical energy. Ultrasonically driven contact-separation of fluorinated ethylene propylene (FEP) particulates in brine generates hydroxyl radicals (•OH) and chlorine radicals (•Cl), forming active chlorine [hypochlorous acid (HOCl) and hypochlorite ions (ClO^−^)], without detectable chlorine gas (Cl_2_) production. Using natural seawater, this strategy achieves active chlorine production of 208.8 μmol g^−1^ h^−1^ and operates for over 700 h without detectable FEP dissolution or toxic intermediates. Cation hydration engineering modulates interfacial electrical double layers, strengthening triboelectric fields and tuning reaction kinetics. This work enables Cl_2_-free disinfectant production from brine without external bias or illumination, opening avenues for heterogeneous catalysis and CE-driven reaction design.

## Abstract (web summary)

Contact electrification is shown to turn seawater into active chlorine disinfectants without chlorine gas, external voltage, light, or metal catalysts, offering a route for distributed disinfectant production and interfacial reaction design.

## Introduction

Contact electrification (CE), the scientific description of triboelectrification, has been recognized for more than two millennia^1^. It is pervasive across natural and astrophysical processes, governing phenomena as diverse as pollen adhesion on insect bodies^2^, lightning formation in thunderclouds^3^, and the aggregation of cosmic dust into protoplanetary bodies^4^. In contemporary research, CE has been extensively exploited in the development of triboelectric nanogenerators for mechanical energy harvesting^5,6^, self-powered sensing platforms and integrated systems^7–10^, as well as interfacial probes^11–13^. Despite these developments, the role of CE in mediating or accelerating chemical transformations, particularly at solid-liquid interfaces, has remained largely overlooked. To address this gap, we define contact-electro-chemistry (CE-Chemistry) as a reaction paradigm in which charge transfer and radical generation arise directly from CE-induced interfacial processes^14,15^. Unlike conventional electrochemistry, CE-Chemistry proceeds without the constraints of electrochemical windows, external bias, or reliance on metallic catalysts^15–17^. The highly localized nature of the transferred charges on dielectrics could suppress side reactions and enable spatiotemporal decoupling of paired redox processes^18^. Recently, proof-of-concept studies have underscored the broad potential of CE-Chemistry. Wang and co-workers^19^ employed contact-induced reactive oxygen species (ROS) to degrade methyl orange. Chen et al.^20^ demonstrated cost-effective hydrogen peroxide synthesis by flowing water through microchannels. Li et al.^21^ achieved complete lithium leaching from spent lithium-ion batteries through CE-driven recycling. Wei and colleagues^15^ further revealed that redox selectivity in CE-Chemistry is governed by the standard electrode potential (SEP) of participating species, identifying a threshold that dictates whether oxidative or reductive pathways dominate.

Despite these advances, current studies in CE-Chemistry have predominantly centered on the electrons and radicals produced during contact events, while overlooking the fundamental role of the electrical double layer (EDL) at solid-liquid interfaces^22–24^. In particular, the dynamic modulation of interfacial EDL structures, which governs charge separation, field amplification, and ion-specific reactivity, remains insufficiently explored. Prior studies have shown that EDL-driven fields can promote diverse reactions, including CO_2_ reduction^25–28^, ammonia synthesis^29^, organic chlorination^30^, and polymer membrane formation^31^, all of which critically depend on intense interfacial fields. Such highly localized electric fields, reaching 10^7^−10^9^ V m^−1^, are not restricted to solid-liquid interfaces but also emerge at gas-liquid interfaces in microdroplets^32–35^ or microbubbles^36–38^, at liquid-liquid interfaces in oil-water emulsions^39,40^, and even at solid-solid interfaces during stick-slip friction such as tape peeling^41,42^. Interfacial triboelectric fields of this magnitude can polarize or activate nearby molecules, generate reactive radical species, reduce activation energy barriers, stabilize transient intermediates, and trigger bond activation under ambient conditions, highlighting their central role in initiating chemistry directly at interfaces^25,27,39,43–46^. In CE-Chemistry, these triboelectric fields are not only intrinsic but tunable. Theoretical simulations by COMSOL Multiphysics reveal that they can readily reach intensities on the order of 10^10^ V m^−1^. Beyond the advantages conferred by such strong interfacial fields, the incorporation of ultrasonication introduces rapid and repeated contact–separation events, which dramatically enhance mass transfer and enable concurrent reactions across multiple interfaces^47,48^. Together, these effects establish CE-Chemistry as a powerful, metal-free platform for driving chemical transformations, laying the foundation for a sustainable paradigm in interfacial chemistry^18,22,49^.

To elucidate the unique advantages of CE-Chemistry and the central function of the interfacial triboelectric field, CE-driven radical generation was employed to achieve metal-free synthesis of active chlorine disinfectants directly from seawater. Active chlorine, comprising hypochlorous acid (HOCl) and hypochlorite ions (ClO^−^), underpins a multibillion-dollar global market, serving as an indispensable disinfectant in water treatment, public health, food safety, and medicine^50,51^. Its importance extends beyond sanitation to sustainable chemical manufacturing, where demand continues to rise in response to population growth, urbanization, and heightened requirements for environmental protection^52–54^. At present, industrial-scale production of active chlorine predominantly relies on the chlor-alkali process, in which chlorine gas (Cl_2_) is generated as a corrosive and toxic intermediate^55,56^. Although this process is industrially mature, it faces inherent safety concerns and limitations for distributed applications: the transportation and storage of Cl_2_ are subject to strict requirements and may pose serious environmental and health risks^57^. A promising pathway lies in metal-free synthetic strategies that bypass Cl_2_ generation altogether and may enable distributed active chlorine production by harnessing high-entropy mechanical energy from the environment. By directly producing active chlorine from abundant natural seawater, this strategy circumvents the dependence on high-purity water and the hazards of Cl_2_, while enabling sustainable distributed production^58,59^. The absence of metal catalysts further eliminates concerns of resource scarcity, toxicity, and secondary pollution, aligning with principles of circular economy and sustainable development^60,61^.

**Fig. 1:**
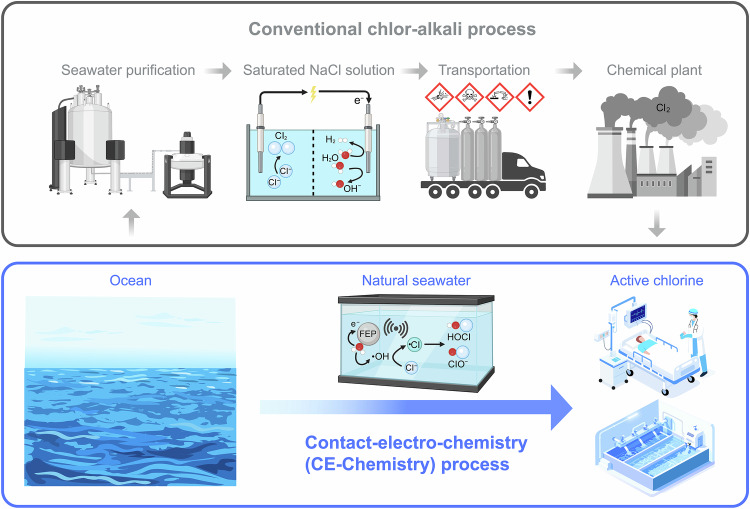
Flow chart of the chlor-alkali process and contact-electro-chemistry (CE-Chemistry) process for the production of disinfectants. The system includes a chlor-alkali process for disinfectant production, specifically consisting of seawater purification, saturated brine electrolysis, transport, and chemical synthesis. In contrast to the conventional chlor-alkali process, the CE-Chemistry synthesis of active chlorine effectively circumvents the production of Cl_2_ as a hazardous intermediate, yielding active chlorine with superior selectivity. The FEP dielectric powders demonstrate remarkable stability and recyclability, permitting multiple utilization cycles. The prepared active chlorine could be applied in public health and water treatment.

Here, we present a sustainable strategy for the direct production of active chlorine from natural seawater via CE-Chemistry, revealing that the driving force for radical generation and subsequent reactions originates from the intense interfacial triboelectric field (up to 10^10^ V m^−1^) formed at the solid-liquid interface. Under ultrasonication, cavitation bubbles induce continuous solid-liquid contact and separation in the presence of fluorinated ethylene propylene (FEP) powder, leading to triboelectric charge accumulation and the establishment of the strong interfacial field. The resulting triboelectric field continuously produces ROS and chlorine radicals (•Cl), enhancing reaction rates by modulating kinetics and reducing activation barriers. Active chlorine then forms in a single step through the coupling of hydroxyl radicals (•OH) with •Cl, resulting in the production of HOCl, which is in equilibrium with H^+^ and ClO^−^ (*K* = ~3.0 × 10^−8^ at 25 °C)^62^. This process avoids the formation of toxic Cl_2_ intermediates, the use of precious metal catalysts, and the requirement for external electric or light fields, while maintaining stability beyond 700 h (Fig. 1). This work delivers a safe, sustainable, and environmentally benign route for distributed active chlorine production directly from seawater, circumventing the limits of conventional electrochemical windows, while also revealing the central role of interfacial triboelectric fields in CE-Chemistry and demonstrating how field strength and reaction pathways can be precisely tuned through regulation of the EDL.

## Results

### Disinfectant synthesis through CE-Chemistry

**Fig. 2:**
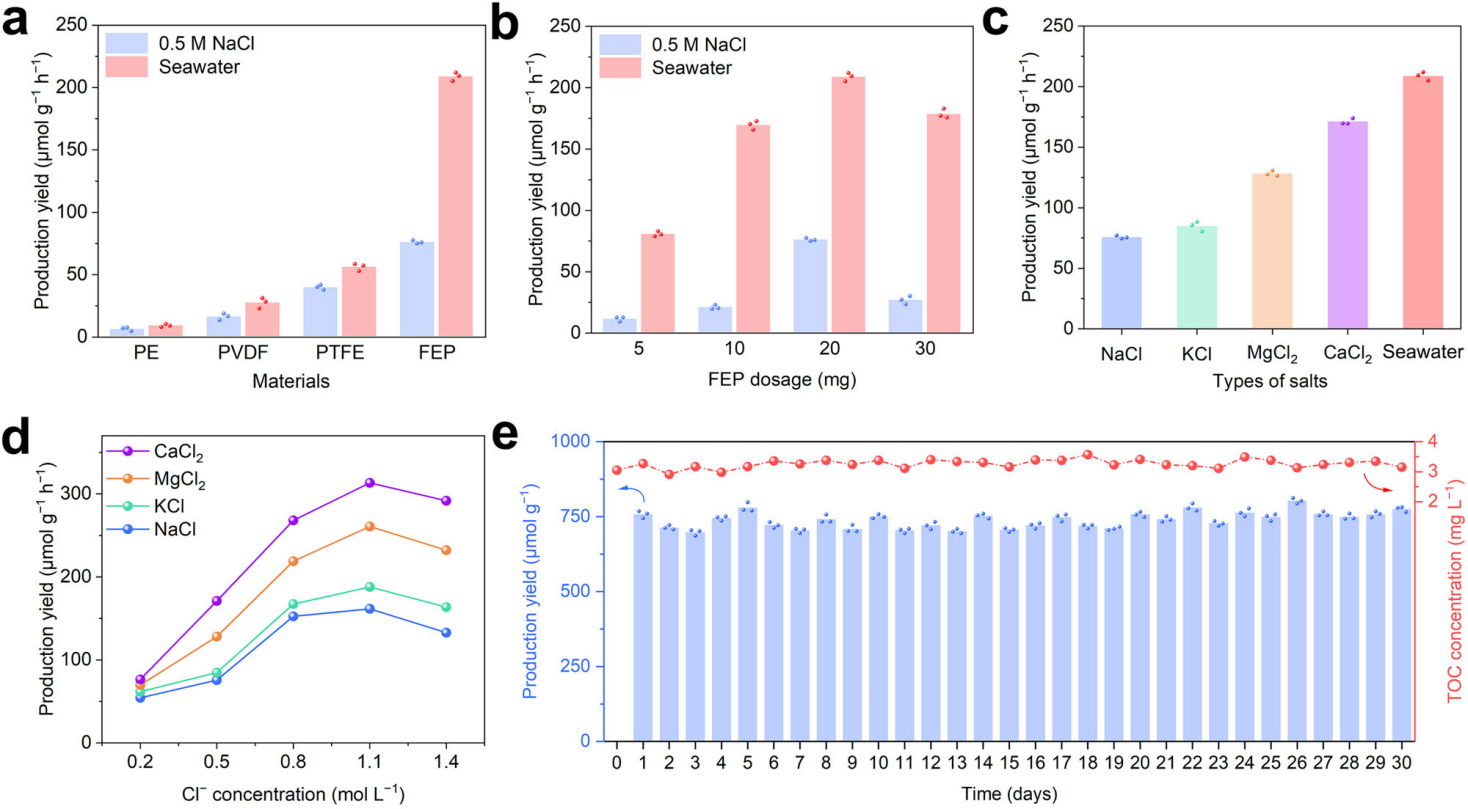
CE-Chemistry disinfectant synthesis in natural seawater. Comparison of active chlorine production yields under **a** different dielectric materials [PE (polyethylene), PVDF (polyvinylidene fluoride), PTFE (polytetrafluoroethylene), fluorinated ethylene propylene (FEP)], **b** different FEP dosages, **c** different salts at the concentration of Cl^−^ at 0.5 M, **d** different concentrations of Cl^−^ from 0.2 to 1.4 M. **e** Stability and recyclability of the dielectrics for active chlorine production for 1 month [total organic carbon (TOC)]. All data in bar charts are presented as mean values from three independent experiments (*n* = 3), with dots indicating individual measurements.

Key parameters influencing the CE-Chemistry process for direct disinfectant production from natural seawater were systematically evaluated, with active chlorine yields determined via the o-tolidine method^58^ (Supplementary Fig. 1). As shown in Fig. 2a, the choice of dielectric material exerts a decisive influence on the active chlorine production yield. FEP exhibited substantially higher yields than PTFE (polytetrafluoroethylene), PVDF (polyvinylidene fluoride), and PE (polyethylene) in both natural (seawater) and synthetic (0.5 M NaCl aqueous solution) brines of comparable salinity, consistent with the triboelectric series of these dielectric materials. The relationship between FEP dosage and yield is presented in Fig. 2b: the yield increases with dosage up to 20 mg, but declines when the dosage is further raised to 20–30 mg. This decrease is most likely attributable to the scattering of ultrasonic waves by excess dielectric powder^21^, thereby reducing CE-Chemistry efficiency and suppressing active chlorine production. In Fig. 2c, the effect of cation species on active chlorine production yield is investigated. For cations of identical valence, smaller hydration sizes correlate with higher yields of chlorine disinfectants, likely caused by weaker EDL shielding and stronger modulation of the interfacial triboelectric field. The underlying mechanism will be elaborated in a later section^63,64^. As shown in Fig. 2d, the relationship between chloride ion concentration and active chlorine yield exhibits a nonlinear trend: the yield increases from 0.2 M to a maximum at ~1.1 M and then declines. This behavior reflects a balance between two competing effects. Higher reactant concentration initially promotes the reaction, but excessive ion concentration intensifies EDL shielding, which ultimately dominates and suppresses interfacial electron transfer and subsequent chemical transformations at the solid-liquid interface^15^. The reaction volume was increased from 10 to 100 mL to evaluate ultrasonic wave attenuation. Negligible attenuation was observed below 50 mL, whereas further increases led to pronounced attenuation, limiting reaction kinetics and reducing active chlorine production, consistent with previous reports^21^ (Supplementary Fig. 2). Temperature exerts a multifaceted influence on the reaction: relatively low temperatures favor product stability and accumulation, while excessively low temperatures limit reaction kinetics. In contrast, higher temperatures enhance reaction kinetics and mass transport, but also accelerate EDL formation and screening, which suppresses interfacial charge transfer and leads to a decrease in CE-Chemistry yield. Therefore, a trade-off exists between active chlorine generation and accumulation, resulting in optimal performance at 10 °C within the range of 5–40 °C^49,65–67^ (Supplementary Fig. 3). Moreover, ultrasonic power directly affects the reaction by controlling the frequency of solid-liquid contact–separation events, with higher power promoting more efficient electron transfer and ion migration, thereby enhancing reaction kinetics and increasing product yield^16,68^ (Supplementary Fig. 4).

### Recyclability and stability of the dielectrics

The recyclability and physicochemical stability of the dielectric material are critical evaluation metrics. To assess this, we examined the long-term, multi-cycle performance of FEP powder in active chlorine production. A continuous CE-Chemistry process was conducted over 24-h cycles under ultrasonication, using natural seawater as the feedstock. At the end of each cycle, samples were collected for yield measurement and total organic carbon (TOC) analysis, while the reacted FEP powder was simply filtered and reused in fresh seawater. As shown in Fig. 2e, the FEP powder maintained stable performance without significant decline over 700 h of continuous operation, confirming its excellent recyclability and stability. Notably, TOC analysis of the solution after each cycle revealed negligible variation in TOC concentration, confirming the exceptional structural stability of FEP powder without detectable degradation or dissolution throughout the CE-Chemistry process (Fig. 2e). To further verify its physicochemical robustness, we systematically characterized the FEP powder before and after long-term operation. Particle size distribution and scanning electron microscopy (SEM) analysis showed no changes in particle size or morphology, excluding aggregation or decomposition (Supplementary Figs. 5 and 6). Complementary spectroscopic studies provided deeper chemical insights: Raman spectrum exhibited identical skeletal vibration modes, Fourier transform infrared (FTIR) spectrum showed a stable fingerprint region below 1500 cm^−1^, and X-ray photoelectron spectroscopy (*XPS*) analysis revealed no shifts in binding energy or emergence of new peaks (Supplementary Figs. 7–9). X-ray diffraction (XRD) patterns further confirmed unchanged diffraction peak positions before and after cycling (Supplementary Fig. 10). Collectively, these results establish that FEP powder retains outstanding recyclability and physicochemical stability as a chemically inert dielectric, enabling reliable active chlorine production via CE-Chemistry.

### Reaction mechanism

**Fig. 3:**
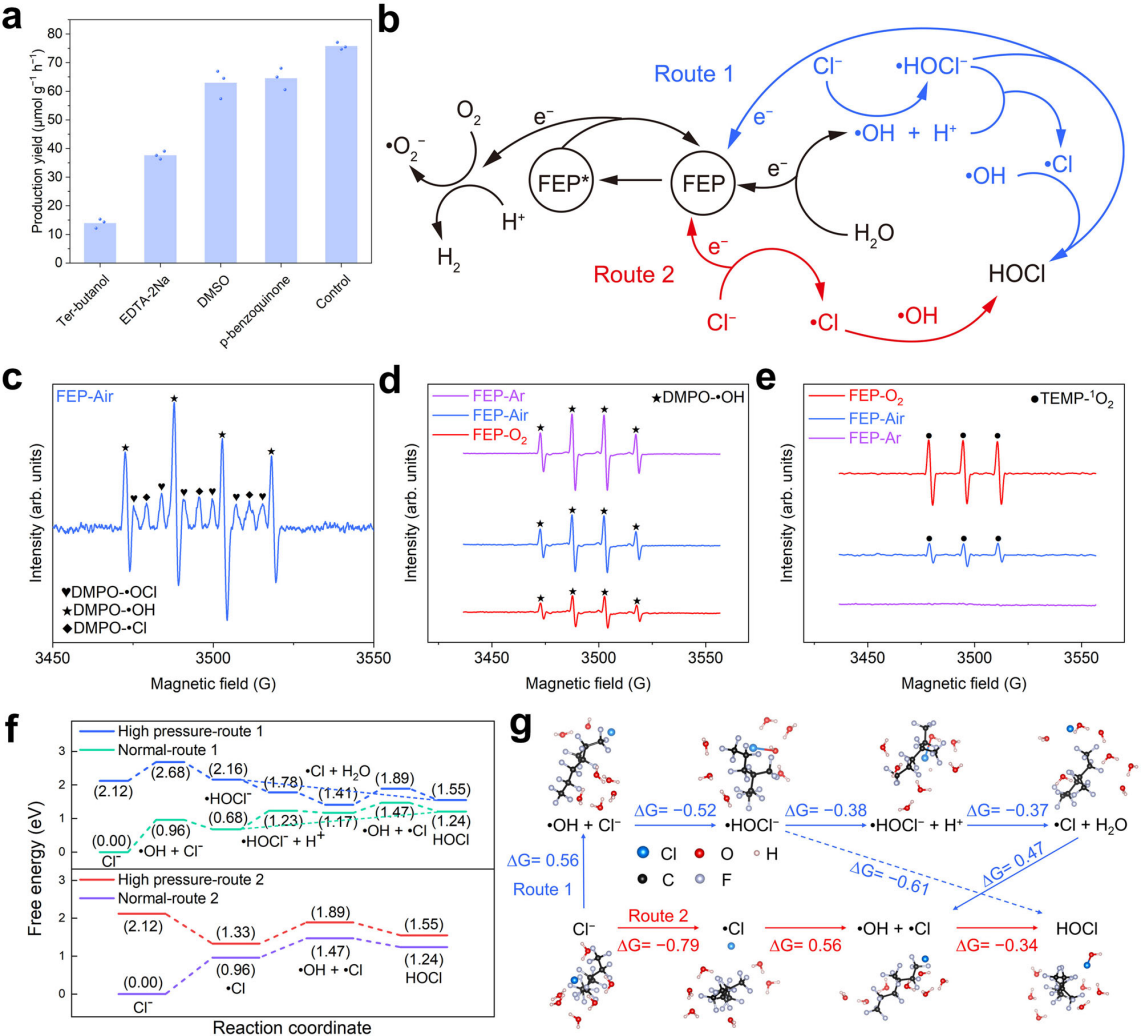
Investigation on the contribution of radicals to CE-Chemistry disinfectant synthesis. **a** Production yields of active chlorine for 1 h with various radical scavengers, including ter-butanol, p-benzoquinone, DMSO, and EDTA-2Na, which are respectively regarded as hydroxyl radical, superoxide radical, electron, and proton scavengers. All data in bar charts are presented as mean values from three independent experiments (*n* = 3), with dots indicating individual measurements. **b** Proposed mechanism for the generation of active chlorine by CE-Chemistry synthesis (FEP* is proposed to describe the charged state of FEP). **c** The electron paramagnetic resonance (EPR) spectra of radical adduct signal trapped by DMPO in the presence of FEP particles during ultrasonication in an air atmosphere. The EPR spectra for **d** DMPO-•OH and **e** TEMP-^1^O_2_ in the presence of FEP particles during ultrasonication in different atmospheres. **f** Free energy profiles of the oxidation of Cl^−^ to HOCl were compared under atmospheric and ultrasonic conditions in two different routes. **g** The required free energy (unit: eV) for each step under ultrasonication conditions in two different routes. Route 1 involves the reaction of Cl^−^ with •OH to form a •${{\mbox{HOCl}}}^{-}$ intermediate, which subsequently either directly produces HOCl or is further converted to •Cl, followed by recombination with •OH. Route 2 involves the direct oxidation of Cl^−^ to •Cl, which is then rapidly captured by •OH to generate HOCl. Atom colors: Cl, blue; O, red; H, light brown; C, black; F, silver gray.

The reaction mechanism underlying active chlorine production via CE-Chemistry was next investigated. Consistent with prior studies, the formation and collapse of cavitation bubbles were induced by ultrasonication, promoting repeated CE at the FEP-water interface. This process induces electron transfer from hydroxide ions (OH^−^), generated by the dynamic equilibrium of water, to the FEP surface, thereby producing •OH radicals^69,70^ (Eq. 1). At the same time, oxygen in the cavitation bubbles can receive electrons from the FEP surface to form superoxide anions^19^ (•${{\mbox{O}}}_{2}^{-}$, Eq. 2), while protons in solution may also accept electrons to generate hydrogen via the hydrogen evolution pathway (H_2_, Supplementary Fig. 11 and Eq. 3). To further elucidate the roles of different active species, experiments were conducted using various scavengers, each maintained at a concentration of 1 mM^19,21^, which resulted in pronounced differences in the active chlorine production yield. The addition of ter-butanol caused a marked reduction in active chlorine yield, indicating that •OH radicals serve as a key active species in the CE-Chemistry process. Similarly, the introduction of EDTA-2Na, a proton scavenger, led to a decrease in active chlorine yield, highlighting the essential role of protons in the reaction. In contrast, the addition of DMSO and p-benzoquinone, acting as electron and superoxide anion scavengers, respectively, produced only minor effects on the yield, suggesting a limited contribution of these species to the active chlorine generation (Fig. 3a). Based on the scavenger experiments described in this report and previous publications, a plausible CE-Chemistry mechanism for the conversion of natural seawater to active chlorine is proposed (Fig. 3b). For Route 1 to generate HOCl, the •${{\mbox{HOCl}}}^{-}$ intermediate is required. Initially, •OH radicals react with Cl^−^ to form the •${{\mbox{HOCl}}}^{-}$ intermediate (Eq. 4), which subsequently interacts with H^+^ to form •Cl radicals^71^ (Eq. 5). These •Cl radicals then combine with •OH radicals to yield HOCl (Eq. 6). Meanwhile, •${{\mbox{HOCl}}}^{-}$ intermediate may also lose electron directly on the surface of FEP and transform into HOCl (Eq. 7). In contrast, Route 2 only requires •Cl and •OH. The •Cl can be generated via direct electron abstraction from Cl⁻, subsequently reacting with •OH to yield HOCl (Eq. 8). The equilibrium (Eq. 9) is strongly pH-dependent, with HOCl dominating at low pH (≤6) and OCl^−^ prevailing at high pH (≥8). Importantly, HOCl is the true disinfectant form, exhibiting 80 times greater efficacy than OCl^−^ due to its superior ability to penetrate microbial membranes^62^. Notably, unlike the conventional chlor-alkali process, this entire reaction sequence proceeds without the formation of Cl_2_ as a toxic intermediate (Supplementary Figs. 12–14) and is free from the limitations of the electrochemical window and the occurrence of inevitable side reactions.

1 $${{{\rm{FEP}}}}+{{{\rm{OH}}}}^{-}\to {{\bullet }}{{{\rm{OH}}}}+{{{\rm{e}}}}^{-}({{{\rm{FEP}}}})$$

2 $${{{\rm{e}}}}^{-}({{{\rm{FEP}}}})+{{{\rm{O}}}}_{2}\to {{\bullet }}{{{\rm{O}}}}_{2}^{-}+{{{\rm{FEP}}}}$$

3 $${2{{{\rm{e}}}}}^{-}({{{\rm{FEP}}}})+{2{{{\rm{H}}}}}^{+}\to {{{\rm{H}}}}_{2}+{{{\rm{FEP}}}}$$

4 $${{\bullet }}{{{\rm{OH}}}}+{{{\rm{Cl}}}}^{-}\to {{\bullet }}{{{\rm{HOCl}}}}^{-}$$

5 $${{\bullet }}{{{{\rm{HOCl}}}}}^{-}+{{{{\rm{H}}}}}^{+}\to {{\bullet }}{{{\rm{Cl}}}}+{{{{\rm{H}}}}}_{2}{{{\rm{O}}}}$$

6 $${{\bullet }}{{{\rm{OH}}}}+{{\bullet }}{{{\rm{Cl}}}}\to {{{\rm{HOCl}}}}$$

7 $${{\bullet }}{{{{\rm{HOCl}}}}}^{-}+{{{\rm{FEP}}}}\to {{{\rm{HOCl}}}}+{{{{\rm{e}}}}}^{-}({{{\rm{FEP}}}})$$

8 $${{{{\rm{Cl}}}}}^{-}+{{{\rm{FEP}}}}\to {{\bullet }}{{{\rm{Cl}}}}+{{{{\rm{e}}}}}^{-}({{{\rm{FEP}}}})$$

9 $${{{\rm{HOCl}}}}\rightleftharpoons {{{{\rm{H}}}}}^{+}+{{{\rm{OCl}}}}^{-}$$

10 $${{{\rm{HOCl}}}}+{{\bullet }}{{{\rm{Cl}}}}\to {{\bullet }}{{{\rm{OCl}}}}+{{{\rm{HCl}}}}$$

11 $$2{{{\rm{HOCl}}}}\to 2{{{\rm{HCl}}}}+{{{{\rm{O}}}}}_{2}$$

In addition, electron paramagnetic resonance (EPR) spectroscopy was employed to confirm the generation of •OH, •Cl, and •${{\mbox{O}}}_{2}^{-}$ species. To detect •OH and •Cl, the spin-trapping reagent DMPO (5,5-dimethyl-1-pyrroline N-oxide) was utilized^72^. Upon addition of DMPO to deionized (DI) water in the presence of FEP powder, a characteristic quadruplet signal corresponding to DMPO-•OH (1:2:2:1) was observed, whereas no signal appeared in the control group without FEP, indicating that the CE-Chemistry process induces the formation of •OH radicals^15,73^ (Supplementary Fig. 15). When DI water was replaced with a 0.5 M NaCl solution, the addition of the DMPO spin-trapping agent yielded not only the characteristic quadruplet signal of DMPO-•OH but also signals corresponding to •Cl and chlorine oxide radicals (•OCl), confirming the generation of •Cl during the CE-Chemistry process^74,75^ (Fig. 3c). The •OCl species is generated from HOCl (Eq. 10). Concurrently, singlet oxygen (^1^O_2_), an oxidative product of •${{\mbox{O}}}_{2}^{-}$, exhibited a pronounced triple-peak signal in the presence of FEP, whereas no signal was observed in its absence, further confirming the reduction of oxygen to •${{\mbox{O}}}_{2}^{-}$ during the CE-Chemistry process (Eq. 2, Supplementary Fig. 16).

Moreover, the reaction mechanism was further corroborated under varying atmospheric and pH conditions through EPR and spectrophotometric analyses (Supplementary Fig. 17). The EPR measurements described above were performed under an air atmosphere. When the CE-Chemistry process was conducted under argon (Ar) or oxygen (O_2_) atmospheres, the DMPO-•OH quadruplet signal was observed to increase under Ar and decrease under O_2_ (Fig. 3d), a trend corroborated by spectrophotometric analysis using the o-phenylenediamine (OPD) reagent^76^ (Supplementary Fig. 18). This behavior might be attributed to the oxidative nature of O_2_, which diminishes CE between the materials, thereby reducing charge transfer and radical generation. In contrast, the •${{\mbox{O}}}_{2}^{-}$ signal exhibited an opposite trend, with its intensity highest under O_2_, intermediate under air, and nearly absent under Ar atmosphere (Fig. 3e). The nitro blue tetrazolium (NBT) reagent tests confirmed the same trend^21^ (Supplementary Fig. 19). These results indicate that, relative to an air atmosphere, •OH concentration increases and •${{\mbox{O}}}_{2}^{-}$ concentration decreases under Ar, whereas the opposite occurs under O_2_. Based on this characteristic, the reaction mechanism was further substantiated: active chlorine production increased under Ar but decreased significantly under O_2_ (Supplementary Fig. 20). This confirms the predominant role of •OH in the CE-Chemistry generation of active chlorine, while •${{\mbox{O}}}_{2}^{-}$ exerts minimal influence on the reaction (Fig. 3b). Additionally, the pH of the CE-Chemistry system markedly affects the active chlorine yield, with the highest output obtained under mildly alkaline conditions (pH = 10, Supplementary Fig. 21). This pH dependence is governed not by a single factor, but by the combined effects of reactive species generation kinetics and the acid-base-equilibrium-dependent speciation of active chlorine. Under acidic conditions, active chlorine predominantly exists as HOCl^58^, which is more susceptible to decomposition under the localized and transient high temperatures generated during cavitation bubble collapse, thereby reducing the overall yield (Eq. 11). In contrast, under strongly alkaline conditions, although OCl^−^ is thermodynamically more stable, the formation of •Cl is significantly suppressed, resulting in diminished active chlorine production. The density functional theory (DFT) calculations further indicate that, at excessively high pH, the high OH^−^ concentration causes FEP to preferentially extract electrons from hydroxide ions, suppressing the •Cl generation pathway (Route 2). On the other hand, excessively high pH also weakens the oxidizing ability of •OH^77–80^, which in turn suppresses the •Cl generation pathway in Route 1. As a result, both •Cl generation routes are inhibited to different extents, suppressing •Cl formation and active chlorine production under strongly alkaline conditions. Notably, the yield does not show a simple intermediate trend in the neutral range of pH = 6-8, because this region represents a sensitive transition window in which HOCl and OCl^−^ rapidly interconvert and the apparent yield is jointly regulated by •Cl generation, •OH participation, active chlorine speciation, and product stability. Consequently, the pH dependence exhibits a pronounced nonlinear behavior rather than a smooth transition, a phenomenon that has also been reported in photocatalytic and electrocatalytic active chlorine synthesis^58,59^. Long-term CE-Chemistry experiments comparing 0.5 M NaCl solution and natural seawater revealed distinct behaviors: in NaCl solution, the yield initially increased, peaked around 1 h, and subsequently declined, whereas in natural seawater, the yield steadily increased and stabilized after 12 h (Supplementary Fig. 22). This difference is primarily governed by pH conditions. In seawater, buffering ion pairs such as ${{\mbox{HCO}}}_{3}^{-}$/${{\mbox{CO}}}_{3}^{-}$ might maintain the pH above 7, resulting in minimal fluctuations^58^, whereas in the NaCl solution, the pH rapidly dropped to approximately 4, creating unfavorable conditions for active chlorine accumulation (Supplementary Fig. 23). To confirm that the yield difference arises from pH rather than salinity, we followed ASTM D 1141-1998 standards to examine different NaCl concentrations (Supplementary Table 1). We also characterized the ionic composition and concentrations of the natural seawater used in this study by ion chromatography (IC), inductively coupled plasma optical emission spectroscopy (ICP-OES), and titration, and found that the Cl^−^ concentration in natural seawater was very close to that in 0.5 M NaCl, while 0.003 M of ${{\mbox{HCO}}}_{3}^{-}$ was also present (Supplementary Table 2). Compared with seawater, concentration showed a negligible effect on yield (Supplementary Fig. 24). The effect of pH on active chlorine yield further supports the proposed reaction mechanism (Fig. 3b). The DFT calculations further verified the reaction pathways, and the high-pressure environment induced by cavitation was also considered by adjusting the unit cell volume^19^. Notably, under the high-pressure conditions of ultrasonication, the energy required for the formation steps of the •${{\mbox{HOCl}}}^{-}$, •Cl, and HOCl decrease to varying degrees (Fig. 3f). This could be attributed to the reduced energy barriers under ultrasonic excitation, which facilitates electron transfer from H_2_O to FEP compared to ambient pressure conditions^19^. Calculated energy barriers for electron transfer between water molecules and FEP are presented in Supplementary Table 3, revealing that smaller unit cells tend to facilitate lower energy barriers, with the corresponding values provided. Consequently, under the influence of ultrasonication, the formation of •Cl and its subsequent reaction with •OH to form HOCl are more likely to occur. The specific two reaction pathways for the formation of active chlorine via CE-Chemistry are shown in Fig. 3g.

### Interfacial triboelectric field

Recent advances in CE-Chemistry have demonstrated that spontaneous interfacial electron transfer and ROS generation occur during the contact and separation of solid dielectrics with liquids, initiating chemical reactions^22,49^. However, the kinetic mechanisms of CE-Chemistry and the regulation of EDL at the solid-liquid interface remain underexplored, leading to a predominant focus on the reactions themselves rather than the fundamental mechanisms. Here, it is proposed that ROS generation and chemical transformations in CE-Chemistry are driven by the intense interfacial triboelectric field. This hypothesis is validated by modulating the EDL to control both the triboelectric field intensity and the reaction dynamics. Specifically, repeated contact and separation between solid dielectrics and water induce coupled electron transfer and ion migration, resulting in triboelectric charge accumulation on the dielectric surface and the formation of EDL at the solid-liquid interface. This powerful interfacial triboelectric field not only triggers ROS formation but also polarizes and activates nearby molecules, lowers activation energy barriers, and stabilizes reactive intermediates.

**Fig. 4:**
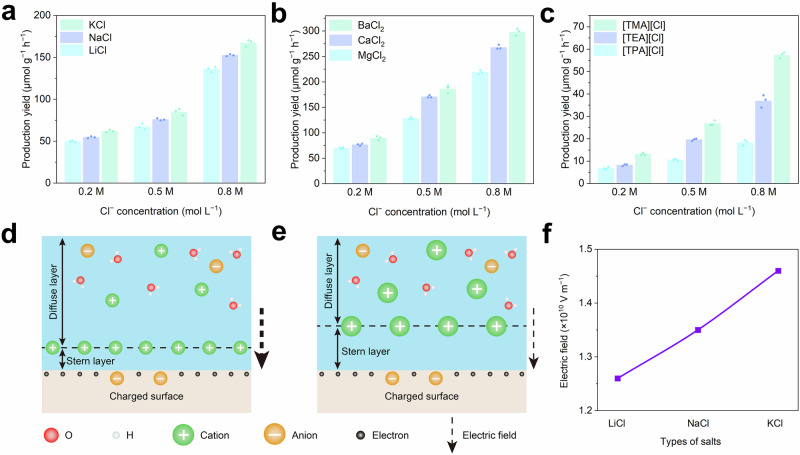
Proposed model of the impact of cation effect on CE-Chemistry. **a** Production yields of active chlorine for 1 h under different concentrations of KCl, NaCl, and LiCl. **b** Production yields of active chlorine for 1 h under different concentrations of BaCl_2_, CaCl_2_, and MgCl_2_. **c** Production yields of active chlorine for 1 h under different concentrations of tetramethylammonium chloride [TMA][Cl], tetraethylammonium chloride [TEA][Cl], and tetrapropylammonium chloride [TPA][Cl]. **d** Cations with smaller hydration radii demonstrate reduced Stern layer thickness, elevated ion concentrations at OHP, and enhanced triboelectric field intensity. **e** Cations with larger hydration radii exhibit expanded Stern layer dimensions, reduced cation concentrations at OHP, and weakened triboelectric field intensity. **f** Comparison of interfacial triboelectric fields within the Stern layer in different salt solutions. All data in bar charts are presented as mean values from three independent experiments (*n* = 3), with dots indicating individual measurements.

Taking active chlorine production via CE-Chemistry as a representative case, the EDL can be modulated by varying cation hydration size, thereby controlling both the interfacial triboelectric field and the reaction kinetics. Figure 4a–c illustrates a consistent trend in active chlorine yield across different concentrations, valences, and inorganic/organic cations: smaller cation hydration sizes correlate with higher yields (under identical conditions). This effect is attributed to cation-dependent modulation of the EDL. During the coupled electron-transfer and ion-migration process, the EDL could be established (Supplementary Fig. 25), with the Stern layer hosting a highly localized solid-liquid interfacial triboelectric field. Cations with smaller hydration shells induce a more compact Stern layer, enhancing the triboelectric field strength (Fig. 4d, e), a trend further corroborated by COMSOL simulations. The hydration sizes of alkali metal cations follow the sequence K^+^ <Na^+^ <Li^+^^81^(Supplementary Table 4). Accordingly, under identical conditions, the EDL in KCl solution is more compact than in NaCl or LiCl solutions (Supplementary Fig. 26), with the interfacial triboelectric field within the Stern layer estimated at 1.46 × 10^10^ V m^−1^, exceeding that of NaCl (1.35 × 10^10^ V m^−1^) and LiCl (1.26 × 10^10 ^V m^−1^) (Fig. 4f). A stronger triboelectric field in the KCl system results in more favorable ROS reaction kinetics, specifically a lower energy barrier of electron transfer from water to FEP (Supplementary Fig. 27). Simultaneously, the more compact, negatively charged EDL structure intensifies shielding effects, weakening the triboelectric field in the diffuse layer and repulsion toward anions (Cl^−^, •HOCl^−^) within that region (Supplementary Fig. 28). Cations with smaller hydration sizes experience reduced mutual repulsion, leading to higher concentrations at the outer Helmholtz plane (OHP) and stronger electrostatic attraction to anions in the diffuse layer. Consequently, smaller cation hydration sizes increase the local concentration of reactant and intermediate anions near the OHP, favoring the reaction (Supplementary Figs. 29 and 30). These results are consistent with previous studies^25,27,82,83^. In summary, reduced cation hydration sizes enhance both the interfacial triboelectric field strength governing ROS kinetics and the local anion concentration in the reaction zone, resulting in higher active chlorine yields and further substantiating the CE-Chemistry interfacial triboelectric field mechanism.

## Discussion

In summary, by harnessing CE, one of the oldest known physical phenomena, we have developed a sustainable CE-Chemistry approach for the direct generation of active chlorine from brine. This process is driven by intense interfacial triboelectric fields at solid-liquid interfaces that act to initiate radical formation and accelerate chemical reactions. The field strength can be precisely regulated via EDL engineering, through the selection of cations with distinct hydration characteristics. The strategy presents distinct practical advantages: the dielectric materials are intrinsically stable and recyclable, the process operates without conventional noble-metal catalysts, and the reaction proceeds independently of electrochemical window constraints or inevitable side reactions, thereby enabling spatiotemporal decoupling of paired redox processes. Critically, active chlorine can be produced in a single step directly from natural seawater, avoiding toxic, corrosive Cl_2_ formation while utilizing an abundant, low-cost feedstock. These findings provide fundamental insights into CE-Chemistry mechanisms and underscore the potential of highly engineered solid-liquid interfaces as quasi-electrodes capable of activating reaction pathways inaccessible in bulk solutions. Such a route may be particularly relevant for niche scenarios that are difficult to address by centralized chlor-alkali infrastructure, such as marine equipment interfaces, ship coatings, localized antibiofouling/anticorrosion systems, and small-scale or emergency disinfection units. This work provides a metal-free, CE-driven proof-of-concept for interfacial chemical transformations, with the potential to enable distributed reactions in the future by harnessing high-entropy mechanical energy from the environment.

## Methods

### Chemical reagents

KCl (99%), NaCl (99%), LiCl (99%), BaCl_2_ (99%), CaCl_2_ (99%), MgCl_2_ (99%), tetramethylammonium chloride [TMA][Cl], tetraethylammonium chloride [TEA][Cl], and tetrapropylammonium chloride [TPA][Cl] were purchased from Shanghai Macklin Biochemical Co., Ltd. Ter-butanol (98.0%), p-benzoquinone (97%), ethylenediaminetetraacetic acid disodium salt (C10H14N2Na2O8·2H2O, EDTA-2Na, 99%), and dimethyl sulfoxide (DMSO) were purchased from Sinopharm Chemical Reagent Co., Ltd. 5,5-dimethyl-1-pyrroline N-oxide (DMPO) and 2,2,6,6-tetramethyl-4-piperidone hydrochloride (TEMP, C9H20N) were purchased from Dojindo Co., Ltd. Natural seawater sampling site: Rizhao City, Shandong Province, China (35°32′28.44″ N, 119°38′9.56″ E).

### Sample preparation

First, salt solutions with different Cl^−^ concentrations (0.2, 0.5, 0.8, 1.1, and 1.4 M) and salt types (NaCl, KCl, MgCl_2_, and CaCl_2_) were prepared using DI water, purged with argon, and stirred for 24 h to obtain the corresponding stable solutions (Natural seawater was subjected to the same pretreatment). Next, polymer powders (PE, PVDF, PTFE, and FEP) with different masses (5, 10, 20, and 30 mg) were added to beakers containing 10 mL of the above salt solutions or natural seawater. A thermostatic ultrasonic bath (40 kHz, 120 W) was used to facilitate the CE-Chemistry reactions while maintaining the solution temperature to 20 °C (Supplementary Fig. 31). The reaction was conducted under optimal conditions of 20 °C, 20 mg FEP, 10 mL of natural seawater, and thermostatic ultrasonication at 40 kHz and 120 W for 1 h. For systematic investigations of the effects of solution volume (10, 25, 50, 75, 100 mL), temperature (5, 10, 20, 30, 40 °C), or ultrasonic power (60, 120, 200, 300, 400 W), all tests were designed based on these optimal conditions, with only a single variable altered each time. Samples for EPR analysis were prepared in 10 mL of DI water/0.5 M NaCl solution containing 20 mg FEP powder, 0.1 mL of DMPO or TEMP was added to the solution as a spin trapping agent to capture radical species. The tests were conducted after 30 min ultrasonication in different atmospheres (Air, O_2_, and Ar). Samples for various scavengers (ter-butanol, p-benzoquinone, DMSO, and EDTA-2Na) were prepared by separately adding each scavenger to the original solution to achieve a final concentration of 1 mM. Samples for the detection of radical species were prepared by adding 20 mg of FEP powder to 10 mL of OPD or NBT solution (10 mg mL^−1^). After ultrasonication for different durations (10, 20, and 30 min) under various atmospheres (Air, O_2_, and Ar), the changes in the UV-Vis absorption peaks at 424 and 259 nm were monitored. Samples for H_2_ analysis were prepared in 50 mL of DI water, 0.5 M NaCl solution, or natural seawater containing 20 mg FEP powder. Prior to ultrasonication, the reaction system was purged with argon to establish an oxygen-free atmosphere. The solution was then subjected to ultrasonication, and gas samples were collected from the reactor headspace at regular intervals during the reaction.

### Determination and quantification of active chlorine using UV-Vis spectroscopy

Active chlorine concentrations were determined and quantified using UV-Vis spectroscopy by the o-tolidine method. The 1 mL solution was taken from the beaker and diluted to 10 mL within the detection range, and then 0.5 mL of o-tolidine solution (1 g L^−1^ o-tolidine, pH adjusted to 0 with hydrochloric acid) was added. The absorption spectrum was measured after 1 min, and the absorption intensity was recorded at 437.5 nm. The standard concentration-absorbance curve was calibrated using a standard solution of sodium hypochlorite.

### Characterization

The UV-Vis absorption spectra of samples were recorded with a Hitachi UV-4150 on a range of 200–700 nm. The SEM images of the samples were obtained using an FEI Nova 450. The energy dispersive X-ray (EDX) was conducted on FEI Nova 450 equipped with an AMETEK Octane Super appendix. FTIR spectroscopy was recorded on a Bruker Vertex 80 v in the range 400–2000 cm^−1^. The chemical states of FEP before/after were measured by a near atmospheric pressure X-ray photoelectron spectrometer (NAP-*XPS*, SPECS, Germany). XRD spectra of FEP were measured using an advanced diffractometer (Bruker-D8, Germany) with a working voltage of 40 kV. Mass spectrometry analyses were performed using a gas chromatography-mass spectrometry system (Shimadzu, GC-MS-QP2020 NX) and an electrospray ionization mass spectrometry system (Thermo Fisher, ESI-MS-Orbitrap Exploris 120). EPR was recorded on a Bruker EMX plus-9.5/12/P/L. The measurements were conducted in X-Band (9.830243 GHz), with amplitude modulation of 1 G, microwave power of 2 mW, amplitude modulation frequency of 100 kHz, and conversion time of 60.00 ms, with a time constant of 40.96 ms. The Raman spectroscopy analysis was conducted on a LabRam HR evolution (HORIBA, SAS France), using a range from 300 to 1500 cm^−1^. The TOC in the samples was measured using a TOC-L CPH analyzer (Shimadzu, Japan). The ionic composition and concentrations of the natural seawater used in the experiments were determined by ion chromatography (IC), inductively coupled plasma optical emission spectroscopy (ICP-OES), and titration. Hydrogen was detected using a gas chromatograph (GC-2030 Nexis, Japan).

### DFT calculations

First-principles calculations based on DFT were performed using the projector-augmented wave (PAW) method, as implemented in the Vienna Ab-initio Simulation Package (VASP) 5.4.4. The exchange-correlation energy was described by the Perdew-Burke-Ernzerhof (PBE) functional within the generalized gradient approximation (GGA). Structural optimizations were performed with a Γ-centered 1 × 1 × 1 k-point grid and a plane-wave energy cutoff of 550 eV. The convergence criteria were set to 10^−6^ eV for the total energy difference between consecutive self-consistent field iterations and 0.005 eV Å^−1^ for the Hellmann-Feynman forces on each atom. To simulate the high-pressure environment induced by unit cell volume adjustment, tetragonal supercells with dimensions of 15 Å × 15 Å × 25 Å and 8 Å × 8 Å × 15 Å were constructed, respectively. Moreover, we have evaluated the effect of different unit cell volumes on the Gibbs free energy of the key reaction (•Cl + •OH → HOCl), which shows good energy convergence across the tested volume range, confirming that the selected cell sizes are reasonable (Supplementary Fig. 32). The atomic coordinates of the optimized computational models can be found in Supplementary Data 1.

### Simulation of EDL and interfacial triboelectric fields

The ion behavior near the FEP surface was theoretically simulated using a commercial finite-element software package, COMSOL Multiphysics 6.3. The numerical simulation was performed based on coupled Poisson and Nernst-Planck equations by setting appropriate boundary parameters. The Nernst-Planck equation defines the flux of each ion species. The ionic flux (${{{{\bf{J}}}}}_{{{{\bf{i}}}}}$) is calculated as:

12 $${{{{\bf{J}}}}}_{{{{\bf{i}}}}}=-{{{{\rm{D}}}}}_{{{{\rm{i}}}}}\nabla {{{{\rm{c}}}}}_{{{{\rm{i}}}}}-{{{{\rm{u}}}}}_{{{{\rm{m}}}},{{{\rm{i}}}}}{{{{\rm{z}}}}}_{{{{\rm{i}}}}}{{{\rm{F}}}}{{{{\rm{c}}}}}_{{{{\rm{i}}}}}\nabla {{{\rm{\phi }}}}$$

where ${{{{\rm{D}}}}}_{{{{\rm{i}}}}}$ is the diffusion coefficient, ${{{{\rm{c}}}}}_{{{{\rm{i}}}}}$ is the concentration, ${{{{\rm{u}}}}}_{{{{\rm{m}}}},{{{\rm{i}}}}}$ is the mobility, ${{{{\rm{z}}}}}_{{{{\rm{i}}}}}$ is the charge of the species *i*, *F* is Faraday’s constant, ${{{\rm{\phi }}}}$ is the electrical potential.

The relationship between the electric potential and ion concentrations can be defined by the Poisson equation:

13 $${{{{\rm{\bigtriangledown }}}}}^{2}\Phi=-\frac{{{{\rm{F}}}}}{{{{\rm{\varepsilon }}}}}{\sum}_{{{{\rm{i}}}}}\,{{{{\rm{Z}}}}}_{{{{\rm{i}}}}}{{{{\rm{c}}}}}_{{{{\rm{i}}}}}$$

where ε is permittivity of the fluid.

According to Gauss’s law, the triboelectric field inside a closed double layer is constant because the layer is uncharged and has a uniform dielectric constant. Thus, based on the Stern theory that describes a dense double electric layer with constant thickness ${{{{\rm{\lambda }}}}}_{{{{\rm{S}}}}}$, the following Robin type boundary conditions are applied to the electrolyte potential:

14 $${{{\rm{\phi }}}}+{{{{\rm{\lambda }}}}}_{{{{\rm{S}}}}}\left({{{\bf{n}}}}\cdot \nabla {{{\rm{\phi }}}}\right)={{{{\rm{\phi }}}}}_{{{{\rm{M}}}}}$$

where ${{{{\rm{\phi }}}}}_{{{{\rm{M}}}}}$ is the solid surface potential, which is measured for the bulk solution, and ${{{\bf{n}}}}$ is the boundary normal vector, pointing from the electrolyte domain to the outside.

For the case where the thickness of the Stern layer is not zero, the condition can be restated as the surface charge condition depending on the potential difference between the solid surface potential and the electrolyte potential${{{{\rm{\phi }}}}}_{\Delta }$ on the outer Helmholtz surface (*x* = 0):

15 $${{{\bf{n}}}}\cdot \left(-{{{\rm{\varepsilon }}}}\nabla {{{\rm{\phi }}}}\right)=-\frac{{{{\rm{\varepsilon }}}}{{{{\rm{\phi }}}}}_{\Delta }}{{{{{\rm{\lambda }}}}}_{{{{\rm{S}}}}}}$$

16 $${{{{\rm{\phi }}}}}_{\Delta }={{{{\rm{\phi }}}}}_{{{{\rm{M}}}}}-{{{\rm{\phi }}}}$$

Generally, the system is simplified in steady-state conditions, and when the system reaches a stationary regime, the ionic flux should satisfy the time- independent continuity:

17 $$\nabla \cdot {{{{\bf{J}}}}}_{{{{\rm{i}}}}}=0$$

The geometric model is one-dimensional (with an interval between 0 and *L*), containing a domain representing the electrolyte phase starting from the solid surface, passing through the diffusion double layer, and finally reaching the electrically neutral bulk solution. The length of *L* is 4.3 nm, and the maximum size of the grid cell at the left end is 4.3 × 10^−3^ nm. The concentration of the cation bulk is set at 0.5 M, and the concentration of Cl^−^ is set at 0.499 M, taking into account that the concentration of the intermediate •${{\mbox{HOCl}}}^{-}$ is 1 mM.

### Reporting summary

Further information on research design is available in the Nature Portfolio Reporting Summary linked to this article.

## Supplementary information

Supplementary Information

Peer Review File

Description of Additional Supplementary Files

Supplementary Data 1–25

Reporting Summary

## Source data

Source data

## Data Availability

All data supporting the findings of this work are available within the paper and its Supplementary Information. Source data are provided with this paper.

## References

[1] Lin, S., Chen, X. & Wang, Z. L. Contact electrification at the liquid–solid interface. *Chem. Rev.* **122**, 5209–5232 (2022). 34160191 10.1021/acs.chemrev.1c00176

[2] Sutton, G. P., Clarke, D., Morley, E. L. & Robert, D. Mechanosensory hairs in bumblebees (Bombus terrestris) detect weak electric fields. *Proc. Natl. Acad. Sci. USA* **113**, 7261–7265 (2016). 27247399 10.1073/pnas.1601624113 PMC4932954

[3] Berdeklis, P. & List, R. The ice crystal–graupel collision charging mechanism of thunderstorm electrification. *J. Atmos. Sci.* **58**, 2751–2770 (2001).

[4] Steinpilz, T. et al. Electrical charging overcomes the bouncing barrier in planet formation. *Nat. Phys.* **16**, 225–229 (2020).

[5] Sun, H. et al. Impedance decoupling strategy to enhance the real-time powering performance of TENG for multi-mode sensing. *Nat. Commun.* **16**, 6001 (2025). 40593744 10.1038/s41467-025-61166-6 PMC12217497

[6] Ji, P. et al. Roadbed tribological energy harvester. *Sci. Adv.* **11**, eadv9379 (2025). 40532008 10.1126/sciadv.adv9379 PMC13112539

[7] Tang, W., Sun, Q. & Wang, Z. L. Self-powered sensing in wearable electronics—a paradigm shift technology. *Chem. Rev.* **123**, 12105–12134 (2023). 37871288 10.1021/acs.chemrev.3c00305 PMC10636741

[8] Jiang, F. et al. Self-healable and stretchable perovskite-elastomer gas-solid triboelectric nanogenerator for gesture recognition and gripper sensing. *Sci. Adv.* **10**, eadq5778 (2024).

[9] Du, Y., Wang, Z. L., Chu, D., Amaratunga, G. & Wei, D. Iontronics for adaptive and flexible pressure sensing. *Iontronics* **2**, 13 (2026).

[10] Sun, Z. et al. Moving toward human-like perception and sensation systems—from integrated intelligent systems to decentralized smart devices. *SmartSys* **1**, e4 (2025).

[11] Liu, T. et al. Liquid–solid triboelectric probes for real-time monitoring of sucrose fluid status. *Adv. Funct. Mater.* **33**, 2304321 (2023).

[12] Zhang, J., Lin, S. & Wang, Z. L. Triboelectric nanogenerator array as a probe for in situ dynamic mapping of interface charge transfer at a liquid–solid contacting. *ACS Nano* **17**, 1646–1652 (2023). 10.1021/acsnano.2c11633 36602519

[13] Wu, Y., Liu, Y., Zhu, C., Kong, X.-Y. & Wen, L. Olfactory-inspired nanofluidic sensor: molecular recognition and transport in confined space. *Iontronics* **2**, 14 (2026).

[14] Liu, J. et al. Nonaqueous contact-electro-chemistry via triboelectric charge. *J. Am. Chem. Soc.* **146**, 31574–31584 (2024). 39527749 10.1021/jacs.4c09318

[15] Gan, T. et al. Unveiling Janus chemical processes in contact-electro-chemistry through oxygen reduction reactions. *J. Am. Chem. Soc.* **147**, 25407–25416 (2025). 40645920 10.1021/jacs.5c05124

[16] Wang, Z. et al. A contact-electro-catalysis process for producing reactive oxygen species by ball milling of triboelectric materials. *Nat. Commun.* **15**, 757 (2024). 38272926 10.1038/s41467-024-45041-4 PMC10810876

[17] Dong, X. et al. Regulating contact-electro-catalysis using polymer/metal janus composite catalysts. *J. Am. Chem. Soc.* **146**, 28110–28118 (2024). 10.1021/jacs.4c07446 39353155

[18] Li, S. et al. A green approach to induce and steer chemical reactions using inert solid dielectrics. *Nano Energy* **122**, 109286 (2024).

[19] Wang, Z. et al. Contact-electro-catalysis for the degradation of organic pollutants using pristine dielectric powders. *Nat. Commun.* **13**, 130 (2022). 35013271 10.1038/s41467-021-27789-1 PMC8748705

[20] Chen, B. et al. Water–solid contact electrification causes hydrogen peroxide production from hydroxyl radical recombination in sprayed microdroplets. *Proc. Natl. Acad. Sci. USA* **119**, e2209056119 (2022). 35914139 10.1073/pnas.2209056119 PMC9371641

[21] Li, H. et al. A contact-electro-catalytic cathode recycling method for spent lithium-ion batteries. *Nat. Energy* **8**, 1137–1144 (2023).

[22] Li, S., Liu, J., Wang, Z. L. & Wei, D. Mechano-driven chemical reactions. *Green Energy Environ.* **10**, 937–966 (2025).

[23] Li, X., Wang, Z. L. & Wei, D. Iontronic logic control driven by dynamic electrical double layer regulation. *Iontronics* **1**, 2 (2025).

[24] Li, J., Yin, J. & Guo, W. Hydrovoltaic energy and intelligence: where ions meet electrons. *Iontronics* **1**, 3 (2025).

[25] McGregor, J.-M. et al. Organic electrolyte cations promote non-aqueous CO_2_ reduction by mediating interfacial electric fields. *Nat. Catal.* **8**, 79–91 (2025).

[26] Deng, B., Huang, M., Zhao, X., Mou, S. & Dong, F. Interfacial electrolyte effects on electrocatalytic CO_2_ reduction. *ACS Catal* **12**, 331–362 (2022).

[27] Malkani, A. S. et al. Understanding the electric and nonelectric field components of the cation effect on the electrochemical CO reduction reaction. *Sci. Adv.* **6**, eabd2569 (2020). 33158873 10.1126/sciadv.abd2569 PMC7673714

[28] Yang, J. et al. Switching reaction pathways of CO_2_ electroreduction by modulating cations in the electrochemical double layer. *Angew. Chem. Int. Ed.* **63**, e202410145 (2024). 10.1002/anie.202410145 38979674

[29] Wen, W. et al. Modulating the electrolyte microenvironment in electrical double layer for boosting electrocatalytic nitrate reduction to ammonia. *Angew. Chem. Int. Ed.* **63**, e202408382 (2024). 10.1002/anie.202408382 38806407

[30] Yan, M., Yang, R., Liu, C., Gao, Y. & Zhang, B. In situ probing the anion-widened anodic electric double layer for enhanced faradaic efficiency of chlorine-involved reactions. *J. Am. Chem. Soc.* **147**, 6698–6706 (2025). 39953989 10.1021/jacs.4c16173

[31] Itoh, Y. et al. Electric double-layer synthesis of a spongelike, lightweight reticular membrane. *Science* **389**, 73–77 (2025). 40608921 10.1126/science.adq0782

[32] Nam, I., Lee, J. K., Nam, H. G. & Zare, R. N. Abiotic production of sugar phosphates and uridine ribonucleoside in aqueous microdroplets. *Proc. Natl. Acad. Sci. USA* **114**, 12396–12400 (2017). 29078402 10.1073/pnas.1714896114 PMC5703324

[33] Mohajer, M. A. et al. Spontaneous formation of urea from carbon dioxide and ammonia in aqueous droplets. *Science* **388**, 1426–1430 (2025). 40570122 10.1126/science.adv2362

[34] Wang, Y. et al. Catalyst-free nitrogen fixation by microdroplets through a radical-mediated disproportionation mechanism under ambient conditions. *J. Am. Chem. Soc.* **147**, 2756–2765 (2025). 39797796 10.1021/jacs.4c15514

[35] Li, K. et al. Room-temperature catalyst-free ammonia decomposition for hydrogen production on water microdroplets. *J. Am. Chem. Soc.* **147**, 20417–20425 (2025). 40460349 10.1021/jacs.5c02072

[36] Bose, S., Mofidfar, M. & Zare, R. N. Direct conversion of N_2_ and air to nitric acid in gas–water microbubbles. *J. Am. Chem. Soc.* **146**, 27964–27971 (2024). 39315452 10.1021/jacs.4c11899

[37] Li, J. et al. Methane C(sp^3^)–H bond activation by water microbubbles. *Chem. Sci.* **15**, 17026–17031 (2024). 10.1039/d4sc05773b PMC11446311 39364074

[38] Yan, X. et al. Bubble energy generator. *Sci. Adv.* **8**, eabo7698 (2022). 35749507 10.1126/sciadv.abo7698 PMC9232101

[39] Shi, L. et al. Water structure and electric fields at the interface of oil droplets. *Nature* **640**, 87–93 (2025). 40108466 10.1038/s41586-025-08702-y PMC12335212

[40] Li, H. et al. Free radicals generated in perfluorocarbon–water (liquid–liquid) interfacial contact electrification and their application in cancer therapy. *J. Am. Chem. Soc.* **146**, 12087–12099 (2024). 38647488 10.1021/jacs.4c02149

[41] Camara, C. G., Escobar, J. V., Hird, J. R. & Putterman, S. J. Correlation between nanosecond X-ray flashes and stick–slip friction in peeling tape. *Nature* **455**, 1089–1092 (2008).

[42] Gao, X. et al. Peeling tape produces strong electric fields via stick–slip friction that drive chemical reactions. *Proc. Natl. Acad. Sci. USA* **122**, e2510504122 (2025). 40560609 10.1073/pnas.2510504122 PMC12232728

[43] Dai, Y., Wang, Z. & Zare, R. N. Unlocking the electrochemical functions of biomolecular condensates. *Nat. Chem. Biol.* **20**, 1420–1433 (2024). 39327453 10.1038/s41589-024-01717-y

[44] Chai, Y., Dai, W., Wu, G., Guan, N. & Li, L. Confinement in a Zeolite and Zeolite Catalysis. *Acc. Chem. Res.* **54**, 2894–2904 (2021). 34165959 10.1021/acs.accounts.1c00274

[45] Zhang, Y. et al. Advances in metastable phase catalysis with thermodynamic-kinetic adaptability. *Mater. Today* **90**, 598–628 (2025).

[46] haik, S., Danovich, D., Kalita, S. & Dubey, K. D. Oriented electric fields─universal catalysts. *Acc. Chem. Res.* **58**, 3071–3080 (2025). 40998307 10.1021/acs.accounts.5c00508 PMC12509264

[47] Didenko, Y. T. & Suslick, K. S. The energy efficiency of formation of photons, radicals and ions during single-bubble cavitation. *Nature* **418**, 394–397 (2002). 12140551 10.1038/nature00895

[48] Suslick, K. S. Sonochemistry. *Science* **247**, 1439–1445 (1990). 17791211 10.1126/science.247.4949.1439

[49] Wang, Z., Dong, X., Tang, W. & Wang, Z. L. Contact-electro-catalysis (CEC). *Chem. Soc. Rev.* **53**, 4349–4373 (2024). 38619095 10.1039/d3cs00736g

[50] Chung, M. et al. Direct propylene epoxidation via water activation over Pd-Pt electrocatalysts. *Science* **383**, 49–55 (2024). 38175873 10.1126/science.adh4355

[51] Yang, J. et al. CO_2_-mediated organocatalytic chlorine evolution under industrial conditions. *Nature* **617**, 519–523 (2023). 37198309 10.1038/s41586-023-05886-z

[52] Wang, J. et al. Engineering the coordination environment of Ir single atoms with surface titanium oxide amorphization for superior chlorine evolution reaction. *J. Am. Chem. Soc.* **146**, 11152–11163 (2024). 10.1021/jacs.3c13834 38498303

[53] Liu, Y. et al. Electrosynthesis of chlorine from seawater-like solution through single-atom catalysts. *Nat. Commun.* **14**, 2475 (2023). 37120624 10.1038/s41467-023-38129-w PMC10148798

[54] Lim, T. et al. Atomically dispersed Pt–N4 sites as efficient and selective electrocatalysts for the chlorine evolution reaction. *Nat. Commun.* **11**, 412 (2020). 31964881 10.1038/s41467-019-14272-1 PMC6972710

[55] Moreno-Hernandez, I. A., Brunschwig, B. S. & Lewis, N. S. Crystalline nickel, cobalt, and manganese antimonates as electrocatalysts for the chlorine evolution reaction. *Energy Environ. Sci.* **12**, 1241–1248 (2019).

[56] Choi, S. et al. A Reflection on sustainable anode materials for electrochemical chloride oxidation. *Adv. Mater.* **35**, 2300429 (2023). 10.1002/adma.202300429 36897816

[57] Karlsson, R. K. B. & Cornell, A. Selectivity between oxygen and chlorine evolution in the chlor-alkali and chlorate processes. *Chem. Rev.* **116**, 2982–3028 (2016). 26879761 10.1021/acs.chemrev.5b00389

[58] Gao, R. et al. Photoelectrochemical production of disinfectants from seawater. *Nat. Sustain.* **8**, 672–681 (2025).

[59] Zhao, S. et al. Selective electrosynthesis of chlorine disinfectants from seawater. *Nat. Sustain.* **7**, 148–157 (2024).

[60] Fang, Y. & Wang, X. Metal-Free Boron-Containing Heterogeneous Catalysts. *Angew. Chem. Int. Ed.* **56**, 15506–15518 (2017). 10.1002/anie.201707824 28872779

[61] Feidenhans’l, A. A. et al. Precious metal free hydrogen evolution catalyst design and application. *Chem. Rev.* **124**, 5617–5667 (2024). 38661498 10.1021/acs.chemrev.3c00712 PMC11082907

[62] Fair, G. M., Morris, J. C., Chang, S. L., Weil, I. & Burden, R. P. The behavior of chlorine as a water disinfectant. *J. Am. Water Works Assoc.* **40**, 1051–1061 (1948). 18145494

[63] Li, X., Wang, Z. L. & Wei, D. Scavenging energy and information through dynamically regulating the electrical double layer. *Adv. Funct. Mater.* **34**, 2405520 (2024).

[64] Lin, S., Xu, L., Chi Wang, A. & Wang, Z. L. Quantifying electron-transfer in liquid-solid contact electrification and the formation of electric double-layer. *Nat. Commun.* **11**, 399 (2020). 31964882 10.1038/s41467-019-14278-9 PMC6972942

[65] Li, W. et al. Contact-electro-catalysis for direct oxidation of methane under ambient conditions. *Angew. Chem. Int. Ed.* **63**, e202403114 (2024). 10.1002/anie.202403114 38488787

[66] Wang, Z. et al. A generalized approach for enhancing contact-electro-catalysis of oxides in a broad temperature range by fluorination. *Nat. Commun.* **16**, 11035 (2025). 41372136 10.1038/s41467-025-66002-5 PMC12695925

[67] Berbille, A. et al. Mechanism for generating H2O2 at water-solid interface by contact-electrification. *Adv. Mater.* **35**, 2304387 (2023). 10.1002/adma.202304387 37487242

[68] Dong, X. et al. Investigations on the contact-electro-catalysis under various ultrasonic conditions and using different electrification particles. *Nano Energy* **99**, 107346 (2022).

[69] Lee, J. K. et al. Spontaneous generation of hydrogen peroxide from aqueous microdroplets. *Proc. Natl. Acad. Sci. USA* **116**, 19294–19298 (2019). 31451646 10.1073/pnas.1911883116 PMC6765303

[70] Mehrgardi, M. A., Mofidfar, M. & Zare, R. N. Sprayed water microdroplets are able to generate hydrogen peroxide spontaneously. *J. Am. Chem. Soc.* **144**, 7606–7609 (2022). 35451822 10.1021/jacs.2c02890

[71] Minakata, D., Kamath, D. & Maetzold, S. Mechanistic insight into the reactivity of chlorine-derived radicals in the aqueous-phase UV–chlorine advanced oxidation process: quantum mechanical calculations. *Environ. Sci. Technol.* **51**, 6918–6926 (2017). 28541663 10.1021/acs.est.7b00507

[72] Xu, C. et al. Contact-electro-chemistry induced by flow electrification in dielectric tubes. *Nano Energy* **134**, 110526 (2025).

[73] Xu, C. et al. Contact-electro-luminescence triggered by triboelectric charge. *Chem. Eng. J.* **501**, 157754 (2024).

[74] Shen, Z. et al. Exhaustive denitrification via chlorine oxide radical reactions for urea based on a novel photoelectrochemical cell. *Water Res.* **170**, 115357 (2020). 31812812 10.1016/j.watres.2019.115357

[75] Xiao, H. et al. New insight of electrogenerated H_2_O_2_ into oxychlorides inhibition and decontamination promotion: from radical to nonradical pathway during anodic oxidation of high Cl^−^-laden wastewater process. *J. Hazard. Mater.* **486**, 136948 (2025). 39721481 10.1016/j.jhazmat.2024.136948

[76] Zhao, Y. et al. The process of free radical generation in contact electrification at solid-liquid interface. *Nano Energy* **112**, 108464 (2023).

[77] Koppenol, W. H. & Liebman, J. F. The oxidizing nature of the hydroxyl radical. A comparison with the ferryl ion (FeO2+). *J. Phys. Chem.* **88**, 99–101 (1984).

[78] Chen, Y., Miller, C. J. & Waite, T. D. pH dependence of hydroxyl radical, ferryl, and/or ferric peroxo species generation in the heterogeneous fenton process. *Environ. Sci. Technol.* **56**, 1278–1288 (2022). 34965094 10.1021/acs.est.1c05722

[79] Wojnárovits, L. & Takács, E. Rate constants of dichloride radical anion reactions with molecules of environmental interest in aqueous solution: a review. *Environ. Sci. Pollut. Res.* **28**, 41552–41575 (2021). 10.1007/s11356-021-14453-w PMC8354983 34086177

[80] Gligorovski, S., Strekowski, R., Barbati, S. & Vione, D. Environmental implications of hydroxyl radicals (•OH). *Chem. Rev.* **115**, 13051–13092 (2015). 26630000 10.1021/cr500310b

[81] Nightingale, E. R. Jr Phenomenological theory of ion solvation. Effective radii of hydrated ions. *J. Phys. Chem.* **63**, 1381–1387 (1959).

[82] Ringe, S. et al. Understanding cation effects in electrochemical CO_2_ reduction. *Energy Environ. Sci.* **12**, 3001–3014 (2019).

[83] Wang, Z. L. & Wang, A. C. On the origin of contact-electrification. *Mater. Today* **30**, 34–51 (2019).

