# Supplementary material for: A triboelectric radical generation route to chlorine disinfectants from brine
Source: Nat Commun. 2026 Jul 7;17:8377. doi: 10.1038/s41467-026-75108-3 (PMC13473123; doi:10.1038/s41467-026-75108-3)
Supplement: Supplementary file 1 [file 41467_2026_75108_MOESM1_ESM.pdf]

Supplementary Information for

A triboelectric radical generation route to chlorine disinfectants from brine

Han Qian[1,2,†], Jianming Liu[1,2,†], Ning Wu[1,2,†], Shaoxin Li[1,2,3], Xinhong Song[1], Feiyao Yang[1], Morten Willatzen[1,2], Jiajia Shao[1,2], Vishnu Shankar[4], Daping Chu[5], Richard N. Zare[3,*], Zhong Lin Wang[1,*], Di Wei[1,5,*]

Affiliations:

[1]Beijing Institute of Nanoenergy and Nanosystems, Chinese Academy of Sciences, Beijing 101400, China

[2]School of Nanoscience and Engineering, University of Chinese Academy of Sciences, Beijing 100049, China

[3]Department of Chemistry, Stanford University, Stanford, CA, 94305, USA

[4]Program in Immunology, Stanford University School of Medicine, Stanford, CA, 94305, USA

[5]Centre for Photonic Devices and Sensors, University of Cambridge, UK

[†]These authors contributed equally: Han Qian, Jianming Liu, Ning Wu.

*Corresponding author. E-mail: zare@stanford.edu; zlwang@binn.cas.cn; weidi@binn.cas.cn

The PDF file includes:

Supplementary Figure S1 to S32

Supplementary Table S1 to S4

Supplementary Figures and Tables

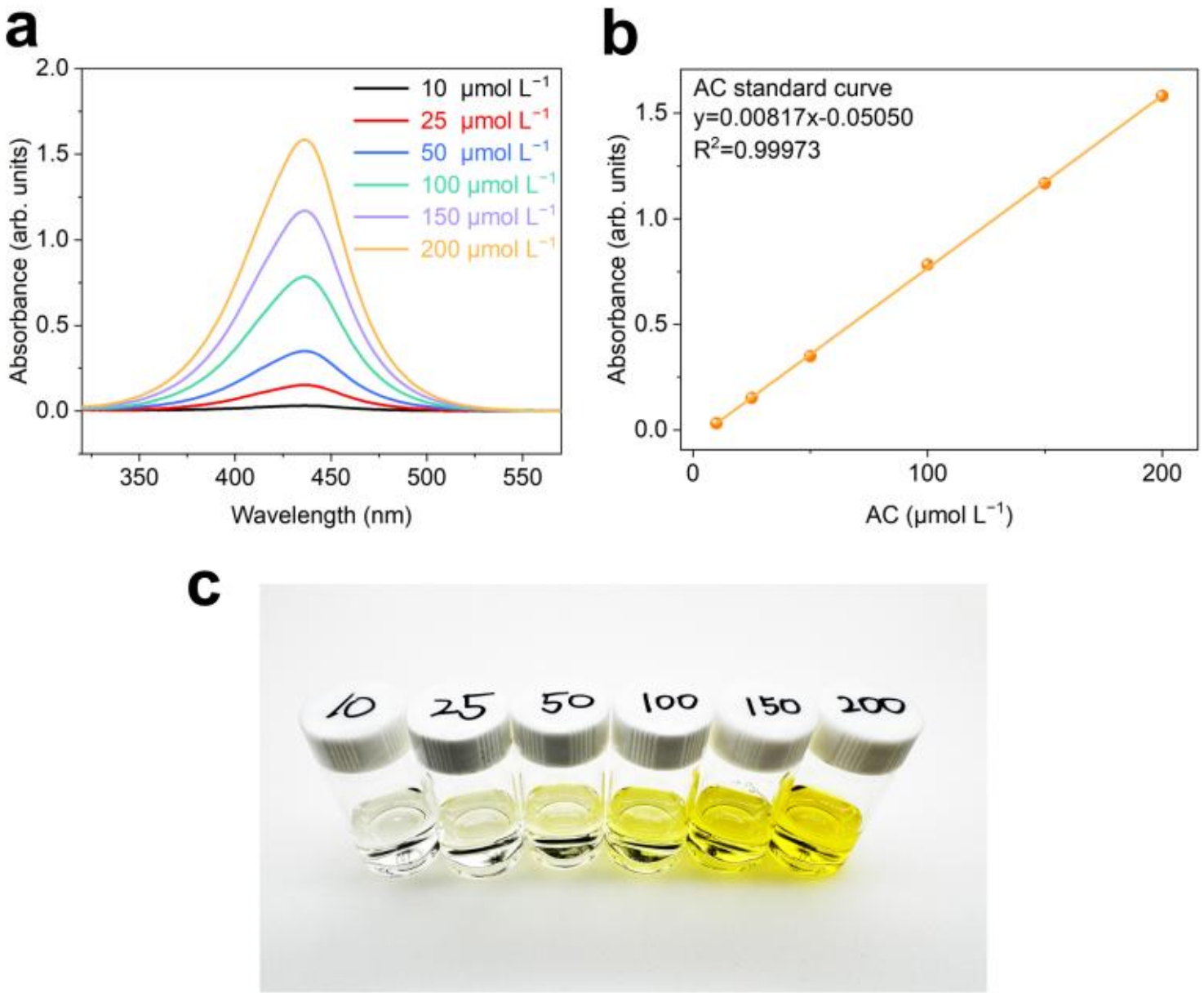

Supplementary Figure 1. The concentration-absorbance UV-Vis calibration curves of ClO^- using different HOCl concentrations as standards. a, b, UV-Vis curves of assays with ClO^- (**a**) and linear fitting results of the calibration curve (**b**). **c,** Digital image of standard solutions of various HOCl concentrations under active chlorine chromogenic agent.

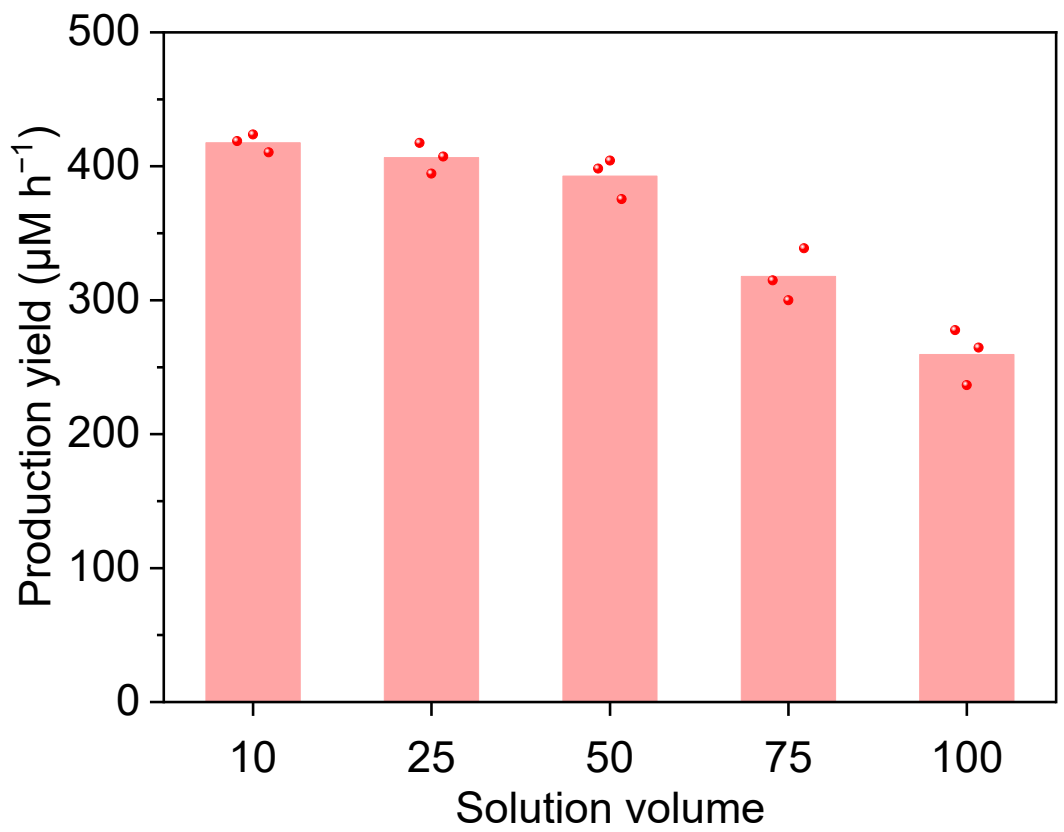

Supplementary Figure 2. Comparison of active chlorine production yields at different solution volumes of natural seawater solution. All data in bar charts are presented as mean values from three independent experiments (n = 3), with dots indicating individual measurements.

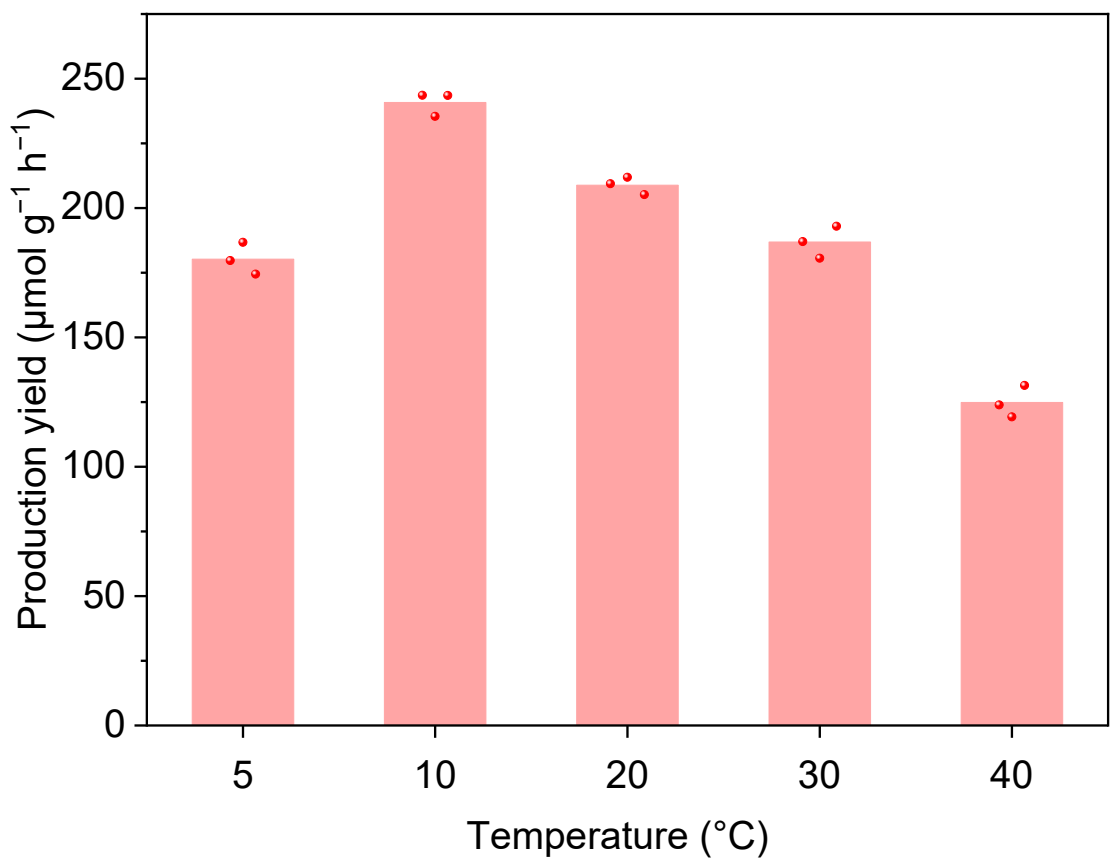

Supplementary Figure 3. Comparison of active chlorine production yields at different temperatures of natural seawater solution. All data in bar charts are presented as mean values from three independent experiments (n = 3), with dots indicating individual measurements.

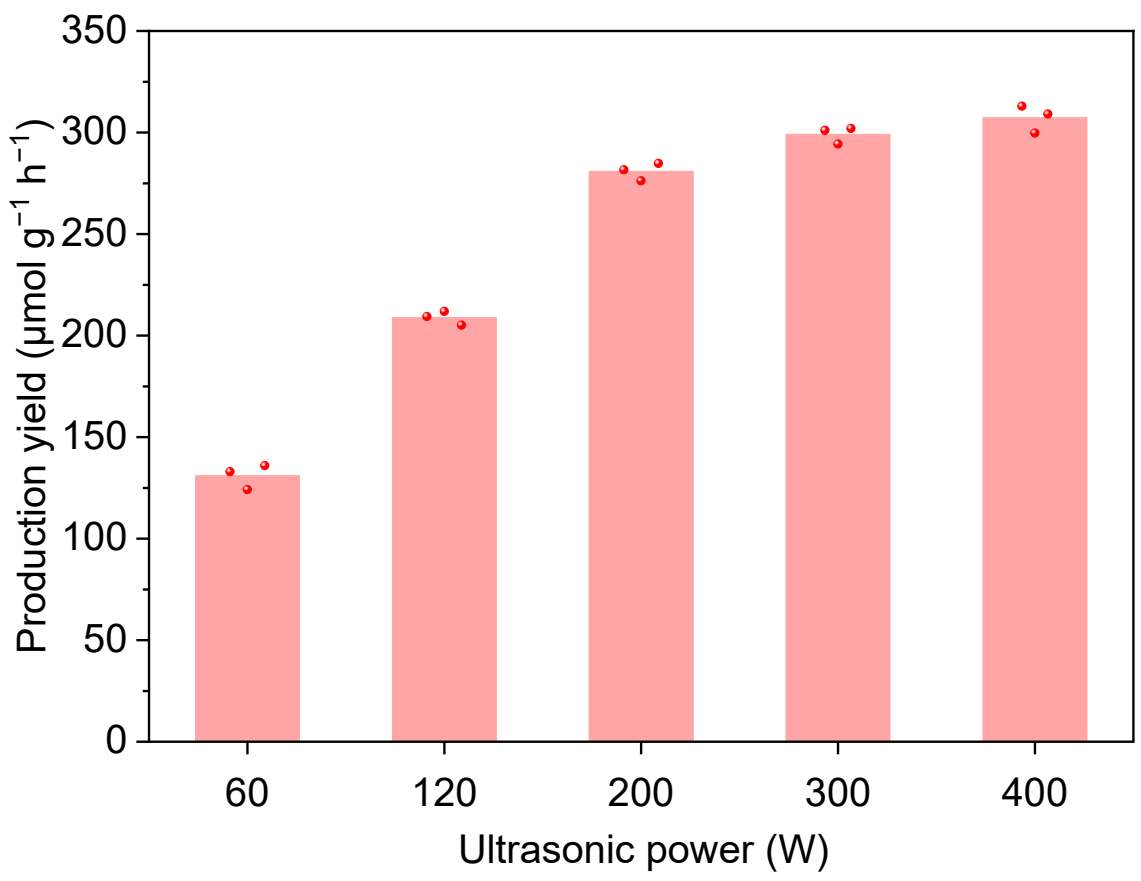

Supplementary Figure 4. Comparison of active chlorine production yields at different ultrasonic powers of natural seawater solution. All data in bar charts are presented as mean values from three independent experiments (n = 3), with dots indicating individual measurements.

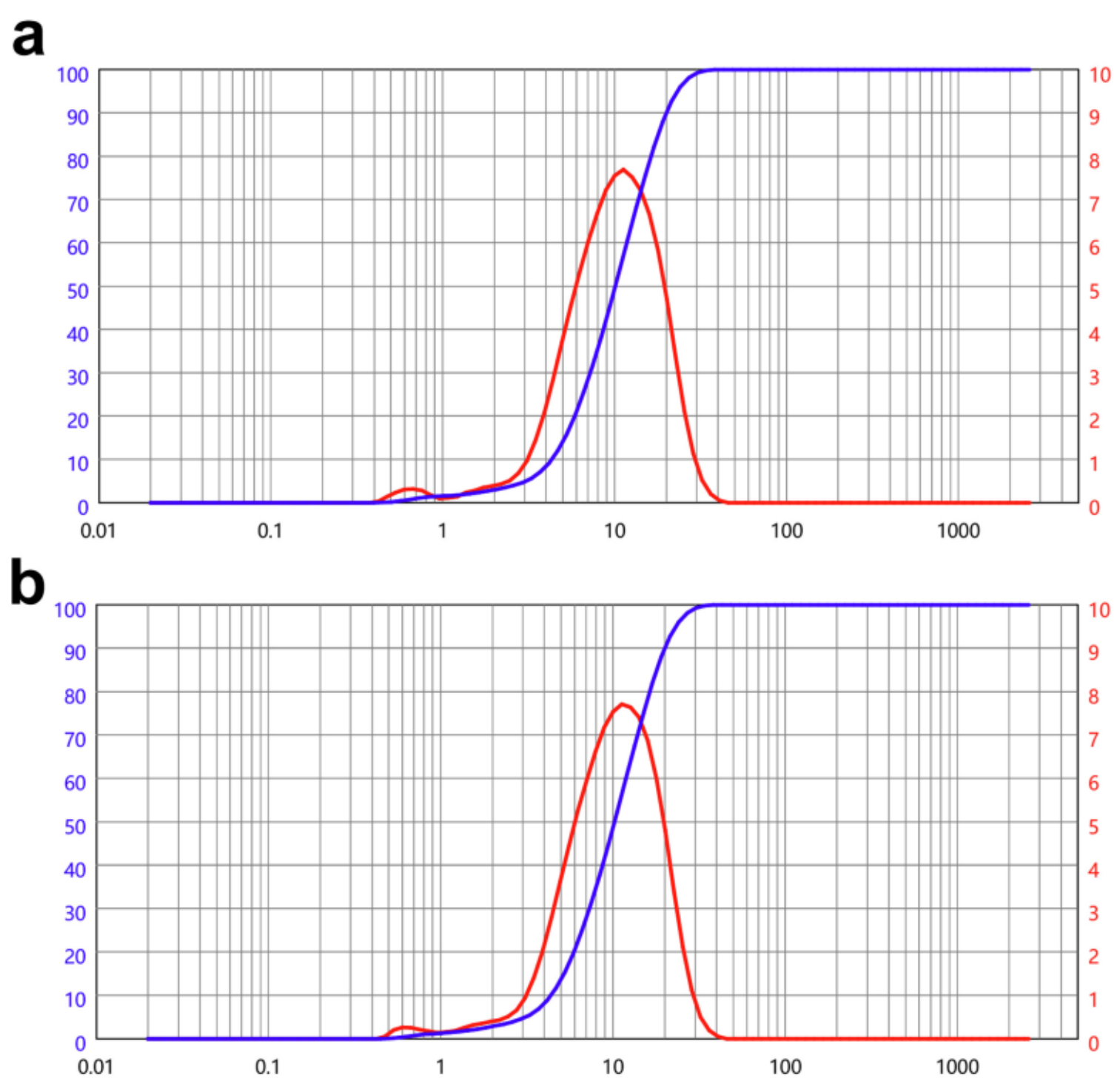

Supplementary Figure 5. The particle size distribution of FEP powder (a) before and (b) after the reaction (x-axis: μm, y-axis: %).

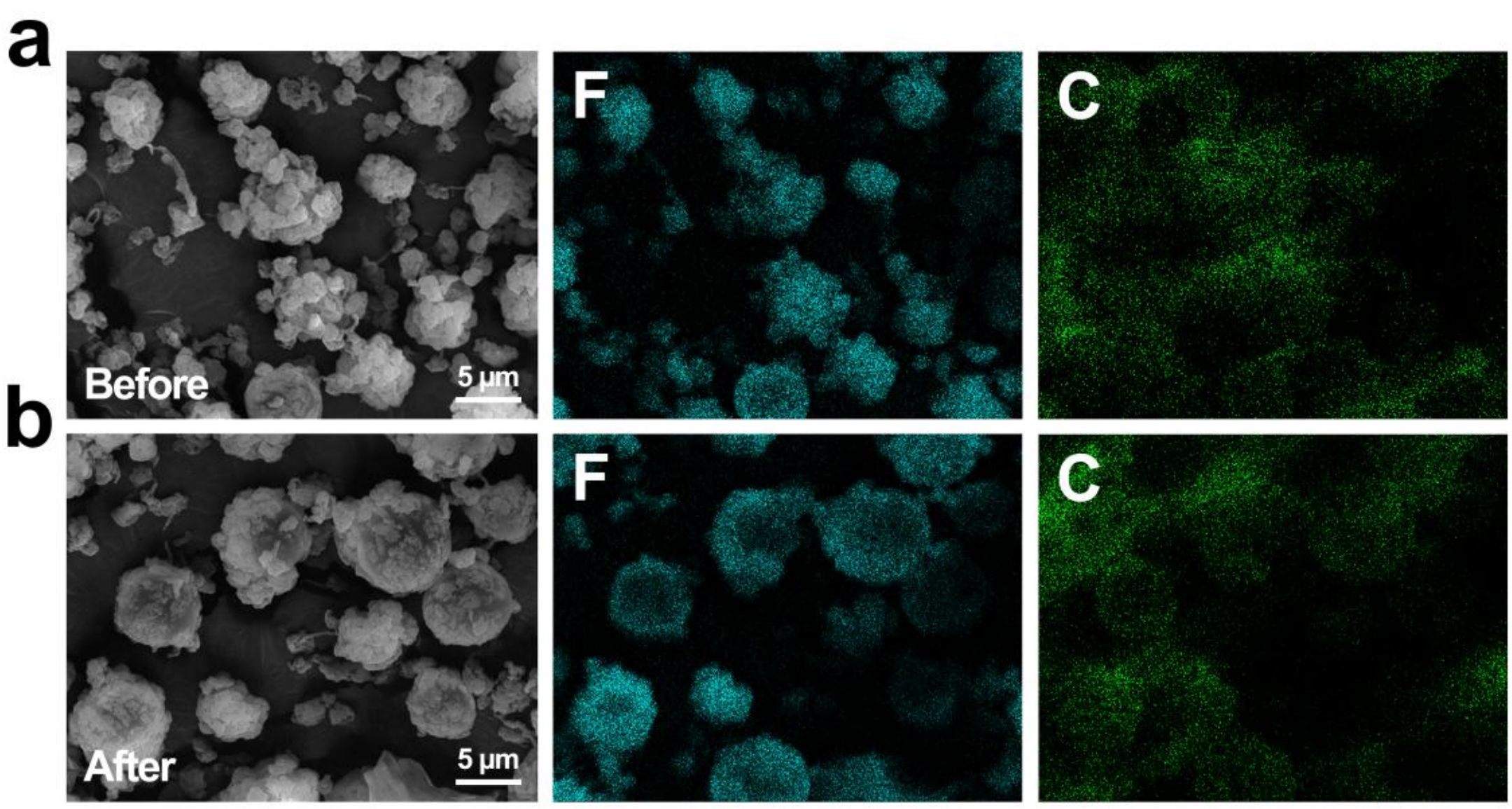

Supplementary Figure 6. Morphological characterization and corresponding energy-dispersive X-ray spectroscopy (EDS) elemental mapping of the FEP powder (a) before and (b) after the reaction.

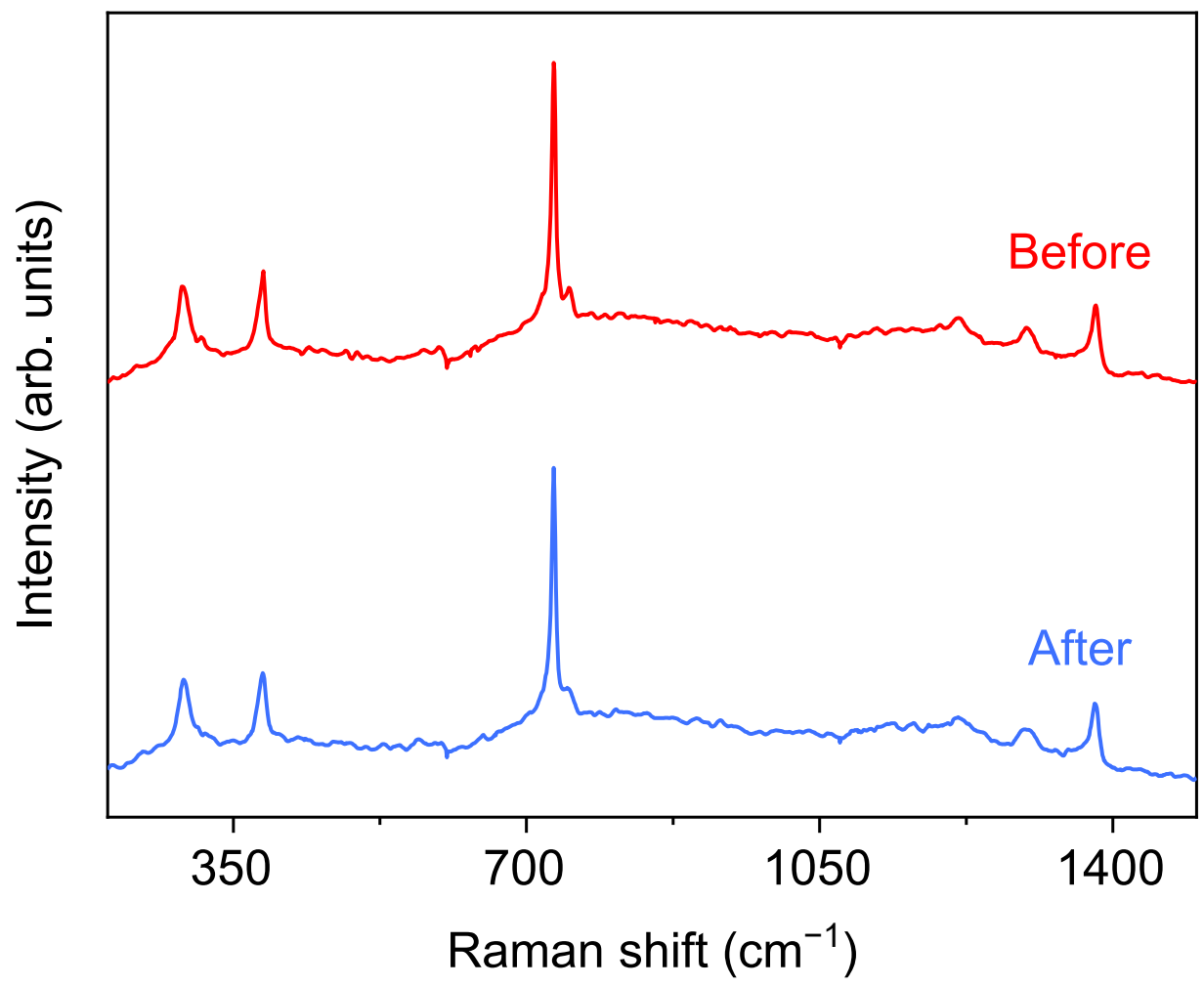

Supplementary Figure 7. Raman spectra of FEP powder before (red) and after (blue) the reaction.

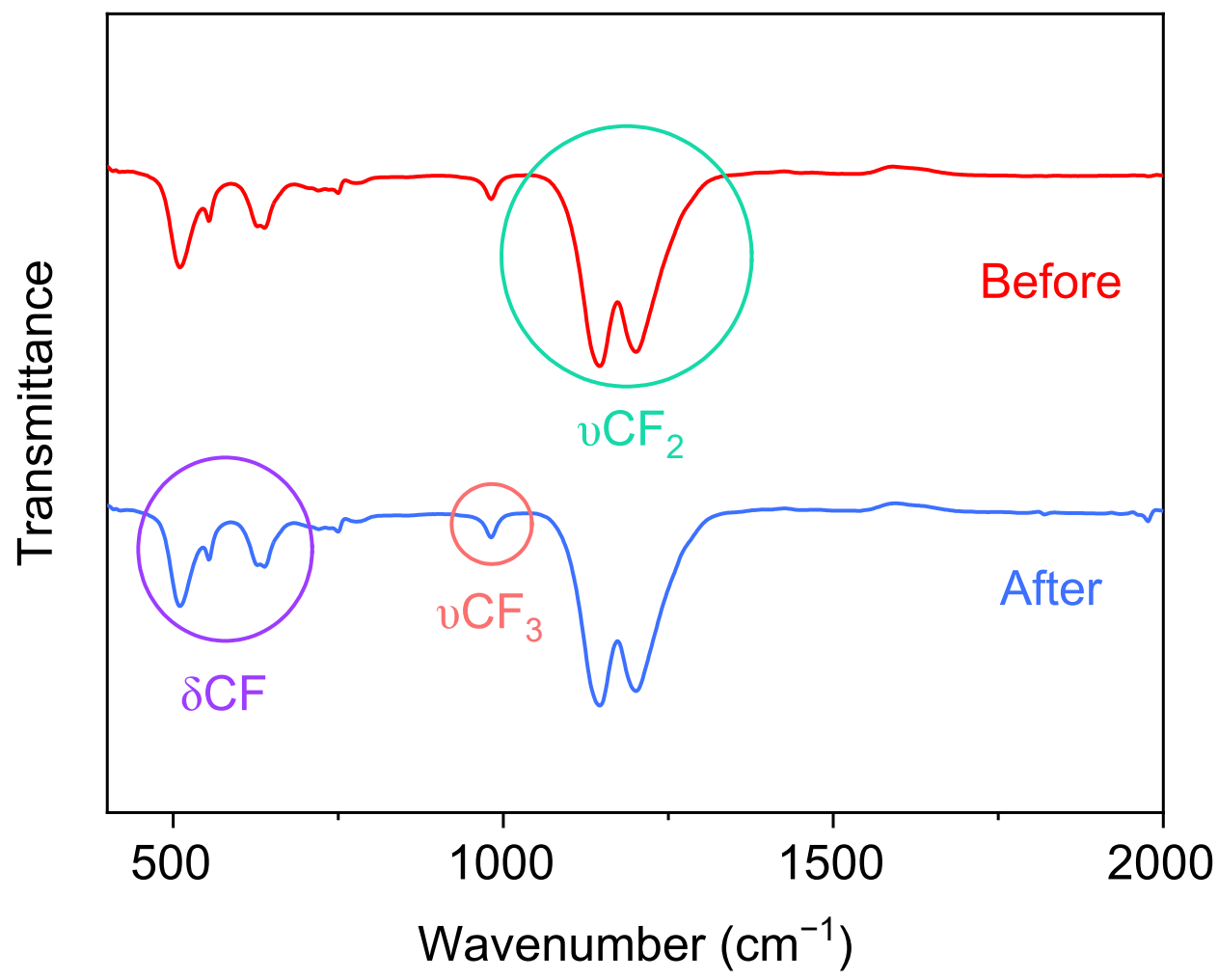

Supplementary Figure 8. FTIR spectra of FEP powder before (red) and after (blue) the reaction.

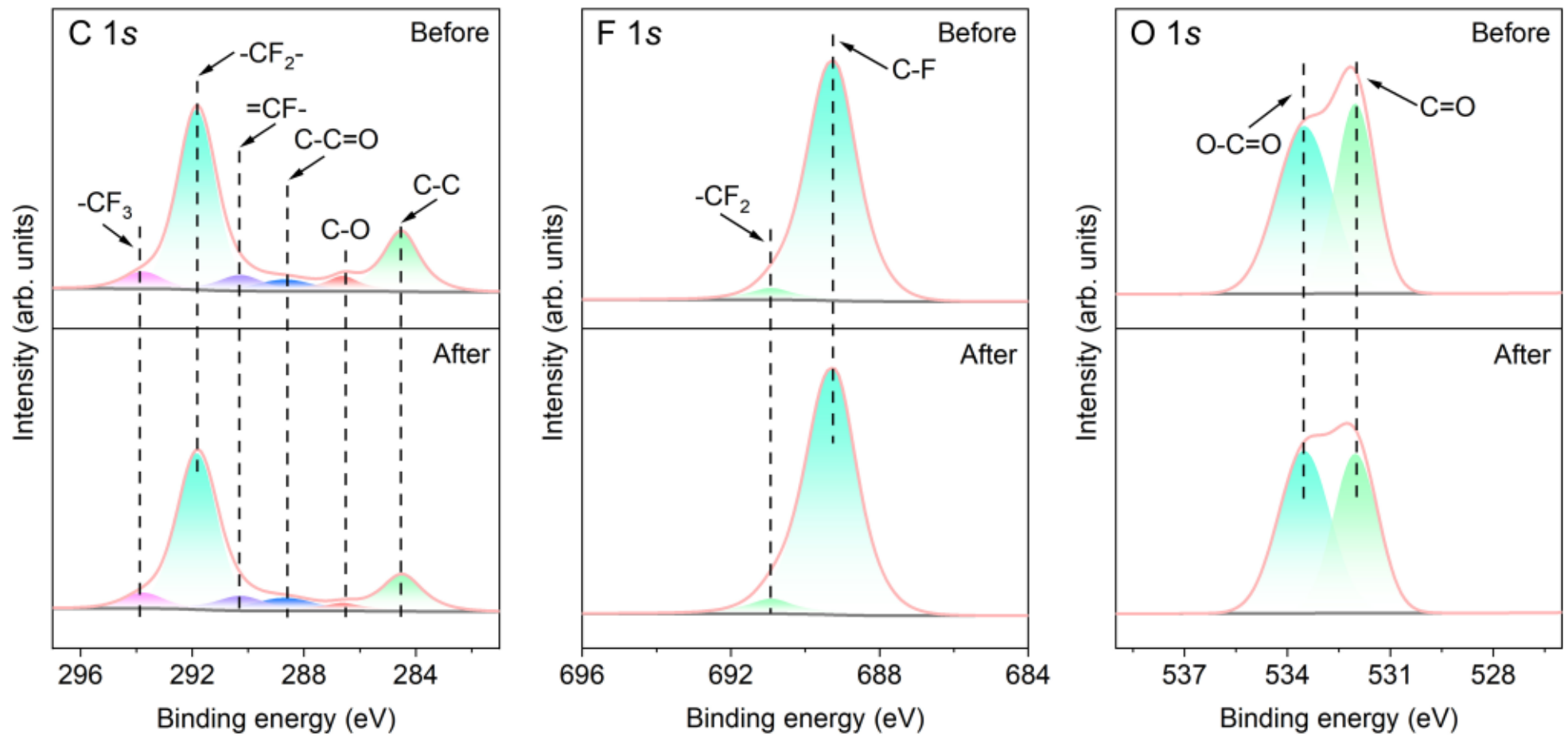

Supplementary Figure 9. ***XPS*** **spectra of FEP powder before and after the reaction.**

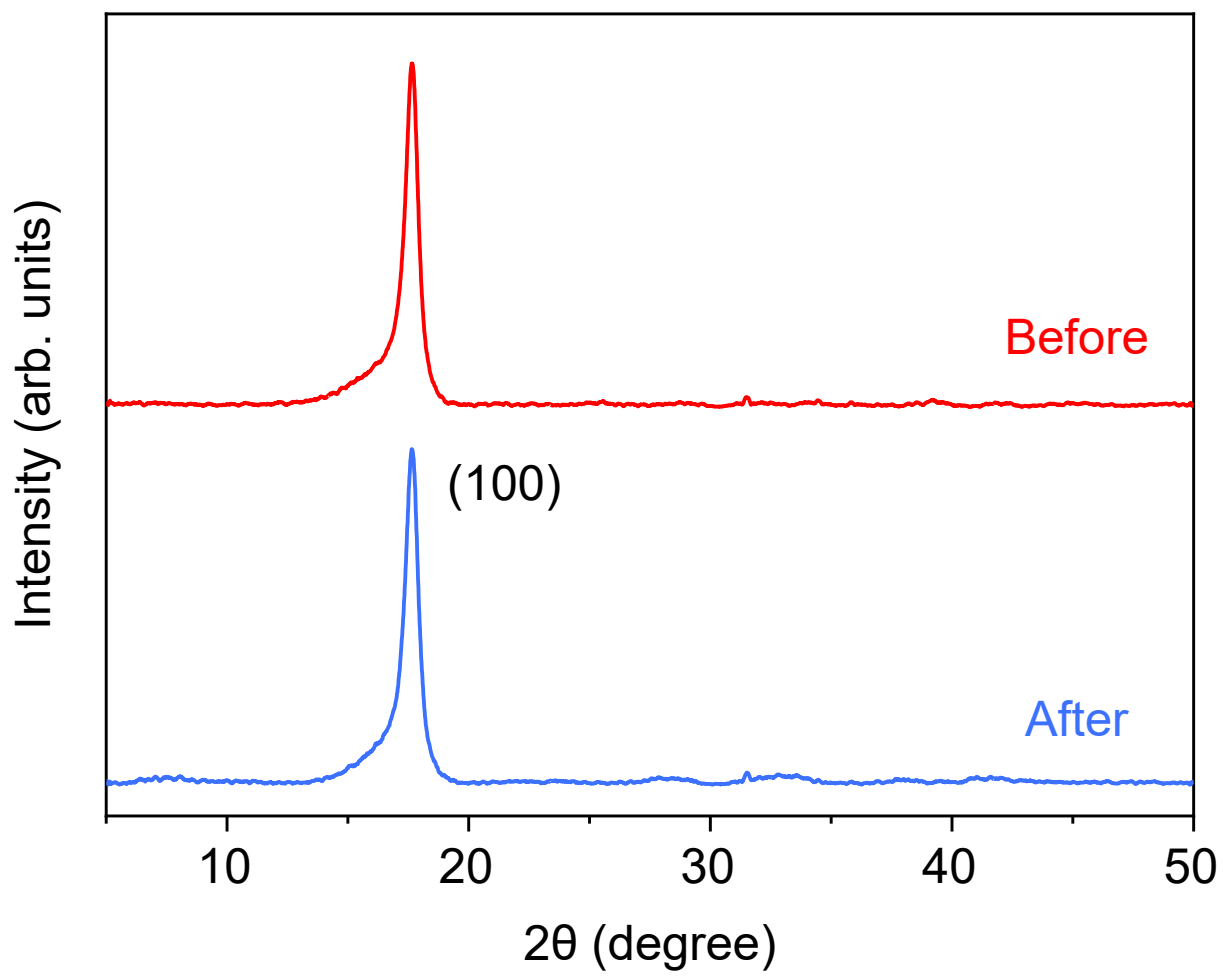

Supplementary Figure 10. XRD patterns of FEP powder before (red) and after (blue) the reaction.

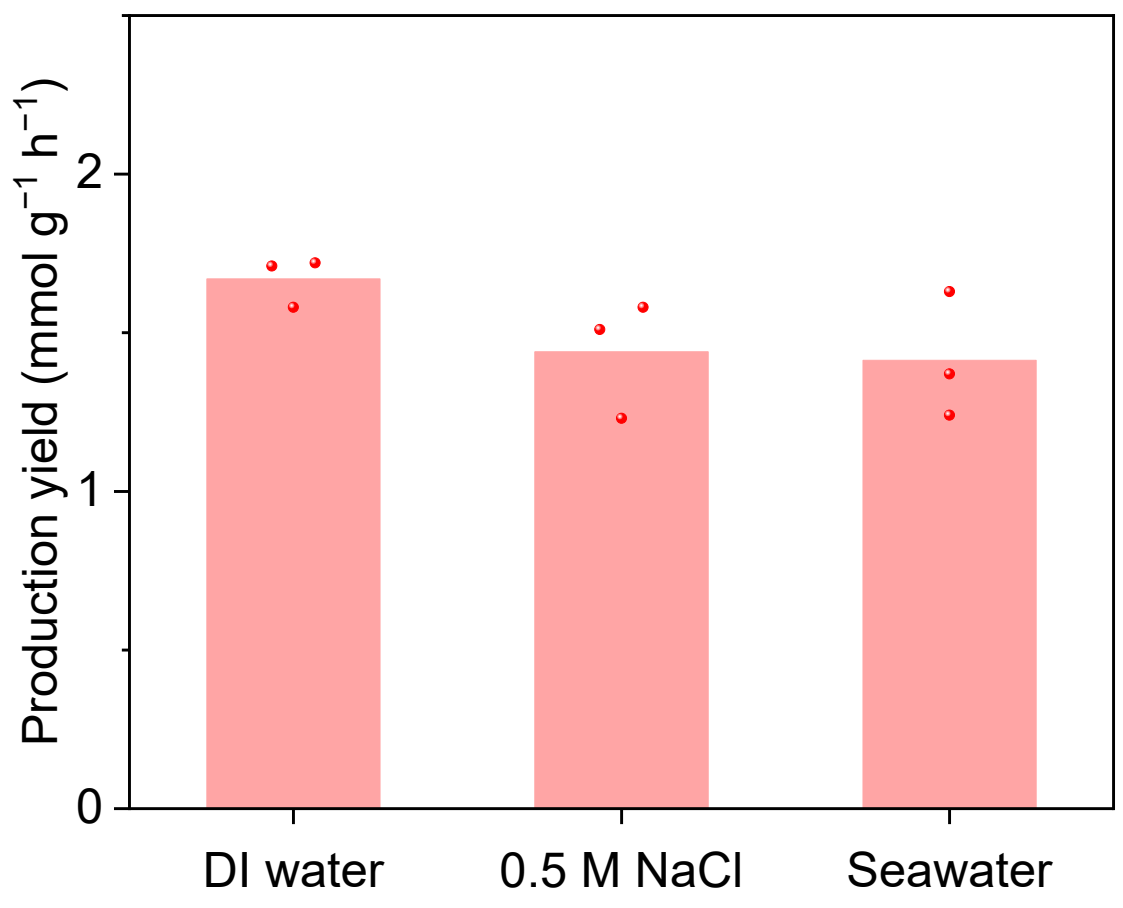

Supplementary Figure 11. Comparison of H_2 production yields under natural seawater and NaCl solutions. All data in bar charts are presented as mean values from three independent experiments (n = 3), with dots indicating individual measurements.

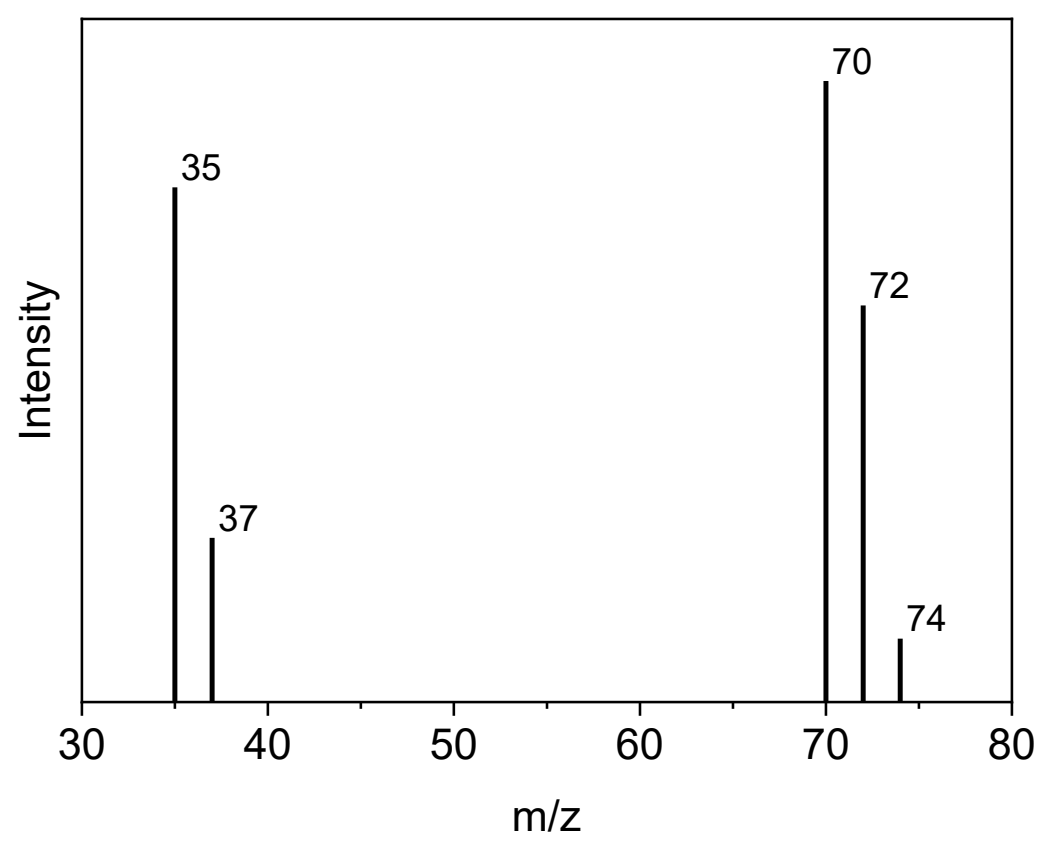

Supplementary Figure 12. Mass spectrum of Cl_2 standard gas.

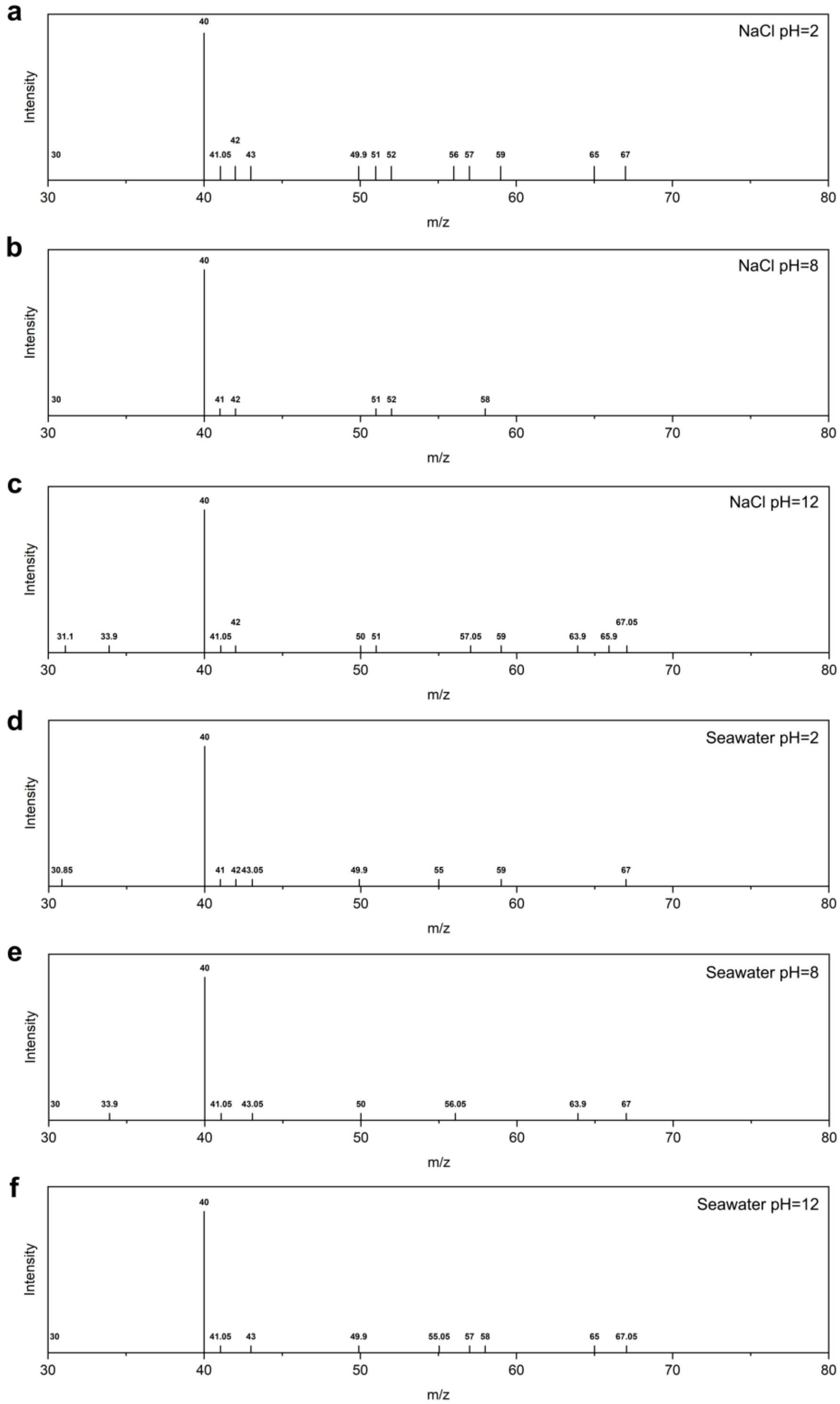

Supplementary Figure 13. Headspace gas chromatography-mass spectrometry (GC-MS) of gaseous and liquid products from the reaction system under different conditions. a, NaCl system at pH=2. **b,** NaCl system at pH=8. **c,** NaCl system at pH=12. **d,** Natural seawater system at pH=2. **e,** Natural seawater system at pH=8. **f,** Natural seawater system at pH=12.

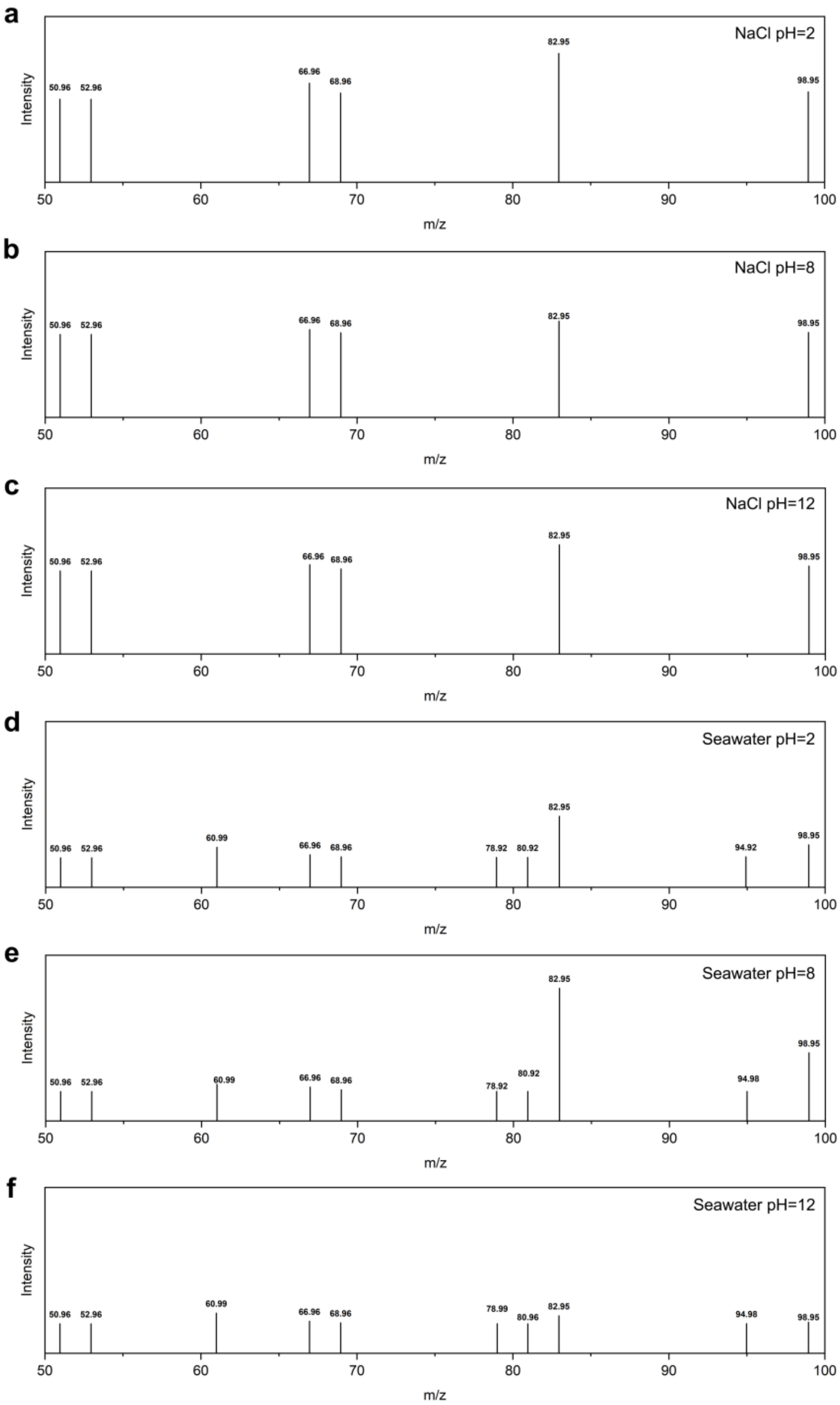

Supplementary Figure 14. Electrospray ionization mass spectrometry (ESI-MS) of liquid products from the reaction system under different conditions. a, NaCl system at pH=2. b, NaCl system at pH=8. c, NaCl system at pH=12. d, Natural seawater system at pH=2. e, Natural seawater system at pH=8. f, Natural seawater system at pH=12.

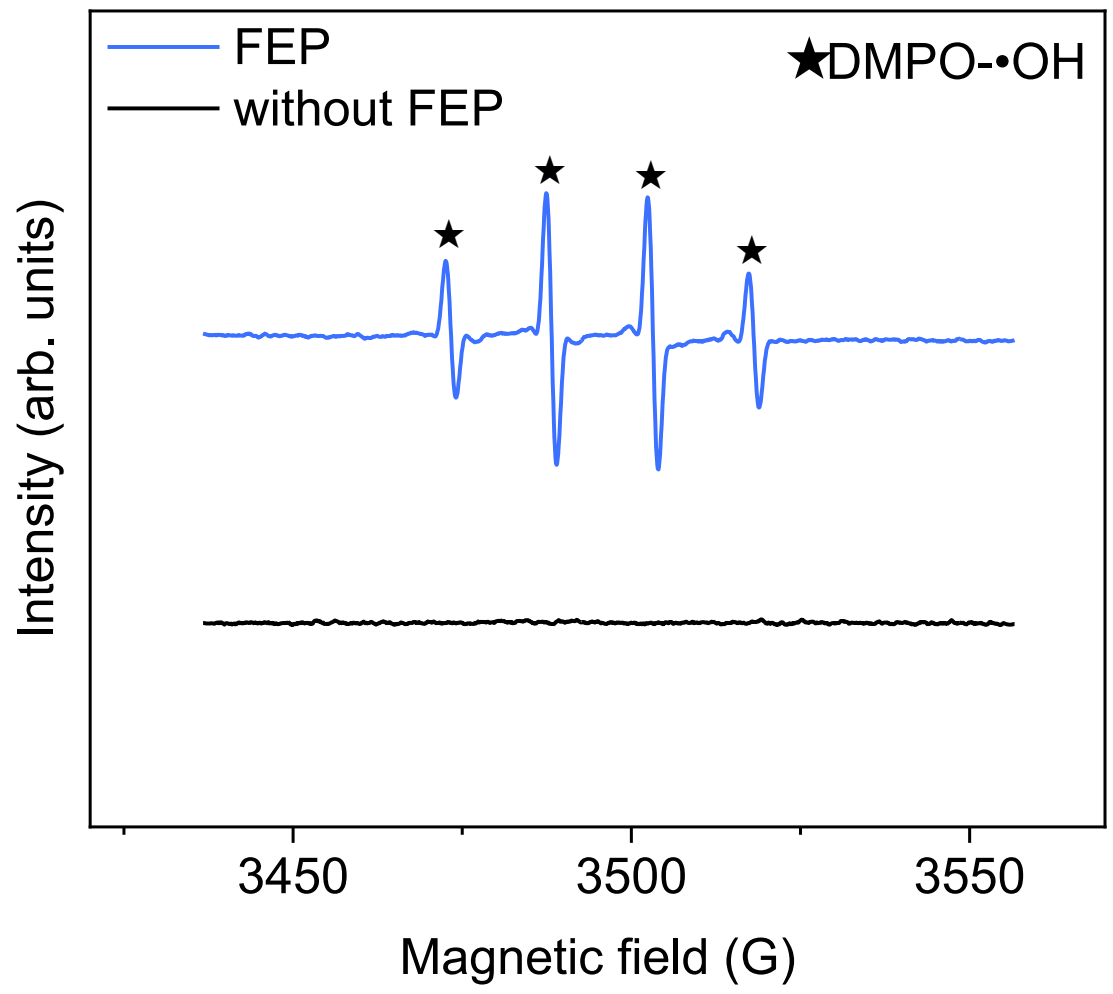

Supplementary Figure 15. EPR spectra of DMPO-•OH with and without FEP.

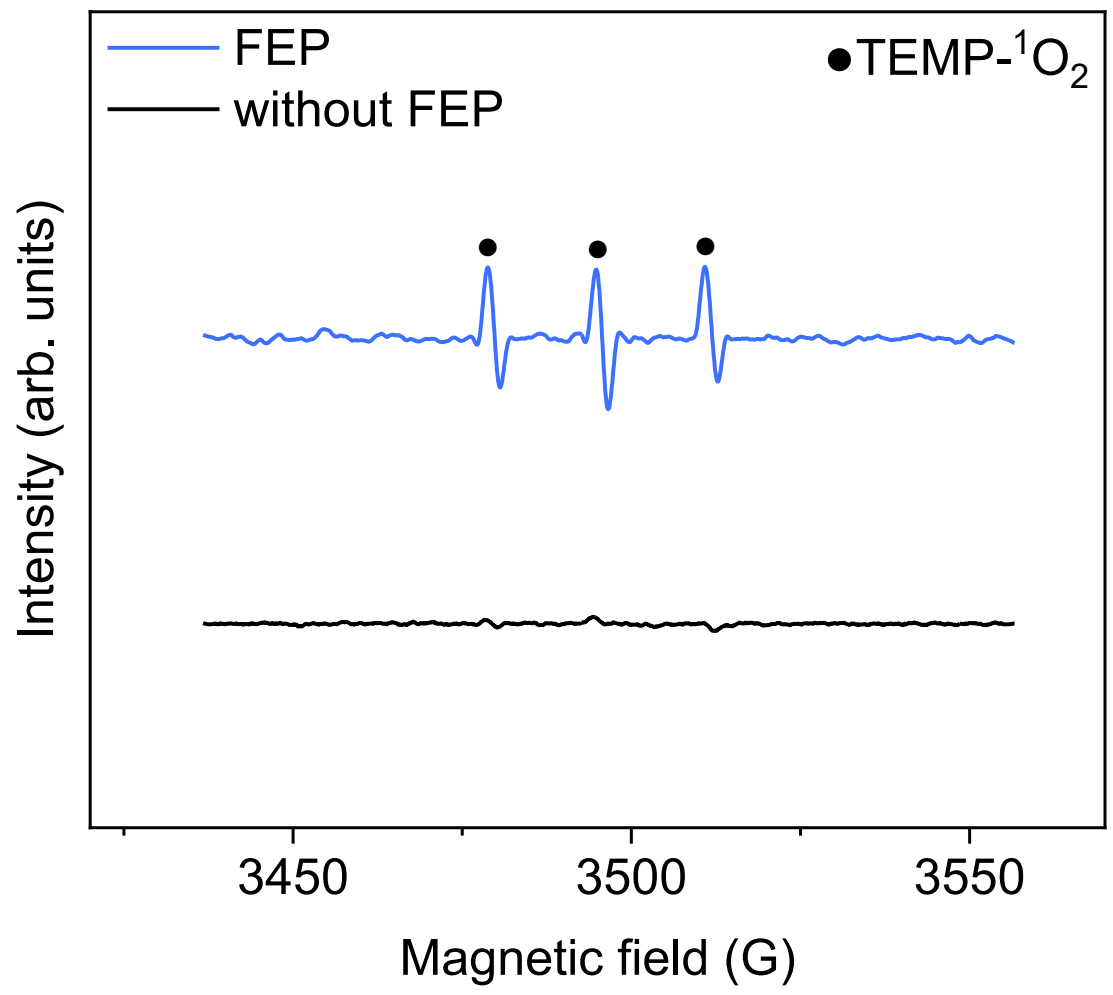

Supplementary Figure 16. EPR spectra of TEMP-$^1O_2$ with and without FEP.

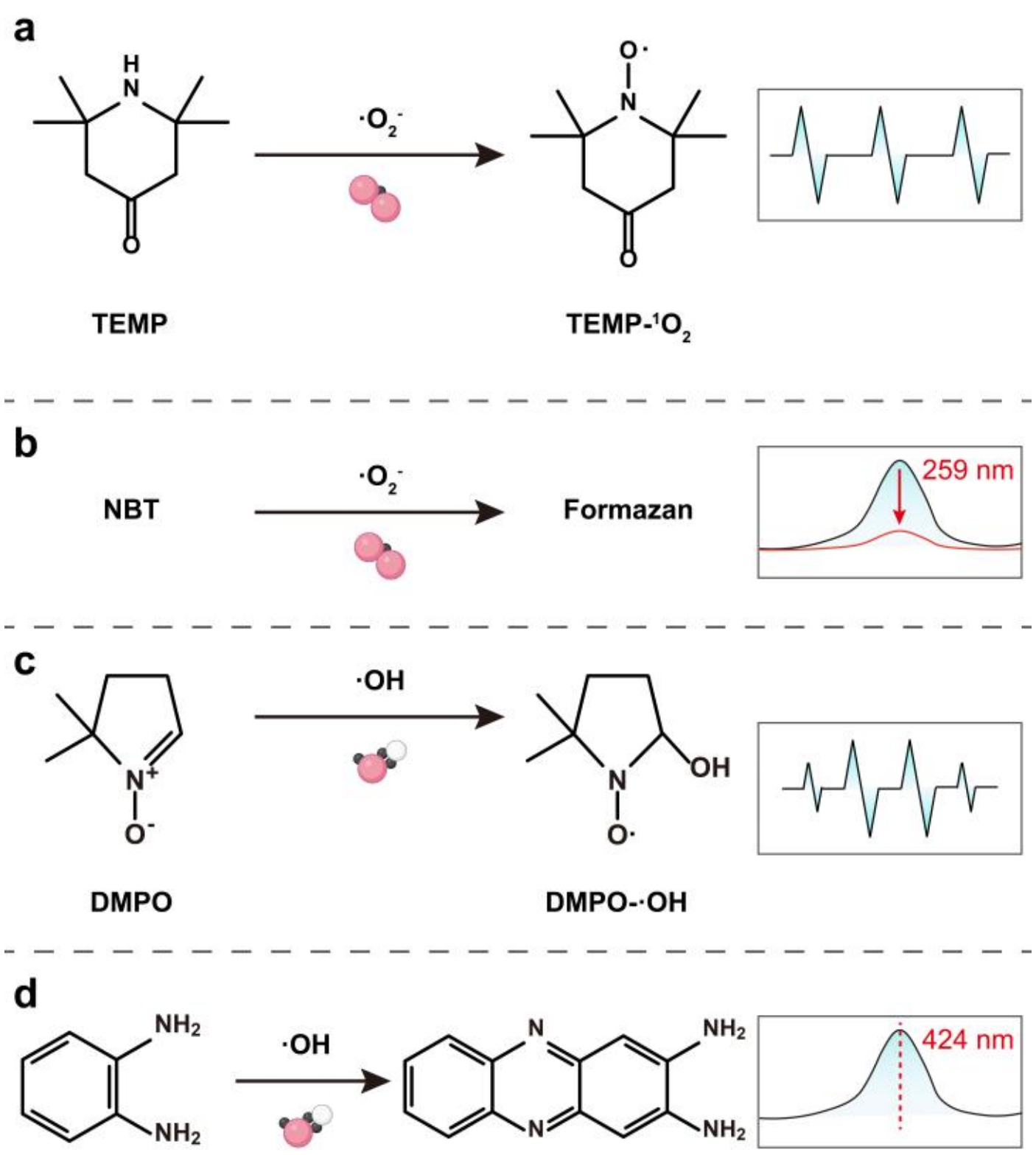

Supplementary Figure 17. Detection of the production of •O_2^- and •OH via EPR and spectrophotometry. a, TEMP reacts with $^1O_2$ to form the triplet characteristic peak. **b,** NBT shows a maximum absorption at 259 nm. **c,** DMPO reacts with •OH to form a quadruplet characteristic peak. **d,** OPD reacts with •OH and forms a maximum absorption at 424 nm.

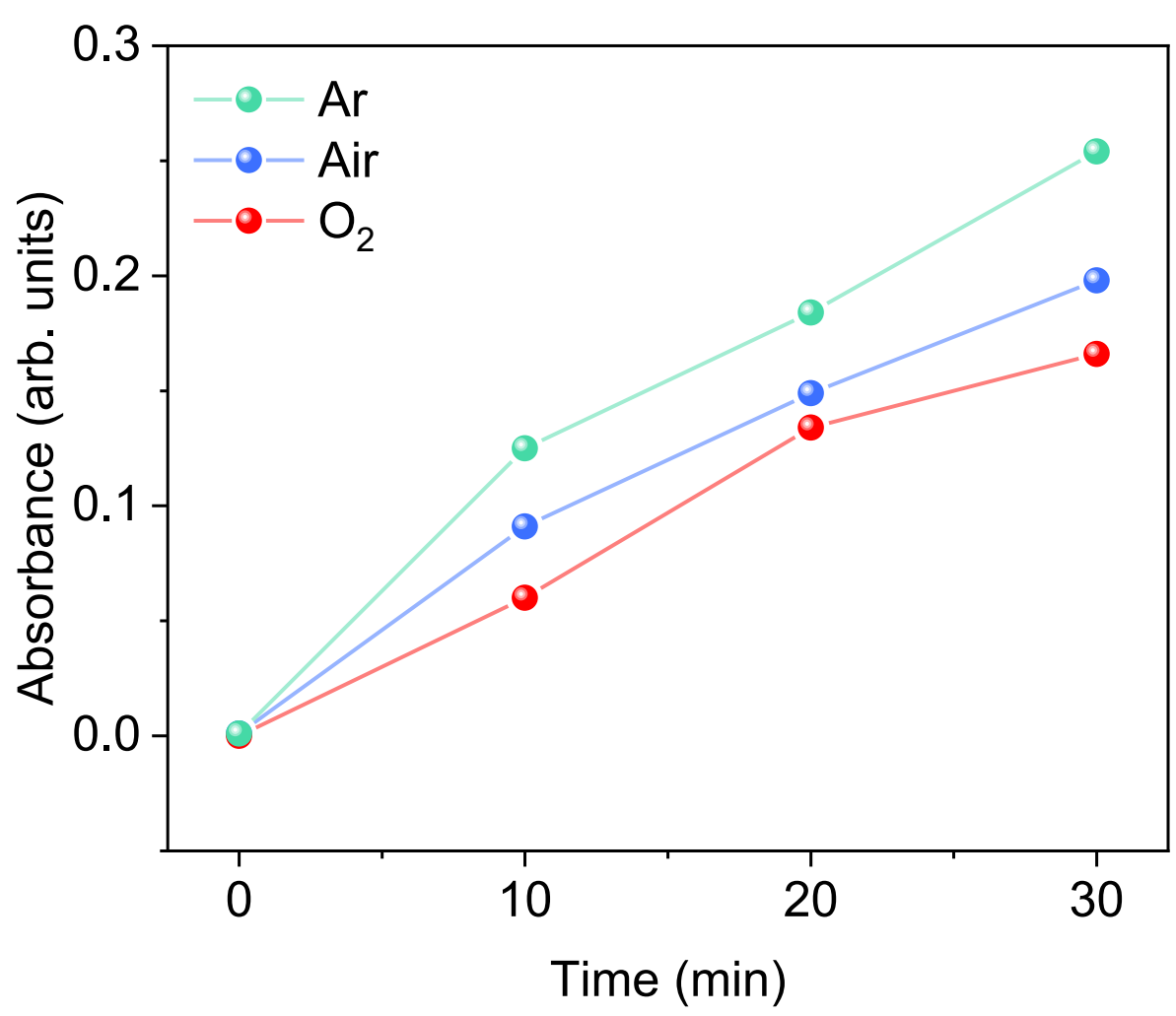

Supplementary Figure 18. Quantification of the amount of •OH using OPD under different atmospheres.

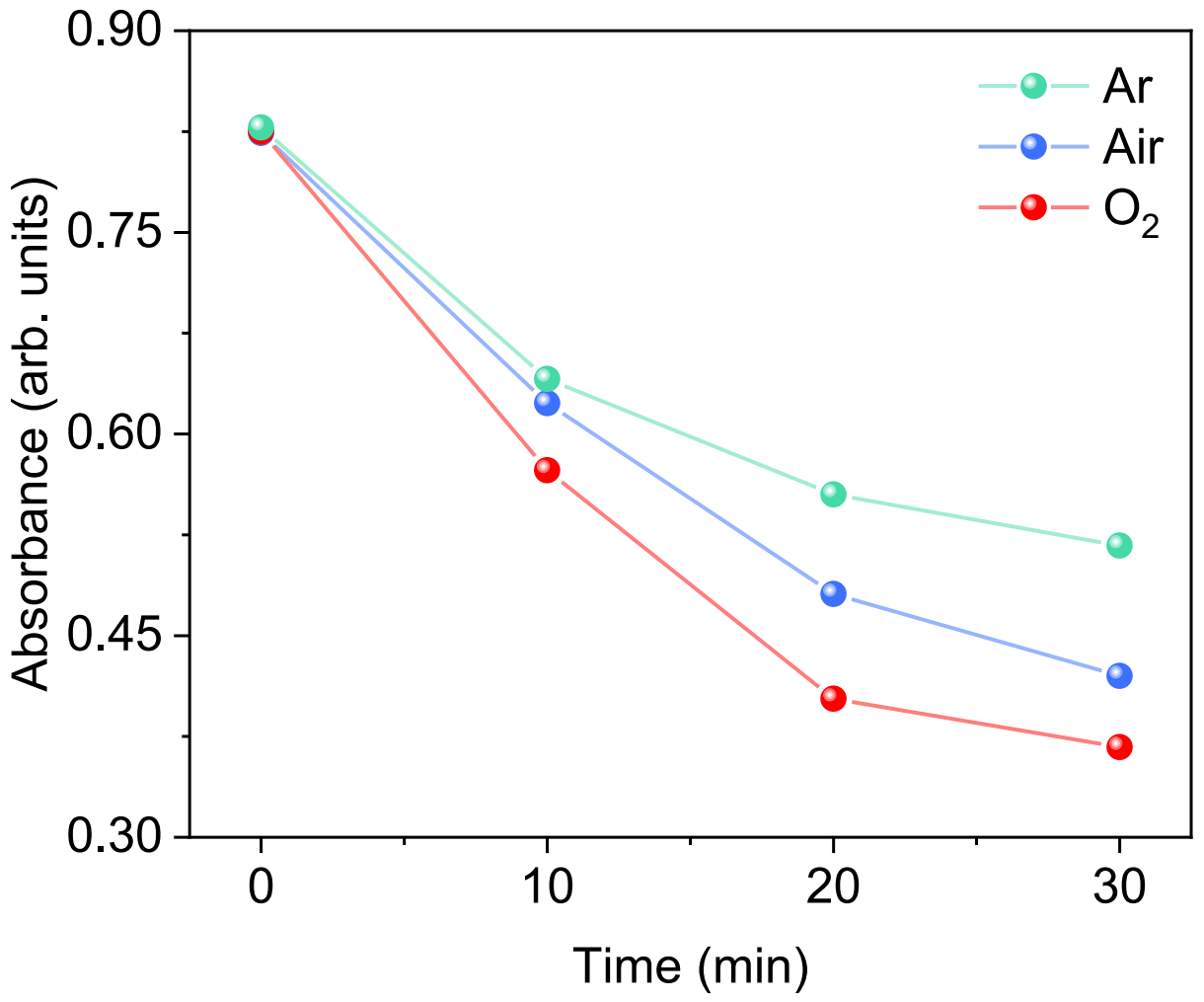

Supplementary Figure 19. Quantification of the amount of $\bullet O_2^-$ using NBT under different atmospheres.

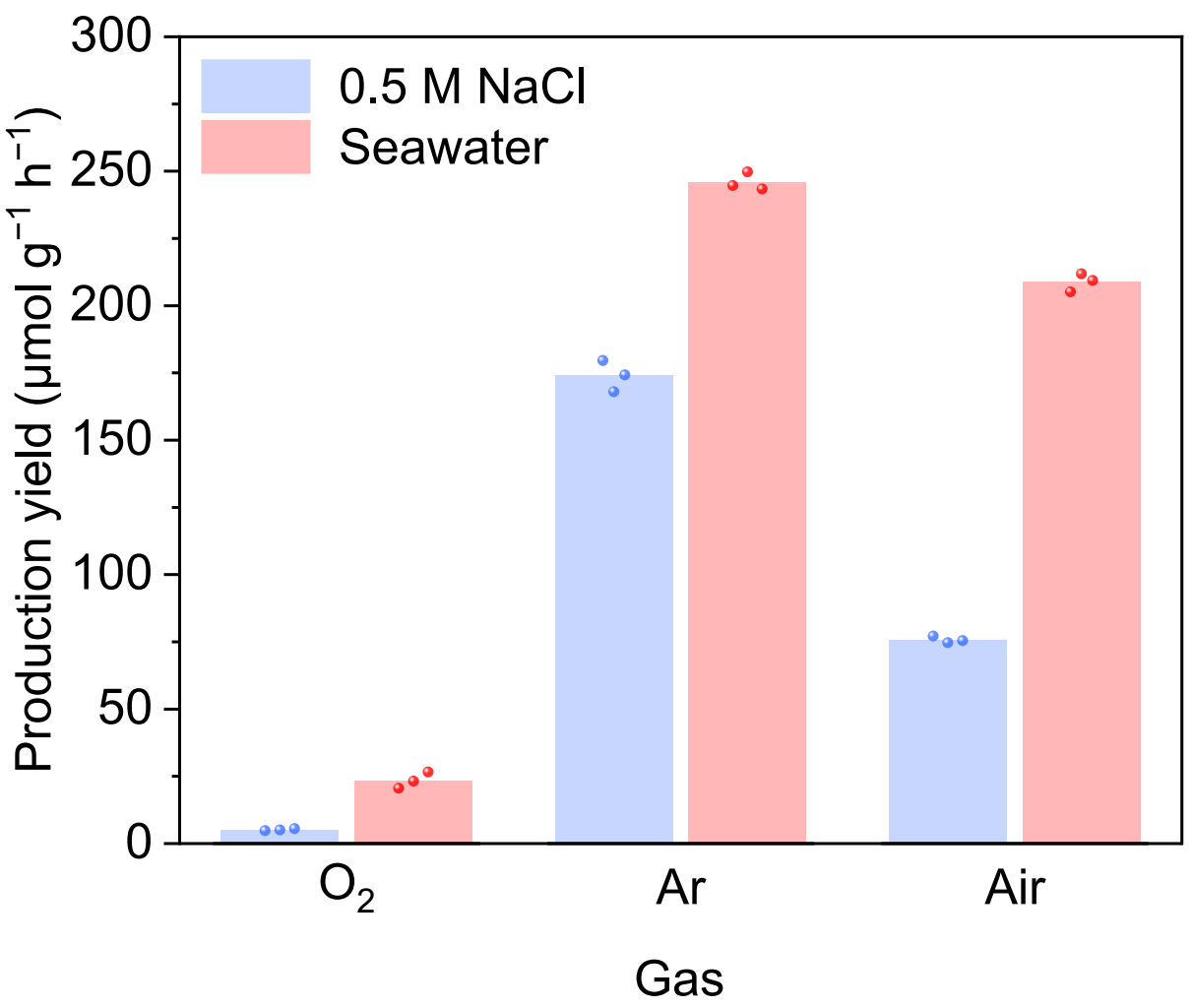

Supplementary Figure 20. Comparison of active chlorine production yields under different atmospheres of natural seawater and NaCl solutions. All data in bar charts are presented as mean values from three independent experiments (n = 3), with dots indicating individual measurements.

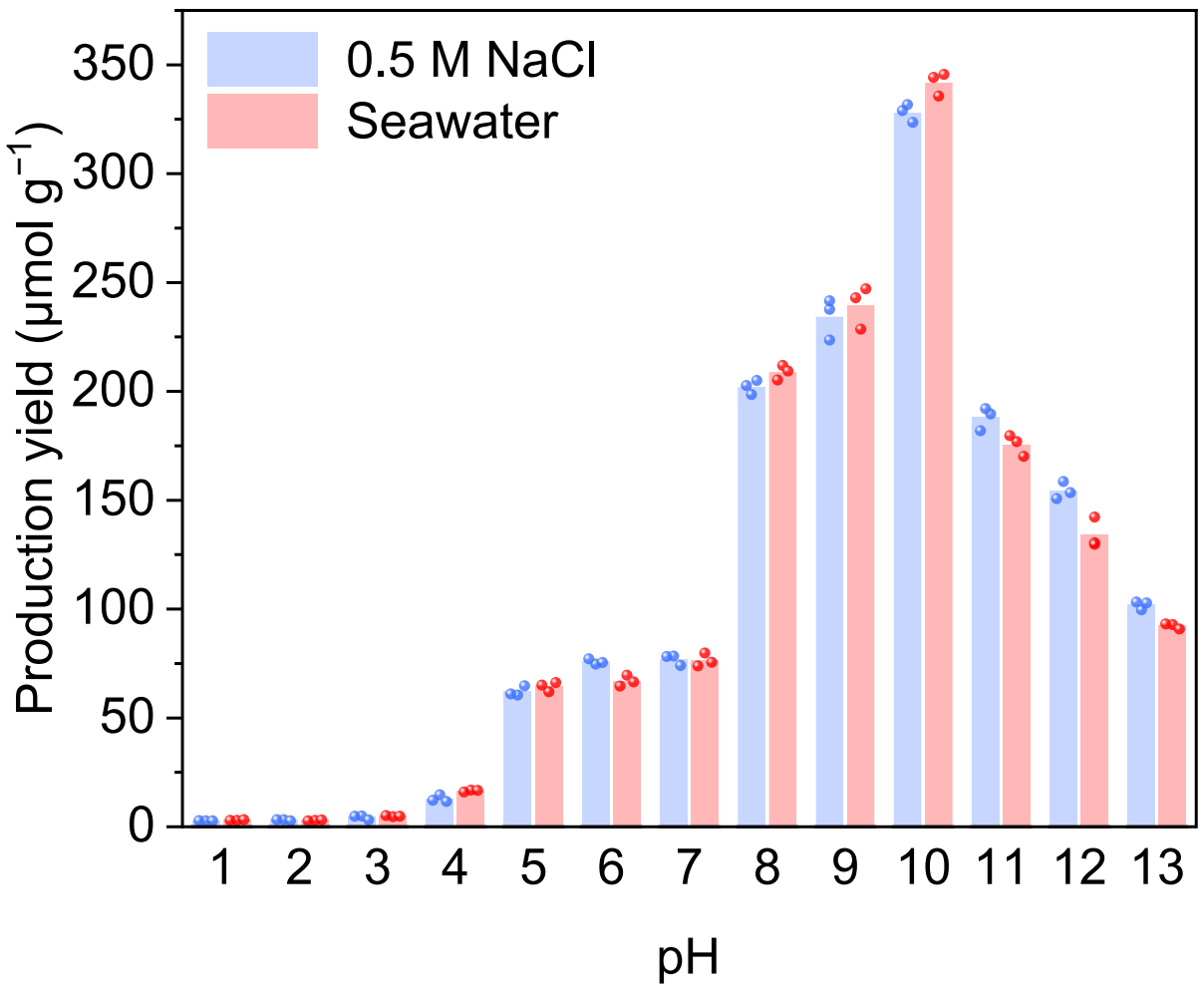

Supplementary Figure 21. Comparison of active chlorine production yields under different pH levels of natural seawater and NaCl solutions. All data in bar charts are presented as mean values from three independent experiments (n = 3), with dots indicating individual measurements.

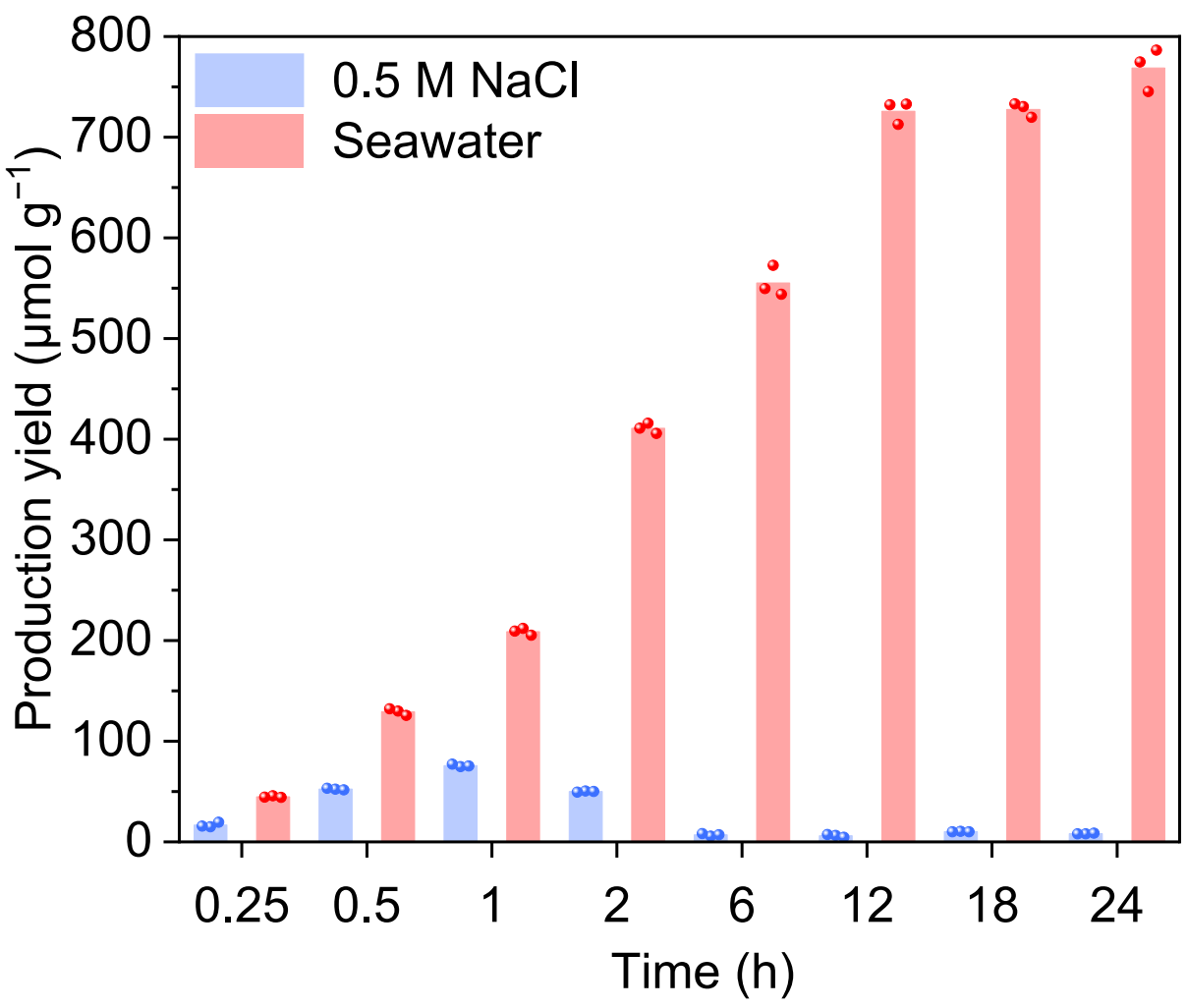

Supplementary Figure 22. Comparison of active chlorine production yields at different ultrasonication times for exposure to natural seawater and NaCl solutions. All data in bar charts are presented as mean values from three independent experiments (n = 3), with dots indicating individual measurements.

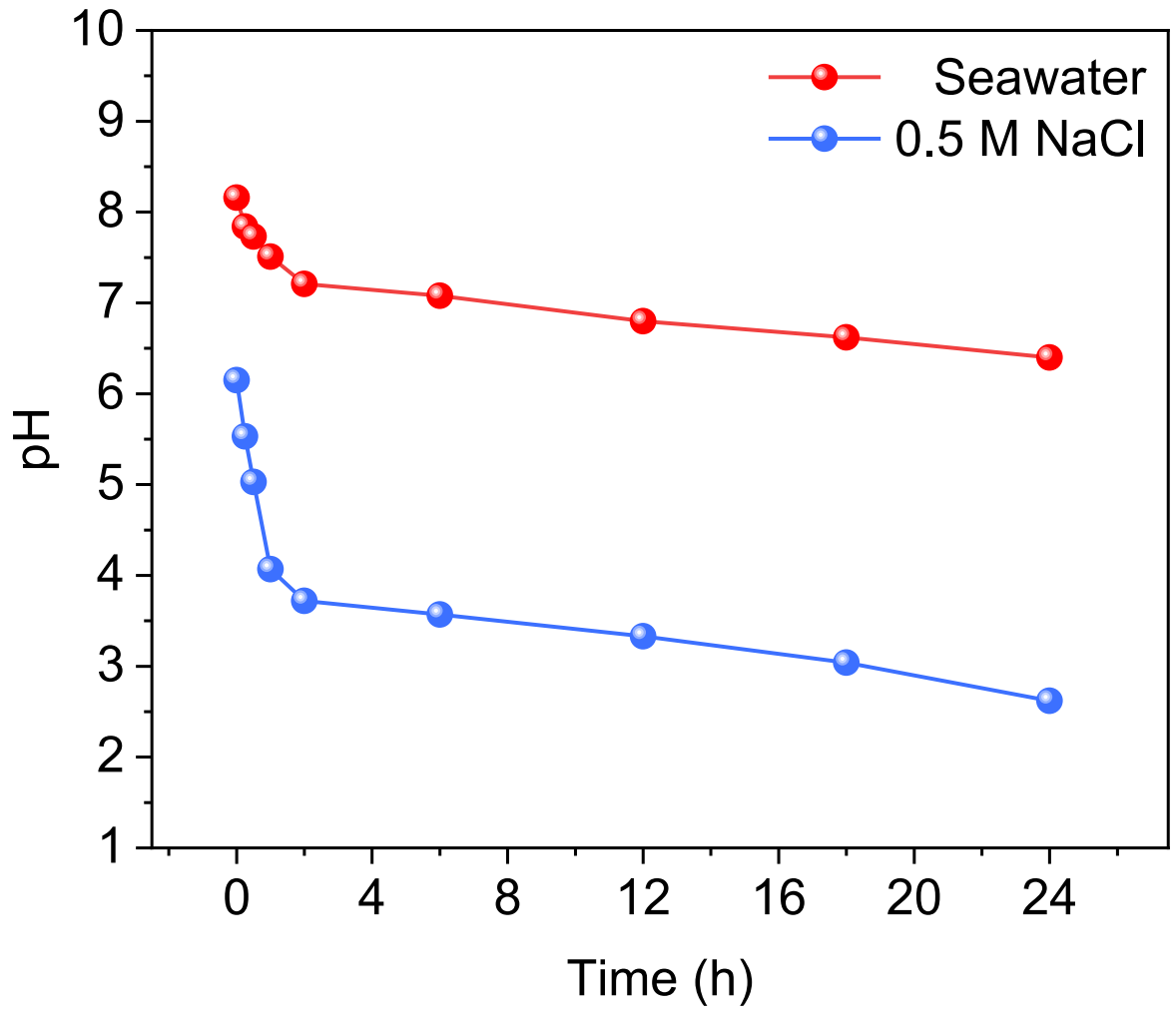

Supplementary Figure 23. Comparison of pH as a function of different ultrasonication times for natural seawater and NaCl solutions.

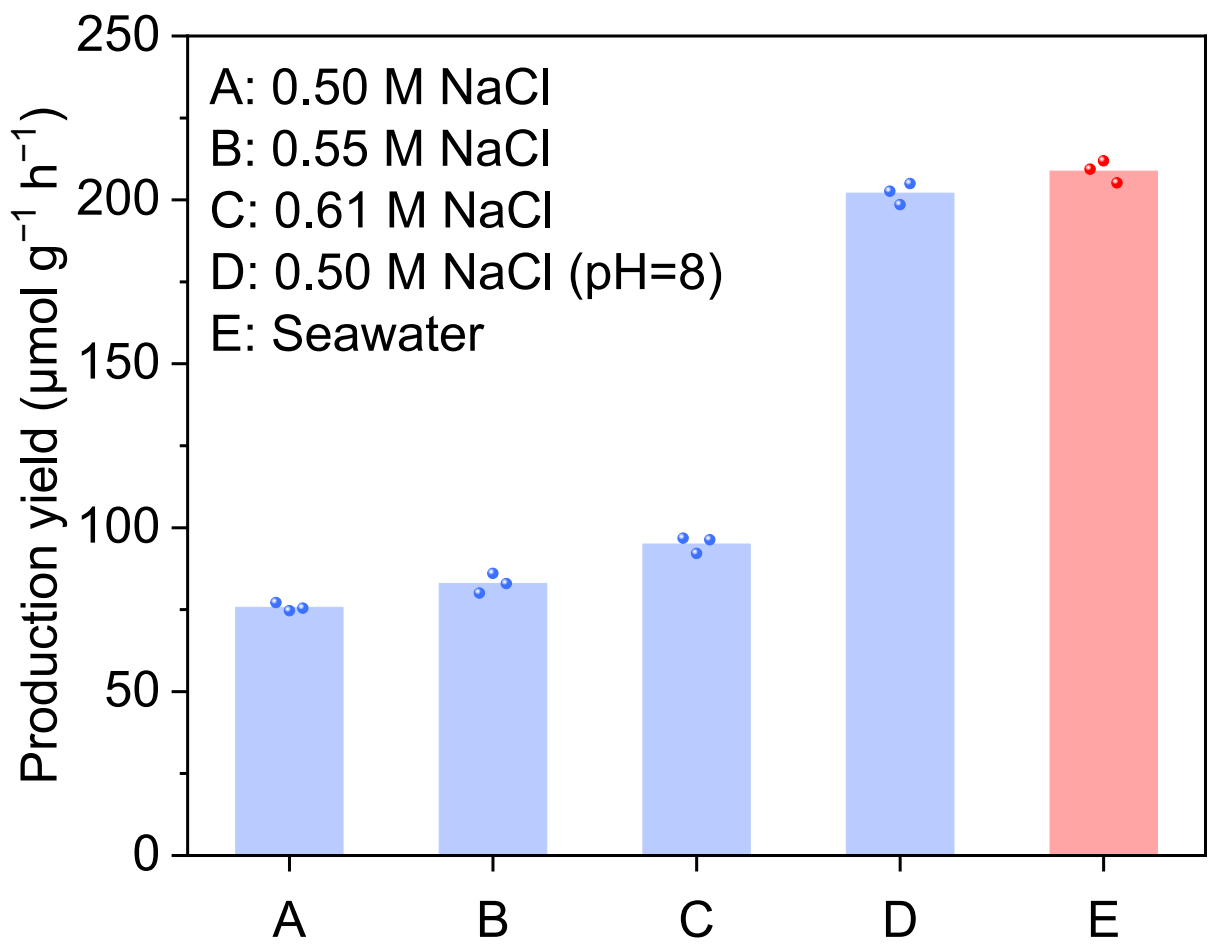

Supplementary Figure 24. Comparison of active chlorine production yields in different solutions. All data in bar charts are presented as mean values from three independent experiments (n = 3), with dots indicating individual measurements.

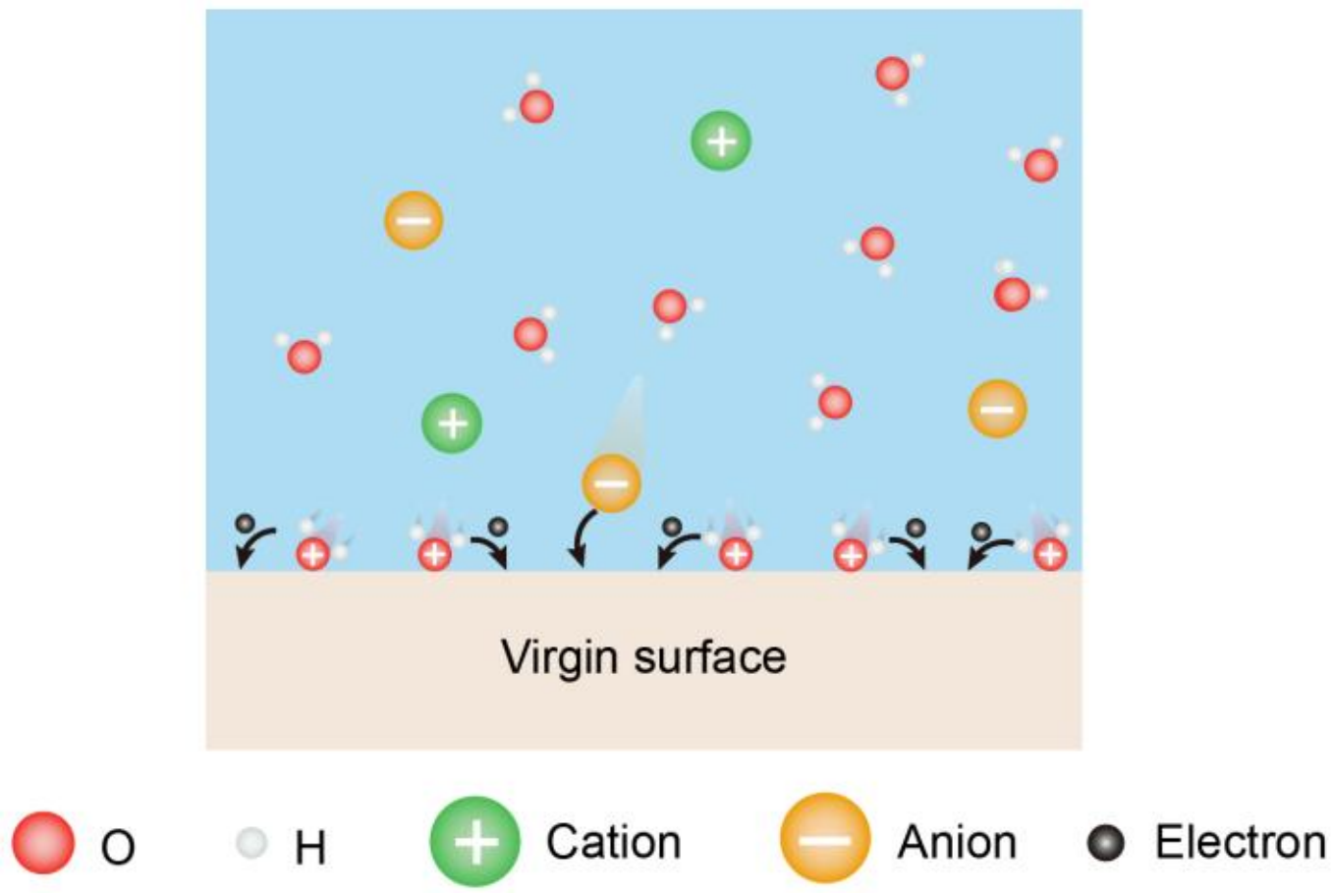

Supplementary Figure 25. EDL formation. During the EDL formation process, water molecules and ions in the liquid impact the virgin surface and electron transfer between the water and the surface.

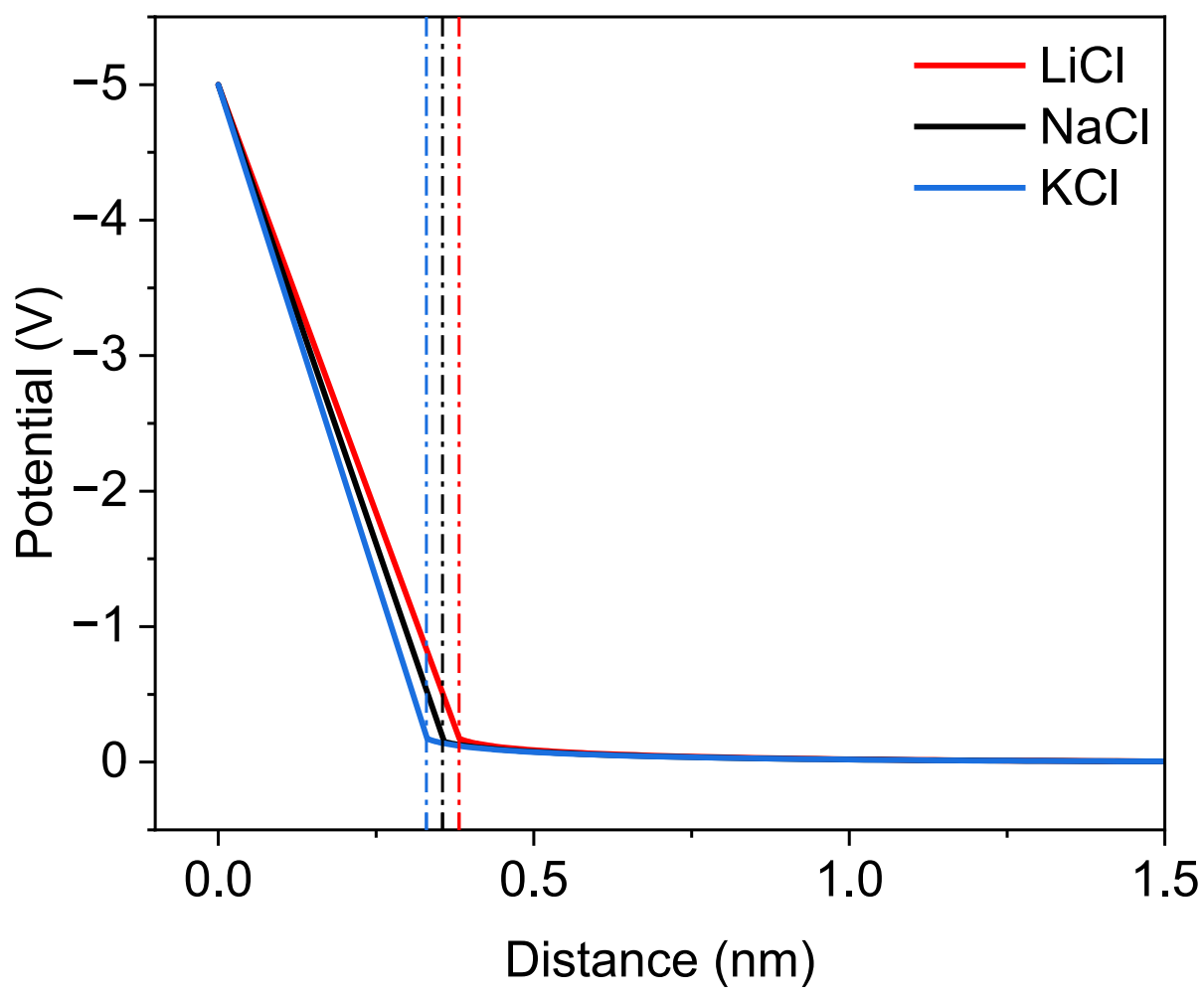

Supplementary Figure 26. The variation of the EDL potential with distance in different salt solutions.

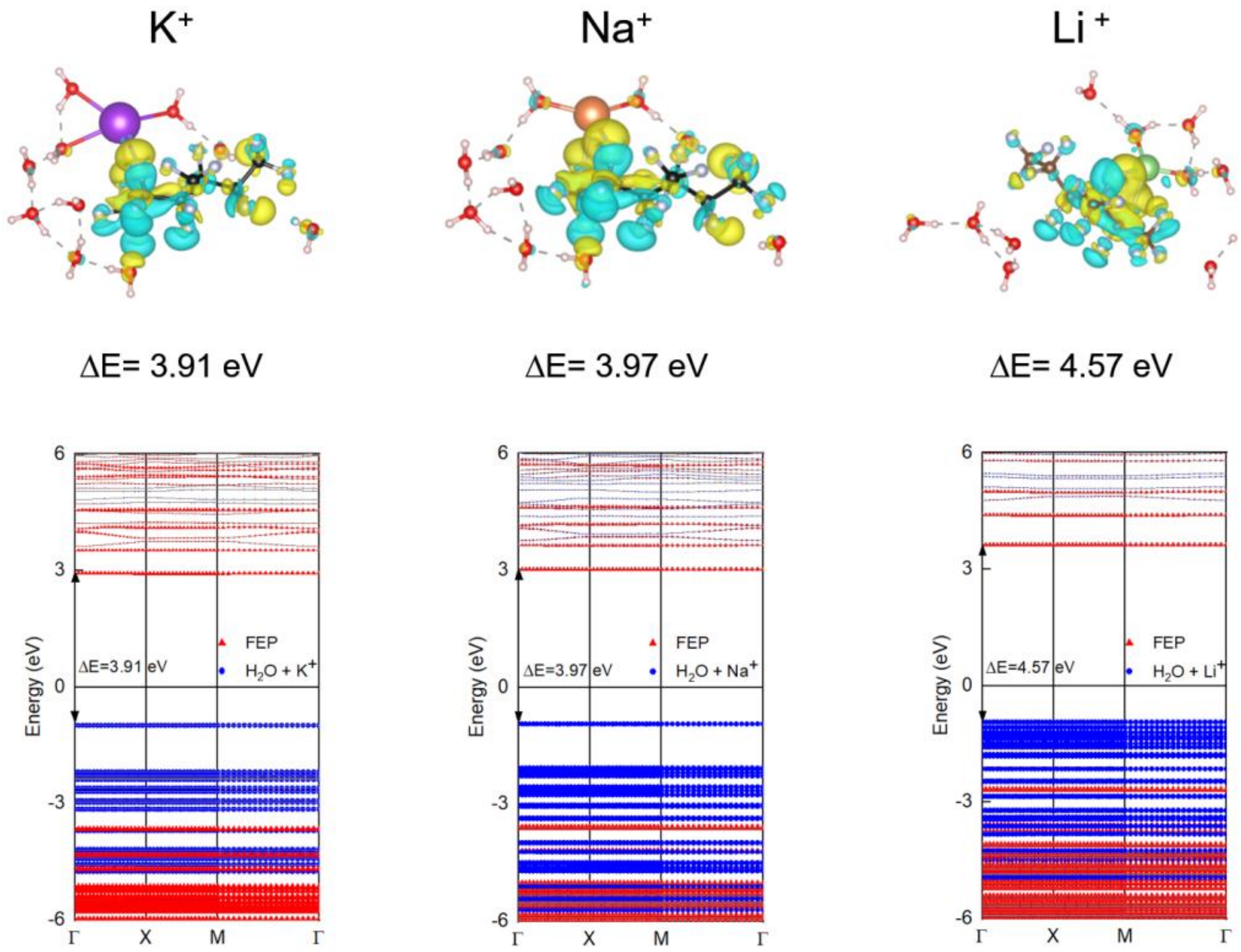

Supplementary Figure 27. DFT calculations of the energy barriers for electron transfer between FEP and H_2O with different cations. Atom colors: K, purple; Na, orange; Li, green; O, red; H, light brown.

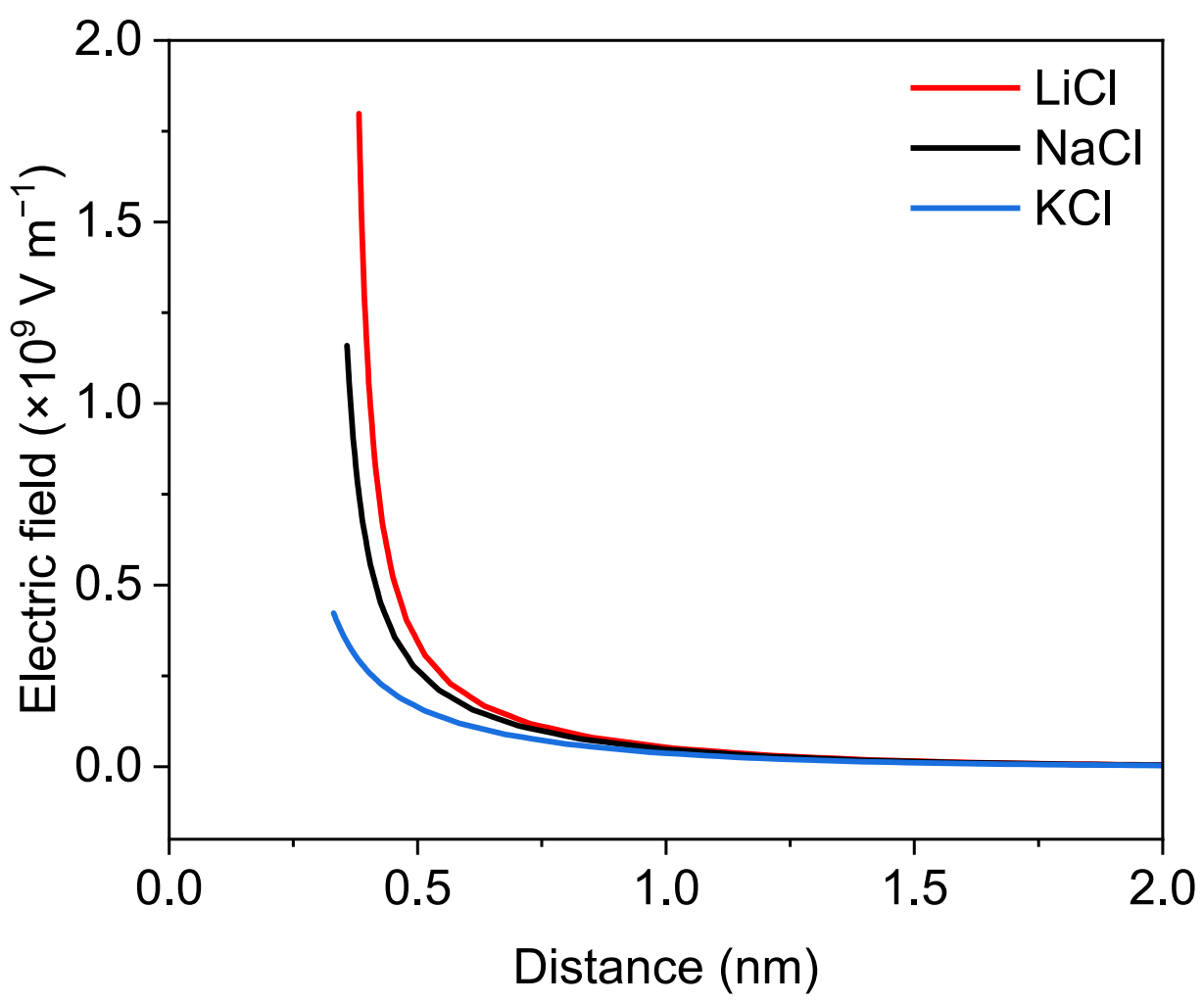

Supplementary Figure 28. The distance-dependent interfacial triboelectric field in the diffusion layer of various salt solutions.

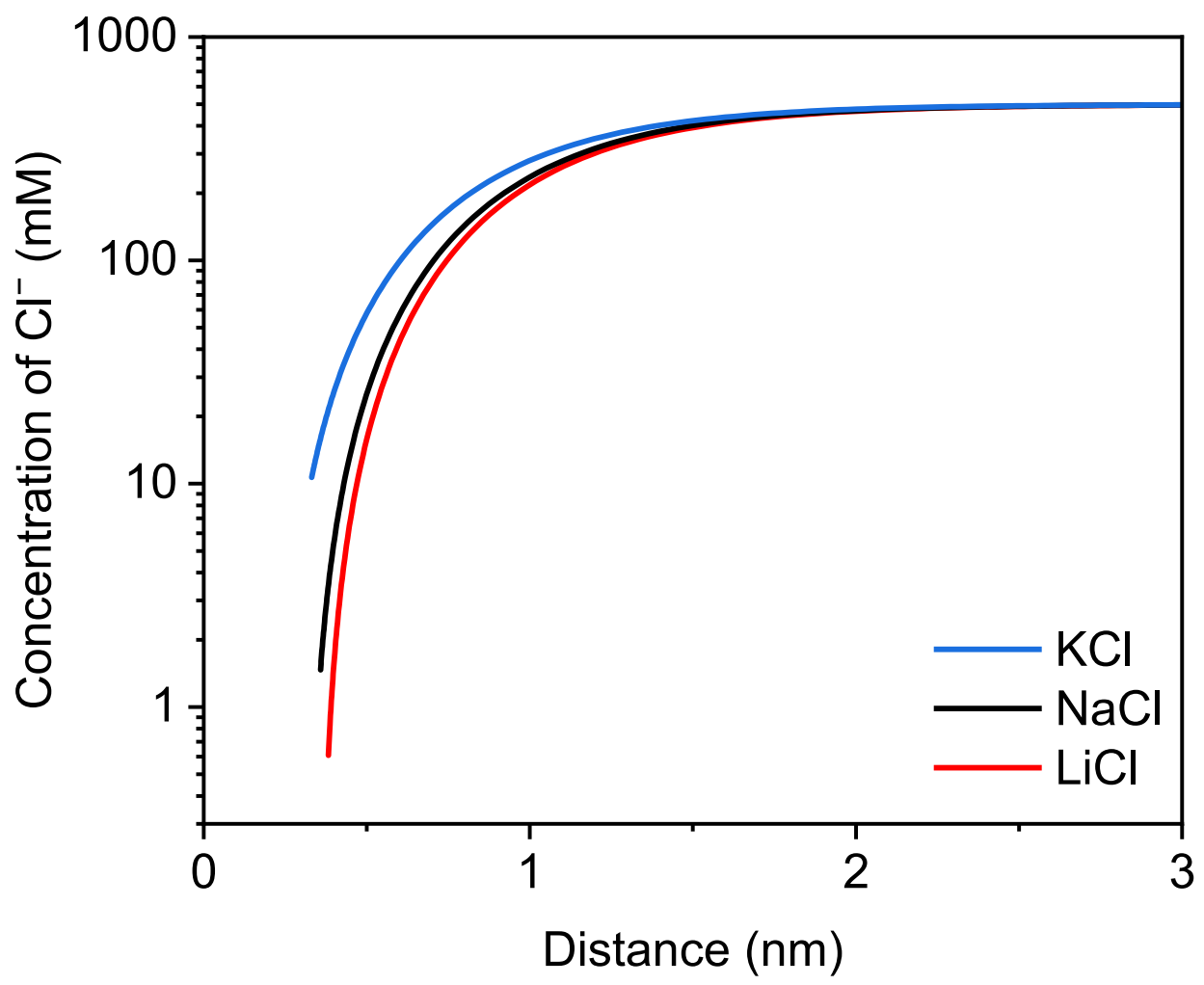

Supplementary Figure 29. Variation of the Cl⁻ concentration with distance in the diffusion layer.

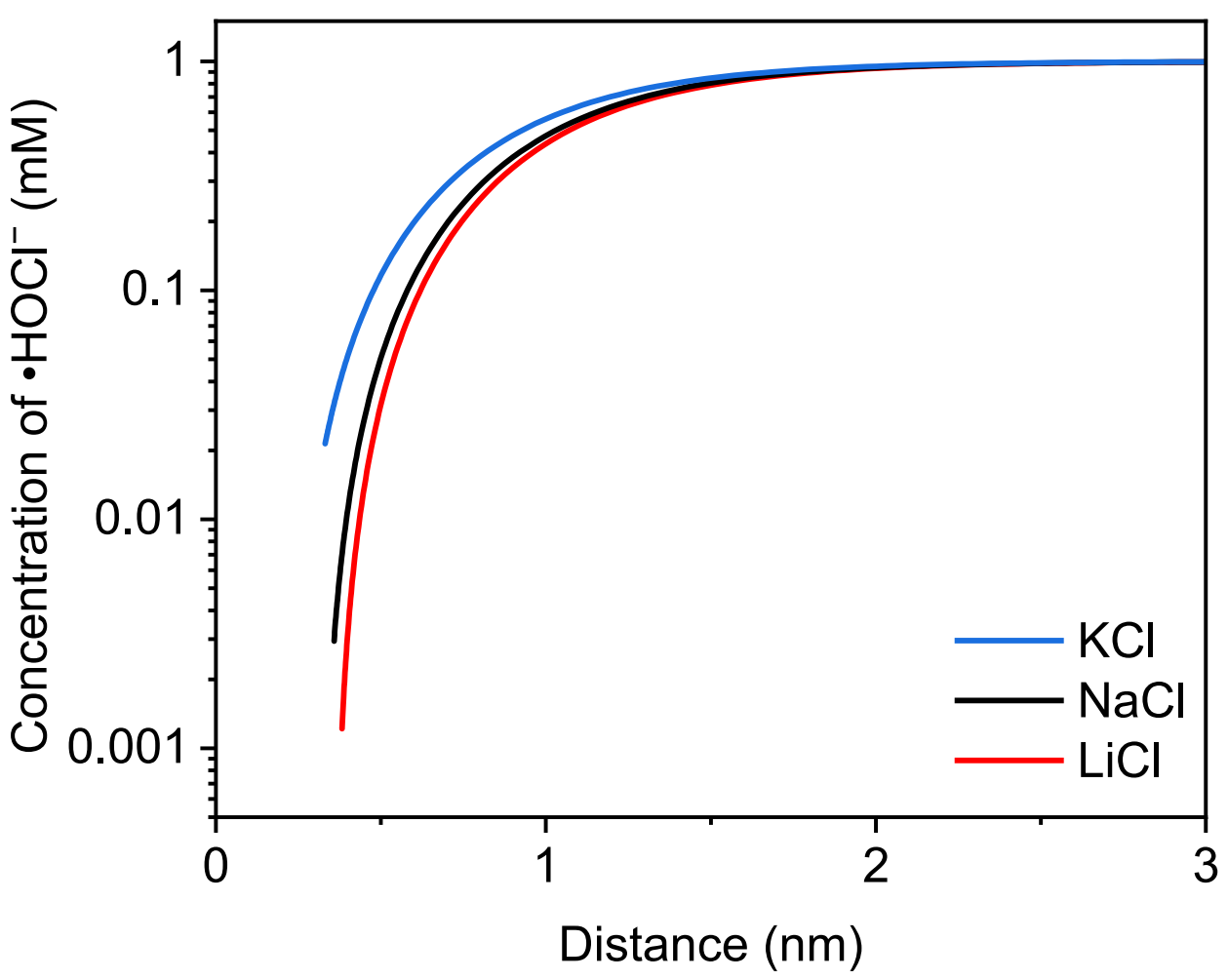

Supplementary Figure 30. Variation of the •HOCl⁻ concentration with distance in the diffusion layer.

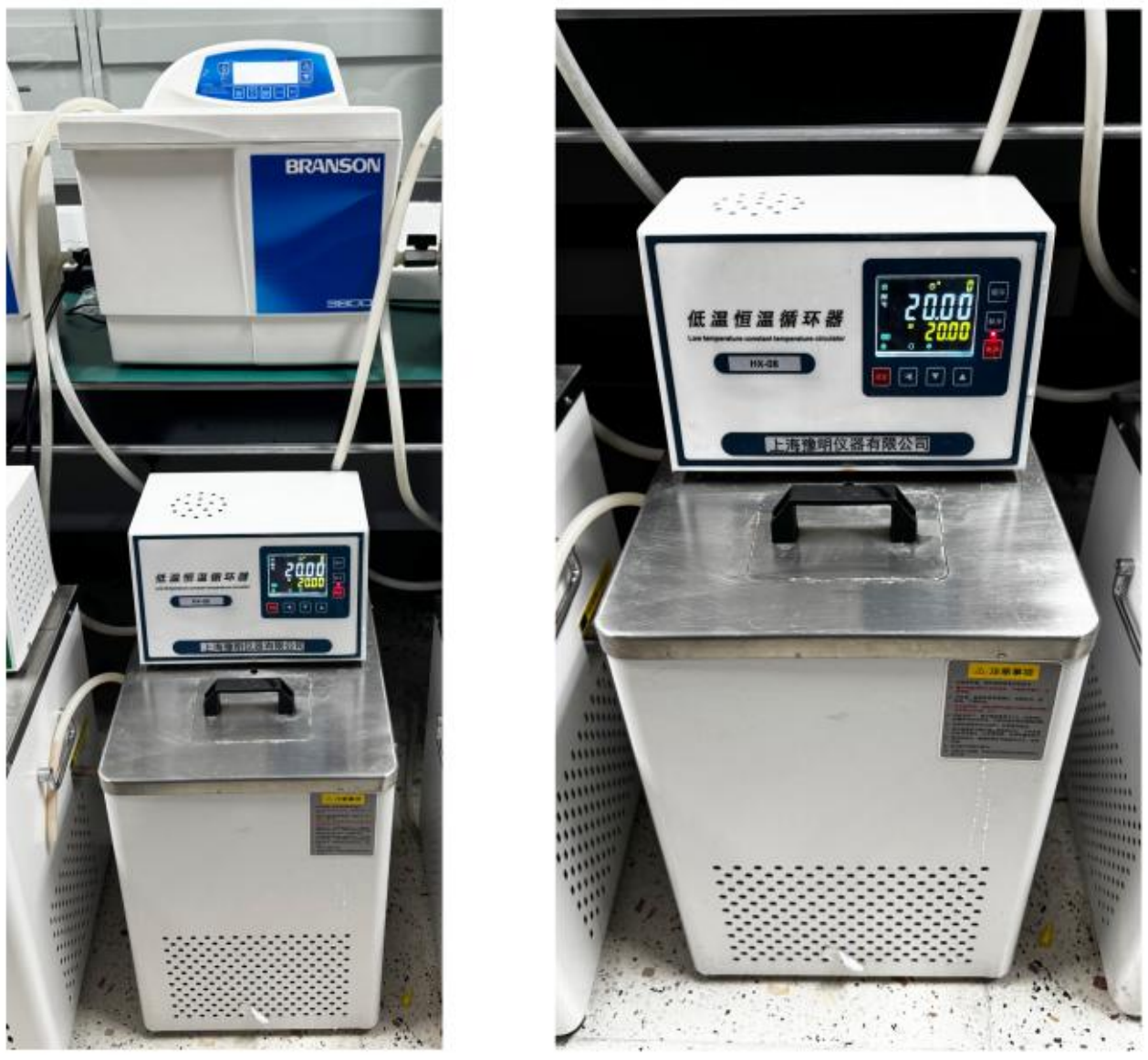

Supplementary Figure 31. Temperature-controlled ultrasonication setup enabling constant-temperature operation.

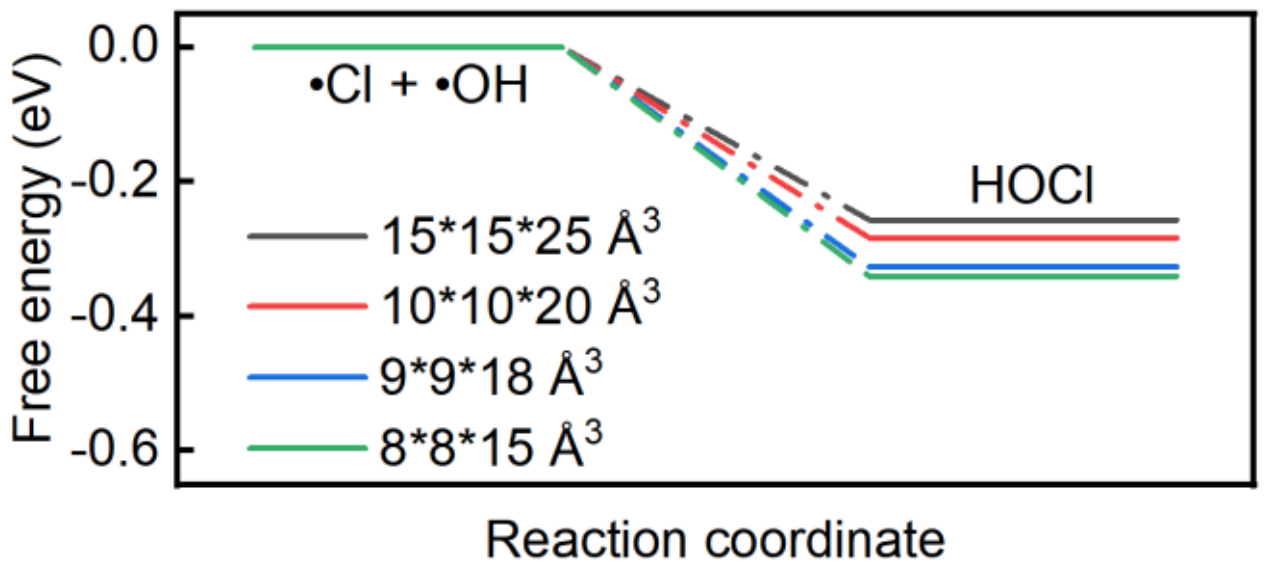

Supplementary Figure 32. Effect of unit cell volume on the Gibbs free energy of the reaction •Cl + •OH → HOCl.

Supplementary Table 1. Component and ion concentrations of the substitute seawater according to ASTM D 1141–1998 (2013).

Component	Amount (g/L)	Ion	Concentration (M)
NaCl	24.53	Na^+	0.447
$MgCl_2$	5.20	Mg^{2+}	0.055
Na_2SO_4	4.09	Ca^{2+}	0.010
$CaCl_2$	1.16	K^+	0.009
KCl	0.695	Cl^-	0.559
-	-	SO_4^{2-}	0.029
Total	35.675	Total	0.610

Supplementary Table 2. Ion composition and concentrations of natural seawater used in this study.

Ion	Concentration (M)
Na^+	0.487
Mg^{2+}	0.040
Ca^{2+}	0.009
K^+	0.008
Cl^-	0.517
SO_4^{2-}	0.017
HCO_3^-	0.003

Supplementary Table 3. DFT calculations of the energy barriers for electron transfer between FEP and H_2O in different chemical environments.

System	15 × 15 × 25 Å^3 (eV)	8 × 8 × 15 Å^3 (eV)
•OH + Cl^-	3.90	3.76
•$HOCl^-$	3.62	3.22
•$HOCl^-$ + H^+	4.90	4.85
•Cl + H_2O	4.84	4.70
•OH + •Cl	5.20	5.16
HOCl	4.97	4.67

Supplementary Table 4. Summary of the hydrated radii of the investigated ions.

Ion	Hydrated radius (Å)
K^+	3.31
Na^+	3.58
Li^+	3.82
Ba^{2+}	4.04
Ca^{2+}	4.12
Mg^{2+}	4.28
