# Supplementary material for: A triboelectric radical generation route to chlorine disinfectants from brine
Source: Nat Commun. 2026 Jul 7;17:8377. doi: 10.1038/s41467-026-75108-3 (PMC13473123; doi:10.1038/s41467-026-75108-3)
Supplement: Supplementary file 2 [file 41467_2026_75108_MOESM2_ESM.pdf]

nature portfolio

Peer Review File

A triboelectric radical generation route to chlorine disinfectants from brine

Corresponding Author: Professor Di Wei

This file contains all reviewer reports in order by version, followed by all author rebuttals in order by version.

Version 0:

Reviewer comments:

Reviewer #1

(Remarks to the Author)
In this manuscript, the authors propose a "contact-electro-chemistry" (CE-Chemistry) strategy for the direct generation of active chlorine disinfectants from natural seawater. By utilizing the ultrasonication-driven contact-separation between fluorinated ethylene propylene (FEP) powder and brine, the authors claim to establish a strong interfacial triboelectric field that triggers the generation of hydroxyl radicals and chlorine radicals, which subsequently form HOCl. The work provides a detailed mechanism regarding the Electrical Double Layer (EDL) modulation via cation hydration and claims a high stability of over 700 hours. The topic is interesting, fitting the scope of Nature Communications. The concept of utilizing CE-Chemistry to bypass the generation of toxic Cl2 is innovative. However, several critical issues need to be addressed before considering publication. Major comments are listed below.

(1) The abstract state this is a "catalyst-free" strategy. However, FEP powder is added to the system, participates in the electron transfer/field generation, and is recovered/reused. In the context of heterogeneous catalysis, FEP acts effectively as a physical catalyst or mediator. Describing it as "catalyst-free" might be misleading to the broad chemistry community.
(2) The experiments are conducted in a 10 mL volume? For practical application, How does the attenuation of ultrasound in larger volumes affect the triboelectric performance? A discussion or a proof-of-concept experiment in a larger reactor (e.g., 100 mL or 1 L) would significantly strengthen the manuscript.
(3) The authors claim the process avoids Cl2 formation (Fig. 3 and Supplementary Fig. 9). Thermodynamically, the combination of two ·Cl radicals or the oxidation of Cl- often leads to Cl2. The authors propose pathways (Eq. 5, 6, 7) where ·Cl reacts with ·OH to form HOCl directly. Can the authors provide calculation or other support specifically comparing the energy barriers of ·Cl+·Cl→Cl2 versus ·Cl+·OH→HOCl in their specific environment? This is crucial to explain or prove the high selectivity.
(4) The active chlorine is detected using the o-tolidine method. However, this method can be interfered by other strong oxidants which are likely generated in this CE-Chemistry system. Although MS was used for gas analysis, did the authors verify the liquid phase selectivity?
(5) One of the main points of this work is "sustainability." However, the system operates at 120 W (ultrasonic power) to treat a relatively small volume with 20 mg of catalyst. The authors should calculate the energy consumption per mole of active chlorine produced (kWh/mol or kWh/kg) and compare it with the industrial chlor-alkali process and other emerging electrochemical seawater splitting technologies. If the energy cost is much higher than those, the claim of a "sustainable paradigm" needs to be justified.
(6) Ultrasonication is known to generate significant heat in the bulk solution over time, yet the manuscript does not specify the temperature control measures during the long-term (700 h) experiments. The authors acknowledge that HOCl decomposition is accelerated at high temperatures. Furthermore, the dielectric constant and triboelectric charge density of polymers like FEP are often temperature-dependent. The authors need to clarify if the reaction temperature was thermostated. If not, how much of the yield fluctuation is influenced by the thermal degradation of HOCl (Equation 10) versus the generation rate?
(7) The yield is shown to decrease when FEP dosage exceeds 20 mg, attributed to ultrasonic scattering. However, the manuscript does not provide the specific surface area (m2/g) of the FEP powder used. Since CE-Chemistry is strictly an interfacial phenomenon, the reaction rate should be normalized by the active surface area rather than mass to allow for reproducible comparisons.
(8) Are the chlorine species concentrations in Supplementary Figures 24 and 25 simulated values or that of experiments? Could the experimental values for actual LiCl be provided?

Reviewer #2

(Remarks to the Author)
In the present manuscript, the authors investigate a contact electrification strategy combined with ultrasonication for active chlorine formation. The system was optimized by varying dielectric materials, powder loading, and salt identity/concentration, ultimately achieving a production rate of 208.8 μmol/g*h using 20 mg of FEP powder over 700 h.

My primary concern relates to the claim that this approach is catalyst- and bias-free. While no external electrochemical bias is applied, the system relies on substantial mechanical energy input via ultrasonication (120 W). When considering the absolute production rate (not normalized to FEP mass), the overall active chlorine yield corresponds only to a few nanomoles per hour. From an energy-efficiency and cost-effectiveness standpoint, this performance is never competitive. Given that the authors benchmark their system against the conventional chlor-alkali process, the present approach does not yet demonstrate a viable alternative for sustainable active chlorine production.

Beyond the practical limitations, I have concerns regarding the fundamental interpretation. Much of the discussion on electric field strength and interfacial effects relies on computational simulations. As the authors noted, on metal surfaces, electric field strength scales with solvated ion size of alkali cations or effective positive charge. However, for the FEP surface examined in this study, no direct experimental evidence (such as measurement of the Stark tuning rate) was provided to validate the proposed interfacial electric field under realistic operating conditions. Without such experimental verification, the mechanistic interpretation remains speculative. Incorporating operando or in situ spectroscopic evidence to quantify interfacial field strength would significantly strengthen this otherwise scientifically fragile aspect of the manuscript.

Overall, the manuscript was well written, but there seem room for improving the quality of the manuscript further for consideration in publication.

Reviewer #3

(Remarks to the Author)
The manuscript entitled “A triboelectric radical generation route to chlorine disinfectants from brine” firstly reported active chlorine prepared by CE-chemistry. Besides, the effect of EDL on CE-chemistry was discussed. However, there are some problems that need to be solved. In particular, the effects of EDL on CE-chemistry and experimental details need to be carefully discussed and supplemented. Considering the overall work, I recommend consideration of the manuscript for publication after a careful revision where the following points are addressed.
1. Why was a 0.5M NaCl solution chosen? Is there any preliminary experiment to support it?
2. What is the impact of ultrasonication on EDL? How will changing the ultrasound power affect the CE-chemistry performance? It is suggested to add relevant discussions.
3. Is there any testing of the composition of seawater and an introduction to its main components? It is suggested to provide additional relevant data.
4. In Fig. 3d and 3e, when there is no O2 present, where do the electrons of FEP go? Did the electrons cause stronger shielding, thereby causing CE-chemistry to cease?
5. In the XPS of Supplementary Fig. 6, the binding energy of C1s is partially missing, and there is no test for the O1s spectrum compared with the reported references of CEC.
6. Regarding the mechanism by which pH affects the reaction, it is recommended to conduct a detailed discussion. The reasons why the reaction is promoted under weakly alkaline conditions and inhibited under strongly alkaline conditions, and why no moderate performance was observed between pH = 6 and pH = 8 under neutral conditions.
7. In Supplementary Fig. 17, what is the reason for the decrease in pH of the NaCl solution over time?
8. For the two reaction routes, which of the three pathways for generating HOCl is the main reaction path?
9. When the solution is acidic, the high temperature generated by ultrasonic cavitation will inhibit the reaction. The influence of temperature on EDL and the effect of synthesizing chlorine species through CE-chemistry should be discussed and supplemented.
10. Is there any reference for the compression ratio of volume during the DFT calculation? Compared with previous literature, is the compression ratio of volume overly compressed?
11. Does the electric field generated by the contact electrification of solid-liquid interfaces play a dominant role in the interface electric field? What proportion does the EDL contribute to the overall electric field and promote the redox reaction process?
12. Through the comparison between Fig. 4a and 4b, the radius and performance of K+ and Ca2+ do not follow exactly the law in the manuscript. The influence of ion valence states on EDL and reactions should be carefully discussed.
13. How does the addition of salt solution affect the electrochemical impedance, and how does it affect the CE-chemistry reaction? These should be discussed.
14. How does the work compare with other works that produce chlorine disinfectants, and does it have a certain advantage in cost, as well as the advantages of industrialization?
15. It is recommended to supplement the charge generation performance of different salt solutions and FEP solid-liquid CE.

Version 1:

Reviewer comments:

Reviewer #1

(Remarks to the Author)
The authors have addressed my concerns, and I have no more comments.

Reviewer #2

(Remarks to the Author)
This reviewer thanks authors for their efforts on responding the reviewer' comments. In this version. I have no more comments and support its publication.

Reviewer #3

(Remarks to the Author)
The authors have perfectly resolved my concerns. I recommend that it can be considered for acceptance.

Open Access This Peer Review File is licensed under a Creative Commons Attribution 4.0 International License, which permits use, sharing, adaptation, distribution and reproduction in any medium or format, as long as you give appropriate credit to the original author(s) and the source, provide a link to the Creative Commons license, and indicate if changes were made.
In cases where reviewers are anonymous, credit should be given to 'Anonymous Referee' and the source.
The images or other third party material in this Peer Review File are included in the article's Creative Commons license, unless indicated otherwise in a credit line to the material. If material is not included in the article's Creative Commons license and your intended use is not permitted by statutory regulation or exceeds the permitted use, you will need to obtain permission directly from the copyright holder.
To view a copy of this license, visit https://creativecommons.org/licenses/by/4.0/

Replies to Comments

A triboelectric radical generation route to chlorine disinfectants from brine

Research Article, NCOMMS-25-103184

Dear Editor and Reviewers:

We deeply appreciate the valuable comments from both the editor and the reviewers, which have helped improve the paper. Provided below is our detailed response to each comment/suggestion. The specific changes made to the Manuscript and Supplementary Information to address each point are highlighted in yellow.

Responses to Reviewers' Comments:

Reviewer #1 (Remarks to the Author):

In this manuscript, the authors propose a "contact-electro-chemistry" (CE-Chemistry) strategy for the direct generation of active chlorine disinfectants from natural seawater. By utilizing the ultrasonication-driven contact-separation between fluorinated ethylene propylene (FEP) powder and brine, the authors claim to establish a strong interfacial triboelectric field that triggers the generation of hydroxyl radicals and chlorine radicals, which subsequently form HOCl. The work provides a detailed mechanism regarding the Electrical Double Layer (EDL) modulation via cation hydration and claims a high stability of over 700 hours. The topic is interesting, fitting the scope of *Nature Communications*. The concept of utilizing CE-Chemistry to bypass the generation of toxic Cl_2 is innovative. However, several critical issues need to be addressed before considering publication. Major comments are listed below.

Response: We are deeply appreciated to the reviewer's precious suggestions and valuable comments to improve the paper. In response to the reviewer's comments, we have made point-to-point modifications to the manuscript.

1. The abstract state this is a "catalyst-free" strategy. However, FEP powder is added to the system, participates in the electron transfer/field generation, and is recovered/reused. In the context of heterogeneous catalysis, FEP acts effectively as a physical catalyst or mediator. Describing it as "catalyst-free" might be misleading to the broad chemistry community.

Response: We thank the reviewer for this insightful comment. In the present work, our intention was to emphasize the absence of metal catalysts (particularly noble or transition metals), rather than to imply the absence of any solid mediator. We acknowledge that the added FEP powder participates in interfacial charge transfer and can function as a physical mediator within the system.

To avoid ambiguity, we have revised the manuscript by replacing "catalyst-free" with "metal-catalyst-free" or "metal-free" throughout. The corresponding clarifications have been incorporated into the revised manuscript, and the relevant sections have been updated accordingly. We sincerely thank the reviewer for this constructive suggestion, which has helped improve the clarity and precision of our presentation.

The corresponding discussions had been added in the revised Manuscript. Detailed revision is as below. Thank you most sincerely!

In the revised Manuscript:

(Page 2) *Utilizing a triboelectric radical generation route from natural seawater, this work demonstrates a metal-catalyst-free and sustainable strategy for active chlorine production, achieving a yield of 208.8 μmol g^{-1} h^{-1} with only 20 mg of FEP powder.*

(Page 14) *The strategy presents distinct practical advantages: the dielectric materials are intrinsically stable and recyclable, the process operates without conventional noble-metal catalysts, and the reaction proceeds independently of electrochemical window constraints or inevitable side reactions, thereby enabling spatiotemporal decoupling of paired redox processes.*

2. The experiments are conducted in a 10 mL volume? For practical application, how does the attenuation of ultrasound in larger volumes affect the triboelectric performance? A discussion or a proof-of-concept experiment in a larger reactor (e.g., 100 mL or 1 L) would significantly strengthen the manuscript.

Response: We sincerely thank the reviewer for this insightful and constructive suggestion. In the present study, experiments were primarily

conducted in a 10 mL system. To evaluate scalability, we performed a proof-of-concept experiment by increasing the volume of natural seawater from 10 mL to 100 mL while maintaining identical operating conditions. The results show that the active chlorine yield remains essentially unchanged up to ~50 mL, indicating negligible attenuation effects in this range. However, further increases in volume lead to pronounced ultrasonic attenuation, which weakens interfacial contact electrification, slows reaction kinetics, and ultimately reduces active chlorine production (**Fig. R1**), in agreement with previous reports (*Nat. Energy, 2023, 8, 1137*). These results highlight that ultrasound propagation plays a critical role in sustaining triboelectric performance at larger scales. For practical applications, optimization of ultrasonic parameters, particularly probe number, power density, and spatial distribution, will be essential to ensure uniform energy delivery and maximize both reaction efficiency and energy conversion efficiency in larger reactors. The corresponding discussion has been added to the revised manuscript.

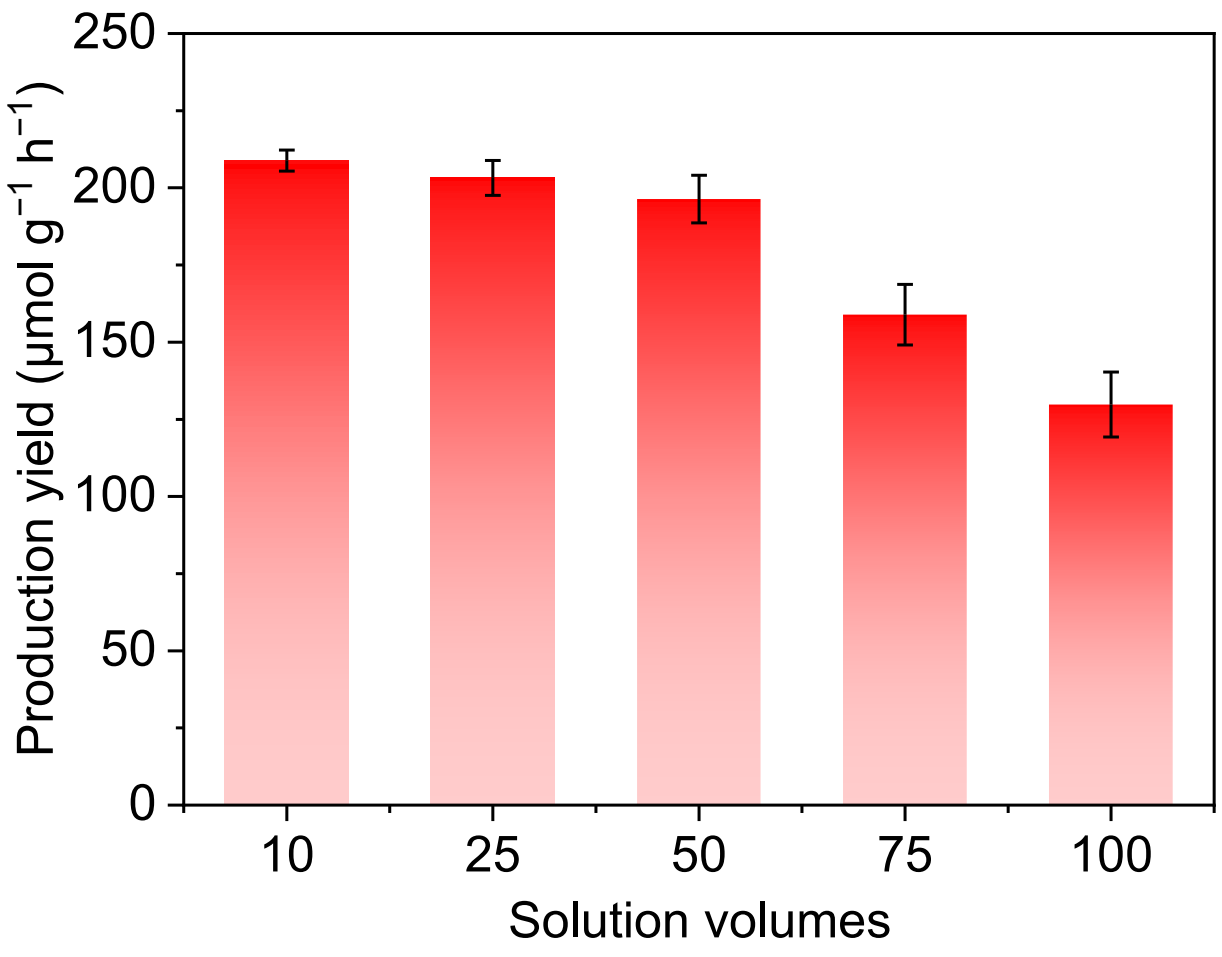

Fig. R1. Comparison of active chlorine production yields at different solution volumes of natural seawater solution.

The corresponding discussions had been added in the revised Manuscript and revised Supplementary Information. Detailed revision is as below.

In the revised Manuscript:

(Page 6) *The reaction volume was increased from 10 to 100 mL to evaluate ultrasonic wave attenuation. Negligible attenuation was observed below 50 mL, whereas further increases led to pronounced attenuation, limiting reaction kinetics and reducing active chlorine production, consistent with previous reports*[21] *(Supplementary Fig. 2).*

(Page 20) *The reaction was conducted under optimal conditions of 20 °C, 20 mg FEP, 10 mL of natural seawater, and thermostatic ultrasonication*

at 40 kHz and 120 W for 1 h. For systematic investigations of the effects of solution volume (10, 25, 50, 75, 100 mL), temperature (5, 10, 20, 30, 40 °C), or ultrasonic power (60, 120, 200, 300, 400 W), all tests were designed based on these optimal conditions, with only a single variable altered each time.

In the revised Supplementary Information:

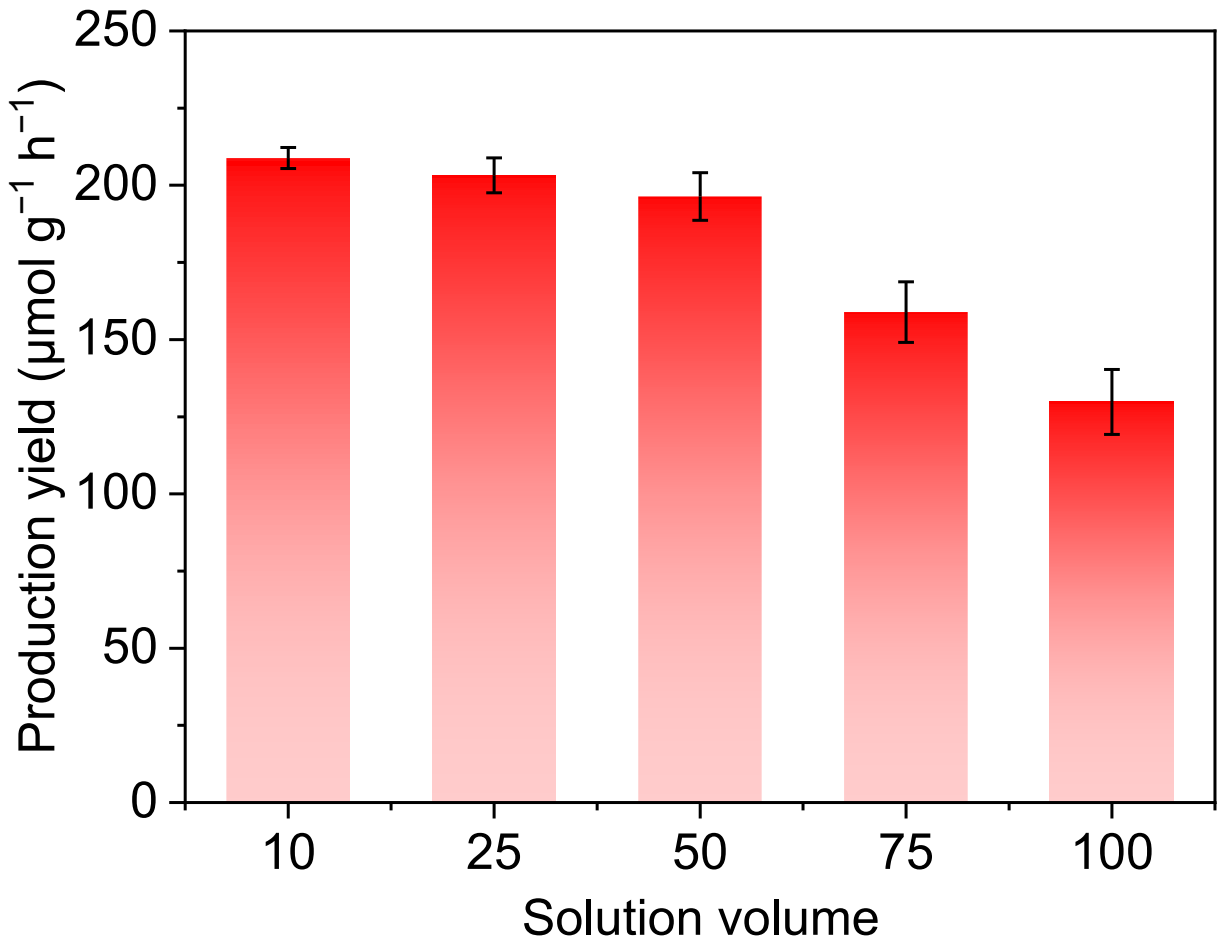

(Page 1) *Supplementary Figure 2. Comparison of active chlorine production yields at different solution volumes of natural seawater solution.*

3. The authors claim the process avoids Cl_2 formation (Fig. 3 and Supplementary Fig. 9). Thermodynamically, the combination of two •Cl radicals or the oxidation of Cl^- often leads to Cl_2. The authors propose pathways (Eq. 5, 6, 7) where •Cl reacts with •OH to form HOCl directly. Can the authors provide calculation or other support specifically

comparing the energy barriers of •Cl + •Cl → Cl_2 versus •Cl + •OH → HOCl in their specific environment? This is crucial to explain or prove the high selectivity.

Response: We thank the reviewer for this important comment. The high selectivity toward HOCl in our system is both theoretically and experimentally well supported. To explicitly address the competing pathways, we have conducted additional density functional theory (DFT) calculations comparing •Cl + •Cl → Cl_2 and •Cl + •OH → HOCl under the transient high-pressure conditions generated by cavitation. The results indicate that both reactions proceed via barrierless radical recombination pathways. Notably, Cl_2 formation is only weakly exothermic, reflecting a negligible thermodynamic driving force, whereas HOCl formation is substantially more exothermic, indicating a markedly stronger thermodynamic preference (*J. Am. Chem. Soc., 1997, 119, 5007-5012*) (**Fig. R2**). Beyond thermodynamics, the pronounced selectivity toward HOCl is further governed by kinetic factors arising from local radical concentrations. In the CE-induced interfacial environment, •OH radicals are continuously generated and maintained at a high local concentration, whereas •Cl radicals remain comparatively dilute. This imbalance enables the rapid capture of •Cl intermediates by •OH to form HOCl along the thermodynamically favored pathway, while suppressing •Cl recombination.

Consequently, although both pathways are kinetically barrierless, ***the synergistic interplay of thermodynamic preference and local concentration-driven kinetics directs the reaction toward highly selective HOCl formation, effectively inhibiting Cl_2 generation***.

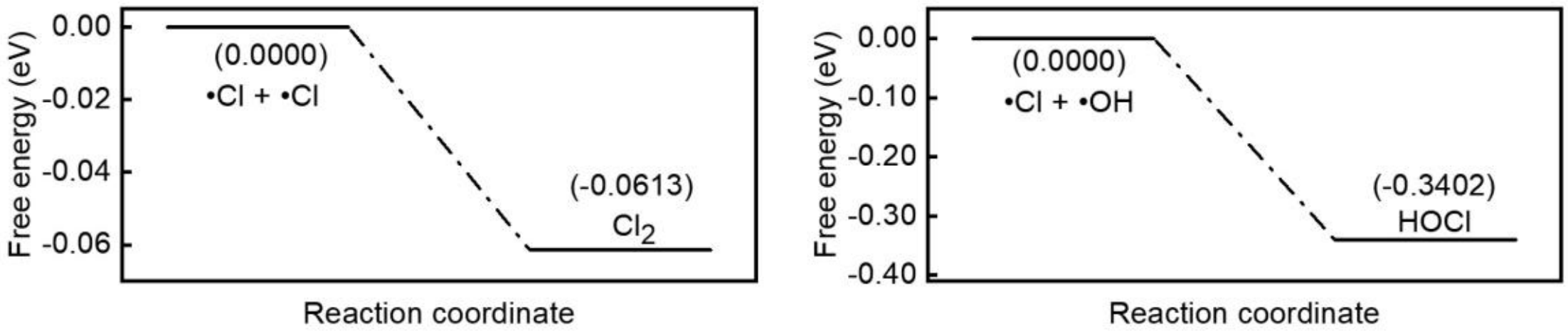

Fig. R2. The Gibbs free energy of •Cl + •Cl → Cl_2 and •Cl + •OH → HOCl reactions.

This conclusion is further corroborated by experimental evidence. The gas-phase mass spectrometry revealed no detectable Cl_2 in either NaCl or natural seawater systems across a broad pH range (**Fig. R3**). Consistently, supplementary experiments for liquid-phase analysis under varied conditions likewise show no evidence of Cl_2 formation (**Fig. R4**). Taken together, both theory and experiment unequivocally demonstrate that •Cl preferentially reacts with •OH to form HOCl rather than undergoing self-coupling. This intrinsic selectivity, arising from the unique CE-driven high-pressure interfacial environment, fundamentally differentiates the present system from conventional chlor-alkali processes

and underpins its advantages as a safe and sustainable route for active chlorine generation.

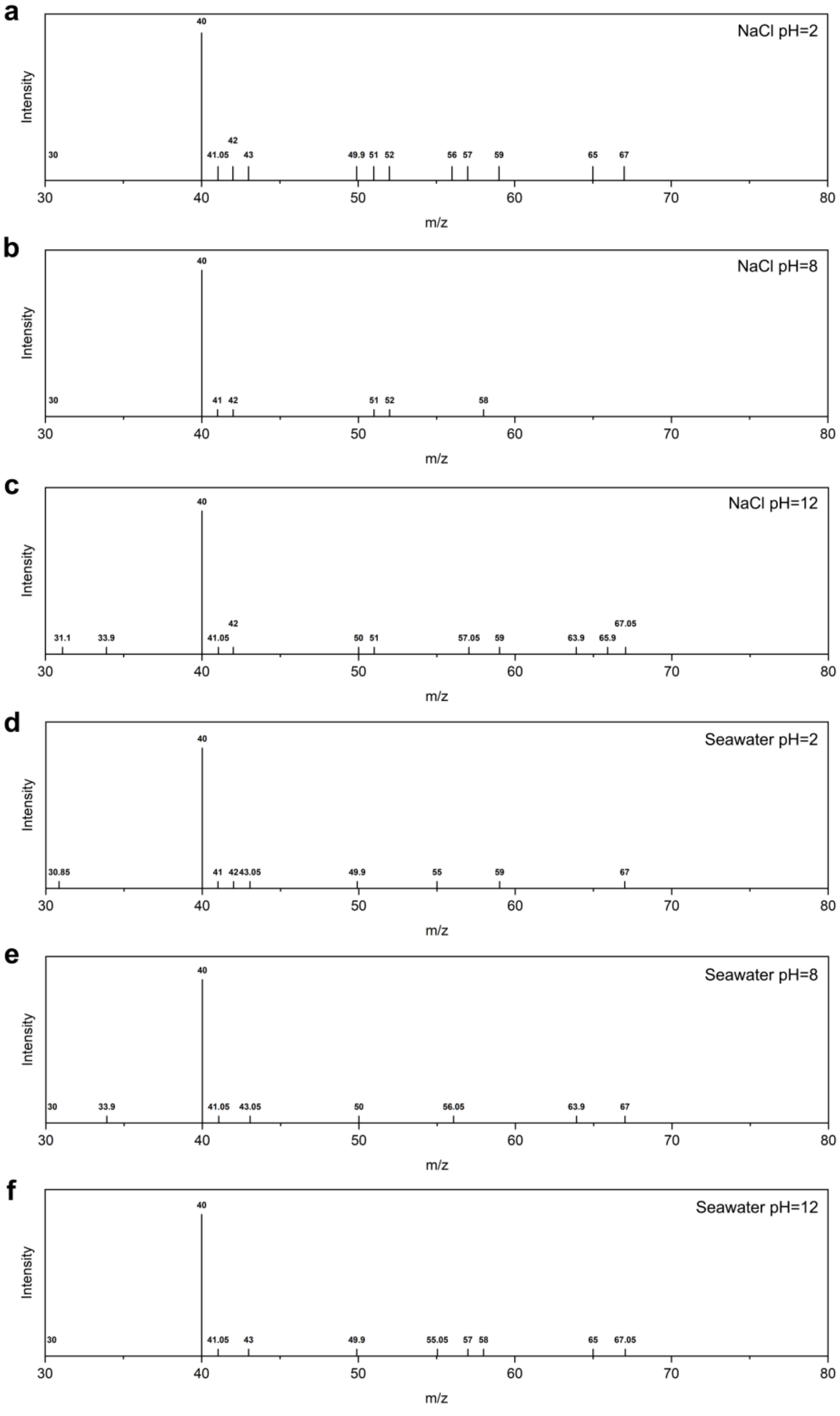

Fig. R3. Headspace gas chromatography-mass spectrometry (GC-MS) of gaseous and liquid products from the reaction system under different

conditions. a, NaCl system at pH=2. b, NaCl system at pH=8. c, NaCl system at pH=12. d, Natural seawater system at pH=2. e, Natural seawater system at pH=8. f, Natural seawater system at pH=12.

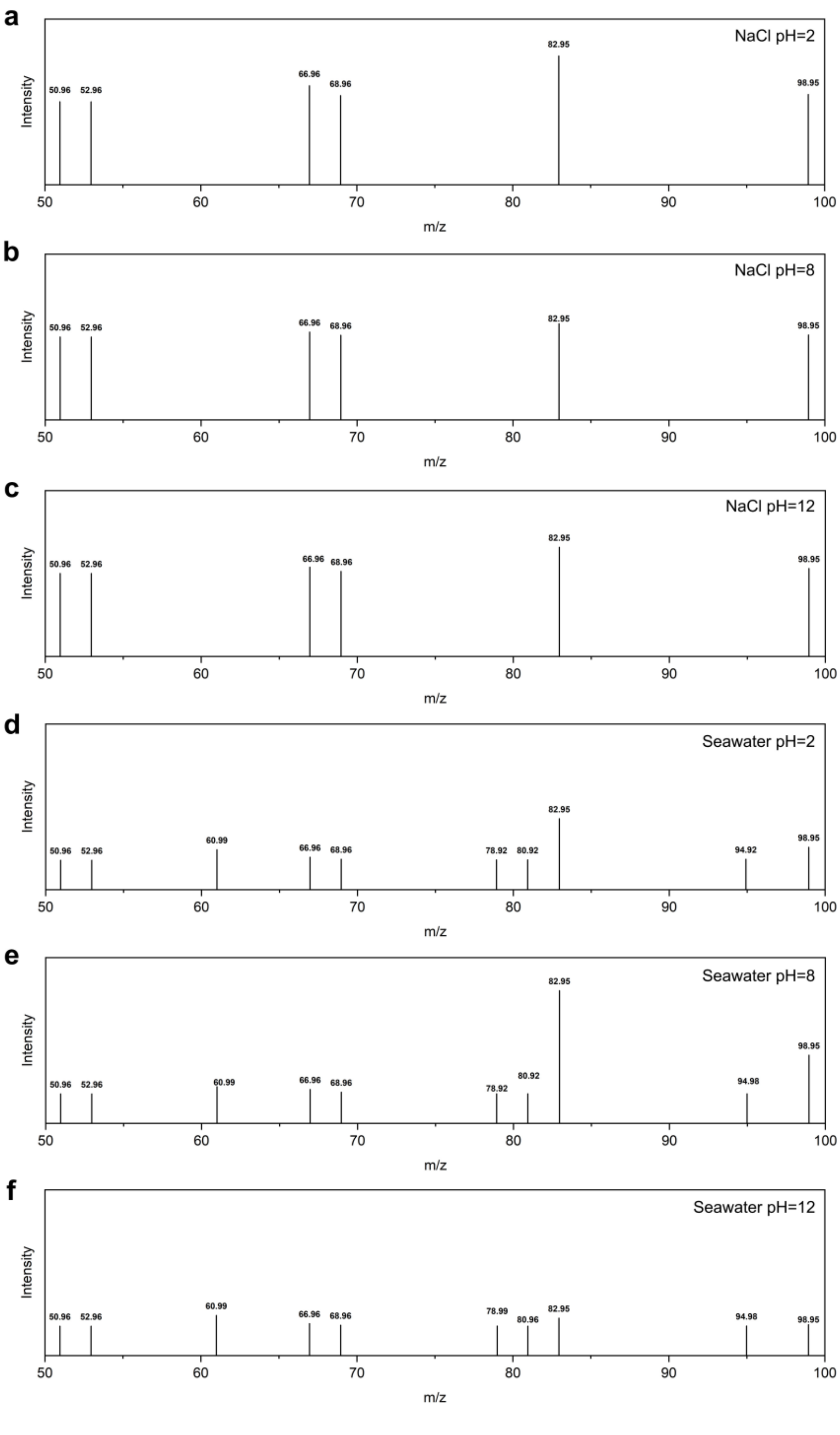

Fig. R4. Electrospray ionization mass spectrometry (ESI-MS) of liquid

products from the reaction system under different conditions. a, NaCl system at pH=2. b, NaCl system at pH=8. c, NaCl system at pH=12. d, Natural seawater system at pH=2. e, Natural seawater system at pH=8. f, Natural seawater system at pH=12.

The corresponding discussions had been added in the revised Manuscript and revised Supplementary Information. Detailed revision is as below. Thank you most sincerely!

In the revised Manuscript:

(Page 9) *Notably, unlike the conventional chlor-alkali process, this entire reaction sequence proceeds without the formation of* Cl_2 *as a toxic intermediate (Supplementary Figs. 12-14) and is free from the limitations of the electrochemical window and the occurrence of inevitable side reactions.*

(Page 21) *Mass spectrometry analyses were performed using a gas chromatography-mass spectrometry system (Shimadzu, GC-MS-QP2020 NX) and electrospray ionization mass spectrometry system (Thermo Fisher, ESI-MS-Orbitrap Exploris 120).*

In the revised Supplementary Information:

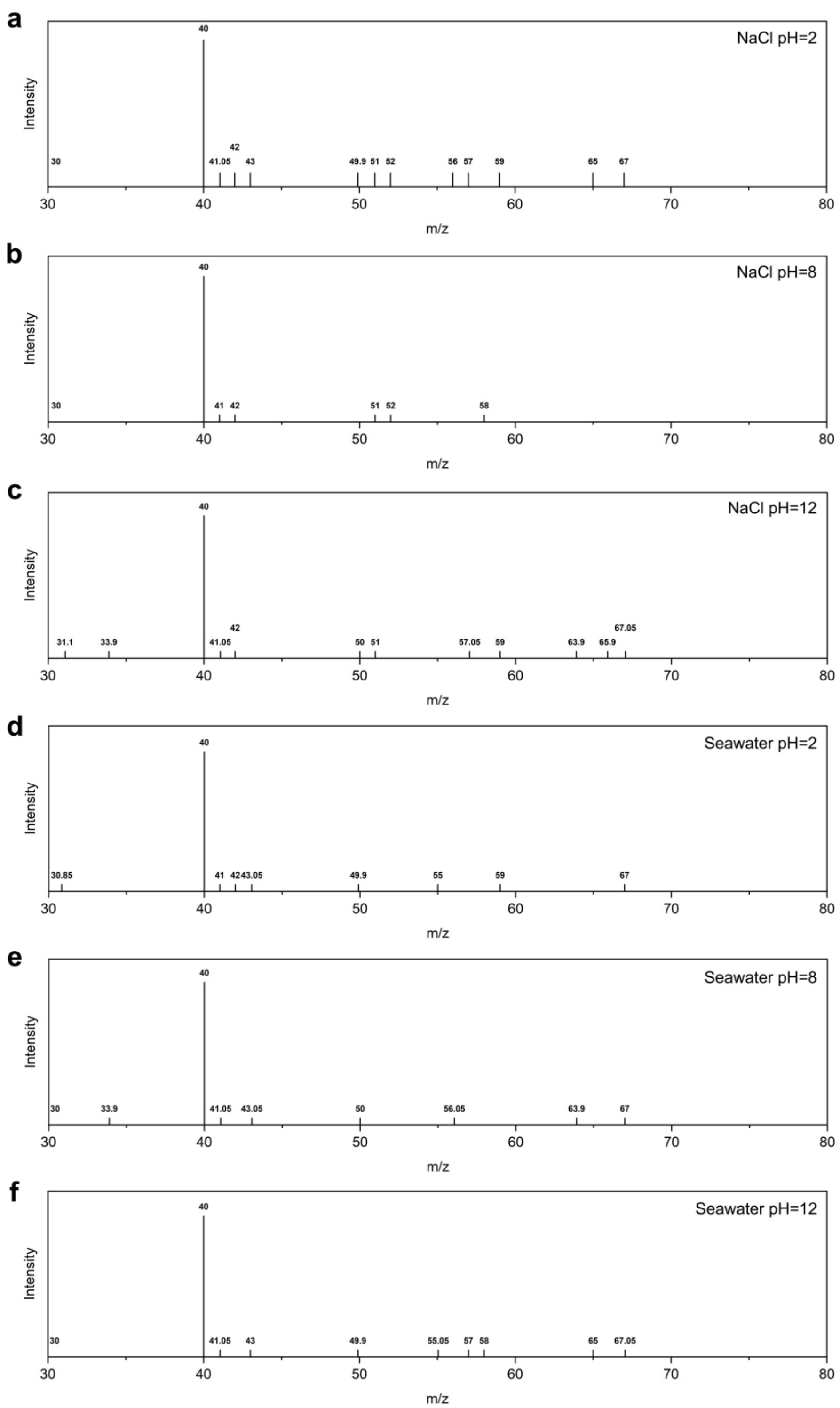

(Page 7) *Supplementary Figure 13. Headspace gas chromatography-mass spectrometry (GC-MS) of gaseous and liquid products from the reaction*

system under different conditions. a, NaCl system at pH=2. b, NaCl system at pH=8. c, NaCl system at pH=12. d, Natural seawater system at pH=2. e, Natural seawater system at pH=8. f, Natural seawater system at pH=12.

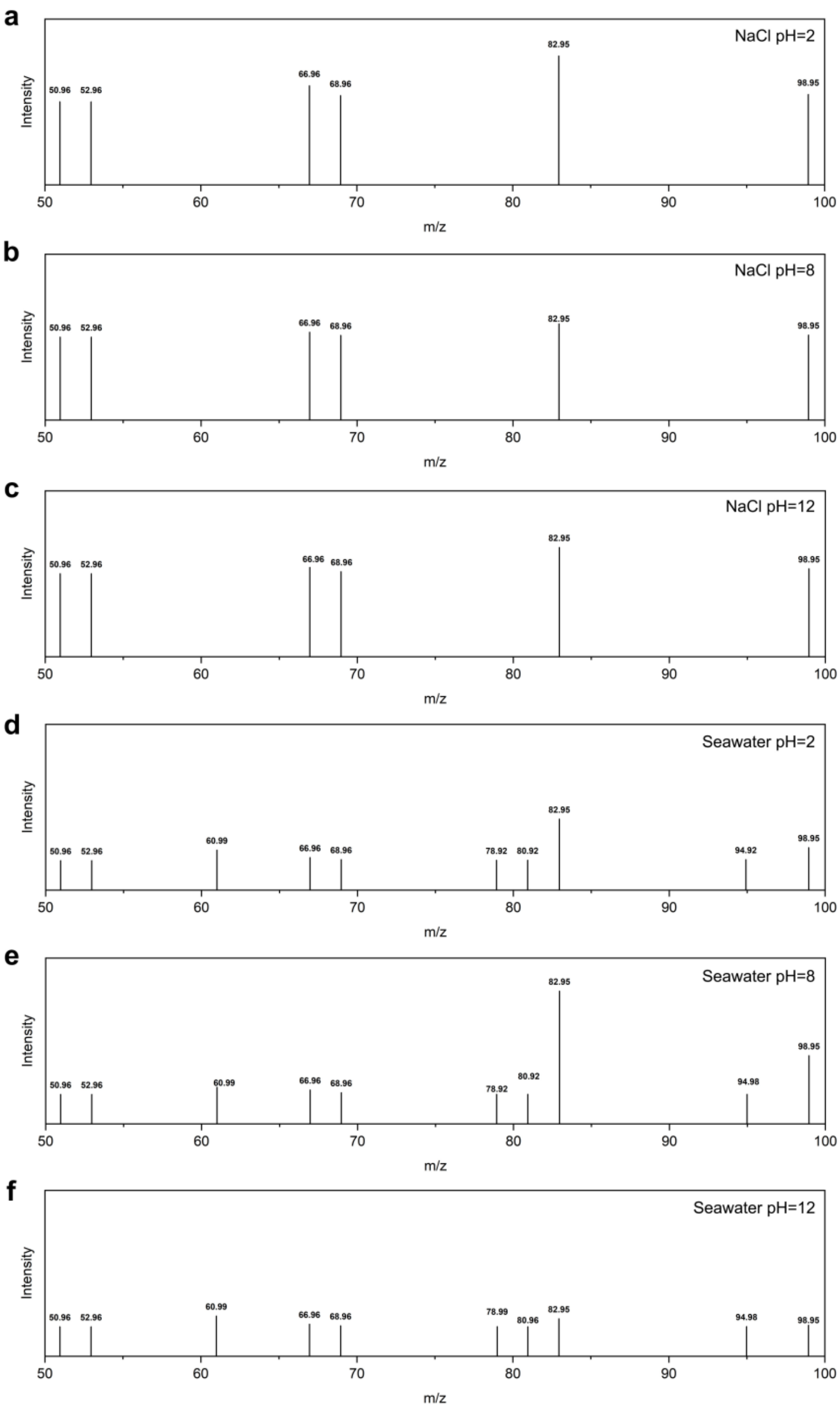

(Page 8) *Supplementary Figure 14. Electrospray ionization mass*

spectrometry (ESI-MS) of liquid products from the reaction system under different conditions. a, NaCl system at pH=2. b, NaCl system at pH=8. c, NaCl system at pH=12. d, Natural seawater system at pH=2. e, Natural seawater system at pH=8. f, Natural seawater system at pH=12.

4. The active chlorine is detected using the o-tolidine method. However, this method can be interfered by other strong oxidants which are likely generated in this CE-Chemistry system. Although MS was used for gas analysis, did the authors verify the liquid phase selectivity?

Response: We thank the reviewer for this important comment regarding the selectivity of active chlorine detection. To exclude potential interference from other oxidants generated in the CE-Chemistry system, particularly H_2O_2 formed via water oxidation (WOR) or oxygen reduction (ORR) pathways, we conducted additional control experiments. Specifically, a 3% H_2O_2 standard solution and a chloride-free system (deionized water replacing natural seawater) were tested using the same o-tolidine method. In both cases, no rapid colorimetric response characteristic of active chlorine was observed. Consistently, UV-Vis spectroscopy did not show the distinct absorption peak at 437.5 nm corresponding to active chlorine (**Fig. R5**). These results demonstrate that, in the absence of chloride ions, other oxidants present in the system do not

produce the same detection signal. Together with the established selectivity of the o-tolidine method reported in the literature (*Nat. Sustain., 2025, 8, 672-681; Angew. Chem. Int. Ed., 2026, 65, e18395; ACS Nano, 2025, 19, 25007-25016; Talanta, 2002, 58, 995-1001*), these findings confirm that the detected signal originates from active chlorine species, thereby validating the liquid-phase selectivity of our system.

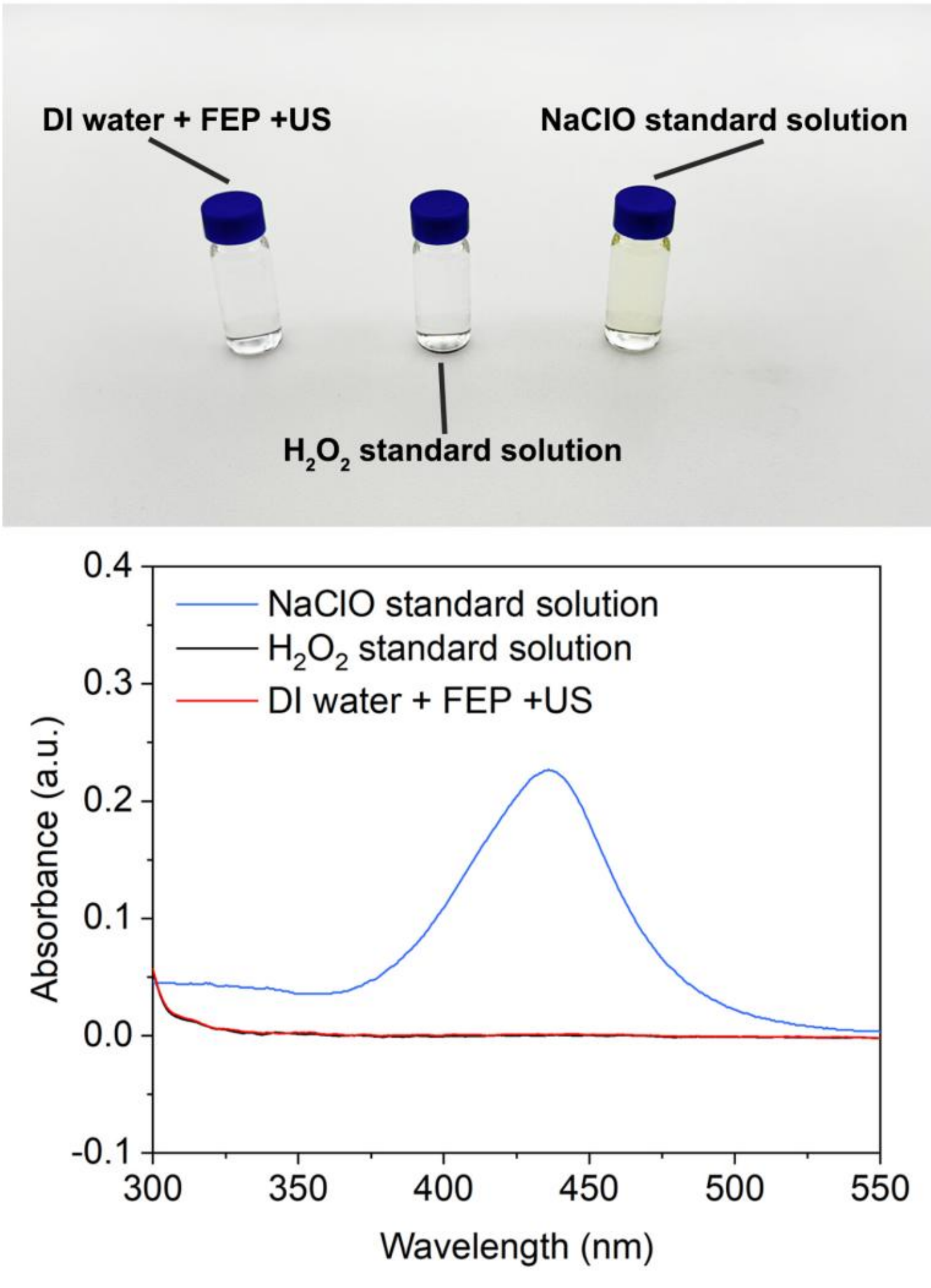

Fig. R5. Control experiments validating the accuracy of the o-tolidine method for active chlorine detection and excluding interference from other factors.

In addition, regarding reaction selectivity, headspace GC-MS analysis presented in the original manuscript, covering both gaseous and liquid-phase products, shows no detectable signals attributable to Cl_2 in either the NaCl system or natural seawater across a range of pH conditions (**Fig. R6**). To further substantiate this, we performed electrospray ionization mass spectrometry (ESI-MS) of the liquid-phase products, which likewise reveals no characteristic signals associated with dissolved Cl_2 (**Fig. R7**). Taken together, these complementary analyses consistently confirm the absence of Cl_2 formation, supporting the high selectivity of the process and highlighting its potential as a scalable and environmentally benign route for the direct production of disinfectants from brine.

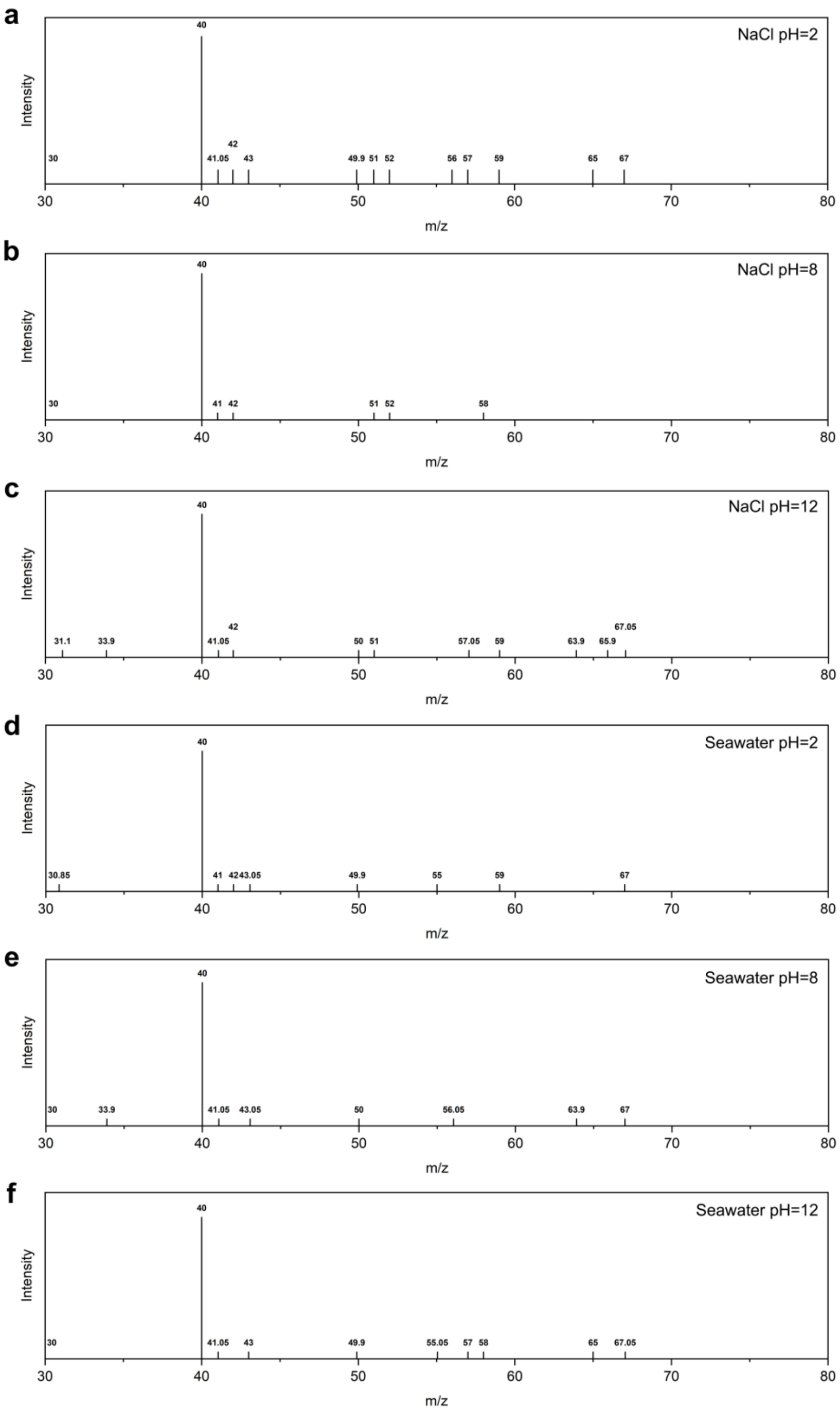

Fig. R6. Headspace gas chromatography-mass spectrometry (GC-MS) of gaseous and liquid products from the reaction system under different conditions. a, NaCl system at pH=2. b, NaCl system at pH=8. c, NaCl system at pH=12. d, Natural seawater system at pH=2. e, Natural seawater system at pH=8. f, Natural seawater system at pH=12.

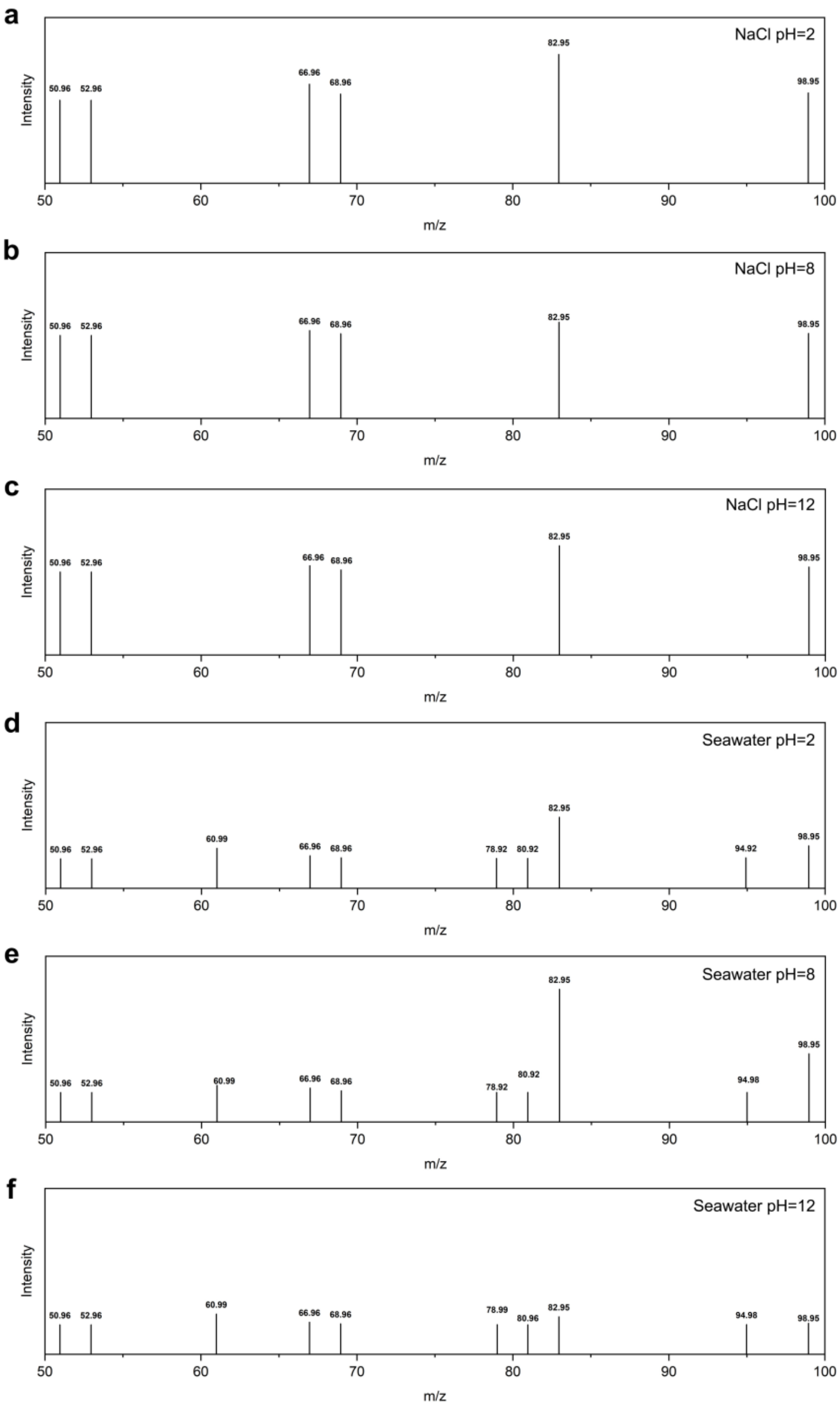

Fig. R7. Electrospray ionization mass spectrometry (ESI-MS) of liquid products from the reaction system under different conditions. a, NaCl system at pH=2. b, NaCl system at pH=8. c, NaCl system at pH=12. d, Natural seawater system at pH=2. e, Natural seawater system at pH=8. f, Natural seawater system at pH=12.

The corresponding discussions had been added in the revised Manuscript and revised Supplementary Information. Detailed revision is as below. Thank you most sincerely!

In the revised Manuscript:

(Page 9) *Notably, unlike the conventional chlor-alkali process, this entire reaction sequence proceeds without the formation of Cl_2 as a toxic intermediate (Supplementary Figs. 12-14) and is free from the limitations of the electrochemical window and the occurrence of inevitable side reactions.*

(Page 21) *Mass spectrometry analyses were performed using a gas chromatography-mass spectrometry system (Shimadzu, GC-MS-QP2020 NX) and electrospray ionization mass spectrometry system (Thermo Fisher, ESI-MS-Orbitrap Exploris 120).*

In the revised Supplementary Information:

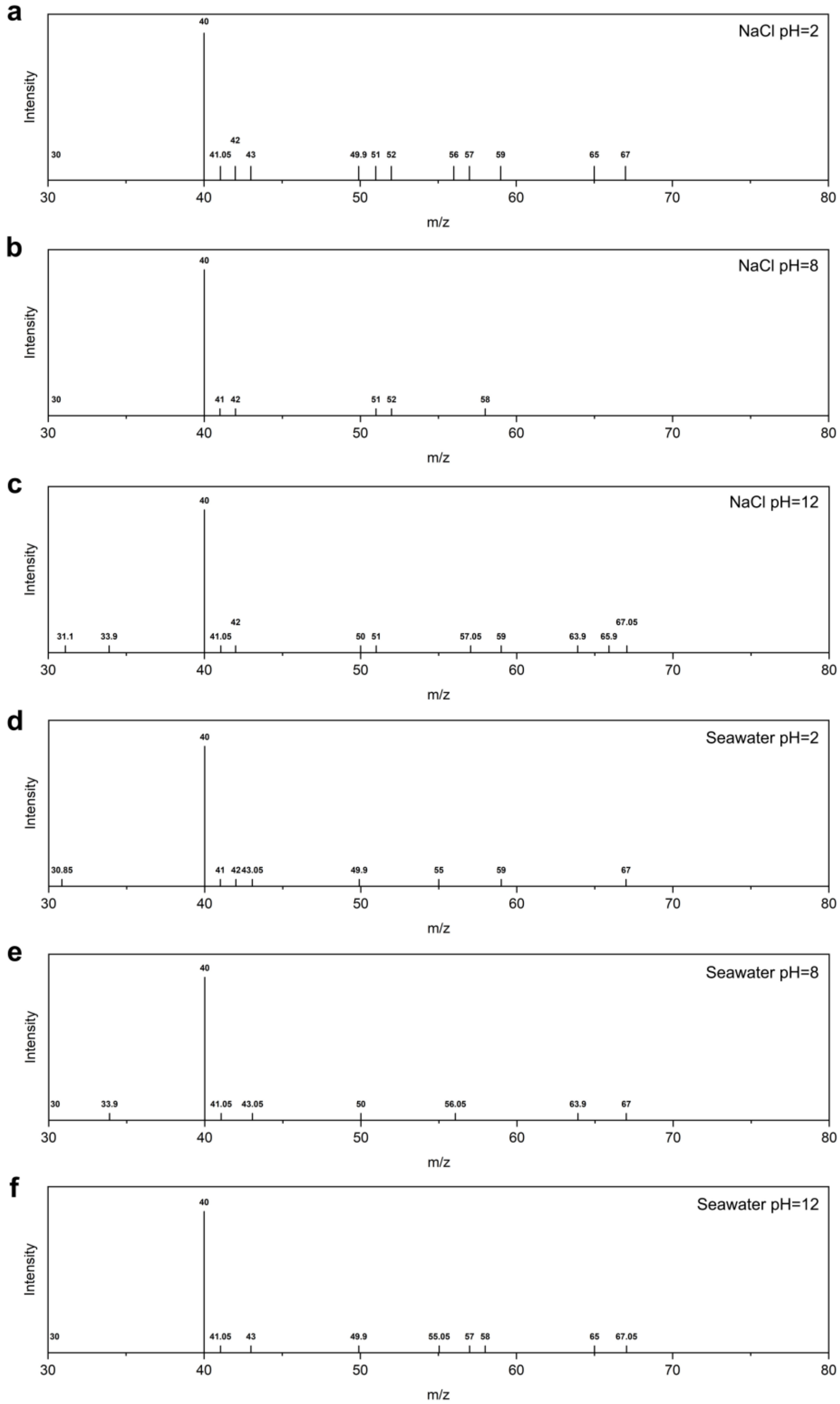

(Page 7) *Supplementary Figure 13. Headspace gas chromatography-mass spectrometry (GC-MS) of gaseous and liquid products from the reaction system under different conditions. a, NaCl system at pH=2. b, NaCl system*

at pH=8. c, NaCl system at pH=12. d, Natural seawater system at pH=2. e, Natural seawater system at pH=8. f, Natural seawater system at pH=12.

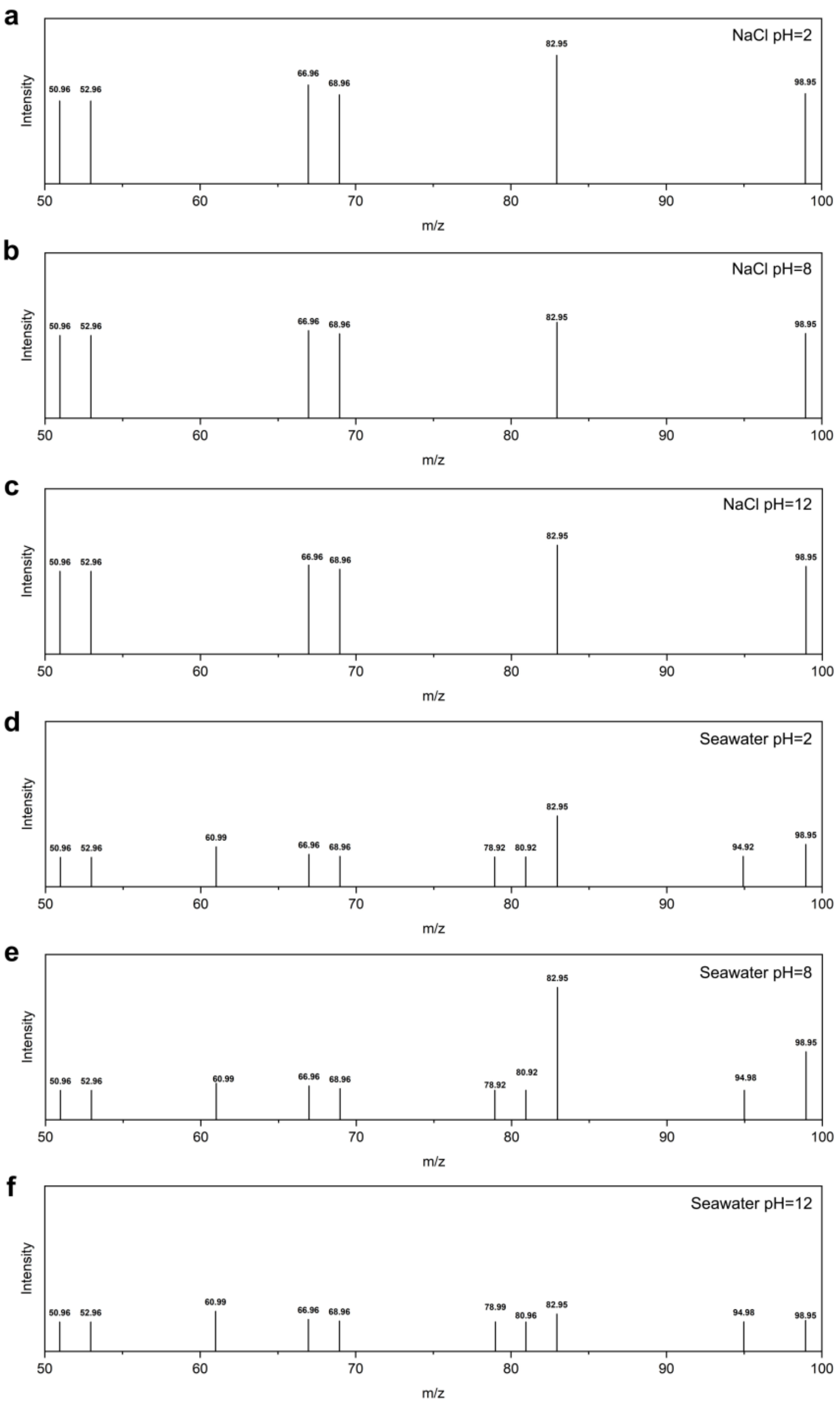

(Page 8) *Supplementary Figure 14. Electrospray ionization mass spectrometry (ESI-MS) of liquid products from the reaction system under*

different conditions. a, NaCl system at pH=2. b, NaCl system at pH=8. c, NaCl system at pH=12. d, Natural seawater system at pH=2. e, Natural seawater system at pH=8. f, Natural seawater system at pH=12.

5. One of the main points of this work is "sustainability." However, the system operates at 120 W (ultrasonic power) to treat a relatively small volume with 20 mg of catalyst. The authors should calculate the energy consumption per mole of active chlorine produced (kWh/mol or kWh/kg) and compare it with the industrial chlor-alkali process and other emerging electrochemical seawater splitting technologies. If the energy cost is much higher than those, the claim of a "sustainable paradigm" needs to be justified.

Response: We sincerely thank the reviewer for this insightful comment regarding energy efficiency and sustainability. In response, we quantified the energy consumption of the system under optimized conditions: with 20 mg FEP in 10 mL of natural seawater subjected to 120 W ultrasonication for 1 h, the active chlorine yield reaches 208.8 μmol g^{-1} h^{-1}, corresponding to an absolute production rate of ~4.18 μmol h^{-1}. This translates to an energy consumption of ~3.9 × 10^5 kWh kg^{-1} active chlorine. For comparison, this is considerably higher than the industrial chlor-alkali process or representative emerging systems such as $NbClO_x/BiVO_4$ (*Nat.*

Sustain., 2025, 8, 672-681; Nat. Sustain., 2024, 7, 148-157). We therefore fully agree that, in its current batch ultrasonication configuration, CE-Chemistry does not yet compete in terms of unit-product energy efficiency.

However, it is important to clarify that the sustainability discussed in our work does not refer to energy efficiency per unit product, but rather to the process itself: ***CE-Chemistry generates active chlorine without producing toxic Cl_2, avoids noble-metal catalysts or external electrical bias, and operates directly in complex media such as natural seawater.*** In this sense, the system represents a fundamental scientific advance, overcoming limitations inherent to conventional chlorination methods.

Moreover, while CE-Chemistry currently requires more energy per unit product than industrial processes, the energy input does not need to come from an external power source. ***The system relies on contact-separation-induced contact electrification, which can, in principle, be driven by low-frequency mechanical energy naturally present in marine environments (e.g., waves, fluid flow, or vibrations)***. In particular, marine wave energy alone has an annual potential exceeding 10,000 TWh, highlighting the great promise of the ocean as a sustainable energy source (*Ocean, 2025, 1, 9470004; Adv. Mater., 2025, 37, e11283*). If realized, this approach could decouple chemical production from conventional high-energy electrical inputs, achieving active chlorine synthesis through

ubiquitous ambient energy, an opportunity that conventional high-efficiency methods cannot access.

Such oceanic mechanical energy could, in principle, directly drive CE-Chemistry for green active chlorine production or, via energy-harvesting technologies, be converted to electrical power for downstream processes (*Nano-Micro Lett., 2025, 17, 124; Adv. Mater., 2025, 37, e11283*). Therefore, the system's most promising applications are not in centralized chlorine manufacture, but in distributed scenarios inaccessible to traditional chlor-alkali methods, e.g., ship-surface coatings, marine equipment interfaces, coastal or small-scale water-treatment units, and emergency disinfection, where ambient waves or fluid motion continuously trigger CE-Chemistry reactions for localized, on-demand antibacterial and anticorrosion functions. Accordingly, the sustainability of this work is better reflected in its novel reaction pathway and application mode, rather than in unit-product energy consumption at this stage.

Accordingly, we have revised the manuscript to moderate claims regarding sustainability. The work is now presented as a proof-of-concept interfacial chemistry platform that introduces a Cl_2-free reaction pathway and highlights a fundamentally new mode of energy-chemistry coupling, rather than as an immediate energy-efficient alternative to established industrial processes. We sincerely appreciate the reviewer's suggestion,

which has strengthened both the scientific clarity and positioning of the manuscript. The corresponding discussions have been revised accordingly.

The corresponding discussions had been moderated and revised in the revised Manuscript. Detailed revisions are as below. Thank you most sincerely!

In the revised Manuscript:

(Page 2) *Here, we reframe CE as a radical-generating mechanism at solid-liquid interfaces and establish contact-electro-chemistry (CE-Chemistry) as a sustainable paradigm, capable of harnessing abundant ambient mechanical energy to drive chemical transformations without the need for metal catalysts.*

(Page 2) *Utilizing a triboelectric radical generation route from natural seawater, this work demonstrates a metal-catalyst-free and sustainable strategy for active chlorine production, achieving a yield of 208.8 μmol g^{-1} h^{-1} with only 20 mg of FEP powder.*

(Page 2) *This work introduces a green and Cl_2-free route for disinfectant production directly from brine and positions CE as a general trigger for*

radical chemistry, opening avenues for heterogeneous catalysis and CE-driven reaction design.

(Page 4) *At present, industrial-scale production of active chlorine predominantly relies on the chlor-alkali process, in which chlorine gas (Cl_2) is generated as a corrosive and toxic intermediate[55,56]. Although this process is industrially mature, it faces inherent safety concerns and limitations for distributed applications: the transportation and storage of Cl_2 are subject to strict requirements and may pose serious environmental and health risks[57]. A promising pathway lies in green, metal-free synthetic strategies that bypass Cl_2 generation altogether and may enable distributed active chlorine production by harnessing high-entropy mechanical energy from the environment. By directly producing active chlorine from abundant natural seawater, this strategy circumvents the dependence on high-purity water and the hazards of Cl_2, while enabling green and sustainable distributed production[58,59].*

(Page 5) *This work delivers a safe, sustainable, and environmentally benign route for distributed active chlorine production directly from seawater, circumventing the limits of conventional electrochemical windows, while also revealing the central role of interfacial triboelectric*

fields in CE-Chemistry and demonstrating how field strength and reaction pathways can be precisely tuned through regulation of the EDL.

(Page 15) *Such a route may be particularly relevant for niche scenarios that are difficult to address by centralized chlor-alkali infrastructure, such as marine equipment interfaces, ship coatings, localized antibiofouling/anticorrosion systems, and small-scale or emergency disinfection units.*

(Page 15) *This work provides a metal-free, CE-driven proof-of-concept for interfacial chemical transformations, with the potential to enable distributed reactions in the future by harnessing high-entropy mechanical energy from the environment.*

6. Ultrasonication is known to generate significant heat in the bulk solution over time, yet the manuscript does not specify the temperature control measures during the long-term (700 h) experiments. The authors acknowledge that HOCl decomposition is accelerated at high temperatures. Furthermore, the dielectric constant and triboelectric charge density of polymers like FEP are often temperature-dependent. The authors need to clarify if the reaction temperature was thermostated. If not, how much of

the yield fluctuation is influenced by the thermal degradation of HOCl (Equation 10) versus the generation rate?

Response: We thank the reviewer for this insightful comment. In our ultrasonication experiments, including long-term (700 h) experiments, the reaction temperature was actively thermostated using a temperature-control system integrated with the ultrasonic setup, and the bulk solution temperature was maintained at 20 °C throughout the operation (**Fig. R8**). We have now clarified this point in the revised Manuscript and Supplementary Information, providing a photograph of the temperature-controlled setup. By maintaining a constant temperature, we minimize both the thermal decomposition of HOCl (Equation 10) and any temperature-dependent variations in the dielectric properties and triboelectric charge density of FEP. Therefore, the observed yield trends primarily reflect changes in the interfacial radical generation/production kinetics rather than thermal effects.

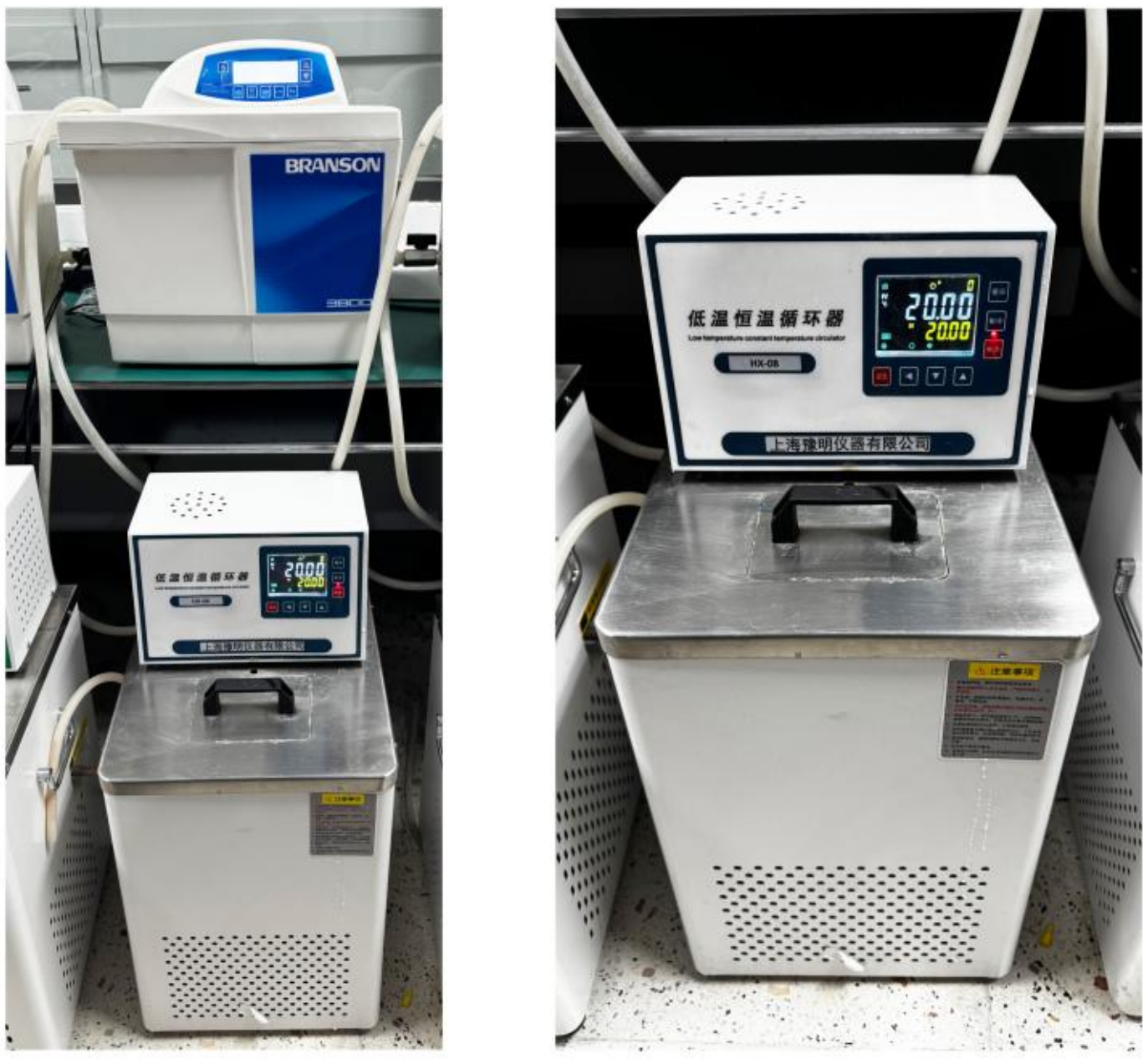

Fig. R8. Temperature-controlled ultrasonication setup enabling constant-temperature operation.

The corresponding discussions had been added in the revised Manuscript and revised Supplementary Information. Detailed revision is as below. Thank you most sincerely!

In the revised Manuscript:

(Page 20) *A thermostatic ultrasonic bath (40 kHz, 120 W) was used to facilitate the CE-Chemistry reactions while maintaining the solution temperature to 20 °C (Supplementary Fig. 31).*

In the revised Supplementary Information:

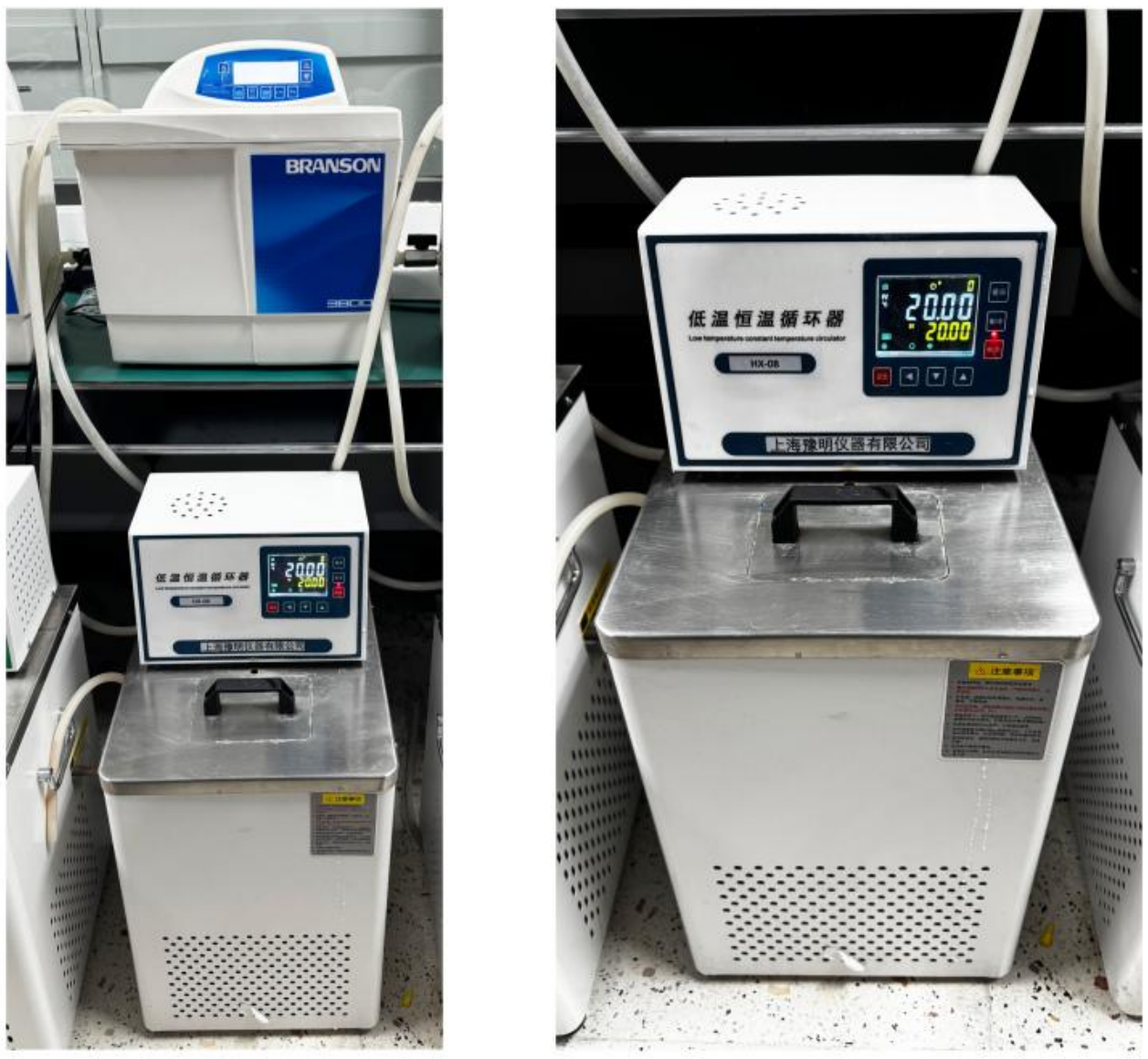

(Page 17) *Supplementary Figure 31. Temperature-controlled ultrasonication setup enabling constant-temperature operation.*

7. The yield is shown to decrease when FEP dosage exceeds 20 mg, attributed to ultrasonic scattering. However, the manuscript does not provide the specific surface area ($m^2\ g^{-1}$) of the FEP powder used. Since CE-Chemistry is strictly an interfacial phenomenon, the reaction rate should be normalized by the active surface area rather than mass to allow for reproducible comparisons.

Response: We sincerely thank the reviewer for this insightful comment. In response, we have measured the BET surface area of the FEP powder used

in this study, which is 3.4372 m^2 g^{-1} (**Fig. R9**). We fully agree that CE-Chemistry is fundamentally an interfacial phenomenon, and that normalization by active surface area would, in principle, provide a more rigorous basis for comparison. However, given the very low specific surface area of the FEP powder, both previous studies and the present work adopt powder mass as a practical and widely accepted parameter for performance benchmarking (*Nat. Energy, 2023, 8, 1137; Adv. Mater., 2023, 35, 2304387; Angew. Chem. Int. Ed., 2024, 63, e202403114; J. Am. Chem. Soc., 2025, 147, 25407*). We note that when the powder dosage exceeds the amount required to cover the liquid surface, the reaction efficiency no longer increases with mass. Instead, the yield decreases due to enhanced ultrasonic scattering and particle aggregation, which reduce the effective utilization of the solid-liquid interface. This explains the observed trend of an initial increase followed by a decrease in yield. While the current FEP powder has a relatively low surface area, this comment highlights an important direction for future development: selecting or designing materials with higher specific surface areas and more accessible interfacial sites to maximize interfacial utilization. We anticipate that such material optimization will significantly enhance the activity, efficiency, and overall performance of CE-Chemistry.

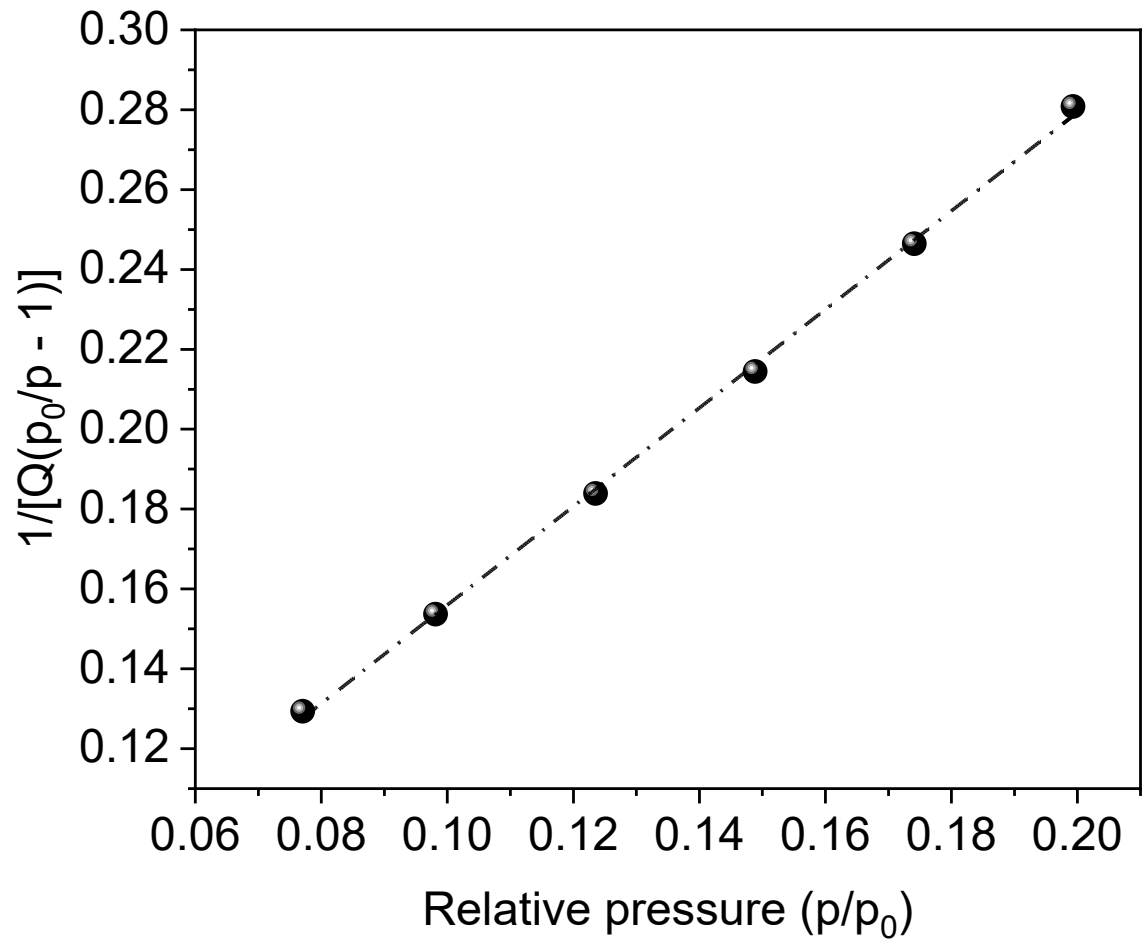

Fig. R9. BET surface area plot.

8. Are the chlorine species concentrations in Supplementary Figures 24 and 25 simulated values or that of experiments? Could the experimental values for actual LiCl be provided?

Response: We thank the reviewer for this valuable comment. The ion distributions within the EDL and the corresponding interfacial electric field, as presented in Supplementary Figures 24 and 25, are derived from COMSOL simulations rather than direct measurement. In our ultrasonication-driven solid-liquid system, the spatial scale of the EDL is typically below 1 nm and highly transient due to cavitation, making *in situ* experimental probing extremely challenging (*Chem. Soc. Rev., 2024, 53, 4349-4373; Chem. Rev. 2022, 122, 5209-5232; Acc. Chem. Res., 2026, 59, 285-297; Chem. Soc. Rev., 10.1039/d5cs01066g*). Direct experimental

quantification for the LiCl, NaCl, and KCl systems was not feasible, therefore, COMSOL simulations were employed to estimate and analyze the ion distribution within the EDL and the associated interfacial electric field.

To improve reliability, we incorporated experimental measurements using a confocal Raman-NaSCN probe, which *ex situ* determined the interfacial electric field to be on the order of 10^{10} V m^{-1} (**Fig. R10 and R11**). These values were used to calibrate the simulation parameters, refining the EDL structure and ion distribution to better reflect the actual system (**Fig. R12-R16**). We emphasize that, while precise operando mapping of ions remains a challenge, this combined simulation, experiment approach provides the most representative description currently achievable, and future advances in high-resolution techniques may allow direct validation.

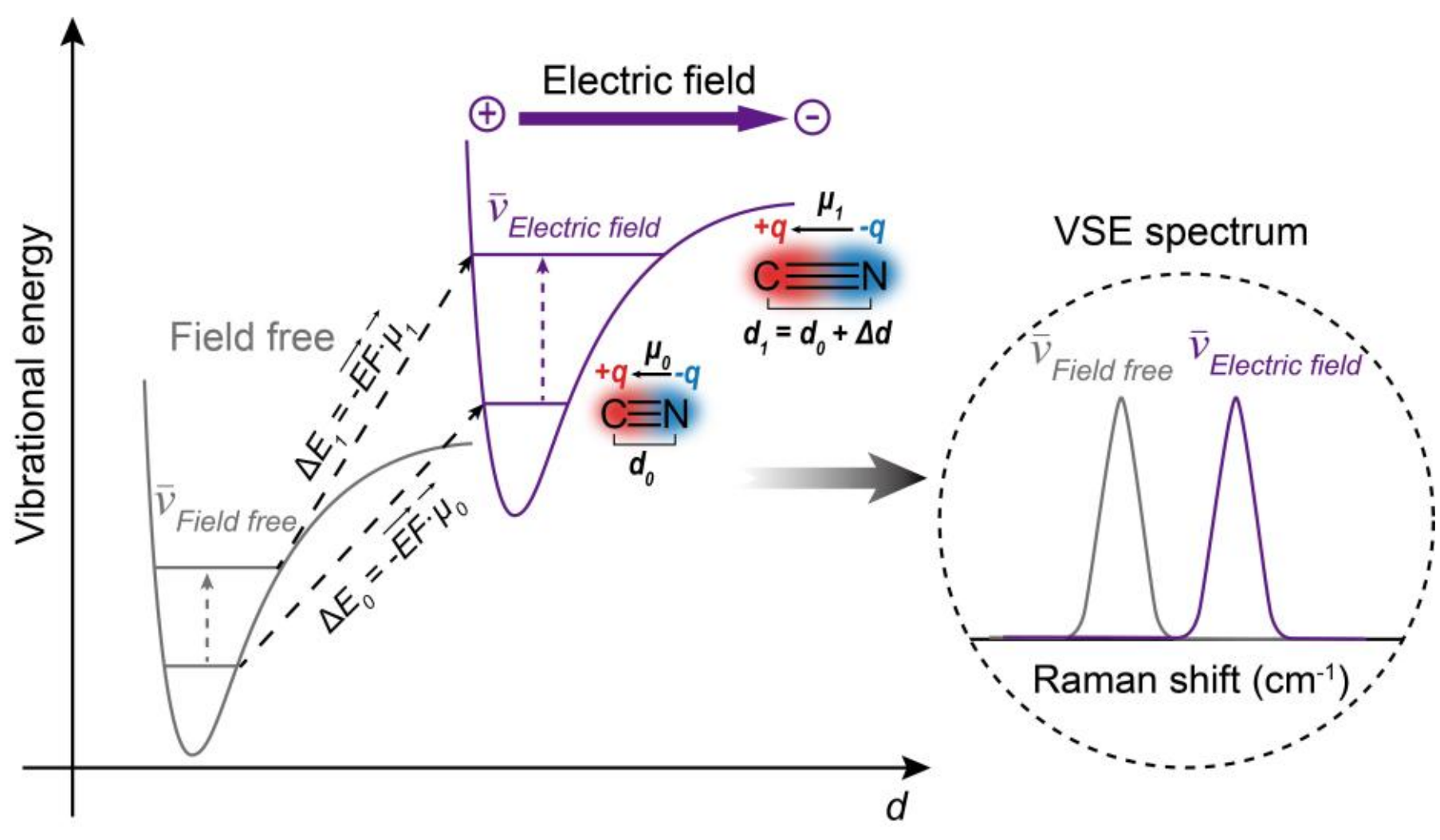

Fig. R10. Illustration of vibrational Stark effect (VSE).

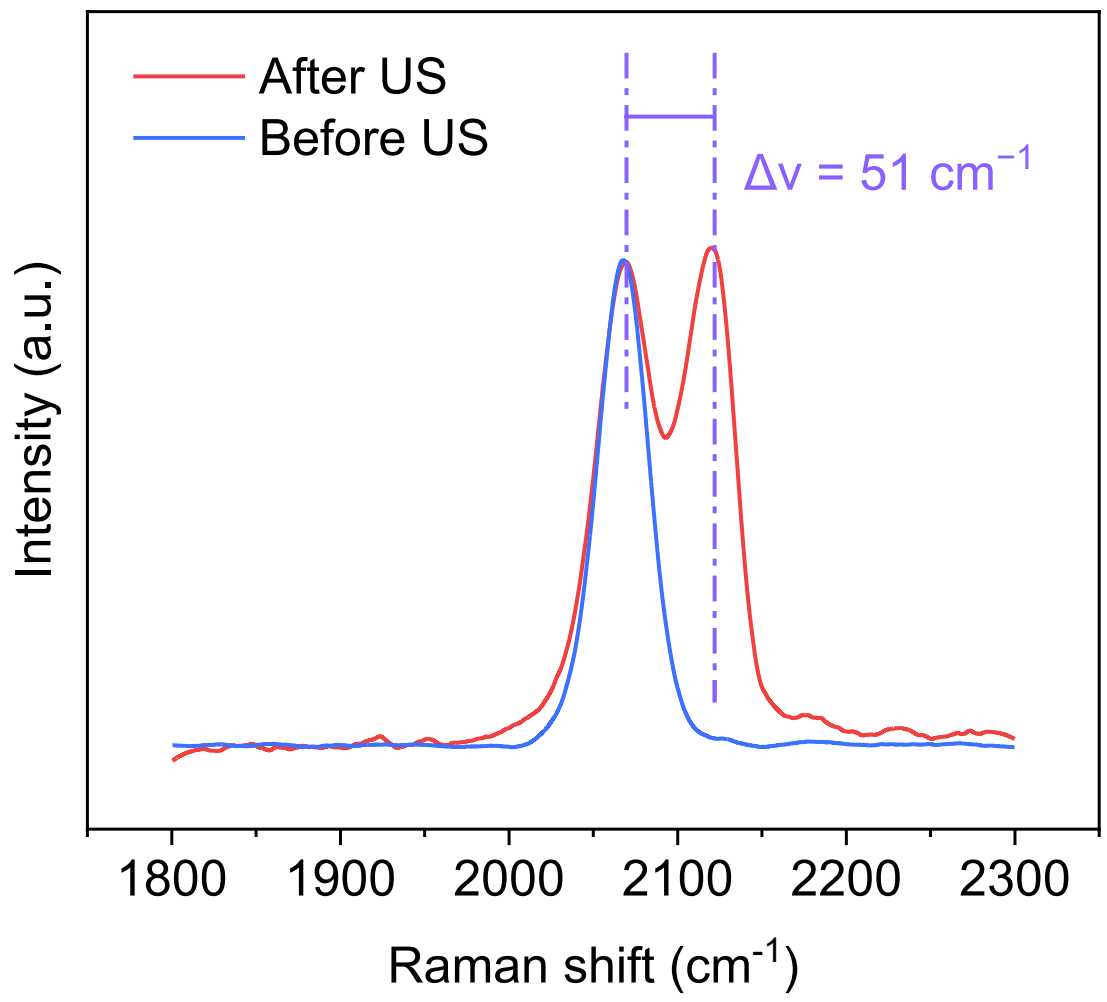

Fig. R11. The dedicated confocal Raman-NaSCN probe platform demonstrates that the triboelectric field at the solid-liquid interface can reach 1.4×10^{10} V m^{-1}.

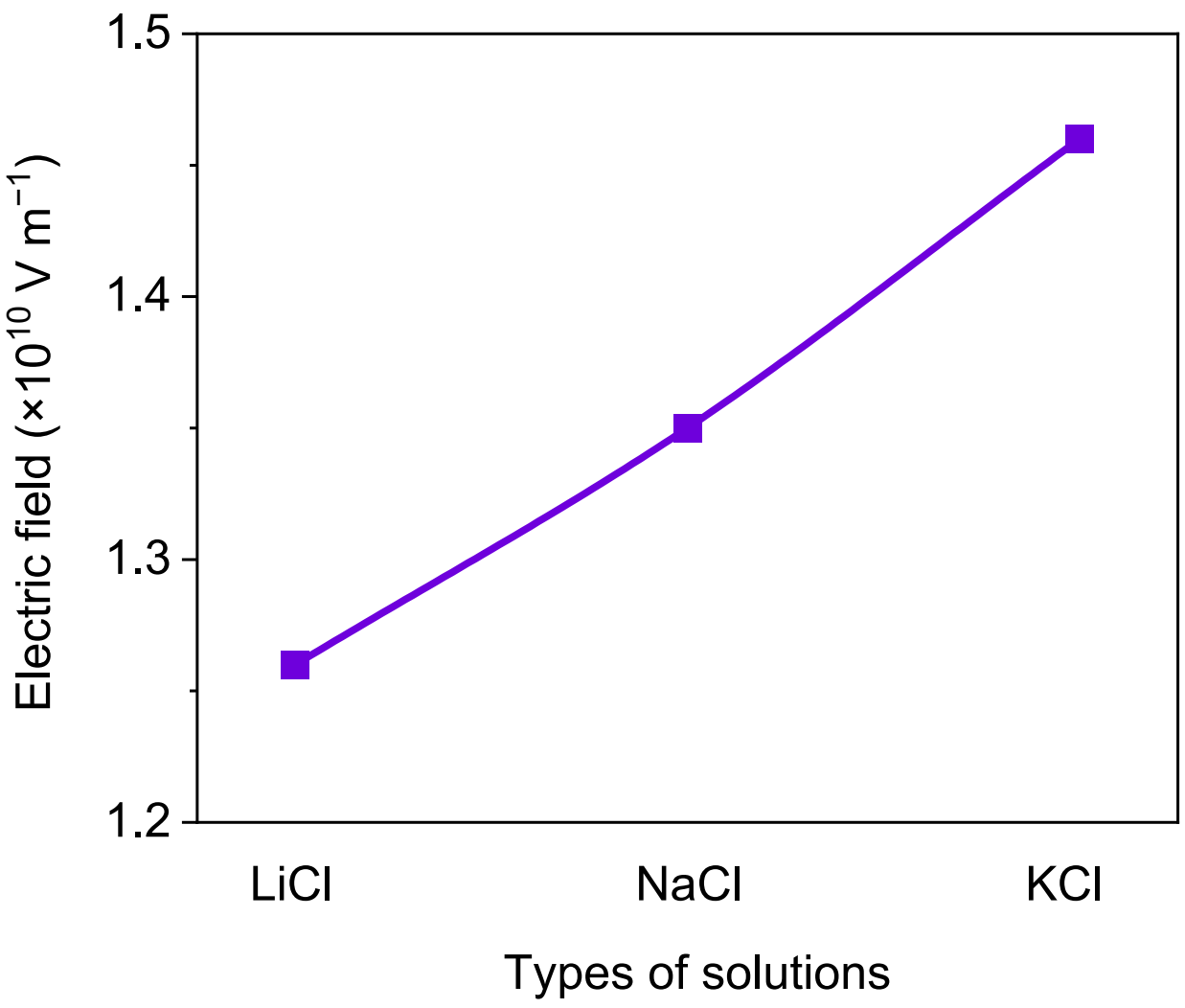

Fig. R12. Comparison of interfacial triboelectric fields within the Stern layer in different salt solutions.

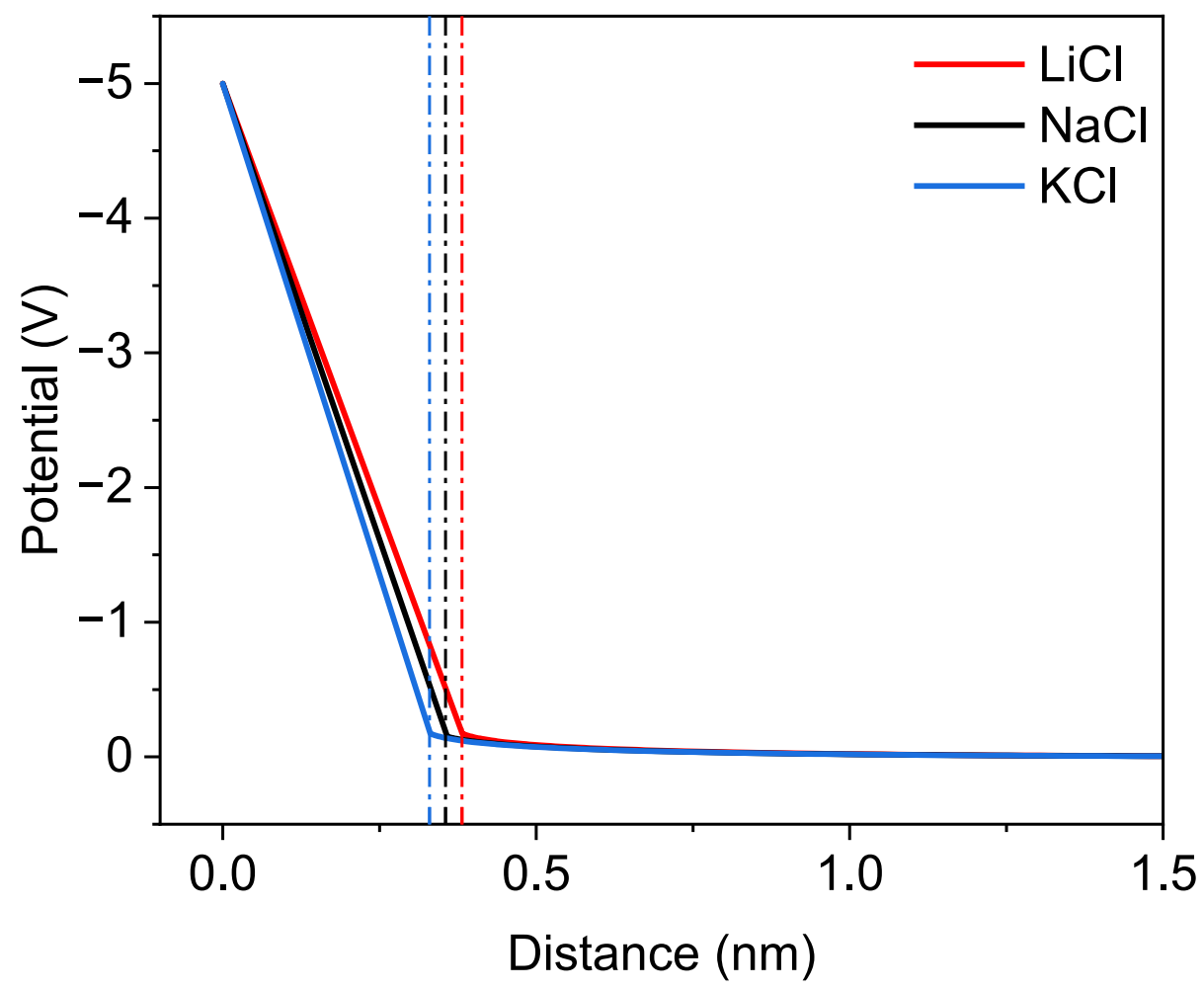

Fig. R13. The variation of the EDL potential with distance in different salt solutions.

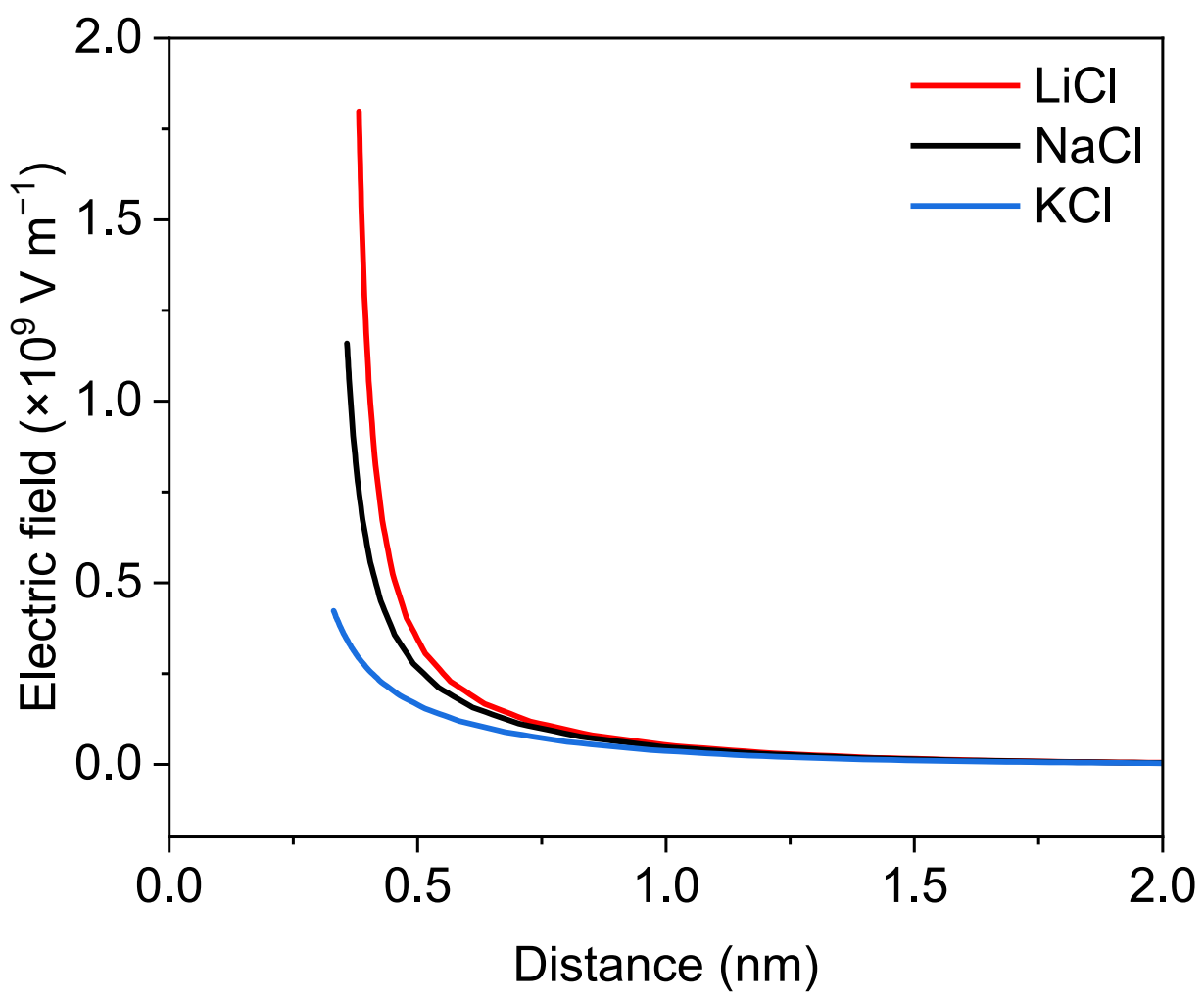

Fig. R14. The distance-dependent interfacial triboelectric field in the diffusion layer of various salt solutions.

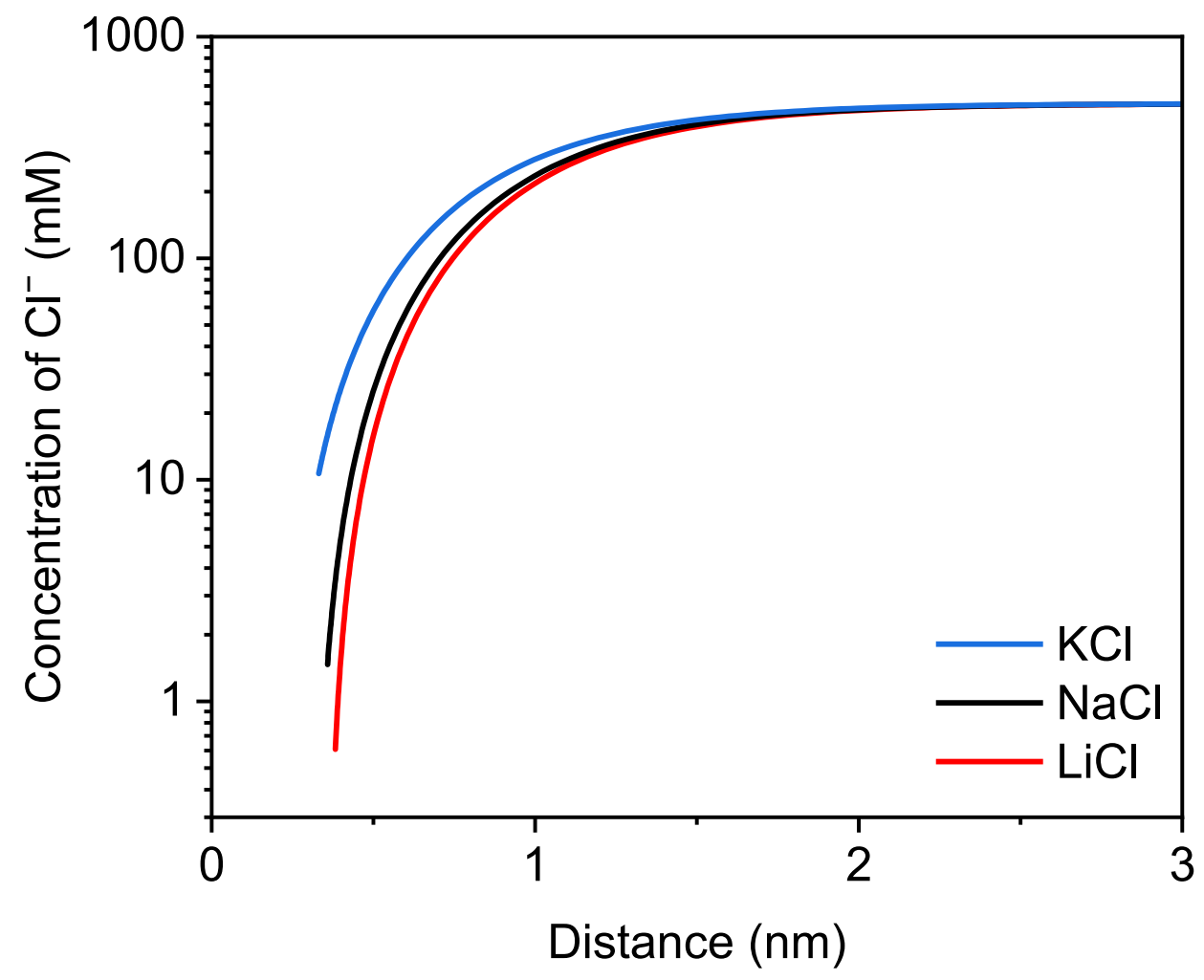

Fig. R15. Variation of the Cl^- concentration with distance in the diffusion layer.

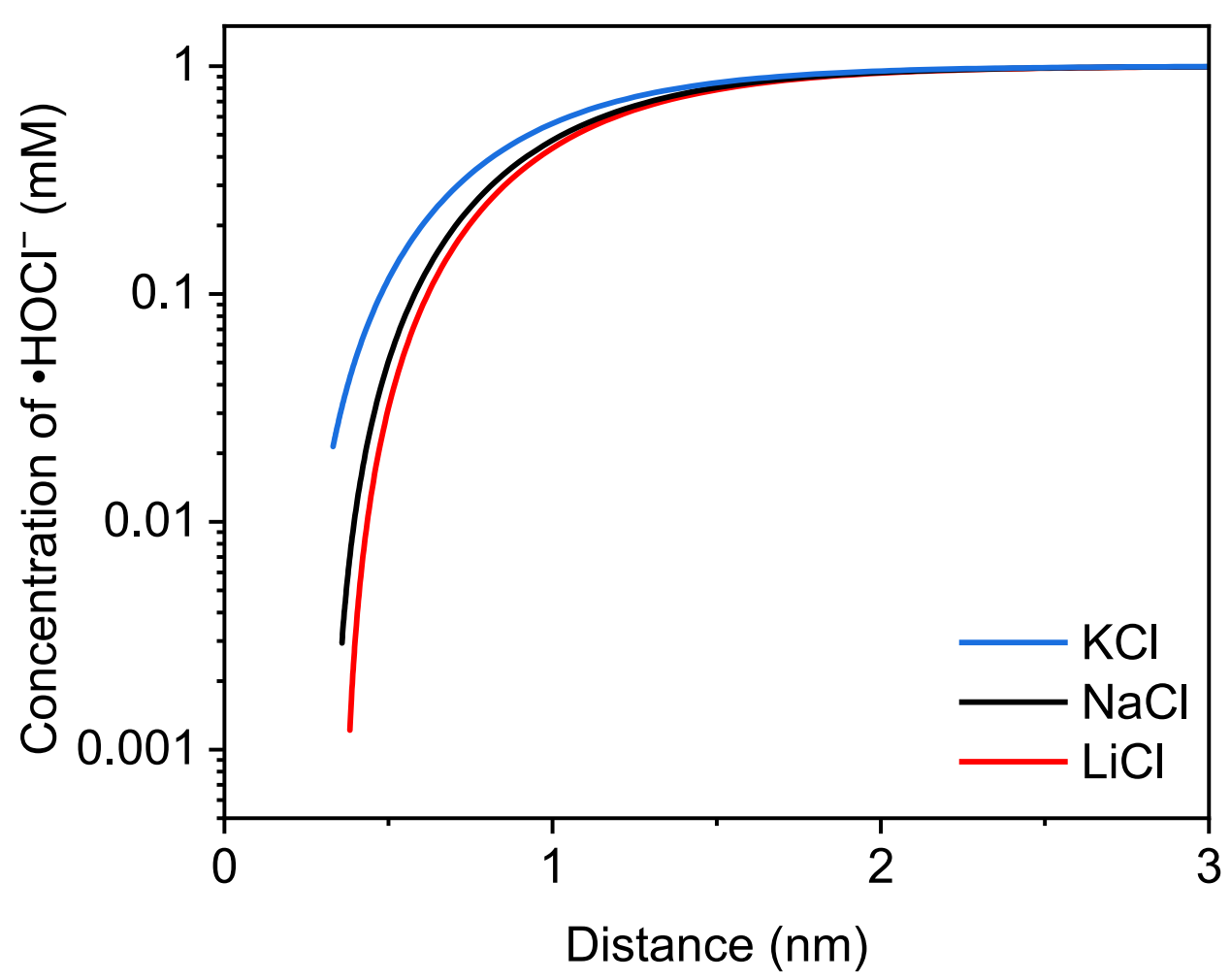

Fig. R16. Variation of the $\bullet HOCl^-$ concentration with distance in the diffusion layer.

The corresponding discussions had been added in the revised Manuscript and revised Supplementary Information. Detailed revision is as below. Thank you most sincerely!

In the revised Manuscript:

(Page 13) *Accordingly, under identical conditions, the EDL in KCl solution is more compact than in NaCl or LiCl solutions (Supplementary Fig. 26), with the interfacial triboelectric field within the Stern layer estimated at 1.46×10^{10} V m^{-1}, exceeding that of NaCl (1.35×10^{10} V m^{-1}) and LiCl (1.26×10^{10} V m^{-1}) (Fig. 4f).*

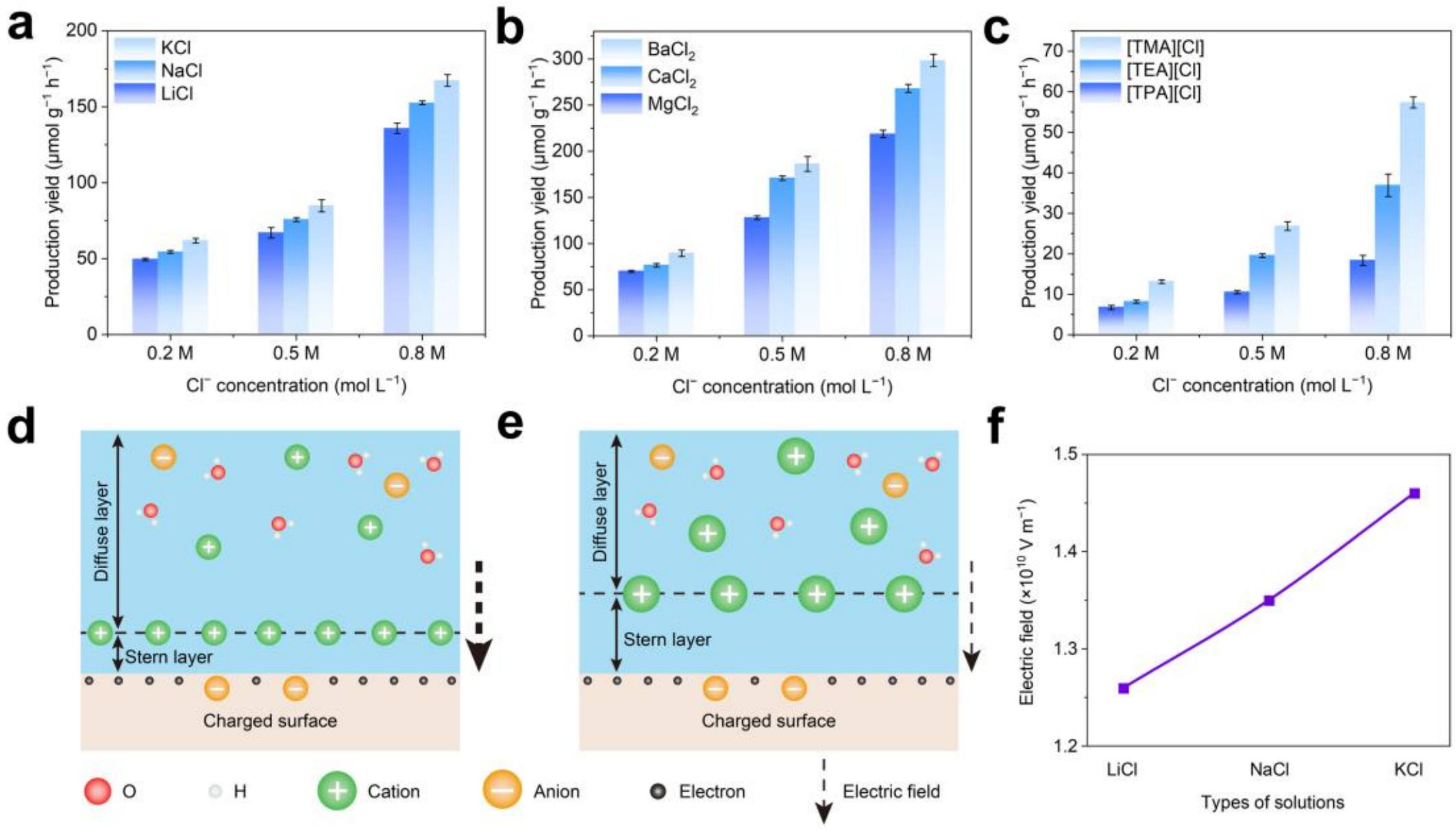

(Page 28) *Fig. 4 Proposed model of the impact of cation effect on CE-Chemistry. f, Comparison of interfacial triboelectric fields within the Stern layer in different salt solutions.*

In the revised Supplementary Information:

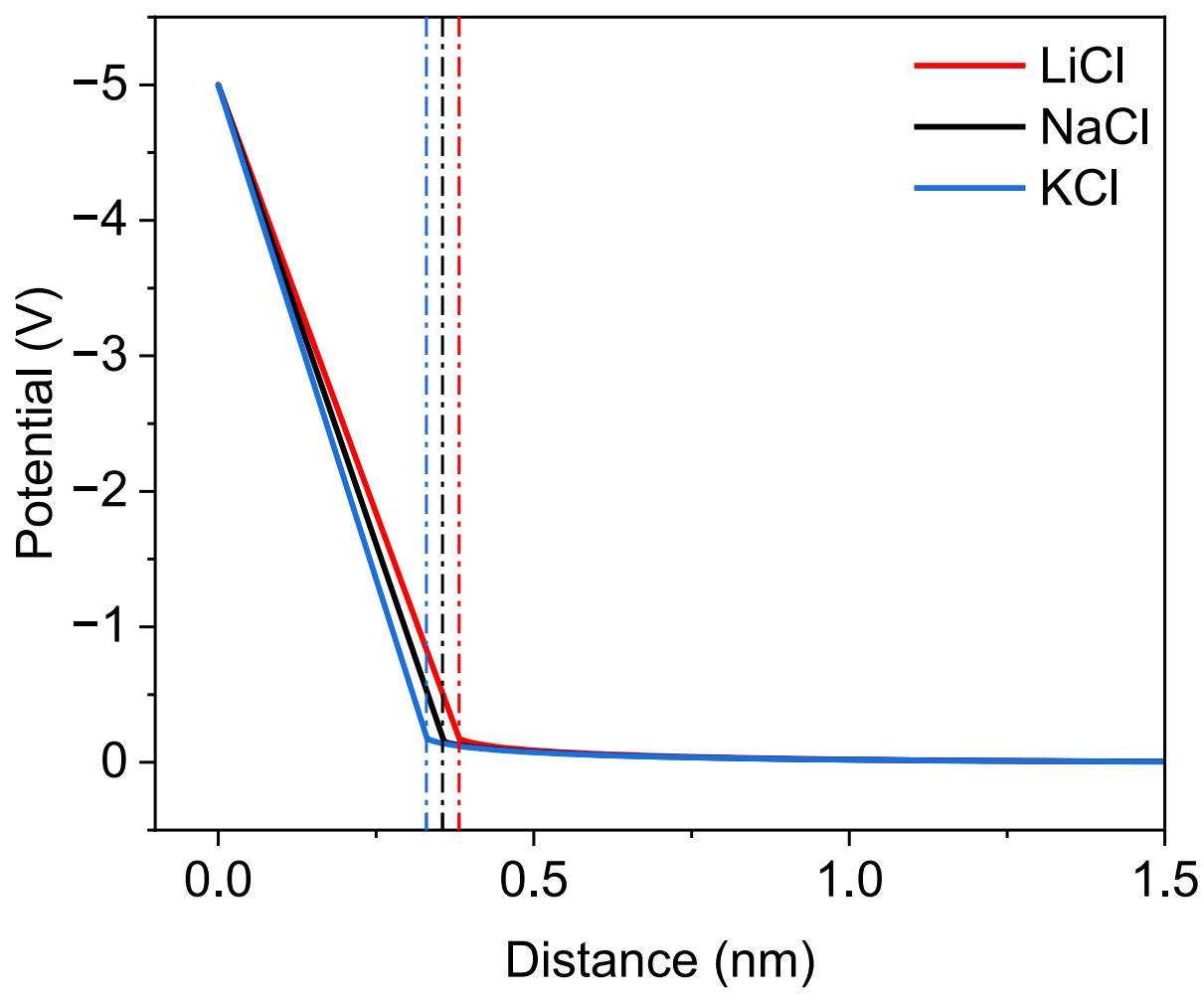

(Page 14) *Supplementary Figure 26. The variation of the EDL potential with distance in different salt solutions.*

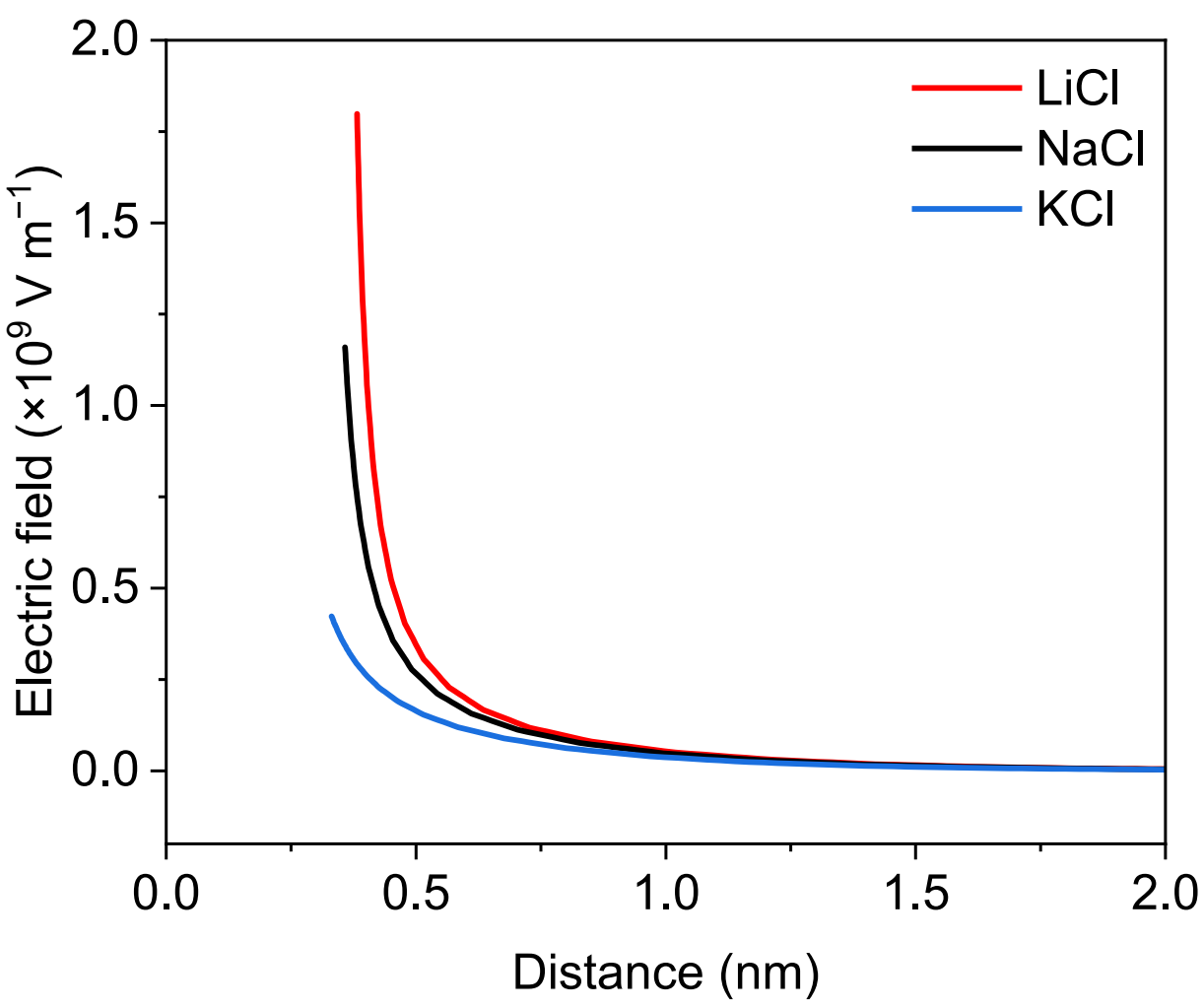

(Page 15) *Supplementary Figure 28. The distance-dependent interfacial triboelectric field in the diffusion layer of various salt solutions.*

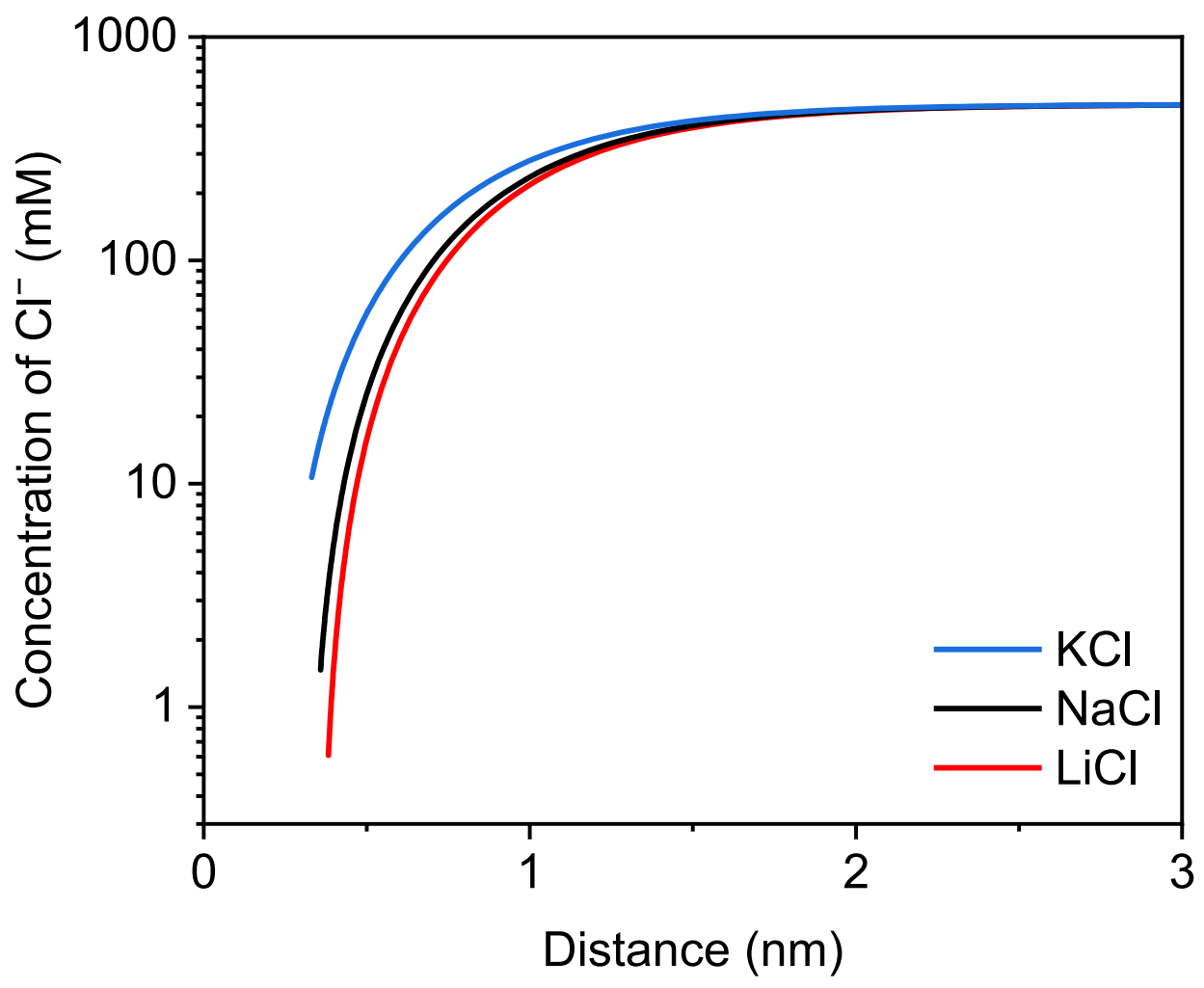

(Page 16) *Supplementary Figure 29. Variation of the Cl^{-} concentration with distance in the diffusion layer.*

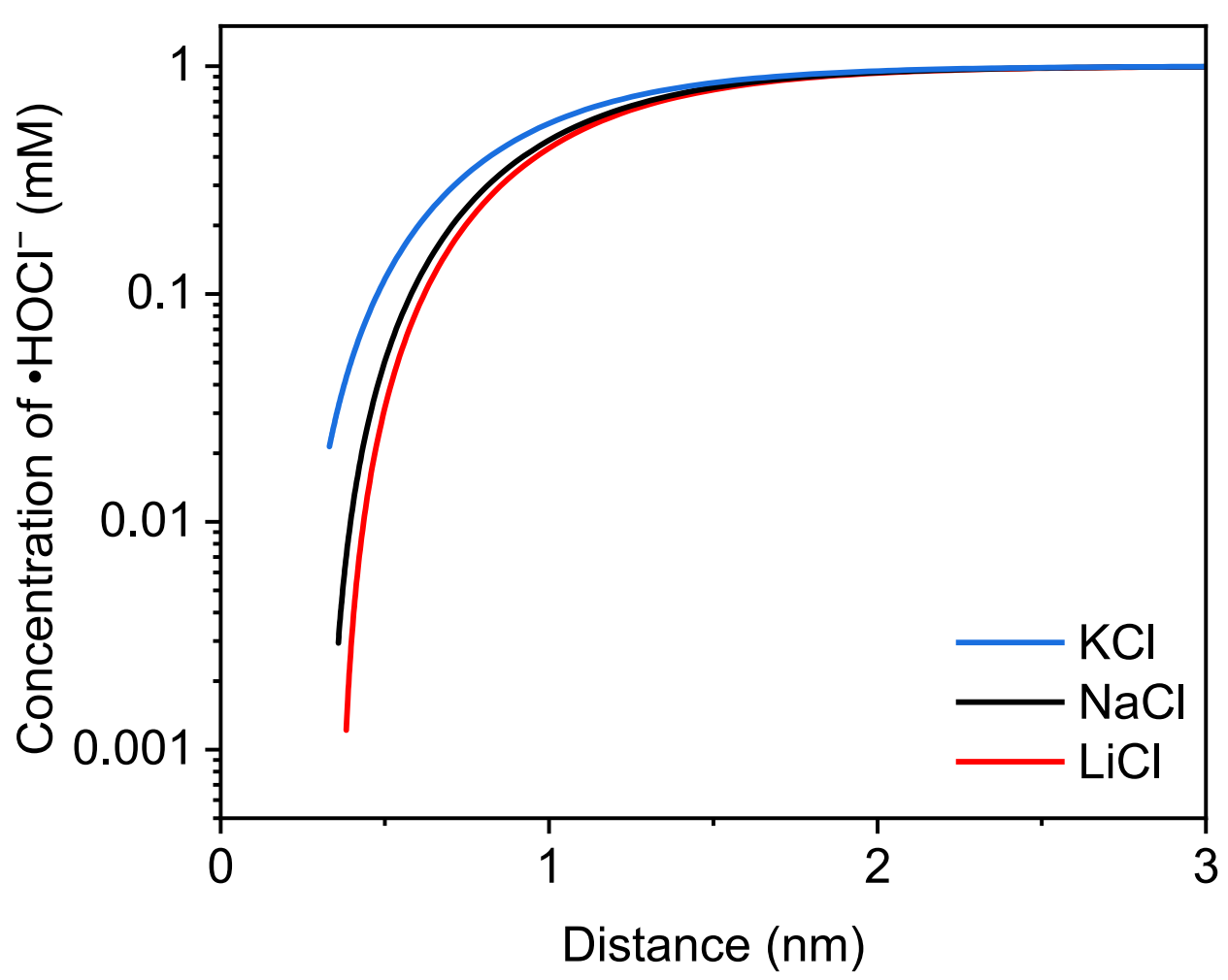

(Page 16) *Supplementary Figure 30. Variation of the • $HOCl^{-}$ concentration with distance in the diffusion layer.*

Reviewer #2 (Remarks to the Author):

In the present manuscript, the authors investigate a contact electrification strategy combined with ultrasonication for active chlorine formation. The system was optimized by varying dielectric materials, powder loading, and salt identity/concentration, ultimately achieving a production rate of 208.8 μmol g^{-1} h^{-1} using 20 mg of FEP powder over 700 h.

Response: We are deeply appreciated to the reviewer's precious suggestions and valuable comments to improve the paper. In response to the reviewer's comments, we have made point-to-point modifications to the manuscript.

My primary concern relates to the claim that this approach is catalyst- and bias-free. While no external electrochemical bias is applied, the system relies on substantial mechanical energy input via ultrasonication (120 W). When considering the absolute production rate (not normalized to FEP mass), the overall active chlorine yield corresponds only to a few nanomoles per hour. From an energy-efficiency and cost-effectiveness standpoint, this performance is never competitive. Given that the authors benchmark their system against the conventional chlor-alkali process, the

present approach does not yet demonstrate a viable alternative for sustainable active chlorine production.

Response: We sincerely thank the reviewer for this insightful comment regarding energy efficiency and sustainability. In response, we quantified the energy consumption of the system under optimized conditions: with 20 mg FEP in 10 mL of natural seawater subjected to 120 W ultrasonication for 1 h, the active chlorine yield reaches 208.8 μmol g^{-1} h^{-1}, corresponding to an absolute production rate of ~4.18 μmol h^{-1}. This translates to an energy consumption of ~3.9 × 10^5 kWh kg^{-1} active chlorine. For comparison, this is considerably higher than the industrial chlor-alkali process or representative emerging systems such as $NbClO_x/BiVO_4$ (*Nat. Sustain., 2025, 8, 672-681; Nat. Sustain., 2024, 7, 148-157*). We therefore fully agree that, in its current batch ultrasonication configuration, CE-Chemistry does not yet compete in terms of unit-product energy efficiency.

However, it is important to clarify that the sustainability discussed in our work does not refer to energy efficiency per unit product, but rather to the process itself: ***CE-Chemistry generates active chlorine without producing toxic Cl_2, avoids noble-metal catalysts or external electrical bias, and operates directly in complex media such as natural seawater.*** In this sense, the system represents a fundamental scientific advance, overcoming limitations inherent to conventional chlorination methods.

Moreover, while CE-Chemistry currently requires more energy per unit product than industrial processes, the energy input does not need to come from an external power source. ***The system relies on contact-separation-induced contact electrification, which can, in principle, be driven by low-frequency mechanical energy naturally present in marine environments (e.g., waves, fluid flow, or vibrations)***. In particular, marine wave energy alone has an annual potential exceeding 10,000 TWh, highlighting the great promise of the ocean as a sustainable energy source (*Ocean, 2025, 1, 9470004; Adv. Mater., 2025, 37, e11283*). If realized, this approach could decouple chemical production from conventional high-energy electrical inputs, achieving active chlorine synthesis through ubiquitous ambient energy, an opportunity that conventional high-efficiency methods cannot access.

Such oceanic mechanical energy could, in principle, directly drive CE-Chemistry for green active chlorine production or, via energy-harvesting technologies, be converted to electrical power for downstream processes (*Nano-Micro Lett., 2025, 17, 124; Adv. Mater., 2025, 37, e11283*). Therefore, the system's most promising applications are not in centralized chlorine manufacture, but in distributed scenarios inaccessible to traditional chlor-alkali methods, e.g., ship-surface coatings, marine equipment interfaces, coastal or small-scale water-treatment units, and emergency disinfection, where ambient waves or fluid motion

continuously trigger CE-Chemistry reactions for localized, on-demand antibacterial and anticorrosion functions. Accordingly, the sustainability of this work is better reflected in its novel reaction pathway and application mode, rather than in unit-product energy consumption at this stage.

Accordingly, we have revised the manuscript to moderate claims regarding sustainability. The work is now presented as a proof-of-concept interfacial chemistry platform that introduces a Cl_2-free reaction pathway and highlights a fundamentally new mode of energy-chemistry coupling, rather than as an immediate energy-efficient alternative to established industrial processes. We sincerely appreciate the reviewer's suggestion, which has strengthened both the scientific clarity and positioning of the manuscript. The corresponding discussions have been revised accordingly.

The corresponding discussions had been moderated and revised in the revised Manuscript. Detailed revisions are as below. Thank you most sincerely!

In the revised Manuscript:

(Page 2) *Here, we reframe CE as a radical-generating mechanism at solid-liquid interfaces and establish contact-electro-chemistry (CE-Chemistry) as a sustainable paradigm, capable of harnessing abundant ambient*

mechanical energy to drive chemical transformations without the need for metal catalysts.

(Page 2) *Utilizing a triboelectric radical generation route from natural seawater, this work demonstrates a metal-catalyst-free and sustainable strategy for active chlorine production, achieving a yield of 208.8 μmol g^{-1} h^{-1} with only 20 mg of FEP powder.*

(Page 2) *This work introduces a green and Cl_2-free route for disinfectant production directly from brine and positions CE as a general trigger for radical chemistry, opening avenues for heterogeneous catalysis and CE-driven reaction design.*

(Page 4) *At present, industrial-scale production of active chlorine predominantly relies on the chlor-alkali process, in which chlorine gas (Cl_2) is generated as a corrosive and toxic intermediate[55,56]. Although this process is industrially mature, it faces inherent safety concerns and limitations for distributed applications: the transportation and storage of Cl_2 are subject to strict requirements and may pose serious environmental and health risks[57]. A promising pathway lies in green, metal-free synthetic strategies that bypass Cl_2 generation altogether and may enable distributed active chlorine production by harnessing high-entropy*

mechanical energy from the environment. By directly producing active chlorine from abundant natural seawater, this strategy circumvents the dependence on high-purity water and the hazards of Cl_2, while enabling green and sustainable distributed production[58,59].

(Page 5) *This work delivers a safe, sustainable, and environmentally benign route for distributed active chlorine production directly from seawater, circumventing the limits of conventional electrochemical windows, while also revealing the central role of interfacial triboelectric fields in CE-Chemistry and demonstrating how field strength and reaction pathways can be precisely tuned through regulation of the EDL.*

(Page 15) *Such a route may be particularly relevant for niche scenarios that are difficult to address by centralized chlor-alkali infrastructure, such as marine equipment interfaces, ship coatings, localized antibiofouling/anticorrosion systems, and small-scale or emergency disinfection units.*

(Page 15) *This work provides a metal-free, CE-driven proof-of-concept for interfacial chemical transformations, with the potential to enable distributed reactions in the future by harnessing high-entropy mechanical energy from the environment.*

Beyond the practical limitations, I have concerns regarding the fundamental interpretation. Much of the discussion on electric field strength and interfacial effects relies on computational simulations. As the authors noted, on metal surfaces, electric field strength scales with solvated ion size of alkali cations or effective positive charge. However, for the FEP surface examined in this study, no direct experimental evidence (such as measurement of the Stark tuning rate) was provided to validate the proposed interfacial electric field under realistic operating conditions. Without such experimental verification, the mechanistic interpretation remains speculative. Incorporating operando or *in situ* spectroscopic evidence to quantify interfacial field strength would significantly strengthen this otherwise scientifically fragile aspect of the manuscript.

Response: We sincerely thank the reviewer for this constructive and insightful comment. We fully agree that inferring the interfacial electric field solely from simulations, without direct experimental validation under realistic reaction conditions, limits the mechanistic interpretation. To address this, we established a dedicated confocal Raman-NaSCN probe platform, inspired by previous gas-liquid interfacial electric field measurements (*Sci. Adv., 2025, 11, eadx5714; Acc. Chem. Res., 2026, 59, 285-297*). Specifically, 500 ppm FEP powder was dispersed in 2 M NaSCN and subjected to water-bath ultrasonication in a quartz reactor, enabling

electron transfer at the solid-liquid interface and generating an interfacial triboelectric field (*Nat. Commun., 2020, 11, 399; Chem. Soc. Rev., 2024, 53, 4349-4373; Chem. Rev., 2022, 122, 5209-5232*).

Confocal Raman spectroscopy of the NaSCN probe revealed a Stark shift ($\Delta\nu_{C\text{-}N}$) of 51 cm^{-1} (**Fig. R17**), corresponding to an interfacial electric field of ~1.4×10^{10} V m^{-1} at the solid-liquid interface based on the established Stark effect relationship ($E = \Delta\nu_{C\text{-}N}/0.36$ MV cm^{-1}) (*J. Phys. Chem. Lett.,2020, 11, 7423-7428; J. Am. Chem. Soc., 2010, 132, 12784-12785*; (**Fig. R18, red line**). In the absence of ultrasonication, which triggers contact electrification, no Stark shift was observed (**Fig. R18, blue line**), indicating that the measured field originates from interfacial triboelectric effects. Notably, this experimentally determined field is substantially higher than previously reported values for gas-liquid or liquid-liquid interfaces (*Nature, 2025, 640, 87-93; Acc. Chem. Res., 2026, 59, 285-297; Chem. Soc. Rev., 10.1039/d5cs01066g; Nat. Chem. Biol., 2024, 20, 1420-1433; Chem, 2023, 9, 1594-1609*) and exceeds our prior theoretical estimates, providing direct validation of the interfacial triboelectric field.

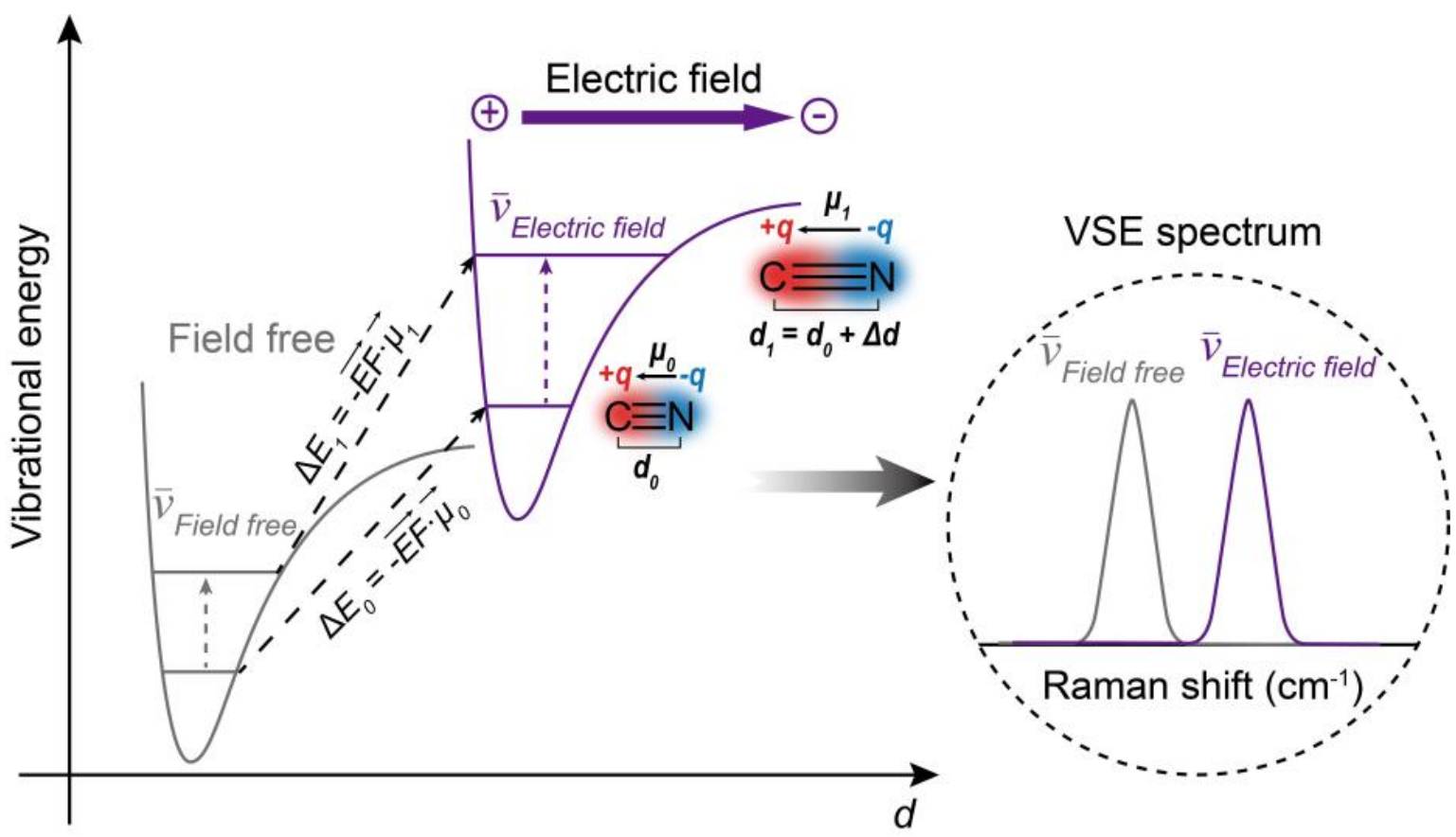

Fig. R17. Illustration of vibrational Stark effect (VSE).

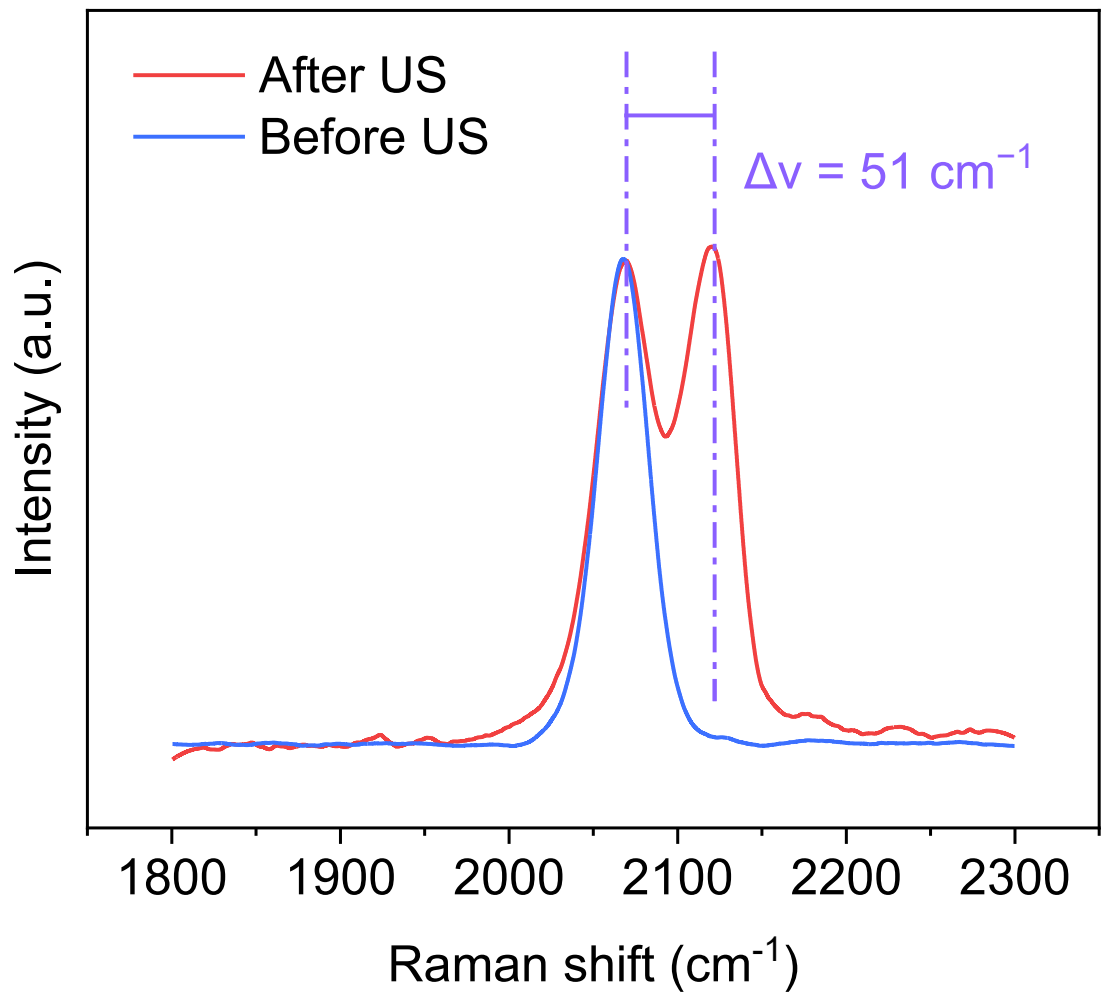

Fig. R18. The dedicated confocal Raman-NaSCN probe platform demonstrates that the triboelectric field at the solid-liquid interface can reach 1.4×10^{10} V m^{-1}.

These results provide direct experimental validation of the triboelectric field, and all theoretical analyses in the manuscript have been recalibrated accordingly (**Fig. R19-R23**). The detailed properties and

mechanistic roles of this field remain a complex topic, which we will continue to explore in future studies. We again sincerely thank the reviewer for this valuable suggestion, which has significantly strengthened the rigor of our work.

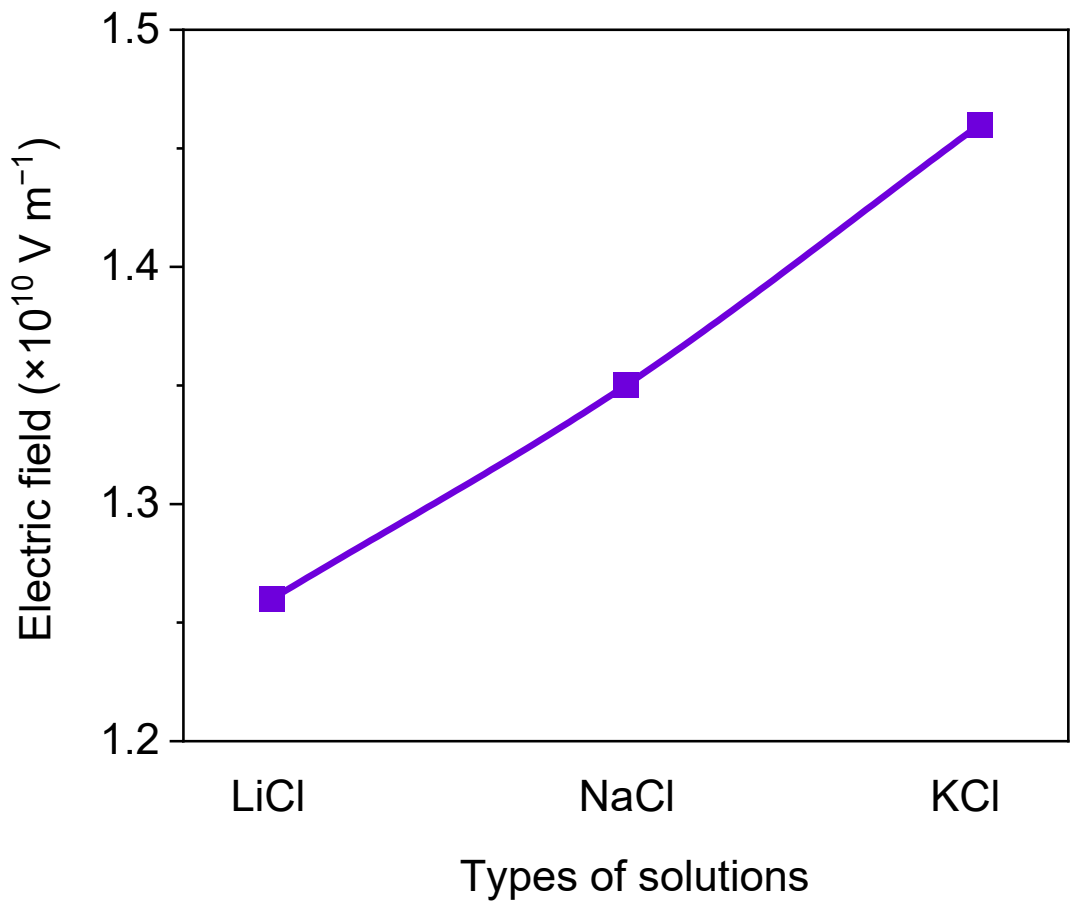

Fig. R19. Comparison of interfacial triboelectric fields within the Stern layer in different salt solutions.

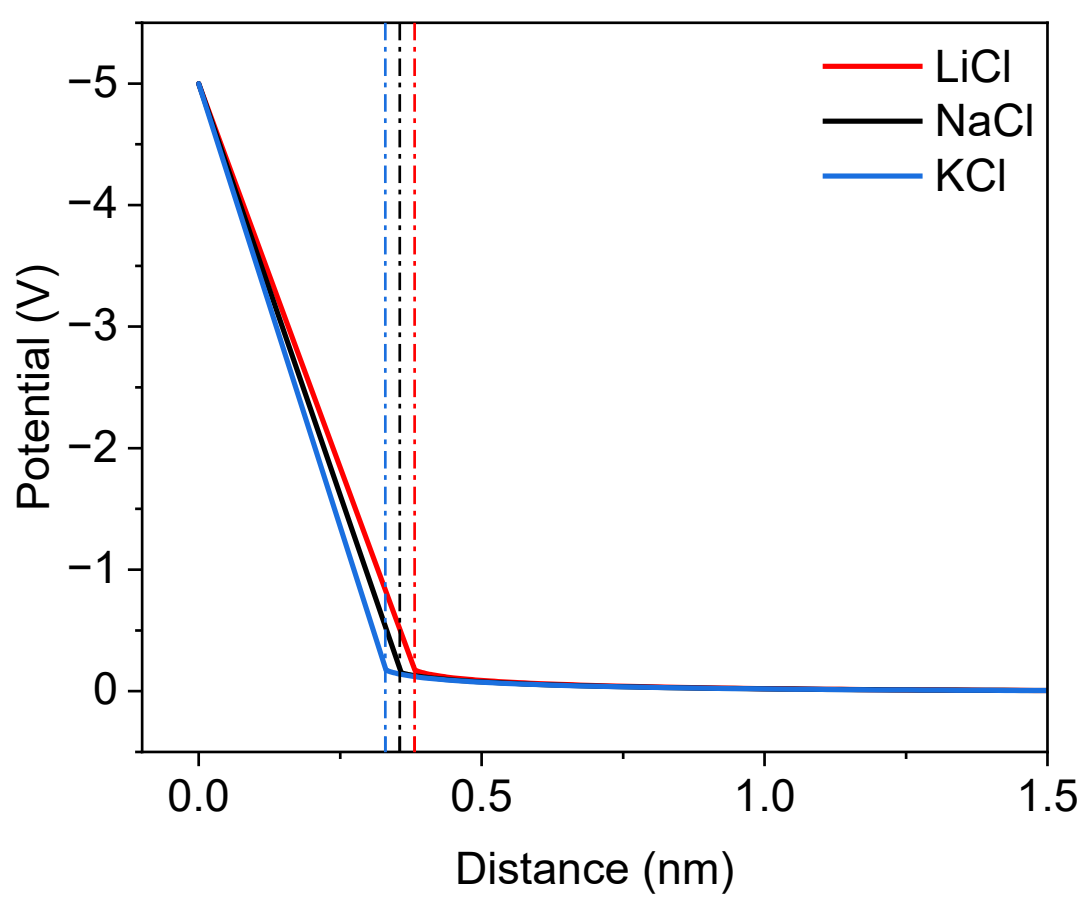

Fig. R20. The variation of the EDL potential with distance in different salt solutions.

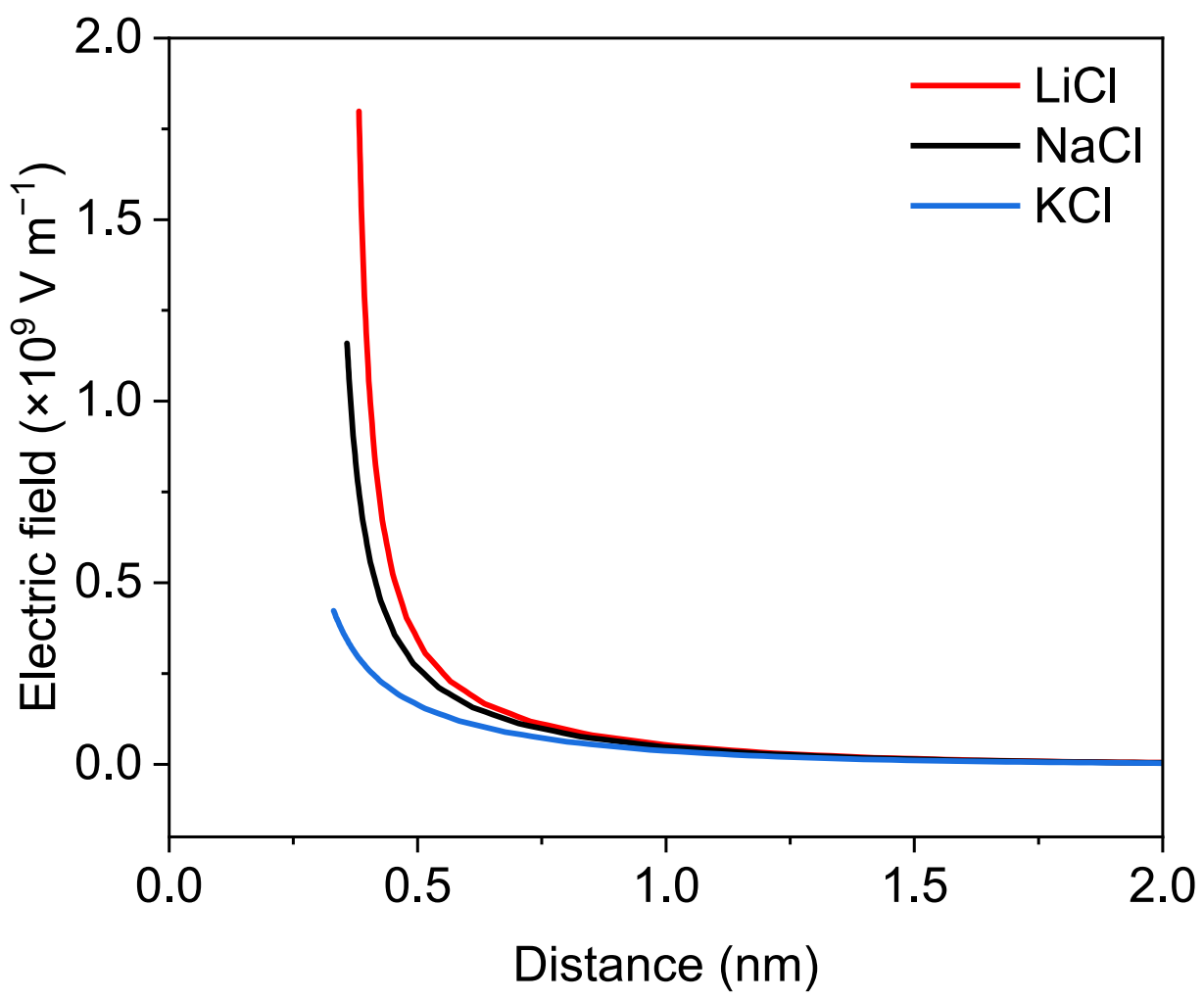

Fig. R21. The distance-dependent interfacial triboelectric field in the diffusion layer of various salt solutions.

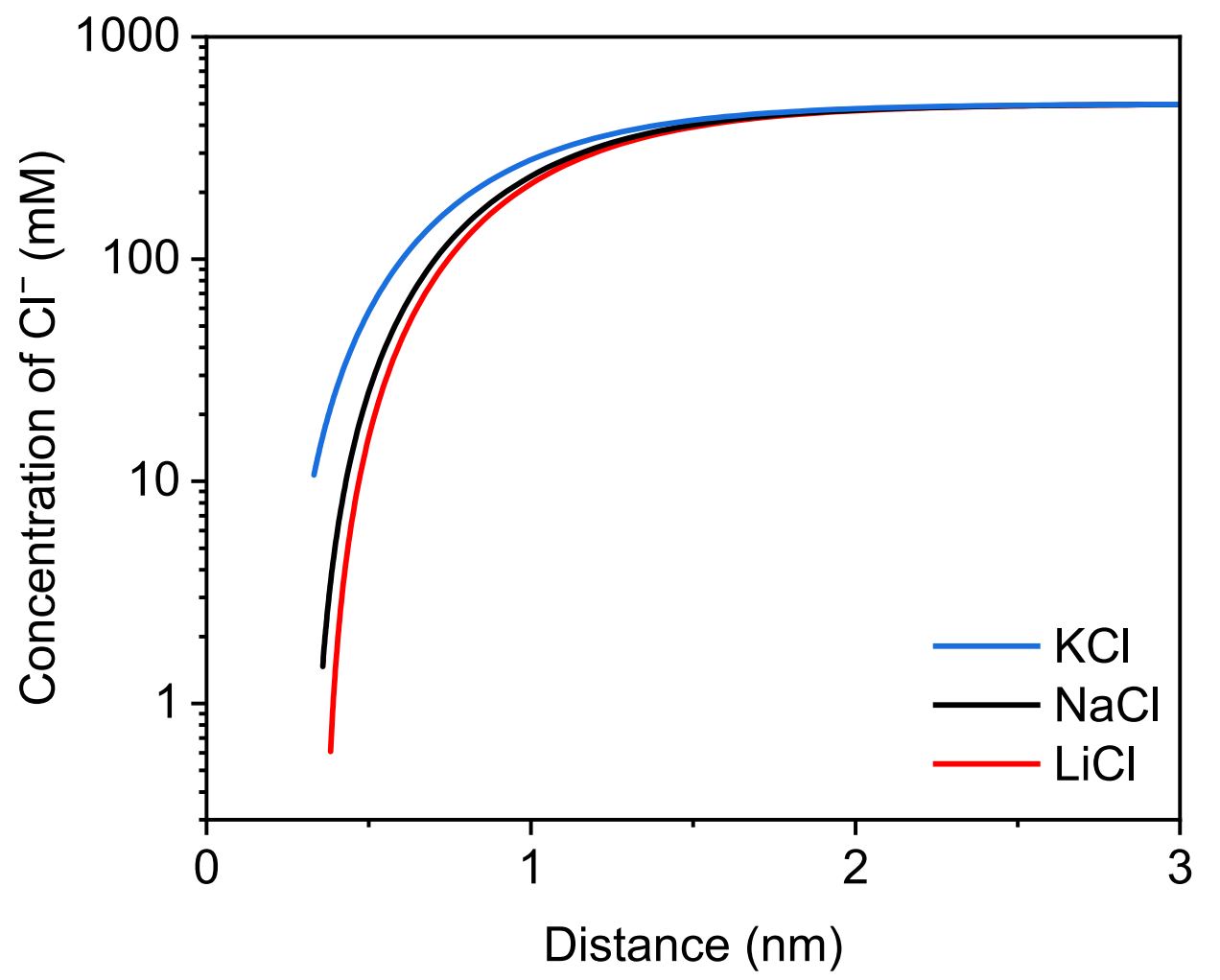

Fig. R22. Variation of the Cl^- concentration with distance in the diffusion layer.

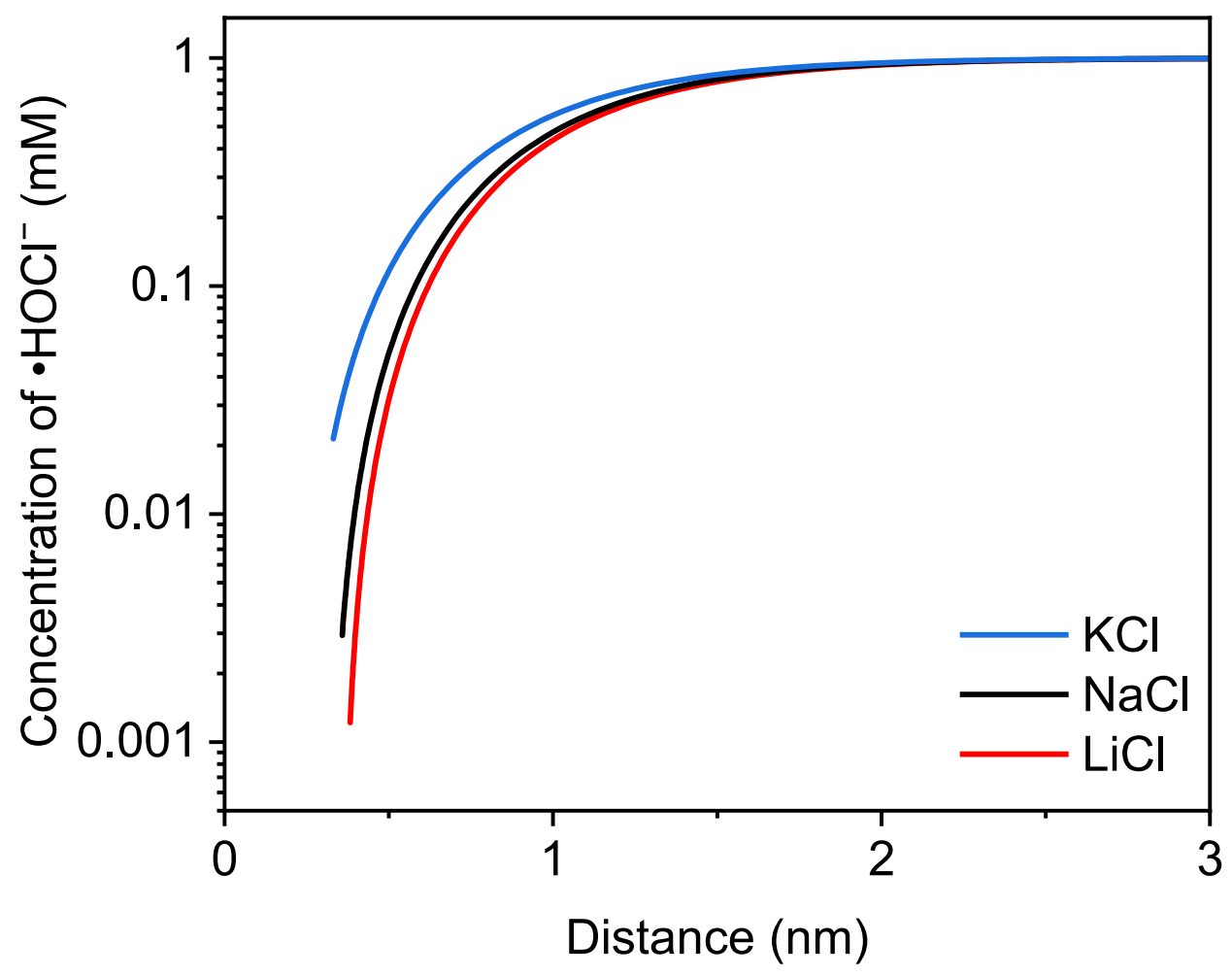

Fig. R23. Variation of the $\bullet HOCl^-$ concentration with distance in the diffusion layer.

The corresponding discussions had been added in the revised Manuscript and revised Supplementary Information. Detailed revision is as below. Thank you most sincerely!

In the revised Manuscript:

(Page 13) *Accordingly, under identical conditions, the EDL in KCl solution is more compact than in NaCl or LiCl solutions (Supplementary Fig. 26), with the interfacial triboelectric field within the Stern layer estimated at 1.46×10^{10} V m^{-1}, exceeding that of NaCl (1.35×10^{10} V m^{-1}) and LiCl (1.26×10^{10} V m^{-1}) (Fig. 4f).*

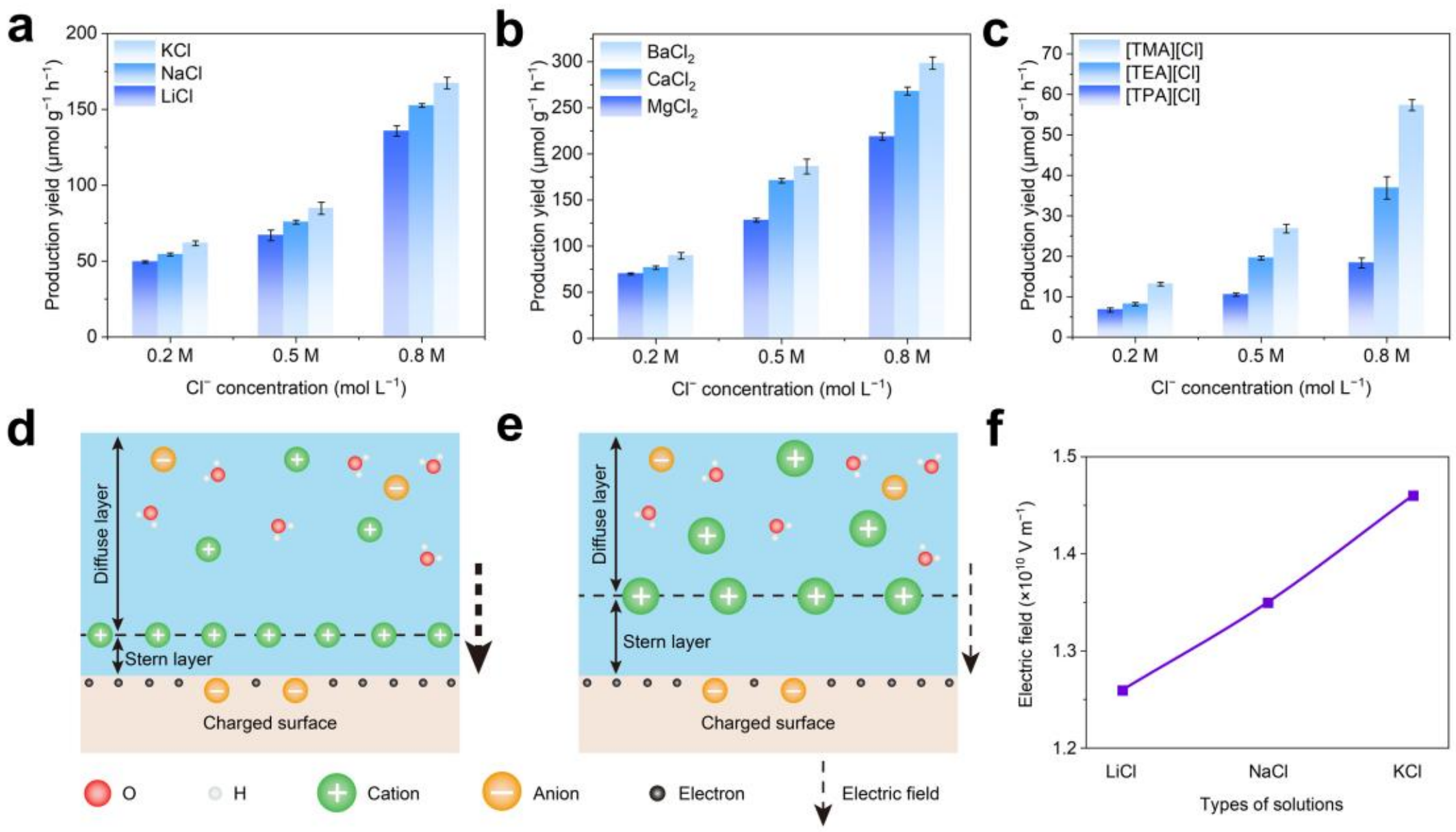

(Page 28) *Fig. 4 Proposed model of the impact of cation effect on CE-Chemistry. f, Comparison of interfacial triboelectric fields within the Stern layer in different salt solutions.*

In the revised Supplementary Information:

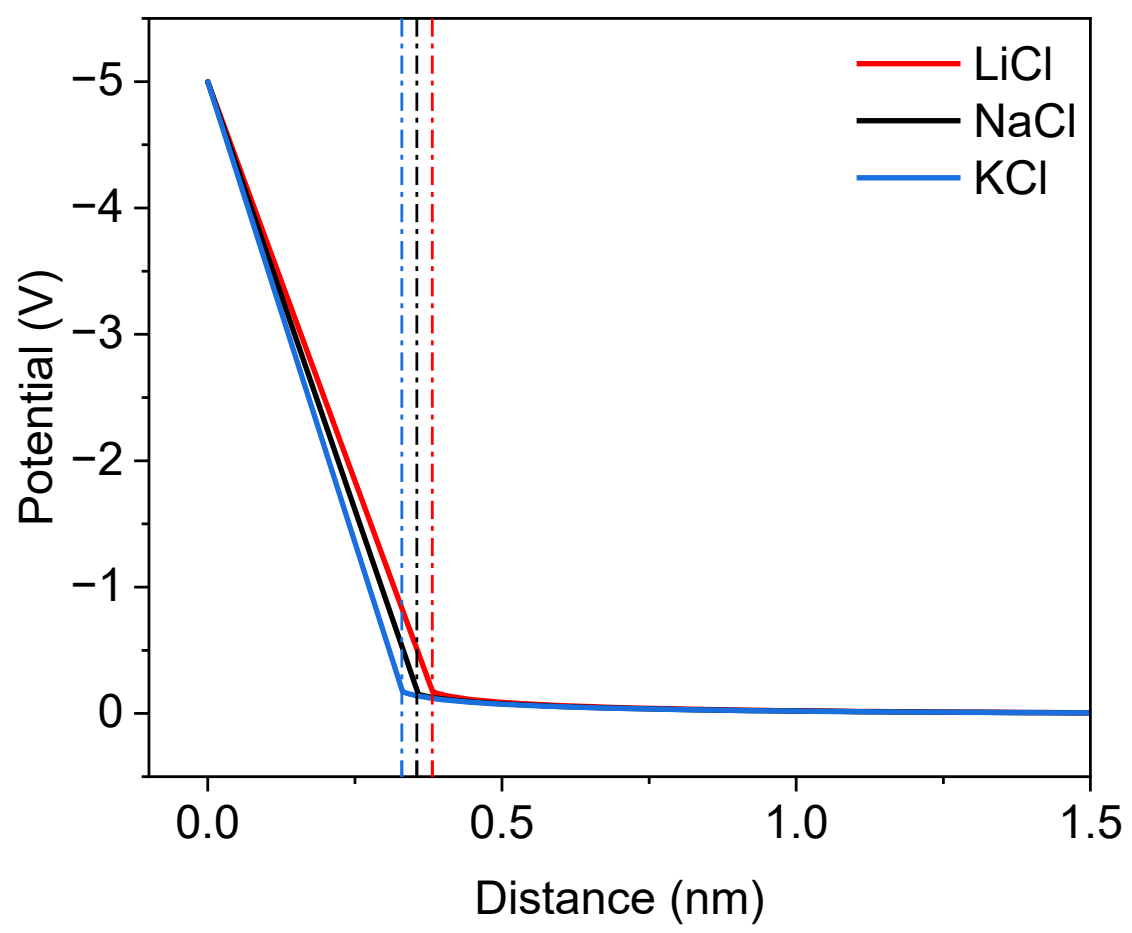

(Page 14) *Supplementary Figure 26. The variation of the EDL potential with distance in different salt solutions.*

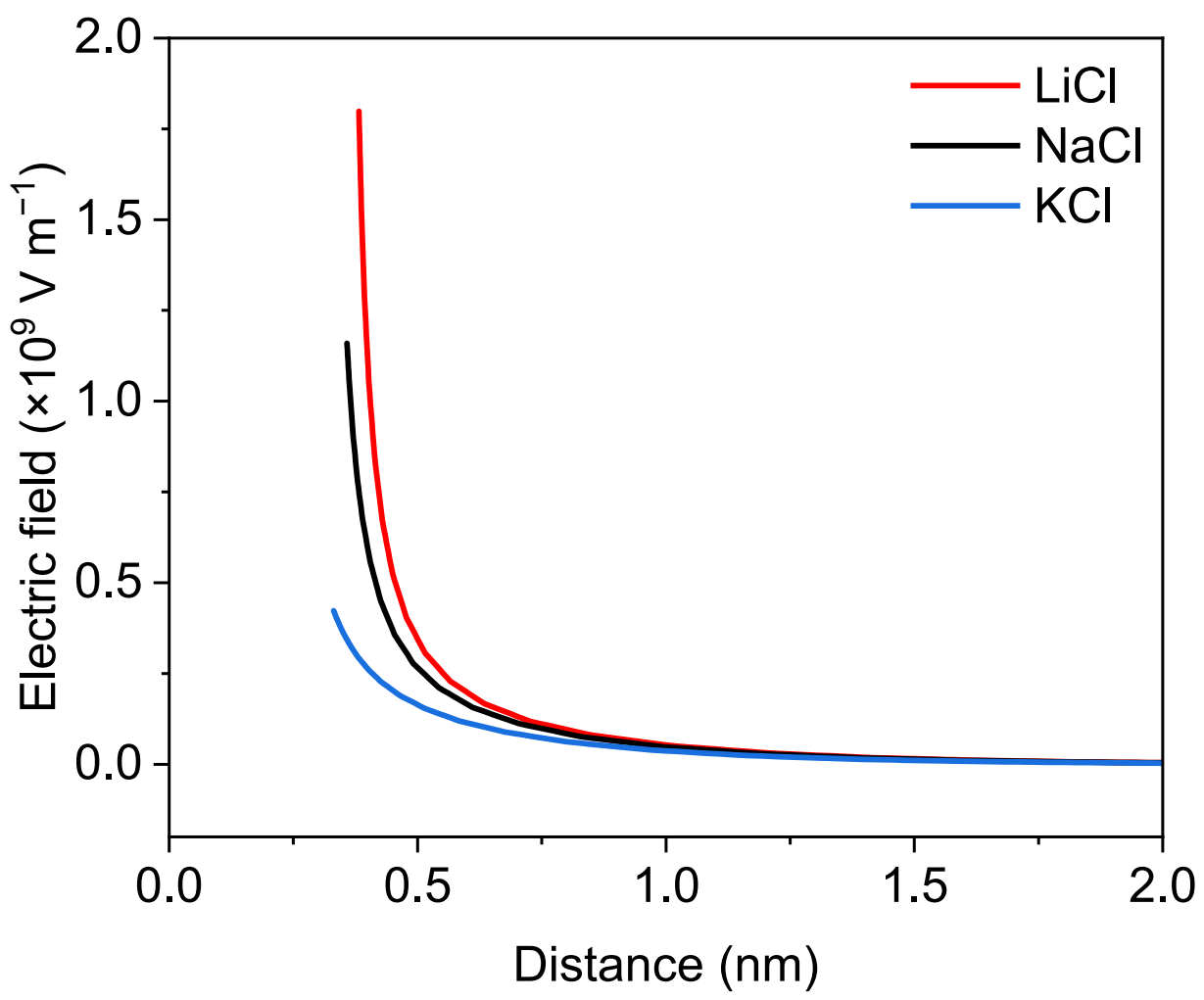

(Page 15) *Supplementary Figure 28. The distance-dependent interfacial triboelectric field in the diffusion layer of various salt solutions.*

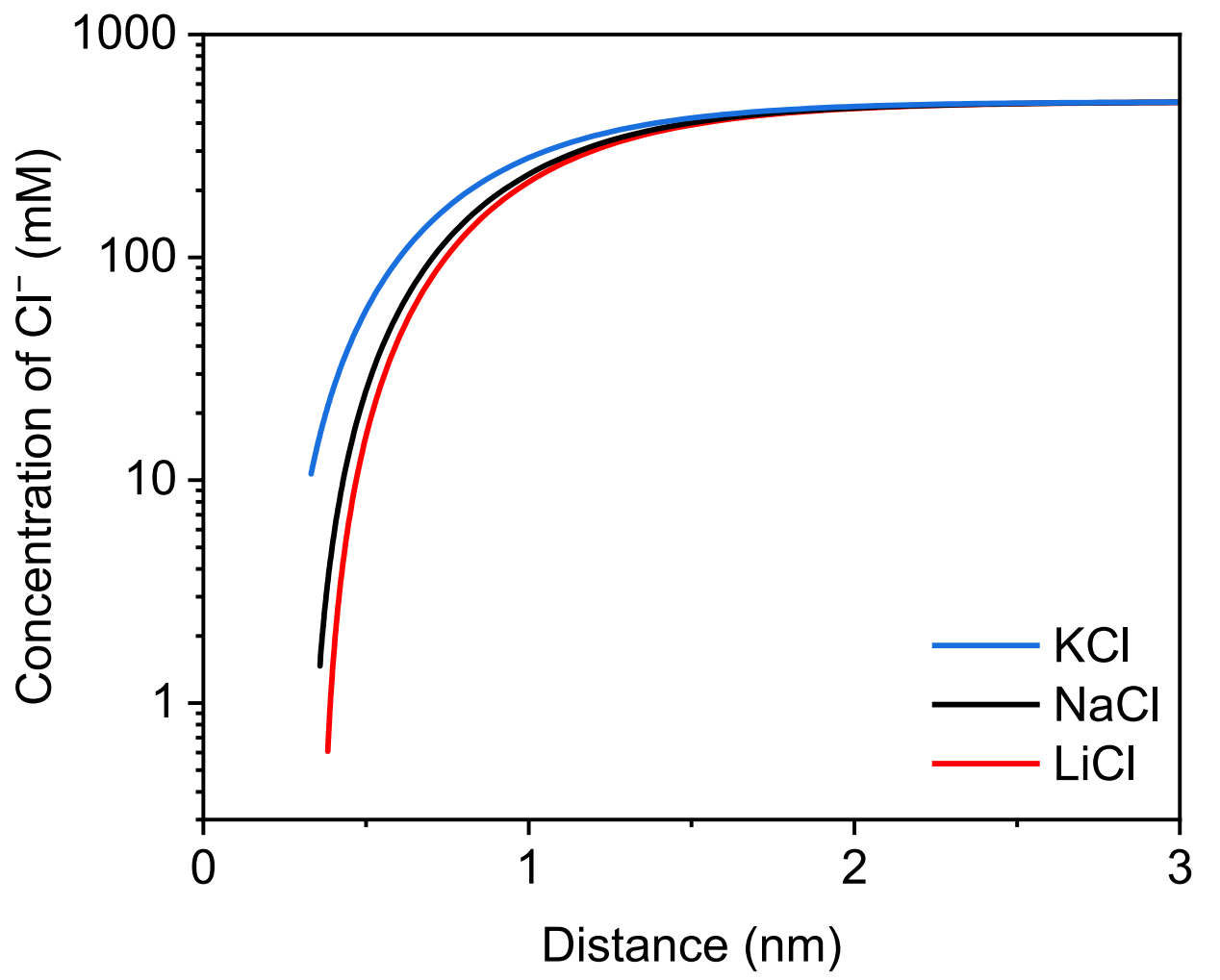

(Page 16) *Supplementary Figure 29. Variation of the Cl^{-} concentration with distance in the diffusion layer.*

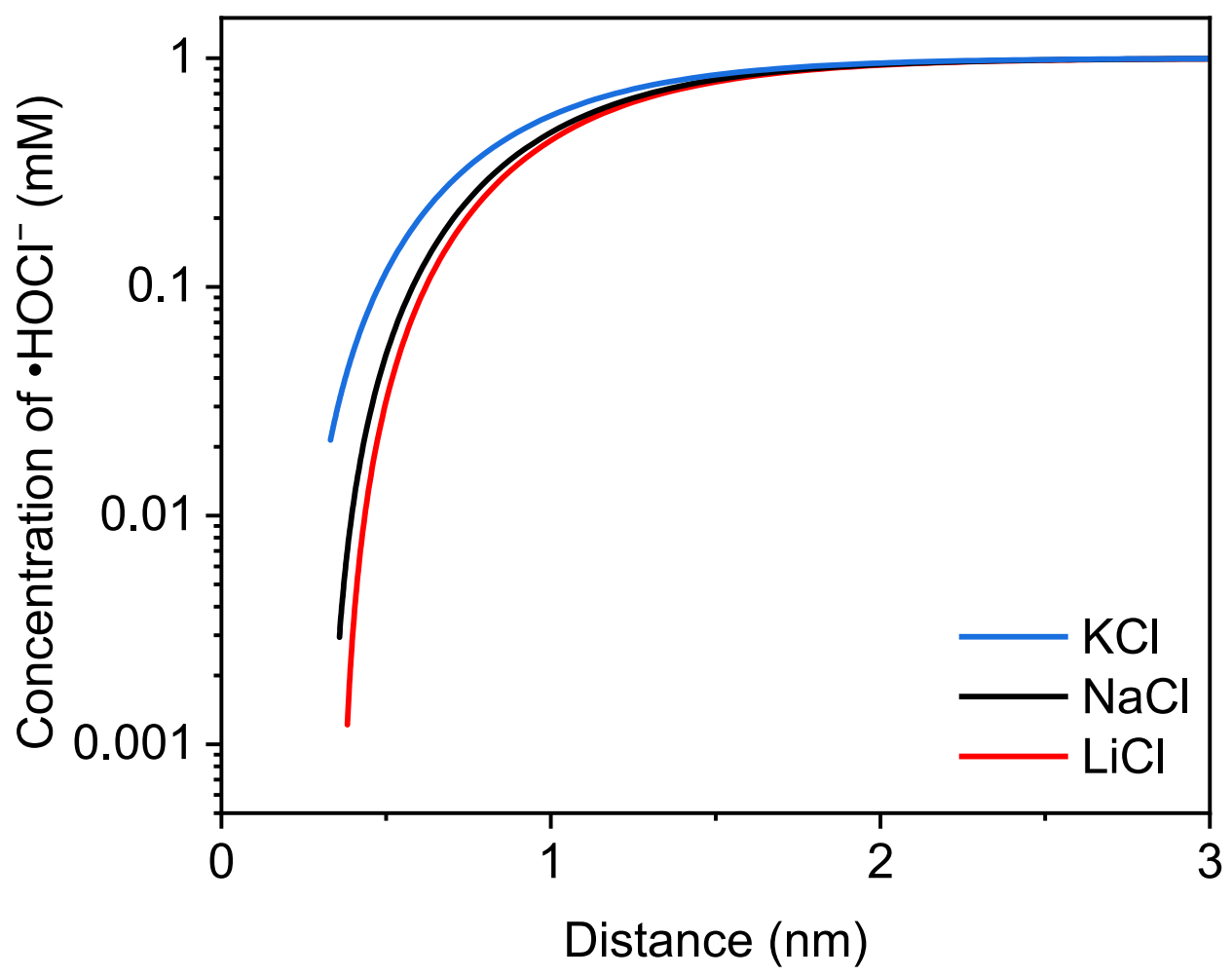

(Page 16) *Supplementary Figure 30. Variation of the • $HOCl^{-}$ concentration with distance in the diffusion layer.*

Overall, the manuscript was well written, but there seem room for improving the quality of the manuscript further for consideration in publication.

Response: We thank the reviewer for the positive assessment of our work, and we have carefully revised our manuscript in response to the valuable comments and suggestions.

Reviewer #3 (Remarks to the Author):

The manuscript entitled "A triboelectric radical generation route to chlorine disinfectants from brine" firstly reported active chlorine prepared by CE-chemistry. Besides, the effect of EDL on CE-chemistry was discussed. However, there are some problems that need to be solved. In particular, the effects of EDL on CE-chemistry and experimental details need to be carefully discussed and supplemented. Considering the overall work, I recommend consideration of the manuscript for publication after a careful revision where the following points are addressed.

Response: We thank the reviewer for the positive assessment of our work, and we have carefully revised our manuscript in response to the valuable comments and suggestions.

1. Why was a 0.5 M NaCl solution chosen? Is there any preliminary experiment to support it?

Response: We sincerely thank the reviewer for this constructive comment. In the previous version, we did not explicitly explain why 0.5 M NaCl was used as a substitute for natural seawater. This choice is based on the fact that NaCl is the predominant component of seawater, and its concentration

in seawater is approximately 0.5 M. Accordingly, previous studies have commonly used 0.5 M NaCl as a simplified model for preliminary experiments (*Nat Rev Mater.,* 2019, 4, 588-605*; Nat Commun.,* 2021 12, 47*; Angew. Chem. Int. Ed., 2020, 25, 8720-8726*). In the original manuscript, we cited the ASTM standard (ASTM D1141-1998) for seawater composition (**Table R1**). To further validate this choice, we have now characterized the ionic composition of the Yellow Sea seawater used in this study via ion chromatography (IC), inductively coupled plasma optical emission spectroscopy (ICP-OES), and titration (**Table R2**). The measured values closely match the ASTM standard, providing complementary support. We have also added the rationale for using 0.5 M NaCl in the revised manuscript.

Table R1. Component and ion concentration of the substitute seawater according to ASTM D 1141-1998.

Component	Amount (g/L)	Ion	Concentration (M)
NaCl	24.53	Na^+	0.447
$MgCl_2$	5.20	Mg^{2+}	0.055
Na_2SO_4	4.09	Ca^{2+}	0.010
$CaCl_2$	1.16	K^+	0.009
KCl	0.695	Cl^-	0.559
-	-	SO_4^{2-}	0.029
Total	35.675	Total	0.610

Table R2. Ion composition and concentrations of natural seawater used in this study.

Ion	Concentration (M)
Na^{+}	0.487
Mg^{2+}	0.040
Ca^{2+}	0.009
K^{+}	0.008
Cl^{-}	0.517
SO_4^{2-}	0.017
HCO_3^{-}	0.003

The corresponding discussions had been added in the revised Manuscript and revised Supplementary Information. Detailed revision is as below.

In the revised Manuscript:

(Page 12) *We also characterized the ionic composition and concentrations of the natural seawater used in this study by ion chromatography (IC), inductively coupled plasma optical emission spectroscopy (ICP-OES), and titration, and found that the Cl^{-} concentration in natural seawater was very close to that in 0.5 M NaCl, while 0.003 M of HCO_3^{-} was also present (Supplementary Table 2).*

(Page 21) *The ionic composition and concentrations of the natural seawater used in the experiments were determined by ion chromatography (IC), inductively coupled plasma optical emission spectroscopy (ICP-OES), and titration.*

In the revised Supplementary Information:

(Page 18) *Supplementary Table 2. Ion composition and concentrations of natural seawater used in this study.*

Ion	***Concentration (M)***
Na^{+}	*0.487*
Mg^{2+}	*0.040*
Ca^{2+}	*0.009*
K^{+}	*0.008*
Cl^{-}	*0.517*
SO_4^{2-}	*0.017*
HCO_3^{-}	*0.003*

2. What is the impact of ultrasonication on EDL? How will changing the ultrasound power affect the CE-Chemistry performance? It is suggested to add relevant discussions.

Response: We sincerely thank the reviewer for the insightful questions.

1. Impact of ultrasonication on the EDL: In CE-Chemistry, ultrasonication acts as a mechanical driving force that continuously generates and collapses cavitation bubbles, enabling repeated contact-separation at the solid-liquid interface. ***This promotes two key processes for EDL formation***: (i) electron transfer, where electrons are transferred from water to the solid dielectric, leading to electron accumulation on the surface and generation of ions (e.g., H^+) and reactive radicals that drive chemical reactions; and (ii) ion migration, where ultrasonically enhanced mass transfer facilitates redistribution of ions toward the charged interface (**Fig. R24**). Additionally, transient high-pressure conditions during cavitation collapse may lower the energy barrier for interfacial electron transfer, further supporting EDL formation (*Mater. Today, 2019, 30, 34; Nat. Commun., 2020, 11, 399; Nat. Commun., 2022, 13, 130; Chem. Rev. 2022, 122, 5209-5232; Chem. Soc. Rev., 2024, 53, 4349-4373*).

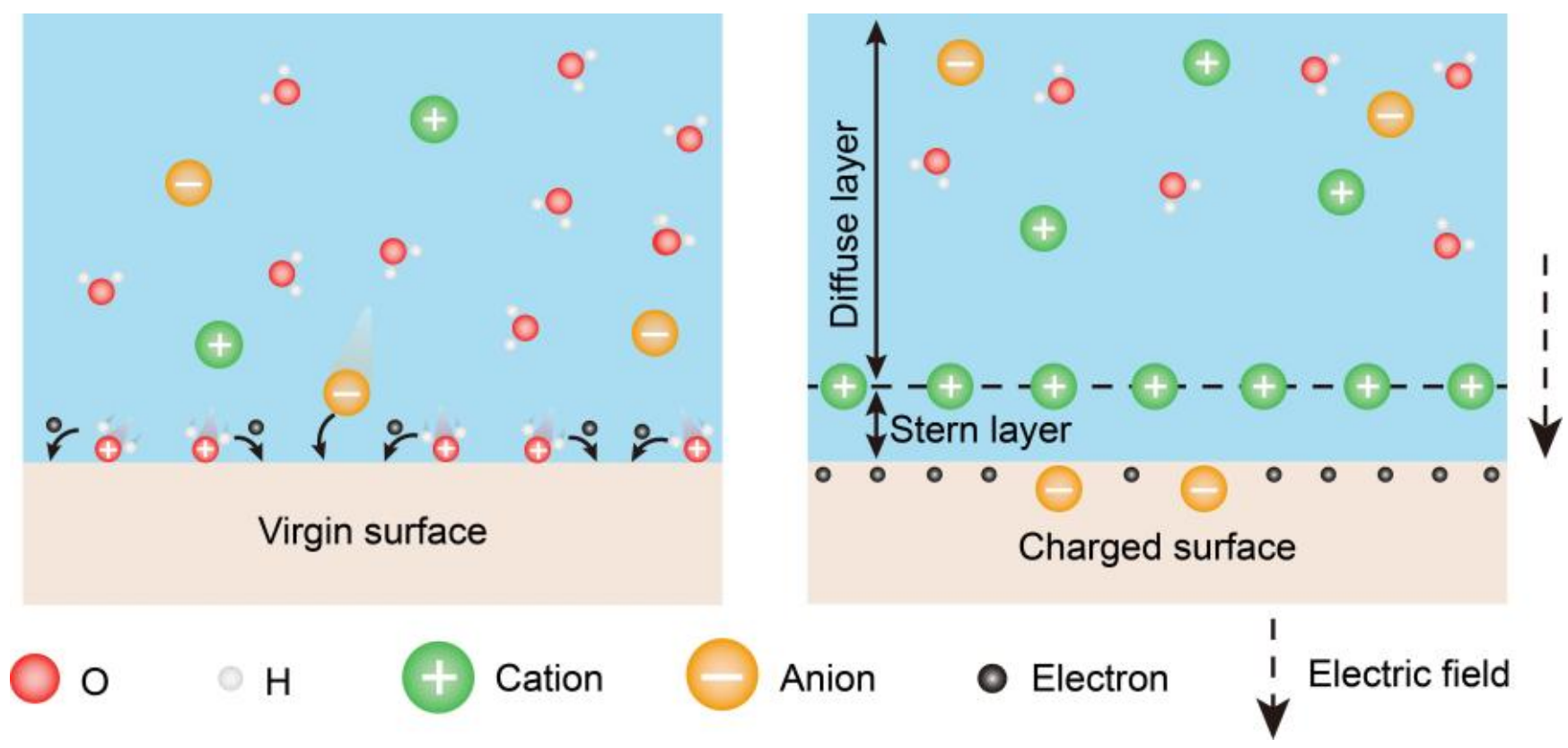

Fig. R24. Formation of the electrical double layer (EDL) and establishment of the interfacial triboelectric field.

2. Effect of ultrasonic power on CE-Chemistry performance: We performed experiments in natural seawater at different ultrasonic powers (60, 120, 200, 300, 400 W). The active chlorine yield increases progressively with ultrasonic power (**Fig. R25**), reflecting more frequent and intense contact-separation events at higher power, which enhance electron transfer, ion migration, and overall reaction kinetics. This observation is also consistent with previous reports (*Nat. Commun., 2024, 15, 757; Nano Energy, 2022, 99, 107346*).

These observations highlight that ultrasonication serves as the direct driving force for EDL formation and CE-Chemistry. Its dual role in facilitating electron accumulation and ion migration underscores the mechanistic importance of interfacial dynamics. The power-dependence data also provide practical guidance for optimizing reaction efficiency, consistent with prior reports (*Nat. Commun., 2024, 15, 757; Nano Energy, 2022, 99, 107346*).

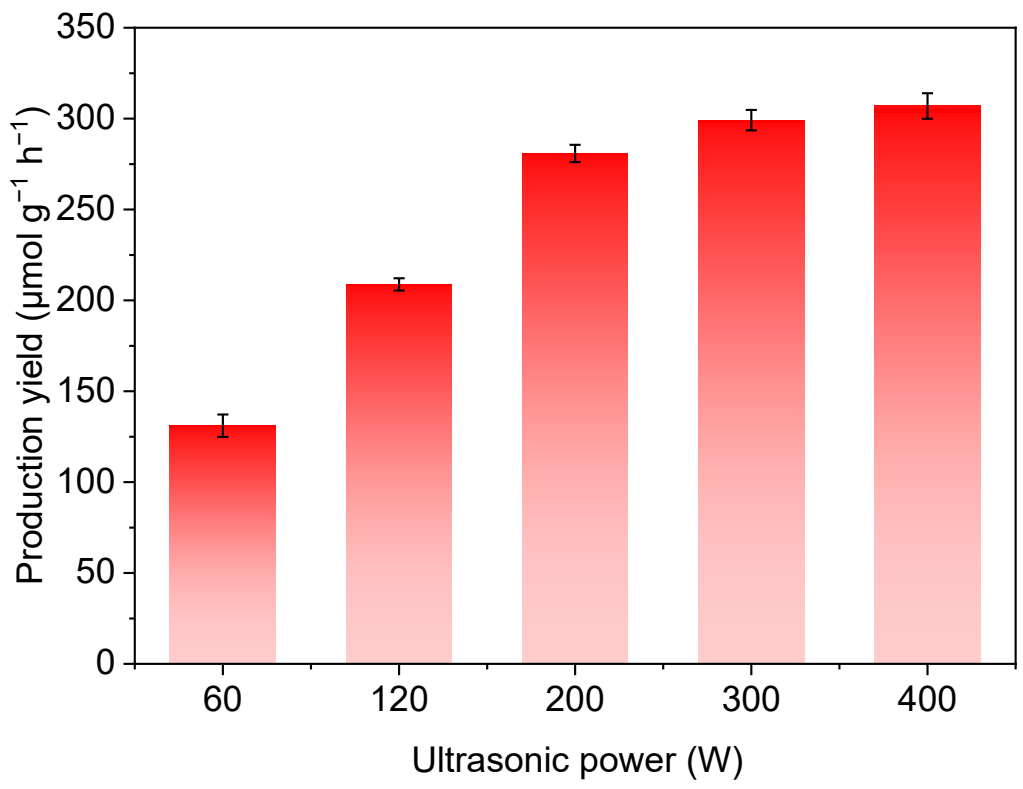

Fig. R25. Comparison of active chlorine production yields at different ultrasonic powers of natural seawater solution.

In the revised Manuscript:

(Page 7) *Moreover, ultrasonic power directly affects the reaction by controlling the frequency of solid-liquid contact-separation events, with higher power promoting more efficient electron transfer and ion migration, thereby enhancing reaction kinetics and increasing product yield[16,68] (Supplementary Fig. 4).*

(Page 20) *The reaction was conducted under optimal conditions of 20 °C, 20 mg FEP, 10 mL of natural seawater, and thermostatic ultrasonication at 40 kHz and 120 W for 1 h. For systematic investigations of the effects of solution volume (10, 25, 50, 75, 100 mL), temperature (5, 10, 20, 30, 40 °C), or ultrasonic power (60, 120, 200, 300, 400 W), all tests were designed based on these optimal conditions, with only a single variable altered each time.*

In the revised Supplementary Information:

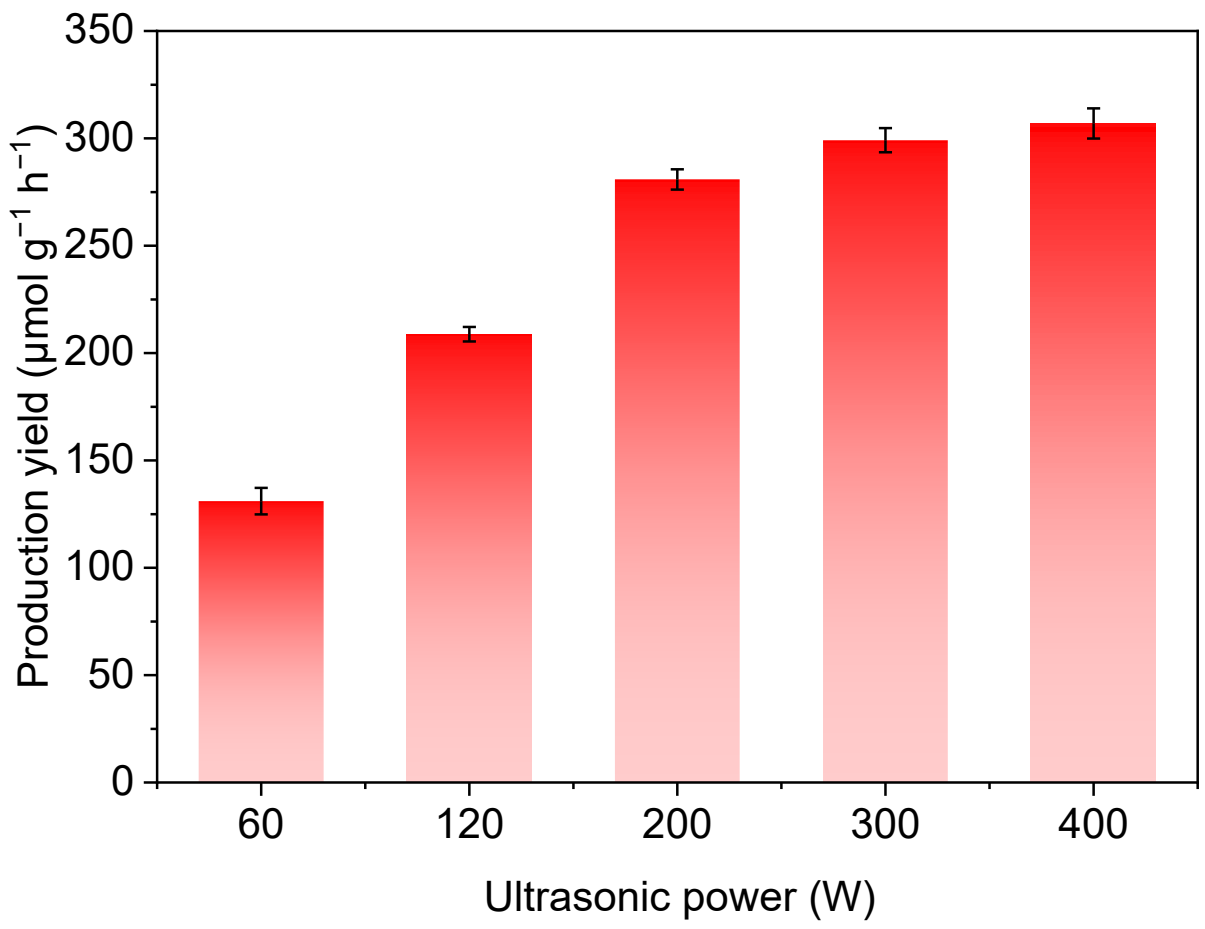

(Page 2) *Supplementary Figure 4. Comparison of active chlorine production yields at different ultrasonic powers of natural seawater solution.*

3. Is there any testing of the composition of seawater and an introduction to its main components? It is suggested to provide additional relevant data.

Response: We are deeply appreciated to the reviewer's precious suggestions and important comments to improve the paper. In the original manuscript, we cited the ASTM standard (ASTM D1141-1998) for seawater composition (**Table R3**). However, considering the regional variation in seawater composition, we have supplemented the ionic composition and concentrations of the seawater collected from the Yellow Sea used in this study by ion chromatography (IC), inductively coupled plasma optical emission spectroscopy (ICP-OES), and titration. The

measured values (**Table R4**) were found to be in very close agreement with the ASTM standard, providing complementary validation.

Table R3. Component and ion concentration of the substitute seawater according to ASTM D 1141-1998.

**Component**	**Amount (g/L)**	**Ion**	**Concentration (M)**
NaCl	24.53	Na^+	0.447
$MgCl_2$	5.20	Mg^{2+}	0.055
Na_2SO_4	4.09	Ca^{2+}	0.010
$CaCl_2$	1.16	K^+	0.009
KCl	0.695	Cl^-	0.559
-	-	SO_4^{2-}	0.029
Total	35.675	Total	0.610

Table R4. Ion composition and concentrations of natural seawater used in this study.

**Ion**	**Concentration (M)**
Na^+	0.487
Mg^{2+}	0.040
Ca^{2+}	0.009
K^+	0.008
Cl^-	0.517
SO_4^{2-}	0.017
HCO_3^-	0.003

The corresponding discussions had been added in the revised Manuscript and revised Supplementary Information. Detailed revision is as below. Thank you most sincerely!

In the revised Manuscript:

(Page 12) *We also characterized the ionic composition and concentrations of the natural seawater used in this study by ion chromatography (IC), inductively coupled plasma optical emission spectroscopy (ICP-OES), and titration, and found that the Cl^- concentration in natural seawater was very close to that in 0.5 M NaCl, while 0.003 M of HCO_3^- was also present (Supplementary Table 2).*

(Page 21) *The ionic composition and concentrations of the natural seawater used in the experiments were determined by ion chromatography (IC), inductively coupled plasma optical emission spectroscopy (ICP-OES), and titration.*

In the revised Supplementary Information:

(Page 18) *Supplementary Table 2. Ion composition and concentrations of natural seawater used in this study.*

Ion	***Concentration (M)***
Na^+	*0.487*
Mg^{2+}	*0.040*
Ca^{2+}	*0.009*
K^+	*0.008*
Cl^-	*0.517*
SO_4^{2-}	*0.017*
HCO_3^-	*0.003*

4. In Fig. 3d and 3e, when there is no O_2 present, where do the electrons of FEP go? Did the electrons cause stronger shielding, thereby causing CE-Chemistry to cease?

Response: We sincerely thank the reviewer for this insightful comment. We fully agree that clarifying the fate of electrons accumulated on dielectric materials such as FEP is essential for a complete understanding of the reaction cycle and electron balance in CE-Chemistry. Unlike conventional electrochemistry, in CE-Chemistry electrons transferred to the dielectric surface are trapped on the FEP surface, where they can either accumulate or react with dissolved species. Specifically, these surface-bound electrons can participate in oxygen reduction with dissolved oxygen

(*Nat. Energy, 2023, 8, 1137-1144; Nat. Commun., 2025, 16, 11035; Nat. Commun., 2022, 13, 130*) or be accepted by protons in solution to generate hydrogen gas (*Chem. Eng. J., 2026, 528, 172290; J. Mater. Chem. A, 2025, 13, 32344-32350*). To directly address the reviewer's concern, we investigated the reaction under an argon atmosphere. The reaction proceeds continuously even without oxygen, indicating that oxygen is not the sole electron acceptor. Under these conditions, excess electrons on the FEP surface participate in the hydrogen evolution reaction, and reaction kinetics remain largely stable under different systems (DI water, 0.5 M NaCl solution, and natural seawater) (**Fig. R26**). This demonstrates that electrons do not accumulate indefinitely to suppress subsequent interfacial reactions; instead, they maintain a dynamic balance through alternative pathways, ensuring continuous system operation. Based on this understanding, we have refined the reaction mechanism in **Fig. 3b** to explicitly incorporate possible electron pathways and balancing routes on the FEP surface (**Fig. R27**), providing a more rigorous and comprehensive mechanistic description.

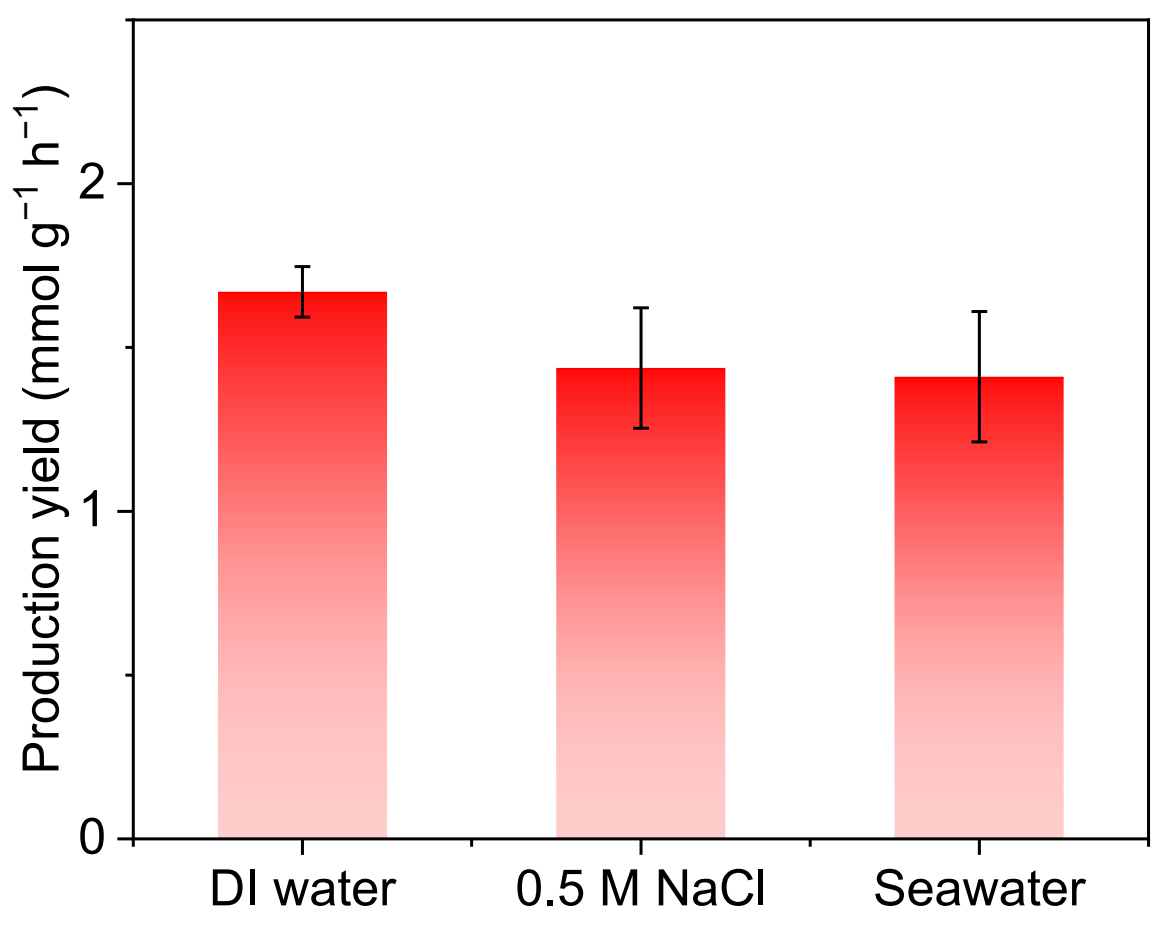

Fig. R26. Comparison of hydrogen production yields in different solutions.

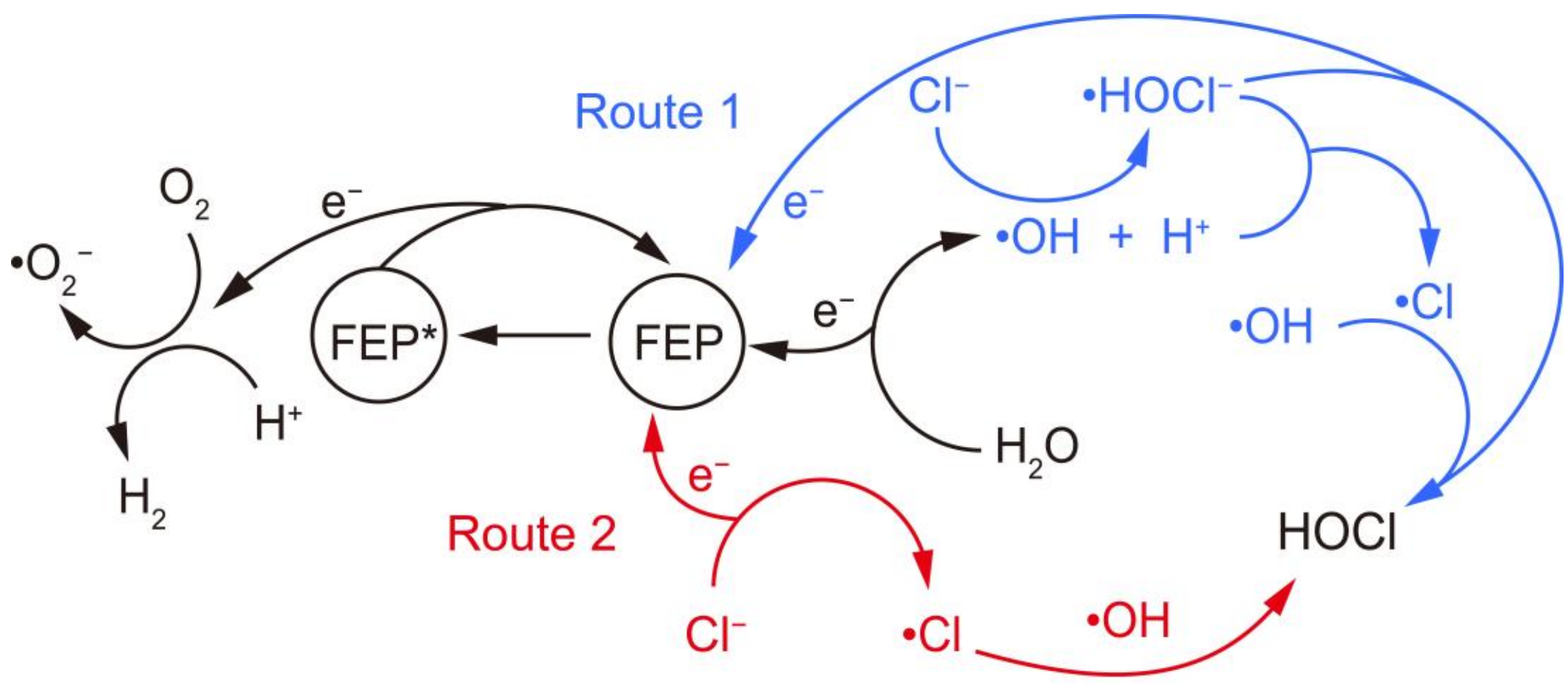

Fig. R27. Proposed mechanism for the generation of active chlorine by CE-Chemistry synthesis (FEP* is proposed to describe the charged state of FEP).

The corresponding discussions had been added in the revised Manuscript and revised Supplementary Information. Detailed revision is as below.

In the revised Manuscript:

(Page 8) *At the same time, oxygen in the cavitation bubbles can receive electrons from the FEP surface to form superoxide anions*[19] *($\bullet O_2^-$, equation (2)), while protons in solution may also accept electrons to generate hydrogen via the hydrogen evolution pathway (H_2, Supplementary Fig. 11 and equation (3)).*

(Page 9)

$$2e^-(FEP) + 2H^+ \rightarrow H_2 + FEP \qquad (3)$$

(Page 20) *Samples for H_2 analysis were prepared in 50 mL of DI water, 0.5 M NaCl solution, or natural seawater containing 20 mg FEP powder. Prior to ultrasonication, the reaction system was purged with argon to establish an oxygen-free atmosphere. The solution was then subjected to ultrasonication, and gas samples were collected from the reactor headspace at regular intervals during the reaction.*

(Page 22) *Hydrogen was detected using a gas chromatograph (GC-2030 Nexis, Japan).*

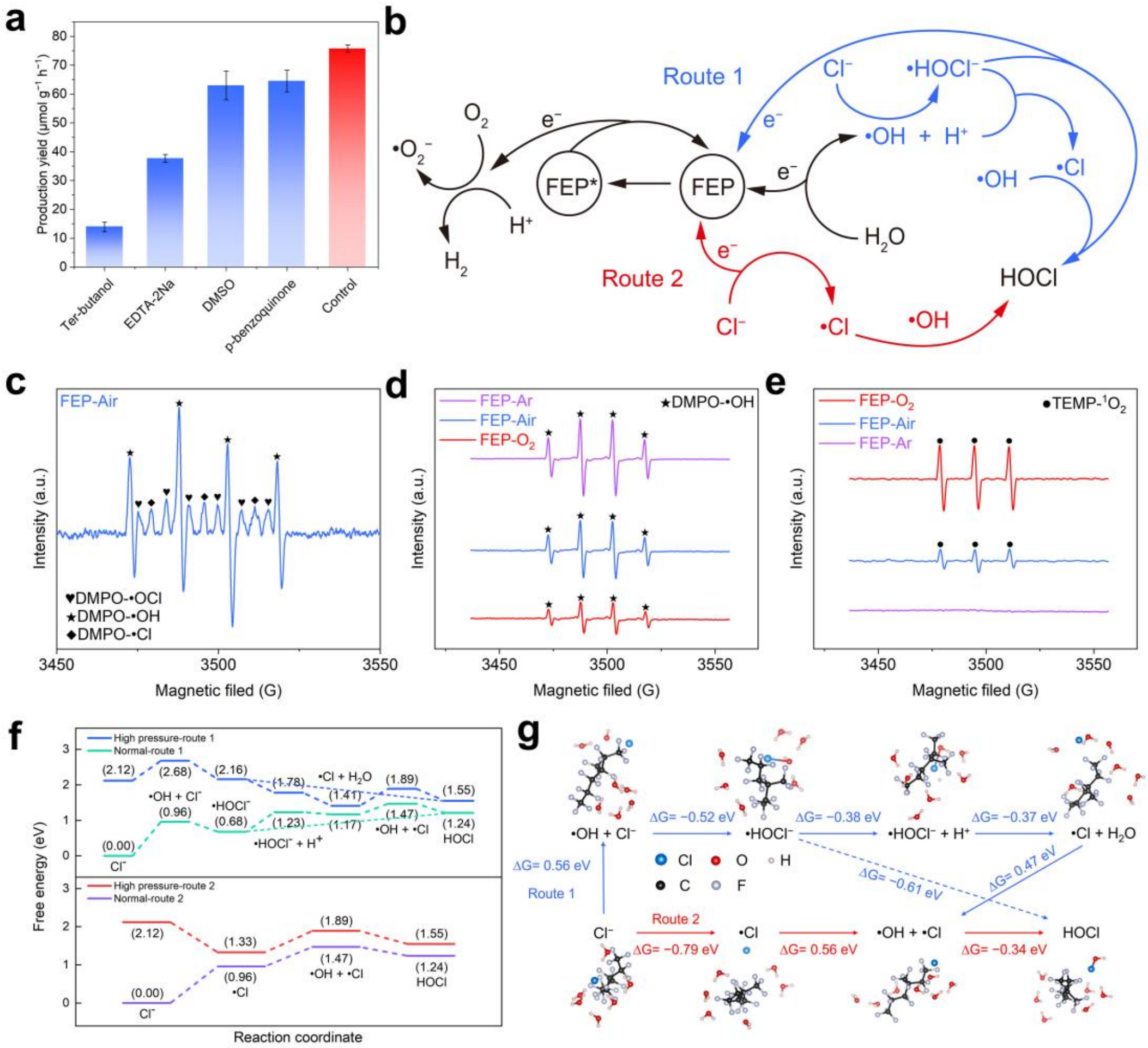

(Page 27) *Fig. 3 Investigation on the contribution of radicals to CE-Chemistry disinfectant synthesis. b, Proposed mechanism for the generation of active chlorine by CE-Chemistry synthesis (FEP* is proposed to describe the charged state of FEP).*

In the revised Supplementary Information:

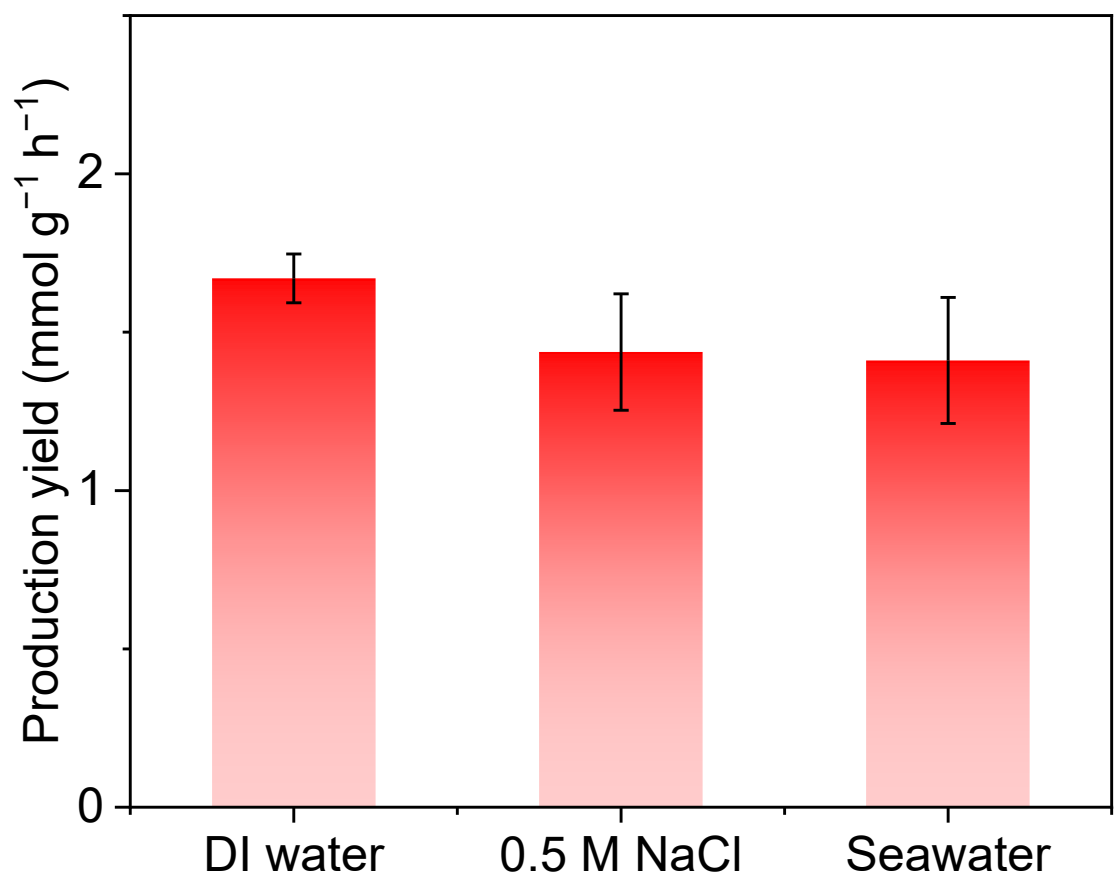

(Page 6) *Supplementary Figure 11. Comparison of H_2 production yields under natural seawater and NaCl solutions.*

5. In the XPS of Supplementary Fig. 6, the binding energy of C 1s is partially missing, and there is no test for the O 1s spectrum compared with the reported references of CEC.

Response: We sincerely thank the reviewer for this valuable and constructive comment. We fully agree that the incomplete labeling of the C 1s binding energies in Supplementary Fig. 6, together with the absence of the O 1s spectrum, may affect the completeness and clarity of this characterization. In response, we have re-measured the relevant samples and incorporated the results and clarifications in the revised manuscript. It is worth noting that XPS probes only a few nanometers in depth over areas of several hundred micrometers, making the spectra highly sensitive to

local surface states. For the nanometer-sized powder samples used here, variations in particle stacking, surface exposure, and local composition can lead to subtle differences in spectral features. The newly obtained C 1s and O 1s spectra are broadly consistent with previous reports in literature (*Nat. Commun., 2022, 13, 130; J. Am. Chem. Soc., 2024, 146, 31574-31584; J. Am. Chem. Soc., 2025, 147, 25407-25416*). To improve completeness and readability, we have now included the full C 1s and O 1s spectra in the revised manuscript (**Fig. R28**) and have supplemented and corrected the corresponding binding energy assignments.

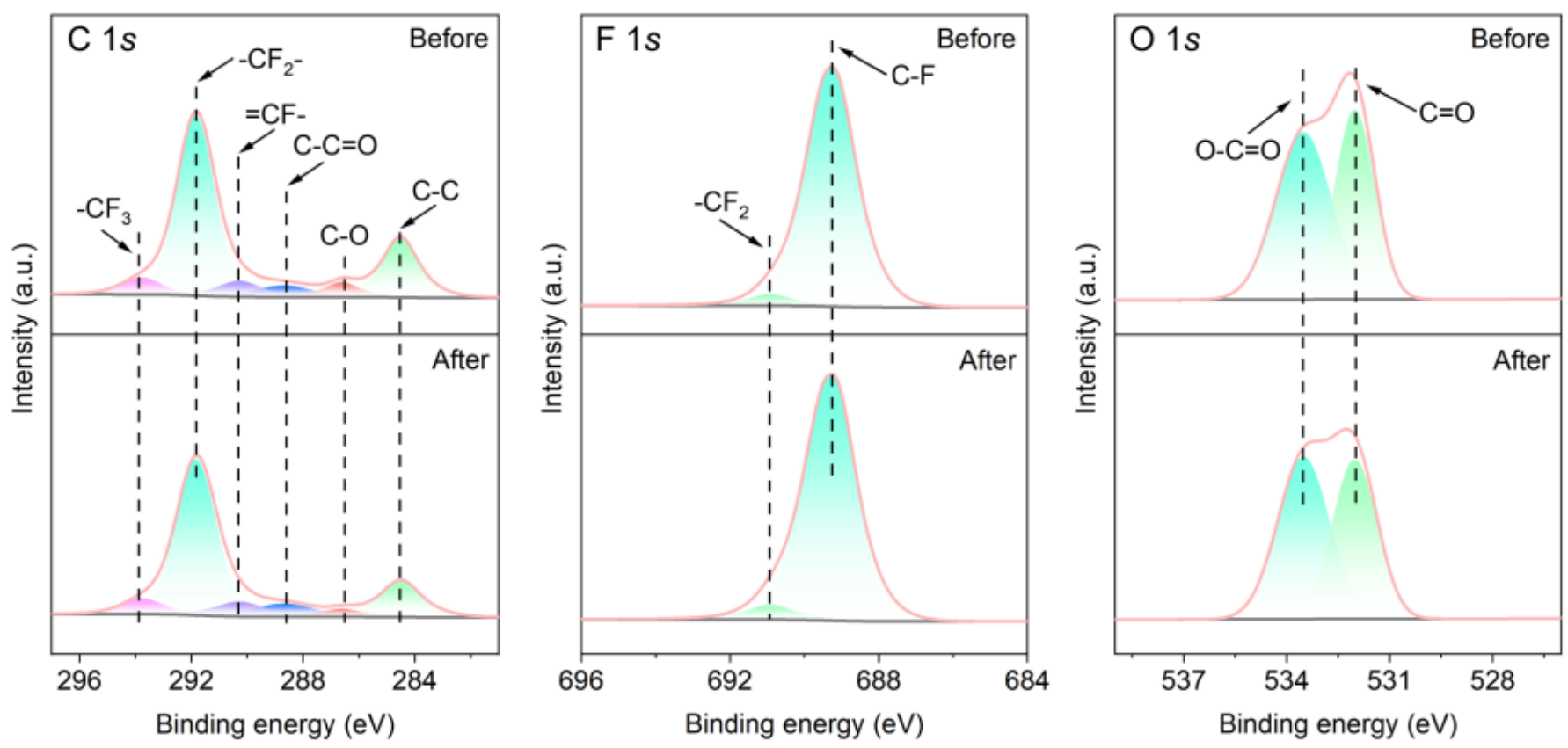

Fig. R28. XPS spectra of FEP powder before and after the reaction.

The corresponding discussions had been added in the revised Supplementary Information. Detailed revision is as below. Thank you most sincerely!

In the revised Supplementary Information:

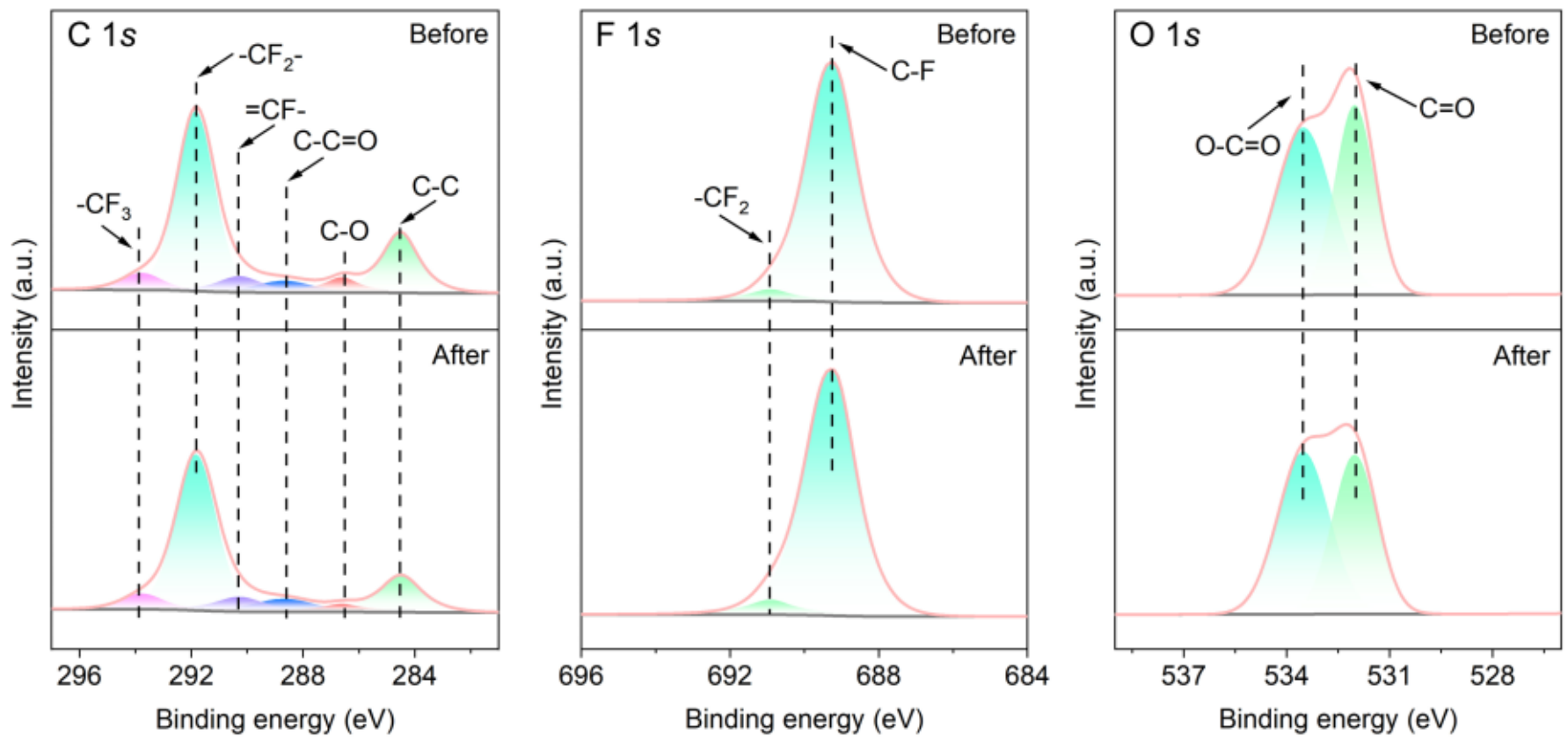

(Page 5) *Supplementary Figure 9. XPS spectra of FEP powder before and after the reaction.*

6. Regarding the mechanism by which pH affects the reaction, it is recommended to conduct a detailed discussion. The reasons why the reaction is promoted under weakly alkaline conditions and inhibited under strongly alkaline conditions, and why no moderate performance was observed between pH = 6 and pH = 8 under neutral conditions.

Response: We sincerely thank the reviewer for this important and insightful comment. We fully agree that a more detailed discussion of the pH effect is warranted. Based on mechanistic analysis, radical evidence, and the pH-dependent experimental results in the original manuscript, we conclude that the role of pH in this system is governed by a combination

of "reactive-species generation kinetics" and the "acid-base-equilibrium-dependent speciation of active chlorine".

The system exhibits the highest active chlorine yield under mildly alkaline conditions (pH=10). Under these conditions, rapid decomposition of HOCl, dominant under acidic conditions, is minimized, while •Cl formation is not significantly suppressed as occurs under strongly alkaline conditions (*J. Am. Water Works Assn., 1948, 40, 1051-1061*; *Nat. Sustain., 2025, 8, 672-681*). Consequently, key reactive species such as •OH and •Cl can act synergistically, optimizing active chlorine generation and accumulation. In contrast, under strongly alkaline conditions, although OCl^- is thermodynamically more stable, both •Cl generation routes are inhibited. The high OH^- concentrations promote electron transfer from OH^- to FEP (**Eq. R1**), suppressing electron transfer pathways critical for •Cl formation *(Environ. Sci. Technol., 2022, 56, 1278-1288*) (**Eq. R2**, **Fig. R29**). Moreover, excessively high pH reduces the oxidizing ability of •OH (*Chem. Rev., 2015, 115, 13051-13092; Environ. Sci. Technol., 2017, 51, 6918-6926; J. Phys. Chem., 1984, 88, 99-101; Environ. Sci. Technol., 2022, 56, 1278-1288*), further suppressing •Cl formation in Route 1 (**Fig. R29**). Together, these effects explain the decreased active chlorine yield under strongly alkaline conditions.

$$\text{FEP} + OH^- \rightarrow \bullet OH + e^-(\text{FEP}) \qquad \textbf{(Eq. R1)}$$

$$Cl^- + \text{FEP} \rightarrow \bullet Cl + e^-(\text{FEP}) \qquad \textbf{(Eq. R2)}$$

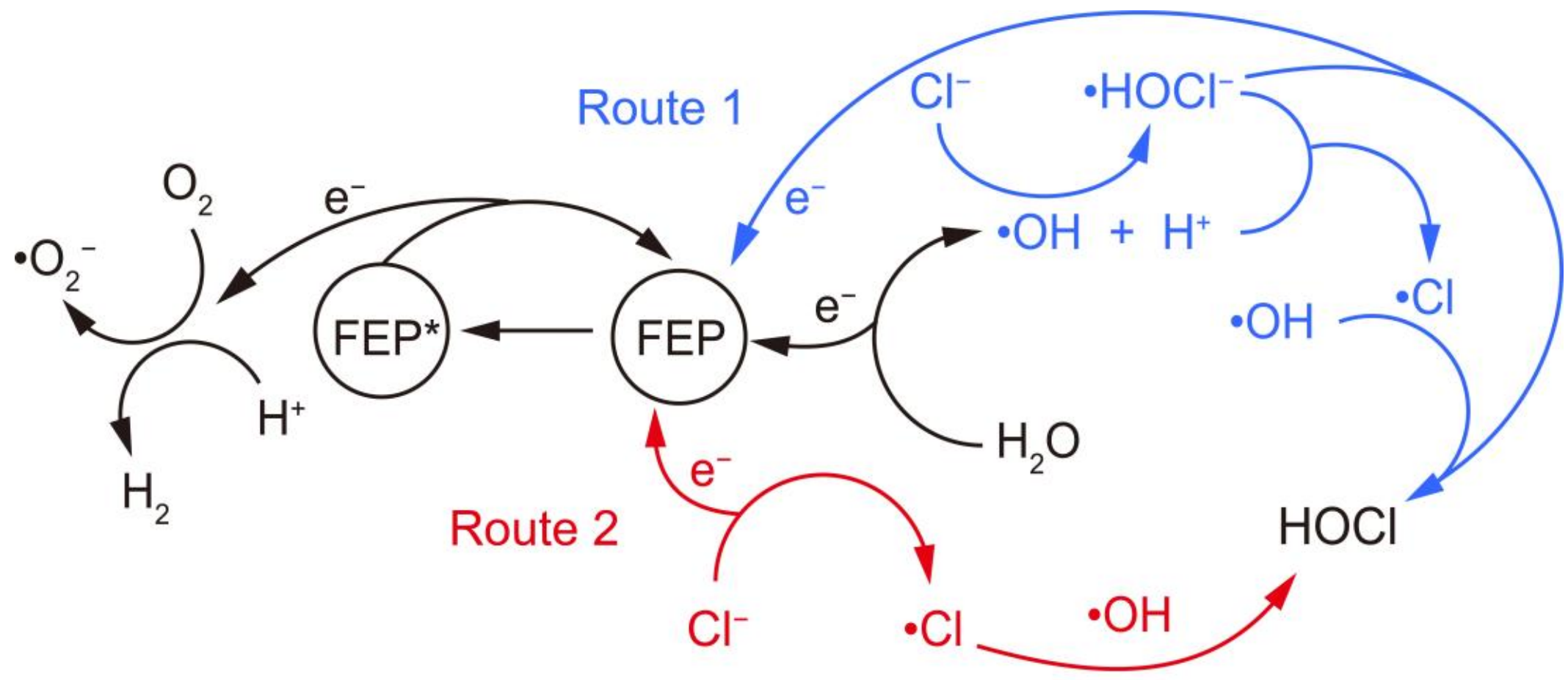

Fig. R29. Proposed mechanism for the generation of active chlorine by CE-Chemistry synthesis (FEP* is proposed to describe the charged state of FEP).

Regarding the reviewer's question on why no clear moderate performance is observed in the neutral pH range (pH 6-8), we fully agree that this point warrants clarification. As noted in the original manuscript, the HOCl/OCl^- equilibrium is highly pH-sensitive: HOCl dominates at pH ≤ 6, while OCl^- prevails at pH ≥ 8 (*J. Am. Water Works Assn., 1948, 40, 1051-1061; Nat. Sustain., 2025, 8, 672-681*). **Supplementary Fig. 21** (**Fig. R30**) shows that the highest yield occurs in the mildly alkaline region, consistent with this mechanism. The neutral range does not represent a simple intermediate plateau, but rather a sensitive window where HOCl and OCl^- interconvert rapidly. Consequently, the system yield is determined by coupled effects of •Cl generation, •OH participation,

HOCl/OCl⁻ interconversion, and product stability, leading to pronounced nonlinear behavior rather than a smooth transition. Within pH 6-8, the system has partially exited the HOCl dominated acidic state but has not yet reached the favorable mildly alkaline window for stable active chlorine accumulation, explaining the absence of a "moderate plateau". Similar nonlinear pH-dependent trends are observed in photocatalytic and electrocatalytic active chlorine synthesis (*Nat. Sustain., 2025, 8, 672-681; Nat. Sustain., 2024, 7, 148-157*). Following the reviewer's suggestion, we have expanded the discussion in the revised manuscript to make this mechanistic interpretation more complete and rigorous.

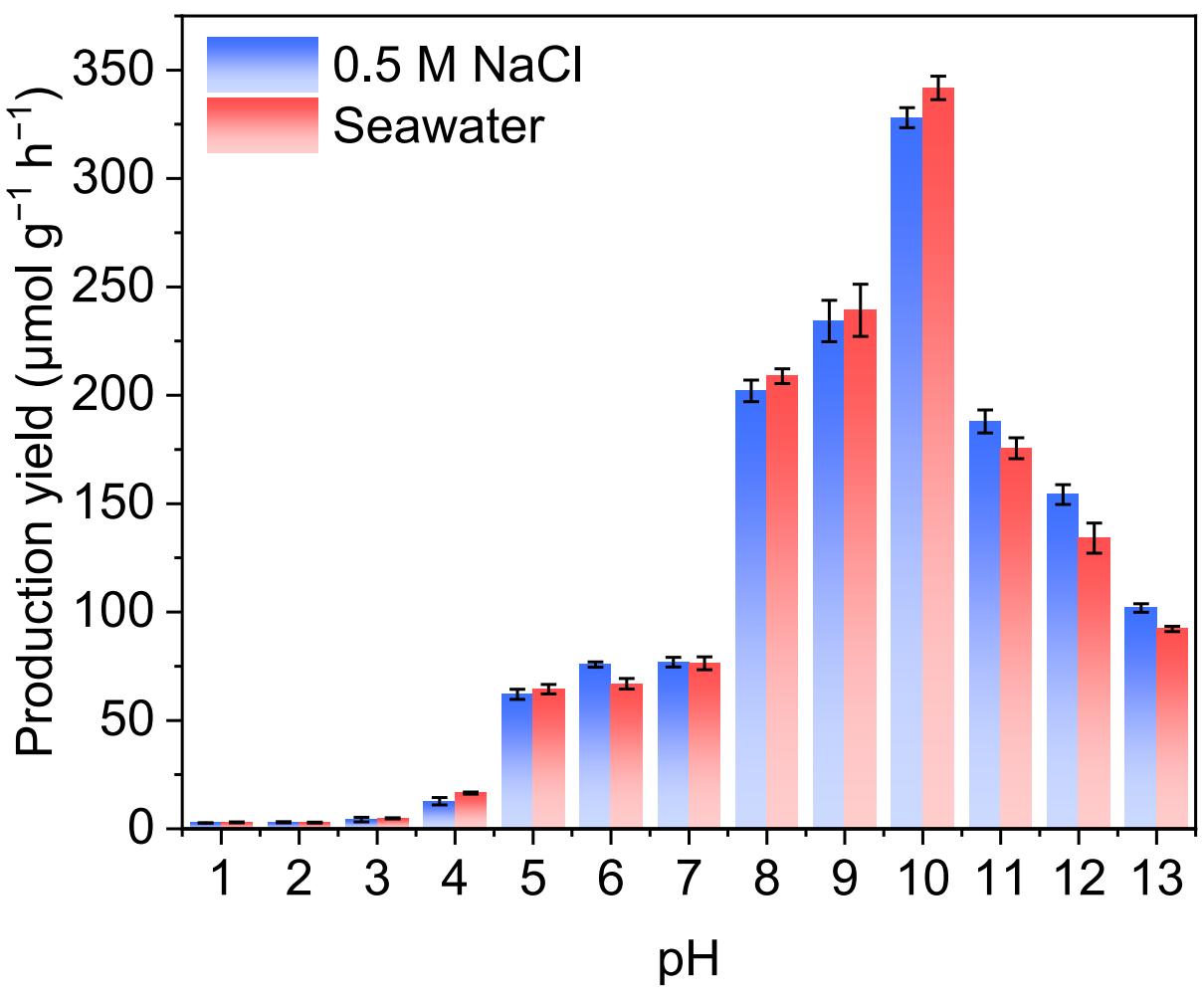

Fig. R30. Comparison of active chlorine production yields under different pH levels of natural seawater and NaCl solutions.

The corresponding discussions had been added in the revised Manuscript.

Detailed revision is as below. Thank you most sincerely!

In the revised Manuscript:

(Page 11) *Additionally, the pH of the CE-Chemistry system markedly affects the active chlorine yield, with the highest output obtained under mildly alkaline conditions (pH = 10, Supplementary Fig. 21). This pH dependence is governed not by a single factor, but by the combined effects of reactive species generation kinetics and the acid-base-equilibrium-dependent speciation of active chlorine. Under acidic conditions, active chlorine predominantly exists as HOCl*[53]*, which is more susceptible to decomposition under the localized and transient high temperatures generated during cavitation bubble collapse, thereby reducing the overall yield (equation (11)). In contrast, under strongly alkaline conditions, although* OCl^- *is thermodynamically more stable, the formation of •Cl is significantly suppressed, resulting in diminished active chlorine production. DFT calculations further indicate that, at excessively high pH, the high* OH^- *concentration causes FEP to preferentially extract electrons from hydroxide ions, suppressing the •Cl generation pathway (Route 2). On the other hand, excessively high pH also weakens the oxidizing ability of •OH*[77-80]*, which in turn suppresses the •Cl generation pathway in Route 1. As a result, both •Cl generation routes are inhibited to different extents,*

suppressing •Cl formation and active chlorine production under strongly alkaline conditions. Notably, the yield does not show a simple intermediate trend in the neutral range of pH = 6-8, because this region represents a sensitive transition window in which HOCl and OCl^- rapidly interconvert and the apparent yield is jointly regulated by •Cl generation, •OH participation, active chlorine speciation, and product stability. Consequently, the pH dependence exhibits a pronounced nonlinear behavior rather than a smooth transition, a phenomenon that has also been reported in photocatalytic and electrocatalytic active chlorine synthesis[58,59].

7. In Supplementary Fig. 17, what is the reason for the decrease in pH of the NaCl solution over time?

Response: We sincerely thank the reviewer for the valuable comment. The gradual pH decrease observed in NaCl solution during CE-Chemistry arises from the interfacial oxidation of water under ultrasonication-induced contact electrification, where water loses electrons to generate hydroxyl radicals (•OH) accompanied by proton (H^+) formation, consistent with prior literature reports (*Nat. Commun., 2022, 13, 130; Angew. Chem. Int. Ed., 2023, 21, e202300604; Adv. Mater., 2023, 35, 2304387*). While •OH is rapidly consumed via recombination, secondary reactions, or quenching,

the majority of generated protons progressively accumulate in the solution. As a result, the bulk pH decreases with prolonged reaction time. In contrast, natural seawater contains bicarbonate/carbonate buffering ion pairs (HCO_3^-/CO_3^-), which can maintain the pH relatively stable to some extent during operation.

8. For the two reaction routes, which of the three pathways for generating HOCl is the main reaction path?

Response: We are deeply appreciated to the reviewer's precious suggestions and valuable comments. To identify the dominant pathway among the three possible routes for HOCl generation (**Eq. R3-R5**), we supplemented DFT calculations focusing on the energy barriers associated with electron transfer from key intermediates (Cl^- and $\bullet HOCl^-$) to FEP, as these electron-loss processes reflect the relative difficulty of the reaction steps compared to other essentially processes (e.g., radical recombination or charge neutralization) (*Nat. Commun., 2025, 16, 11035*).

$$\mathbf{Cl^- \rightarrow Cl^- + \bullet OH \rightarrow \bullet HOCl^- \rightarrow HOCl} \qquad \textbf{(Eq. R3)}$$

$$Cl^- \rightarrow \bullet HOCl^- \rightarrow \bullet Cl \rightarrow \bullet Cl + \bullet OH \rightarrow HOCl \qquad \textbf{(Eq. R4)}$$

$$Cl^- \rightarrow \bullet Cl \rightarrow \bullet Cl + \bullet OH \rightarrow HOCl \qquad \textbf{(Eq. R5)}$$

The calculated results show that the electron transfer barrier from Cl^- is 2.959 eV, whereas that from $\bullet HOCl^-$ is significantly lower at 2.454 eV,

indicating that oxidation of the •HOCl⁻ intermediate is energetically more favorable (**Fig. R31**). When combined with the Gibbs free energy profile (**Fig. R32**), this clearly demonstrates that **Eq. R3**, the direct protonation pathway via the •HOCl⁻ intermediate (dashed line in **Fig. R32**), is the dominant reaction pathway. Specifically, in **Eq. R3**, the lower electron transfer barrier facilitates the formation and consumption of •HOCl⁻, and its subsequent conversion to HOCl is thermodynamically favorable ($\Delta G = -0.61$ eV), resulting in higher overall kinetic efficiency compared to alternative routes. ***Therefore, the combination of a reduced activation barrier and favorable reaction energetics establishes Eq. R3 in Route 1 as the primary pathway for selective HOCl generation in our system.***

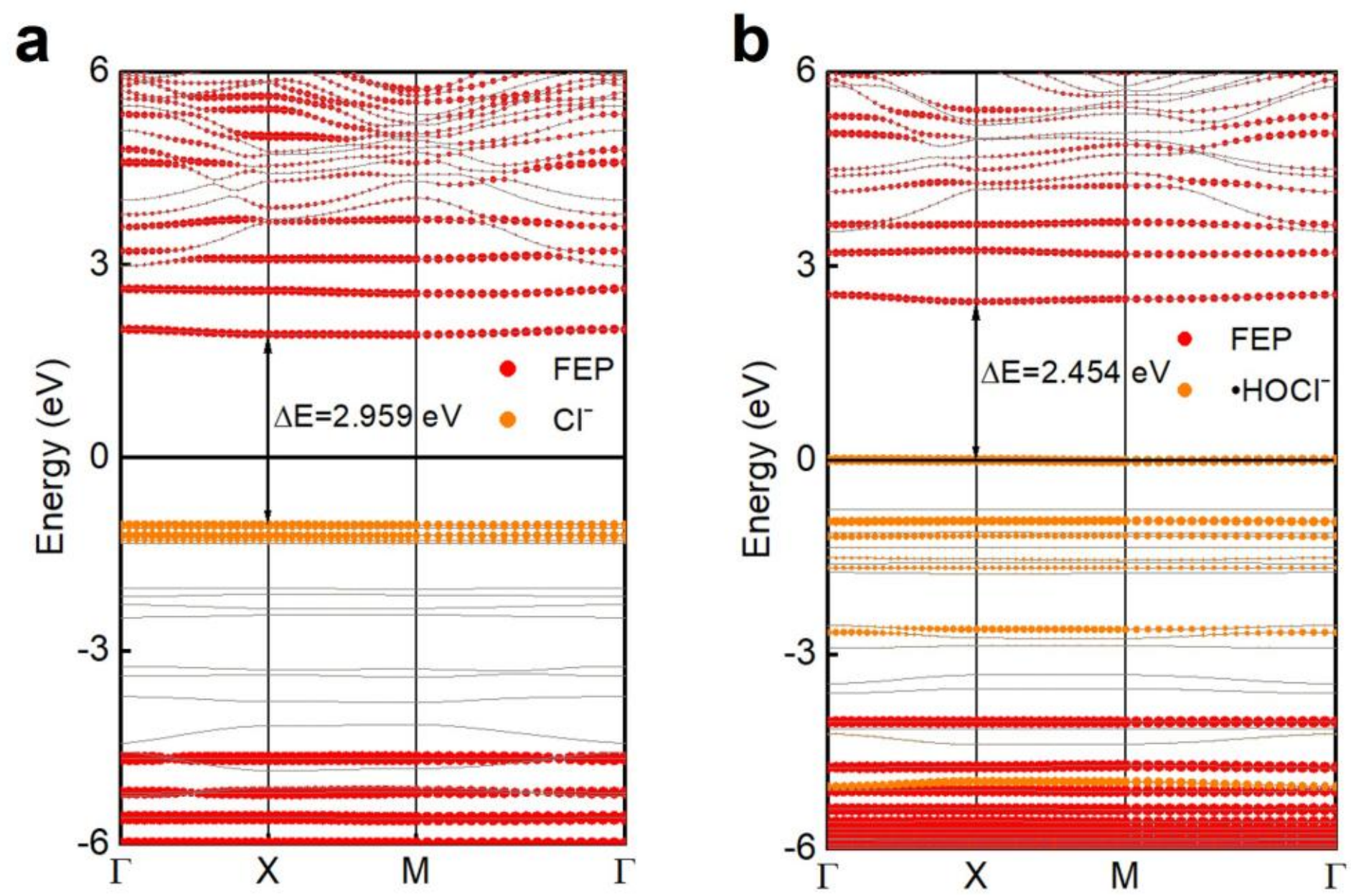

Fig. R31. Calculated energy barriers for electron transfer (a) from Cl⁻ to FEP and (b) from •HOCl⁻ to FEP.

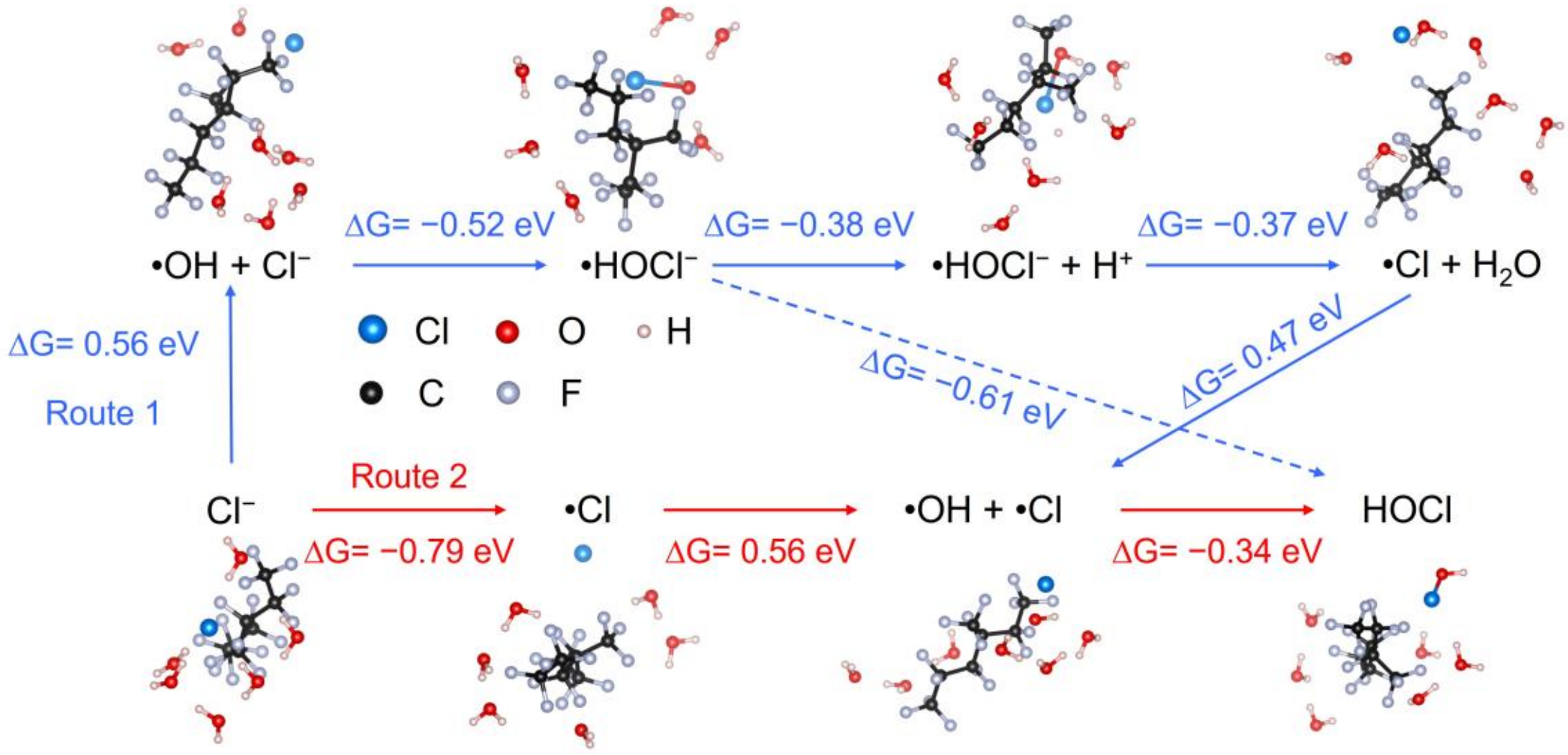

Fig. R32. The required free energy for each step under ultrasonication conditions in two different routes.

9. When the solution is acidic, the high temperature generated by ultrasonic cavitation will inhibit the reaction. The influence of temperature on EDL and the effect of synthesizing chlorine species through CE-Chemistry should be discussed and supplemented.

Response: We sincerely thank the reviewer for this insightful comment. We agree that the influence of temperature on the EDL and active chlorine generation requires further clarification. In CE-Chemistry, temperature affects not only reaction kinetics but also the structure and dynamics of the EDL. Specifically, increasing temperature enhances the diffusion and mobility of ions, particularly protons under acidic conditions. This accelerates charge screening at the solid-liquid interface, leading to more

rapid formation of a compact EDL (*Mater. Today, 2024, 74, 2-11*). As a result, electron transfer efficiency and subsequent reactive species generation are suppressed. In addition, higher bulk temperatures reduce the buffering effect against cavitation-induced local heating, which can further promote the thermal decomposition of active chlorine species. Conversely, at lower temperatures, slower ion diffusion delays EDL screening and helps maintain a stronger interfacial electric field, favoring reactive species generation, although excessively low temperatures may limit reaction kinetics (*Chem. Rev. 2022, 122, 5209-5232*; *Adv. Mater. 2023, 35, 2304387*). Overall, the temperature dependence reflects a balance between enhanced ion transport (which strengthens EDL screening) and interfacial charge utilization, together governing the efficiency of CE-Chemistry (**Fig. R33**). These considerations have now been incorporated into the revised manuscript to provide a more complete mechanistic understanding.

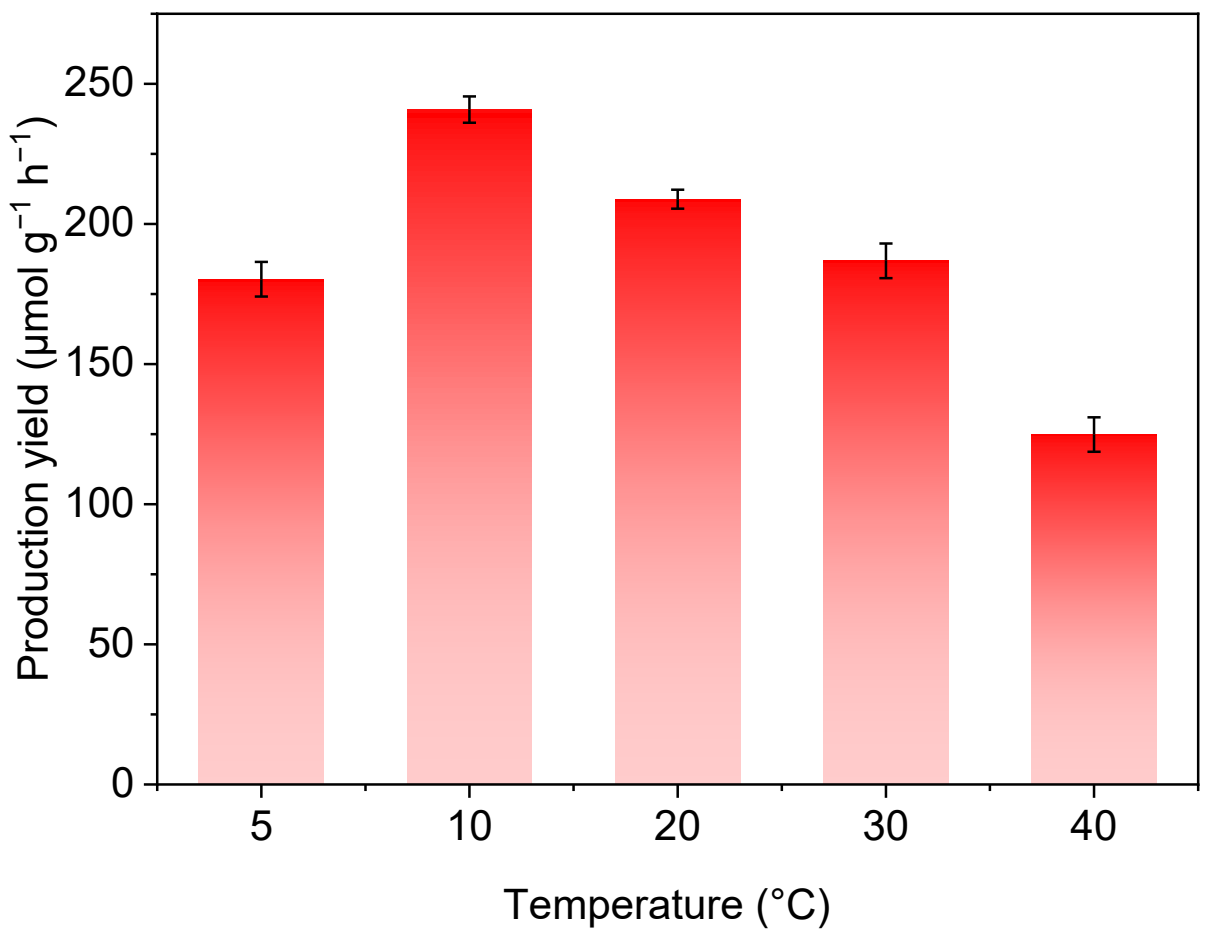

Fig. R33. Comparison of active chlorine production yields at different temperatures of natural seawater solution.

The corresponding discussions had been added in the revised Manuscript and revised Supplementary Information. Detailed revision is as below.

In the revised Manuscript:

(Page 6) *Temperature exerts a multifaceted influence on the reaction: relatively low temperatures favor product stability and accumulation, while excessively low temperatures limit reaction kinetics. In contrast, higher temperatures enhance reaction kinetics and mass transport, but also accelerate EDL formation and screening, which suppresses interfacial charge transfer and leads to a decrease in CE-Chemistry yield. Therefore, a trade-off exists between active chlorine generation and accumulation, resulting in optimal performance at 10 ℃ within the range of 5-40 ℃ [49,65-67] (Supplementary Fig. 3).*

(Page 20) *The reaction was conducted under optimal conditions of 20 °C, 20 mg FEP, 10 mL of natural seawater, and thermostatic ultrasonication at 40 kHz and 120 W for 1 h. For systematic investigations of the effects of solution volume (10, 25, 50, 75, 100 mL), temperature (5, 10, 20, 30, 40 °C), or ultrasonic power (60, 120, 200, 300, 400 W), all tests were designed based on these optimal conditions, with only a single variable altered each time.*

In the revised Supplementary Information:

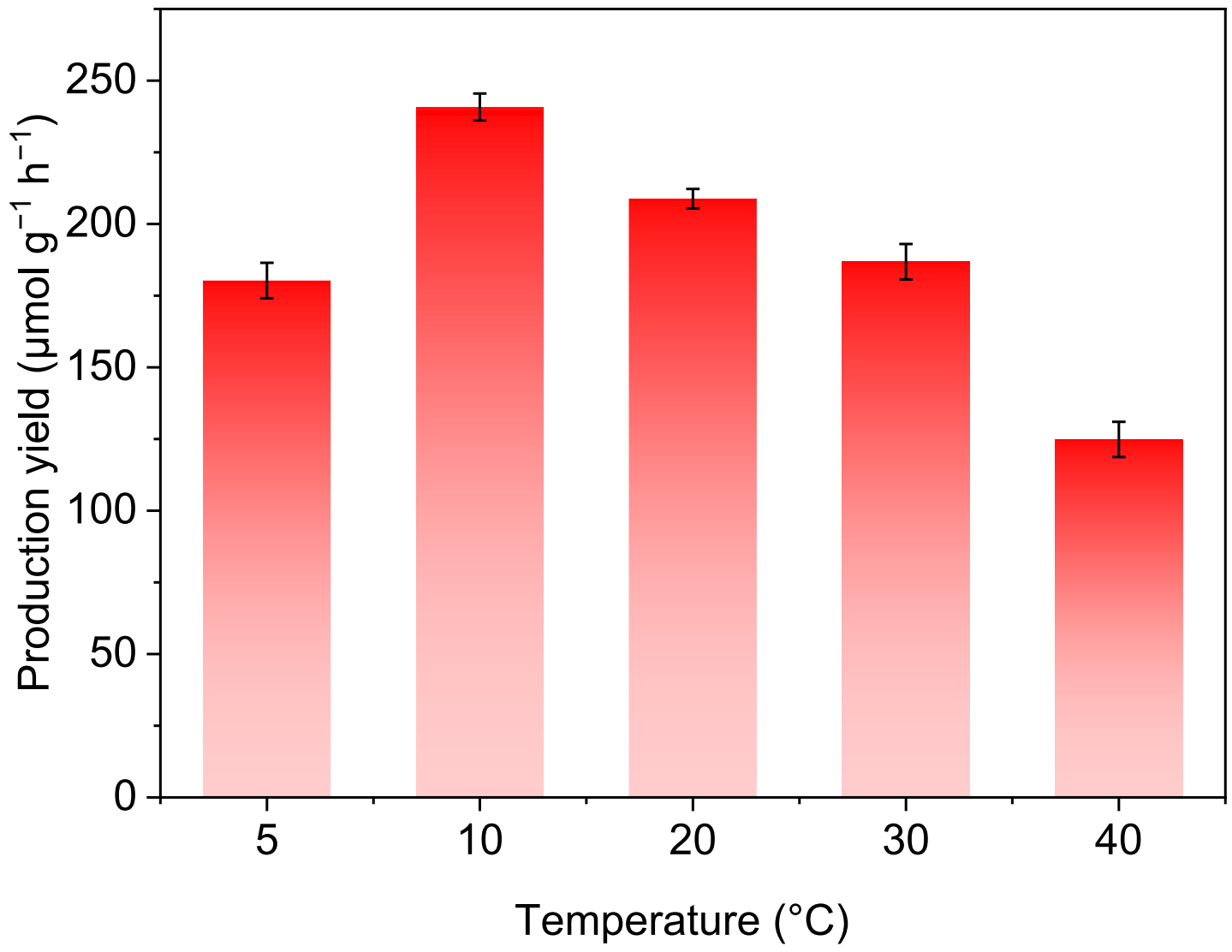

(Page 2) *Supplementary Figure 3. Comparison of active chlorine production yields at different temperatures of natural seawater solution.*

10. Is there any reference for the compression ratio of volume during the DFT calculation? Compared with previous literature, is the compression ratio of volume overly compressed?

Response: We sincerely thank the reviewer for raising this critical question and for the valuable suggestion. We agree that the description of the compression ratio used in the DFT calculations was not sufficiently clear in the original manuscript. After re-examining the relevant literature, we found that previous studies simulating the high-pressure environment induced by ultrasound-triggered cavitation bubble collapse in DFT

calculations have often employed a volume compression model, with a typical compression ratio of approximately 30% (for example, compressing the cell from 10 Å × 10 Å × 5 Å to 10 Å × 7 Å× 5 Å). In the present work, however, we adopted a linear compression model, with compressions of approximately 46.7%, 46.7%, and 40.0% along the three directions, corresponding to an equivalent average linear compression ratio of approximately 44.5%. This value is slightly higher than the 30% linear compression ratio reported in previous studies (*Nat. Commun., 2022, 13, 130*), which in fact reflects the difference between two compression schemes. Both models are essentially intended to simulate, to different extents, the extreme high-pressure environment generated by cavitation bubble collapse.

To assess whether the chosen volume range is excessively compressed, we calculated the Gibbs free energy profiles for the key reaction (•Cl + •OH → HOCl) under four different cell volumes. The results show that the overall free energy profile remains consistent across all volumes, indicating that the reaction mechanism is not altered by compression. Meanwhile, the decrease in Gibbs free energy becomes more pronounced with decreasing volume, confirming that elevated pressure enhances the thermodynamic driving force of the reaction (**Fig. R34**). Importantly, even at the smallest cell (8 Å × 8 Å × 15 Å), no anomalous or irregular behavior is observed, and the energy shows good convergence, suggesting that the

model does not suffer from over-compression artifacts. Therefore, the selected volume range is both physically reasonable and methodologically reliable, and it effectively captures the influence of pressure on the reaction thermodynamics.

Finally, this is indeed an important issue that deserves further investigation, because previous studies in this area remain very limited and no unified theoretical framework has yet been established. We will therefore specifically address the influence of different compression models and parameters on the reaction metrics in our future work.

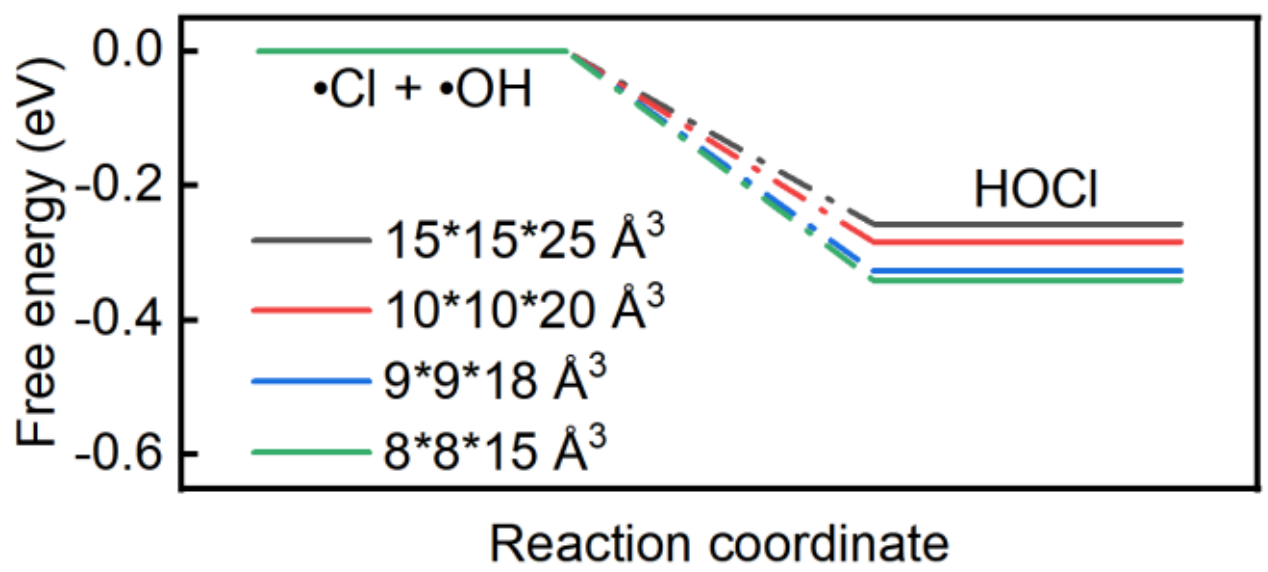

Fig. R34. Effect of unit cell volume on the Gibbs free energy of the reaction •Cl + •OH → HOCl.

The corresponding discussions had been added in the revised Manuscript and revised Supplementary Information. Detailed revision is as below. Thank you most sincerely!

In the revised Manuscript:

(Page 22) *Moreover, we have evaluated the effect of different unit cell volumes on the Gibbs free energy of the key reaction (•Cl + •OH →HOCl), which shows good energy convergence across the tested volume range, confirming that the selected cell sizes are reasonable (Supplementary Fig. 32).*

In the revised Supplementary Information:

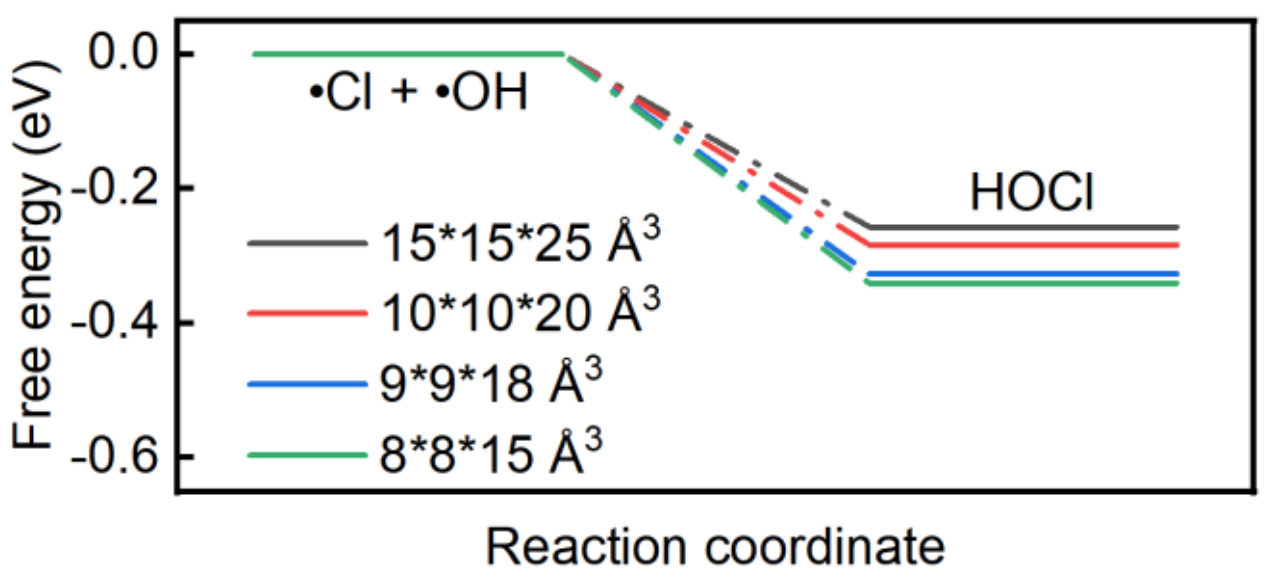

(Page 17) *Supplementary Figure 32. Effect of unit cell volume on the Gibbs free energy of the reaction •Cl + •OH → HOCl.*

11. Does the electric field generated by the contact electrification of solid-liquid interfaces play a dominant role in the interface electric field? What proportion does the EDL contribute to the overall electric field and promote the redox reaction process?

Response: We are deeply appreciated to the reviewer's precious suggestions and valuable comments to improve the paper. By introducing

mechanical stimulation, such as ultrasonication, at the solid-liquid interface, contact electrification can be induced, thereby promoting electron transfer and ion migration and ultimately leading to the formation of the EDL. In this process, the electrons accumulated on the surface of the solid dielectric material can give rise to an interfacial triboelectric field (*Chem. Soc. Rev., 2024, 53, 4349-4373; Chem. Rev. 2022, 122, 5209-5232*).

To further examine this point, we established a dedicated confocal Raman-NaSCN probe platform, inspired by previous gas-liquid interfacial electric field measurements (*Sci. Adv., 2025, 11, eadx5714; Acc. Chem. Res., 2026, 59, 285-297*). Specifically, 500 ppm FEP powder was dispersed in 2 M NaSCN and subjected to water-bath ultrasonication in a quartz reactor, enabling electron transfer at the solid-liquid interface and generating an interfacial triboelectric field (*Nat. Commun., 2020, 11, 399; Chem. Soc. Rev., 2024, 53, 4349-4373; Chem. Rev., 2022, 122, 5209-5232*).

Confocal Raman spectroscopy of the NaSCN probe revealed a Stark shift ($\Delta\nu_{C\text{-}N}$) of 51 cm^{-1} (**Fig. R35**), corresponding to an interfacial electric field of ~1.4×10^{10} V m^{-1} at the solid-liquid interface based on the established Stark effect relationship ($E = \Delta\nu_{C\text{-}N}/0.36$ MV cm^{-1}) (*J. Phys. Chem. Lett.,2020, 11, 7423-7428; J. Am. Chem. Soc., 2010, 132, 12784-12785*; (**Fig. R36, red line**). In the absence of ultrasonication, which triggers contact electrification, no Stark shift was observed (**Fig. R36, blue line**), indicating that the measured field originates from interfacial

triboelectric effects. Notably, this experimentally determined field is substantially higher than previously reported values for gas-liquid or liquid-liquid interfaces (*Nature, 2025, 640, 87-93; Acc. Chem. Res., 2026, 59, 285-297; Chem. Soc. Rev., 10.1039/d5cs01066g; Nat. Chem. Biol., 2024, 20, 1420-1433; Chem, 2023, 9, 1594-1609*) and exceeds our prior theoretical estimates, providing direct validation of the interfacial triboelectric field.

Based on these observations, we respectfully suggest that ***the contact electrification-induced triboelectric field makes a dominant contribution to the overall interfacial electric field in the present system***. Previous studies of multiphase interfaces, such as the gas-liquid interface of microdroplets and the liquid-liquid interface of emulsions, have also reported interfacial electric fields on the order of 10^6-10^9 V m^{-1}, arising from factors such as oriented water dipoles and ion redistribution (*Acc. Chem. Res., 2026, 59, 285-297; Chem. Soc. Rev., 10.1039/d5cs01066g*). In comparison, our current experimental results suggest that the contributions of these factors to the total electric field at the solid-liquid interface may be relatively limited, whereas contact electrification appears to be the predominant source of the interfacial field in our system. Meanwhile, we fully acknowledge that the origin of electric fields at multiphase interfaces, as well as the quantitative distinction between the respective contributions of different factors, remains an important and challenging issue in this field.

We believe that this question could be addressed more rigorously in future studies with continued advances in characterization techniques and theoretical simulations.

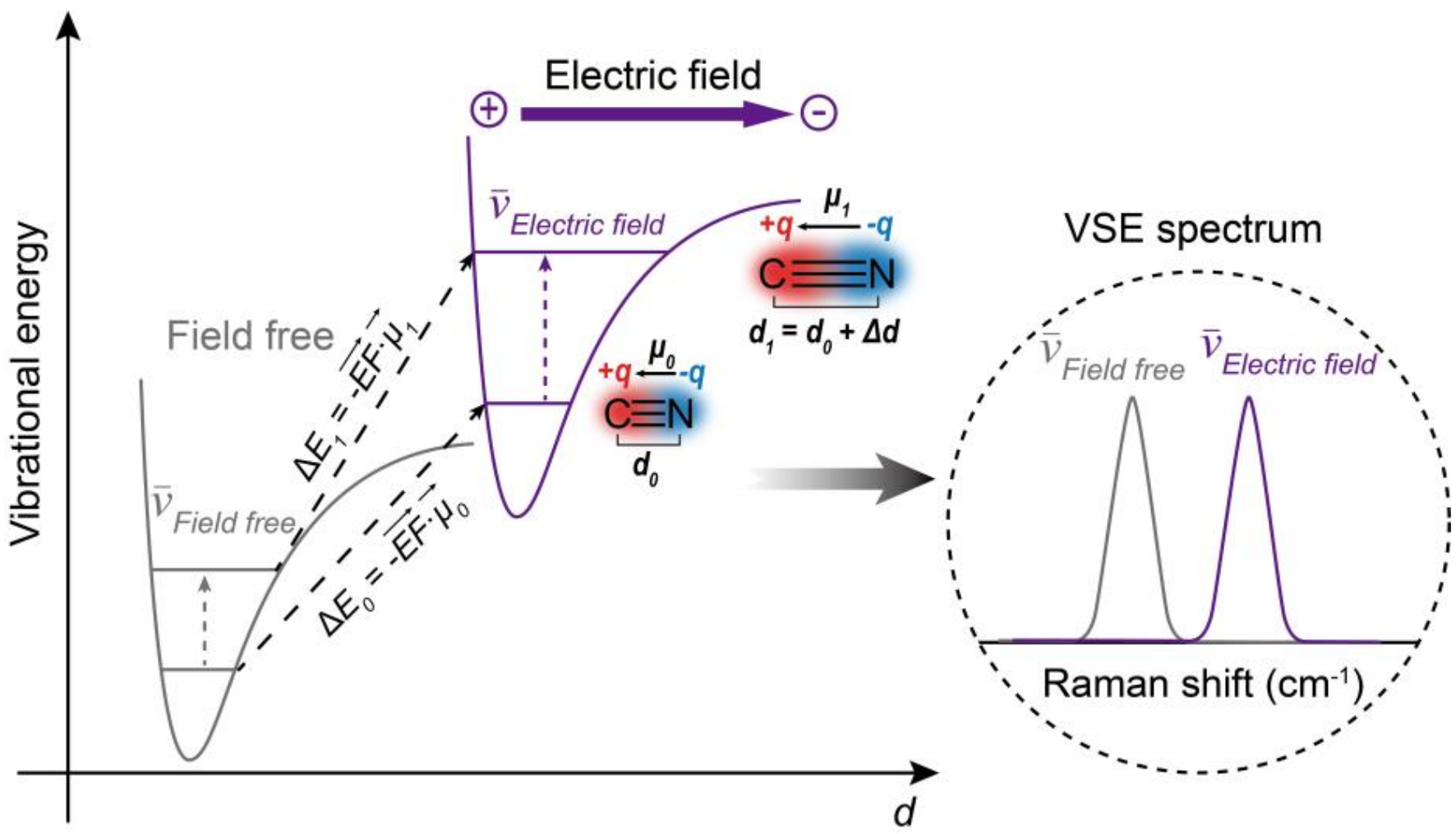

Fig. R35. Illustration of vibrational Stark effect (VSE).

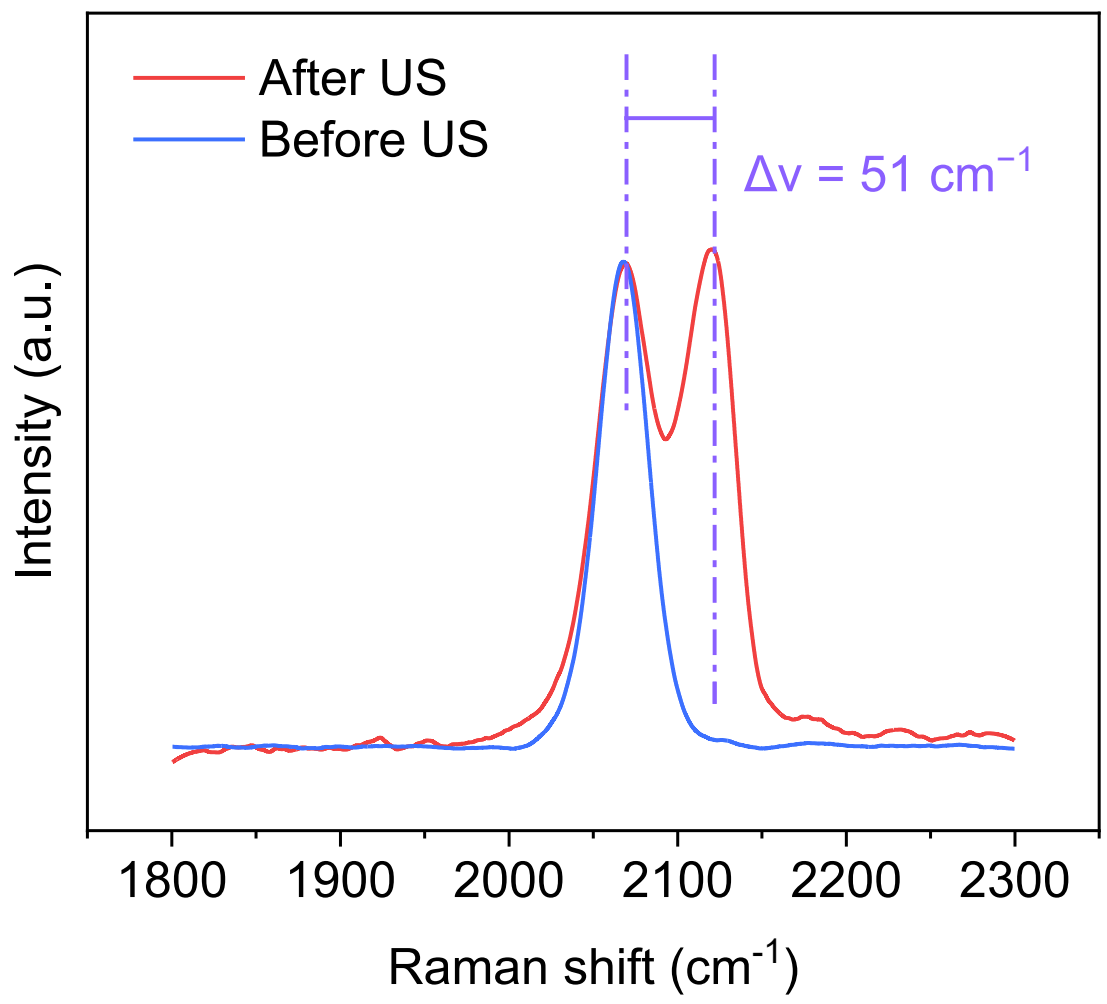

Fig. R36. The dedicated confocal Raman-NaSCN probe platform demonstrates that the triboelectric field at the solid-liquid interface can reach 1.4×10^{10} V m^{-1}.

In addition, as the reviewer insightfully noted, both oxidation and reduction processes are involved in CE-Chemistry. These reactions are generally enabled by various reactive species generated under the strong interfacial electric field, including reactive oxygen species (ROS), such as hydroxyl radicals (•OH) and superoxide radicals ($\bullet O_2^-$); as well as electrons accumulated on the dielectric surface. Hydroxyl radicals and electrons primarily initiate oxidation and reduction reactions, respectively, whereas superoxide radicals may participate in either process depending on the substrate involved (*J. Am. Chem. Soc., 2025, 147, 25407-25416; Nano Energy, 2024, 122, 109286*). Therefore, in our understanding, the interfacial electric field plays important roles in inducing the generation of reactive species and, to some extent, in lowering reaction barriers, stabilizing reaction intermediates, and regulating the distribution of charged species (*Nature, 2016, 537, 382-386; J. Am. Chem. Soc., 2025, 147, 36136-36145; Acc. Chem. Res., 2026, 59, 285-297; Chem. Soc. Rev., 10.1039/d5cs01066g; Chem. Soc. Rev., 2024, 53, 2022*).

The corresponding discussions had been added in the revised Manuscript. Detailed revision is as below. Thank you most sincerely!

In the revised Manuscript:

(Page 13) *Accordingly, under identical conditions, the EDL in KCl solution is more compact than in NaCl or LiCl solutions (Supplementary Fig. 26), with the interfacial triboelectric field within the Stern layer estimated at 1.46 × 10^{10} V m^{-1}, exceeding that of NaCl (1.35 × 10^{10} V m^{-1}) and LiCl (1.26 × 10^{10} V m^{-1}) (Fig. 4f).*

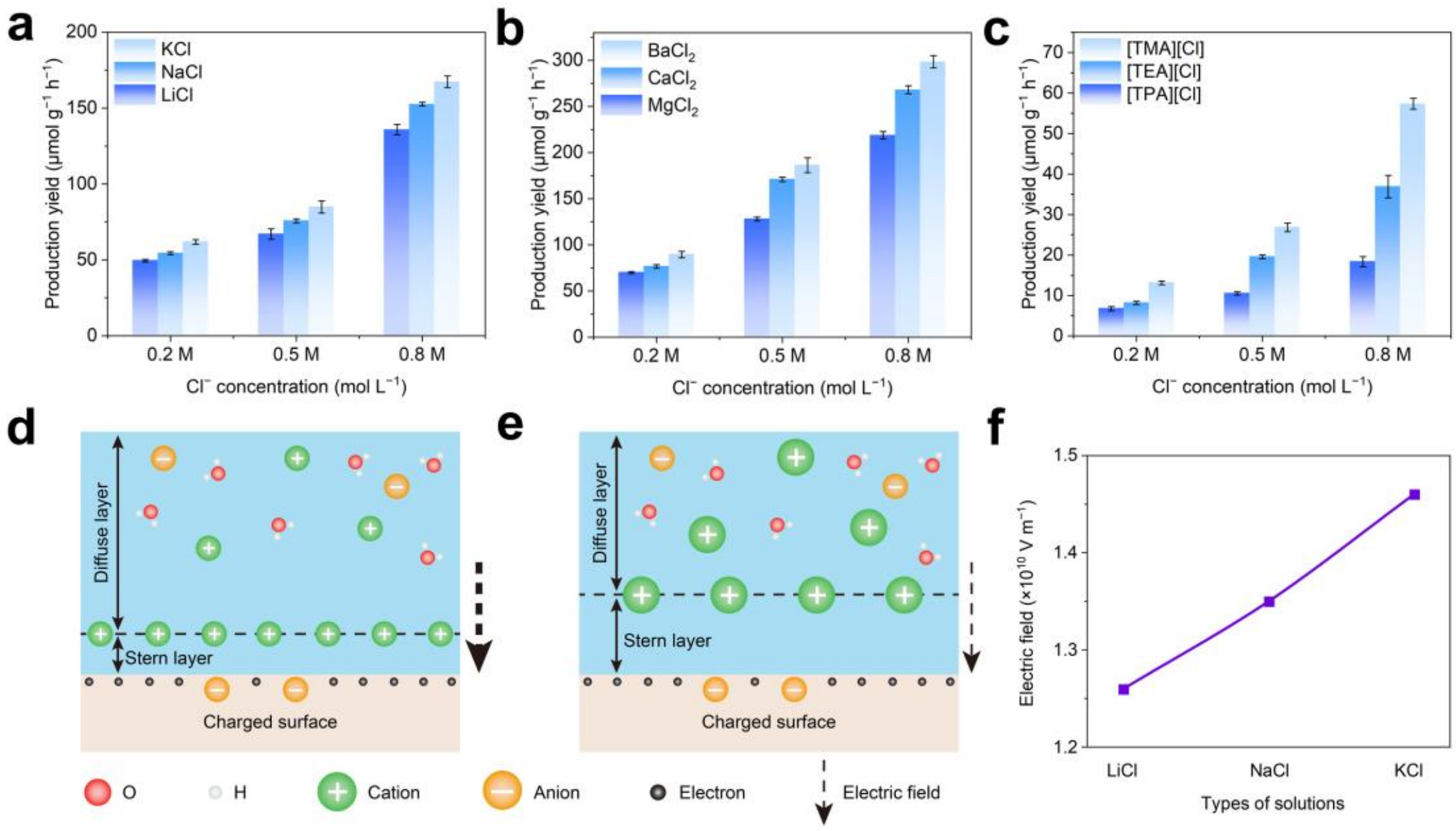

(Page 28) *Fig. 4 Proposed model of the impact of cation effect on CE-Chemistry. f, Comparison of interfacial triboelectric fields within the Stern layer in different salt solutions.*

12. Through the comparison between Fig. 4a and 4b, the radius and performance of K^+ and Ca^{2+} do not follow exactly the law in the manuscript. The influence of ion valence states on EDL and reactions should be carefully discussed.

Response: We sincerely thank the reviewer for this insightful comment. We agree that the comparison between **Fig. 4a** and **4b** requires more careful interpretation, particularly regarding the role of ion valence. When considered separately, both monovalent and divalent cations follow the expected trend that a smaller hydrated radius corresponds to a higher active chlorine yield. However, this relationship is valid only within the same valence state.

In our experiments, the Cl^- concentration was fixed, resulting in different cation concentrations for different electrolytes (e.g., 0.5 M KCl vs. 0.25 M $CaCl_2$). Consequently, the concentration of Ca^{2+} is only half that of K^+, and direct comparison based solely on hydrated radius is not appropriate. Although K^+ has a smaller hydrated radius than Ca^{2+}, the lower yield observed in KCl can be attributed, at least in part, to stronger EDL screening arising from the higher cation concentration, which suppresses interfacial charge transfer and reaction kinetics (*J. Am. Chem. Soc., 2024, 146, 31574-31584; Adv. Mater., 2023, 35, 2304387; Angew. Chem. Int. Ed., 2024, 63, e202403114; Chem. Soc. Rev., 2024, 53, 4349-4373; Chem. Rev., 2022, 122, 5209-5232*). Previous related studies have also mainly discussed the influence of ions with the same valence state on reaction kinetics (*Nat. Catal., 2025, 8, 79-91; Sci. Adv., 2020, 6, eabd2569; Energy Environ. Sci., 2019, 12, 3001-3014*). We further note that ion effects on EDL structure and reactivity arise from the coupled influence of valence,

concentration, hydration, and interfacial organization, making it difficult to establish a universal trend across different valence states. Accordingly, we have revised the manuscript to avoid overgeneralization and to clarify the distinct roles of ion valence and concentration.

The corresponding discussions had been added in the revised Manuscript and revised Supplementary Information. Detailed revision is as below. Thank you most sincerely!

In the revised Manuscript:

(Page 13) *Figures 4a-c illustrated a consistent trend in active chlorine yield across different concentrations, valences, and inorganic/organic cations: smaller cation hydration sizes correlate with higher yields (under otherwise identical conditions).*

(Page 13) *The hydration sizes of alkali metal cations follow the sequence* $K^+ < Na^+ < Li^+$ *(Supplementary Table 4).*

In the revised Supplementary Information:

(Page 19) *Supplementary Table 4. Summary of the hydrated radii of the investigated ions.*

Ion	***Hydrated radius (Å)***
K^+	*3.31*
Na^+	*3.58*
Li^+	*3.82*
Ba^{2+}	*4.04*
Ca^{2+}	*4.12*
Mg^{2+}	*4.28*

13. How does the addition of salt solution affect the electrochemical impedance, and how does it affect the CE-chemistry reaction? These should be discussed.

Response: We sincerely thank the reviewer for this insightful comment. We agree that the role of salt solutions in both electrochemical impedance and CE-Chemistry performance requires further clarification. To address this point, we supplemented electrochemical impedance and conductivity measurements for salt solutions with different concentrations and cation species using an electrochemical workstation and a conductivity meter. The results show that the impedance of the salt solutions follows the same trend as the hydrated radius of the cations: for ions with the same valence state, a smaller hydrated radius corresponds to a lower impedance (**Table R5 and R6**).

Considering that CE-Chemistry is intrinsically an interfacial process, its behavior is governed more strongly by the interfacial electric field and the generation of reactive species at the solid-liquid interface than by the bulk solution resistance. This distinction is reflected in the dependence of active chlorine yield on salt concentration. While impedance measurements show a continuous decrease with increasing salt concentration over the range of 10^{-2} to 1.5 M, the active chlorine yield does not exhibit a corresponding monotonic increase, indicating that interfacial electrostatics rather than bulk conductivity dominate the reaction outcome. As shown in **Fig. 2d (Fig. R37)**, the active chlorine yield increases with Cl^- concentration up to ~1.1 M and then decreases, reflecting a balance between enhanced reactant availability and suppressed interfacial electron transfer due to stronger EDL screening. Furthermore, cation identity plays a critical role. For cations with the same valence, a smaller hydrated radius leads to higher yields. Mechanistically, such cations form a more compact Stern layer, strengthening the local interfacial electric field, facilitating electron transfer, and promoting the accumulation of Cl^- and reactive intermediates near the interface. In contrast, larger hydrated cations weaken these effects and suppress the reaction, which is also consistent with previous reports (*Sci. Adv., 2020, 6, eabd2569; Nat. Catal., 2025, 8, 79-91; Energy Environ. Sci., 2019, 12, 3001-3014*). Overall, we conclude that salt solutions influence CE-Chemistry predominantly through

modulation of the interfacial EDL structure and local reaction environment, rather than through bulk impedance.

Table R5. Electrochemical impedance (R_S) and conductivity (σ) of different solutions.

**Salts**	**Concentration (M)**	R_S (Ω)	σ (S m^{-1})
KCl	1.5	5.90	15.01
NaCl	1.5	7.23	11.36
LiCl	1.5	7.87	9.44
KCl	1	7.49	10.20
NaCl	1	9.08	7.95
LiCl	1	9.93	6.79
KCl	10^{-1}	60.15	1.21
NaCl	10^{-1}	71.79	1.01
LiCl	10^{-1}	79.79	0.91
KCl	10^{-2}	547.87	0.14
NaCl	10^{-2}	662.38	0.12
LiCl	10^{-2}	720.25	0.10
$BaCl_2$	1.5	4.98	16.41
$CaCl_2$	1.5	5.22	16.16
$MgCl_2$	1.5	5.92	13.60
$BaCl_2$	1	6.28	12.52
$CaCl_2$	1	6.34	12.43
$MgCl_2$	1	7.18	10.56
$BaCl_2$	10^{-1}	40.05	1.82

$CaCl_2$	10^{-1}	40.88	1.81
$MgCl_2$	10^{-1}	44.21	1.66
$BaCl_2$	10^{-2}	326.16	0.23
$CaCl_2$	10^{-2}	332.74	0.22
$MgCl_2$	10^{-2}	358.19	0.21

Table R6. Summary of the hydrated radii of the investigated ions.

Ion	Hydrated radius (Å)
K^+	3.31
Na^+	3.58
Li^+	3.82
Ba^{2+}	4.04
Ca^{2+}	4.12
Mg^{2+}	4.28

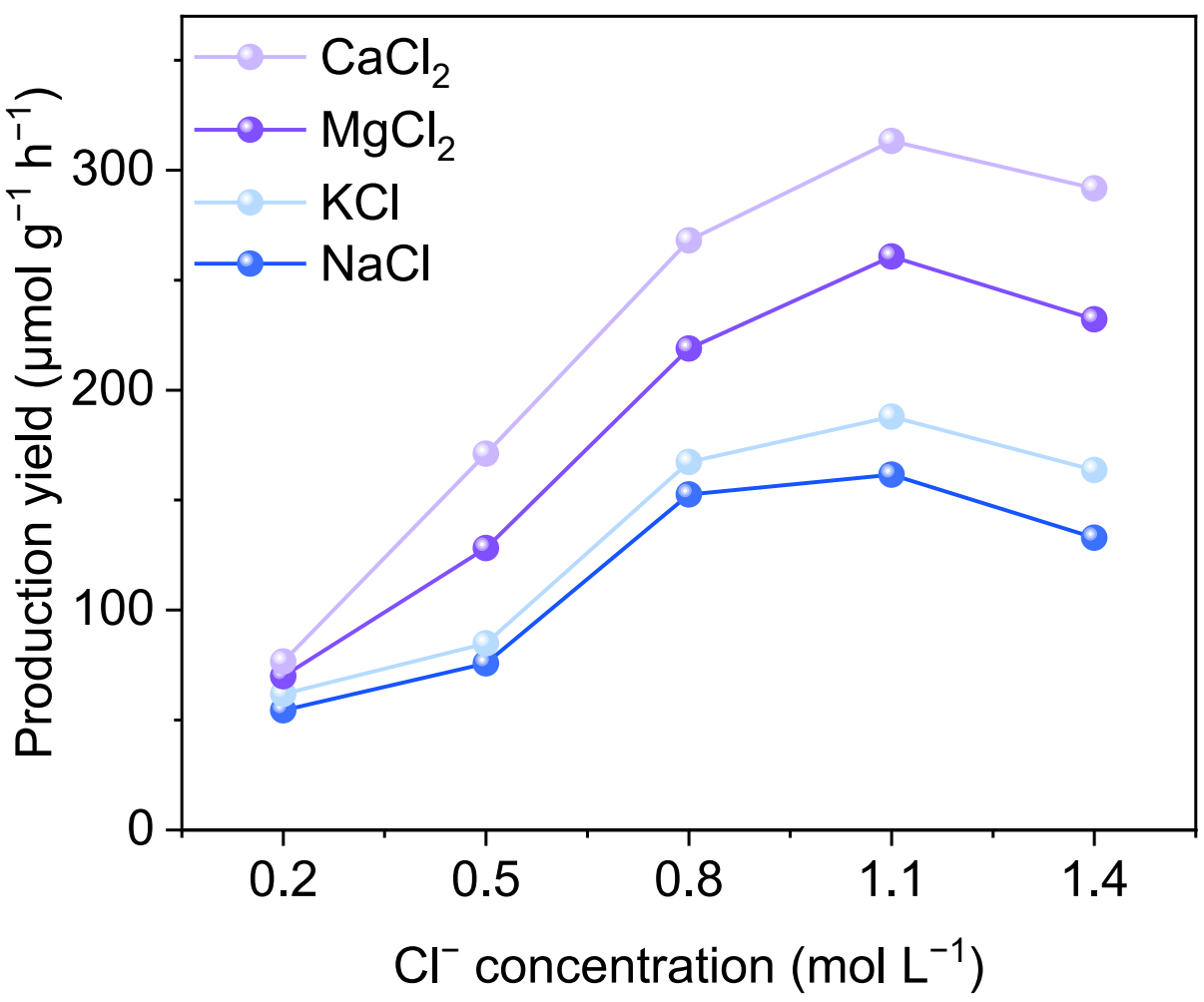

Fig. R37. Comparison of active chlorine production yields under different concentrations of Cl^- from 0.2 M to 1.4 M.

The corresponding discussions had been added in the revised Manuscript and revised Supplementary Information. Detailed revision is as below. Thank you most sincerely!

In the revised Manuscript:

(Page 6) *As shown in Fig. 2d, the relationship between chloride ion concentration and active chlorine yield exhibits a nonlinear trend: the yield increases from 0.2 M to a maximum at ~1.1 M and then declines. This behavior reflects a balance between two competing effects. Higher reactant concentration initially promotes the reaction, but excessive ion concentration intensifies EDL shielding, which ultimately dominates and suppresses interfacial electron transfer and subsequent chemical transformations at the solid-liquid interface*[15].

In the revised Supplementary Information:

(Page 19) *Supplementary Table 4. Summary of the hydrated radii of the investigated ions.*

Ion	***Hydrated radius (Å)***
K^+	*3.31*
Na^+	*3.58*
Li^+	*3.82*
Ba^{2+}	*4.04*
Ca^{2+}	*4.12*
Mg^{2+}	*4.28*

14. How does the work compare with other works that produce chlorine disinfectants, and does it have a certain advantage in cost, as well as the advantages of industrialization?

Response: We sincerely thank the reviewer for this insightful comment regarding energy efficiency and sustainability. In response, we quantified the energy consumption of the system under optimized conditions: with 20 mg FEP in 10 mL of natural seawater subjected to 120 W ultrasonication for 1 h, the active chlorine yield reaches 208.8 μmol g^{-1} h^{-1}, corresponding to an absolute production rate of ~4.18 μmol h^{-1}. This translates to an energy consumption of ~3.9×10^5 kWh kg^{-1} active chlorine. For comparison, this is considerably higher than the industrial chlor-alkali process or representative emerging systems such as $NbClO_x/BiVO_4$ (*Nat.*

Sustain., 2025, 8, 672-681; Nat. Sustain., 2024, 7, 148-157). We therefore fully agree that, in its current batch ultrasonication configuration, CE-Chemistry does not yet compete in terms of unit-product energy efficiency.

However, it is important to clarify that the sustainability discussed in our work does not refer to energy efficiency per unit product, but rather to the process itself: ***CE-Chemistry generates active chlorine without producing toxic Cl_2, avoids noble-metal catalysts or external electrical bias, and operates directly in complex media such as natural seawater.*** In this sense, the system represents a fundamental scientific advance, overcoming limitations inherent to conventional chlorination methods.

Moreover, while CE-Chemistry currently requires more energy per unit product than industrial processes, the energy input does not need to come from an external power source. ***The system relies on contact-separation-induced contact electrification, which can, in principle, be driven by low-frequency mechanical energy naturally present in marine environments (e.g., waves, fluid flow, or vibrations)***. In particular, marine wave energy alone has an annual potential exceeding 10,000 TWh, highlighting the great promise of the ocean as a sustainable energy source (*Ocean, 2025, 1, 9470004; Adv. Mater., 2025, 37, e11283*). If realized, this approach could decouple chemical production from conventional high-energy electrical inputs, achieving active chlorine synthesis through

ubiquitous ambient energy, an opportunity that conventional high-efficiency methods cannot access.

Such oceanic mechanical energy could, in principle, directly drive CE-Chemistry for green active chlorine production or, via energy-harvesting technologies, be converted to electrical power for downstream processes (*Nano-Micro Lett., 2025, 17, 124; Adv. Mater., 2025, 37, e11283*). Therefore, the system's most promising applications are not in centralized chlorine manufacture, but in distributed scenarios inaccessible to traditional chlor-alkali methods, e.g., ship-surface coatings, marine equipment interfaces, coastal or small-scale water-treatment units, and emergency disinfection, where ambient waves or fluid motion continuously trigger CE-Chemistry reactions for localized, on-demand antibacterial and anticorrosion functions. Accordingly, the sustainability of this work is better reflected in its novel reaction pathway and application mode, rather than in unit-product energy consumption at this stage.

Accordingly, we have revised the manuscript to moderate claims regarding sustainability. The work is now presented as a proof-of-concept interfacial chemistry platform that introduces a Cl_2-free reaction pathway and highlights a fundamentally new mode of energy-chemistry coupling, rather than as an immediate energy-efficient alternative to established industrial processes. We sincerely appreciate the reviewer's suggestion,

which has strengthened both the scientific clarity and positioning of the manuscript. The corresponding discussions have been revised accordingly.

The corresponding discussions had been moderated and revised in the revised Manuscript. Detailed revisions are as below. Thank you most sincerely!

In the revised Manuscript:

(Page 2) *Here, we reframe CE as a radical-generating mechanism at solid-liquid interfaces and establish contact-electro-chemistry (CE-Chemistry) as a sustainable paradigm, capable of harnessing abundant ambient mechanical energy to drive chemical transformations without the need for metal catalysts.*

(Page 2) *Utilizing a triboelectric radical generation route from natural seawater, this work demonstrates a metal-catalyst-free and sustainable strategy for active chlorine production, achieving a yield of 208.8 μmol g^{-1} h^{-1} with only 20 mg of FEP powder.*

(Page 2) *This work introduces a green and Cl_2-free route for disinfectant production directly from brine and positions CE as a general trigger for*

radical chemistry, opening avenues for heterogeneous catalysis and CE-driven reaction design.

(Page 4) *At present, industrial-scale production of active chlorine predominantly relies on the chlor-alkali process, in which chlorine gas (Cl_2) is generated as a corrosive and toxic intermediate*[55,56]*. Although this process is industrially mature, it faces inherent safety concerns and limitations for distributed applications: the transportation and storage of Cl_2 are subject to strict requirements and may pose serious environmental and health risks*[57]*. A promising pathway lies in green, metal-free synthetic strategies that bypass Cl_2 generation altogether and may enable distributed active chlorine production by harnessing high-entropy mechanical energy from the environment. By directly producing active chlorine from abundant natural seawater, this strategy circumvents the dependence on high-purity water and the hazards of Cl_2, while enabling green and sustainable distributed production*[58,59]*.*

(Page 5) *This work delivers a safe, sustainable, and environmentally benign route for distributed active chlorine production directly from seawater, circumventing the limits of conventional electrochemical windows, while also revealing the central role of interfacial triboelectric*

fields in CE-Chemistry and demonstrating how field strength and reaction pathways can be precisely tuned through regulation of the EDL.

(Page 15) *Such a route may be particularly relevant for niche scenarios that are difficult to address by centralized chlor-alkali infrastructure, such as marine equipment interfaces, ship coatings, localized antibiofouling/anticorrosion systems, and small-scale or emergency disinfection units.*

(Page 15) *This work provides a metal-free, CE-driven proof-of-concept for interfacial chemical transformations, with the potential to enable distributed reactions in the future by harnessing high-entropy mechanical energy from the environment.*

15. It is recommended to supplement the charge generation performance of different salt solutions and FEP solid-liquid CE.

Response: We sincerely thank the reviewer for this important and constructive suggestion. We fully agree that supplementing the charge generation performance of solid-liquid contact electrification under different salt-solution conditions is essential for a clearer understanding of the relationship between "charge generation" and "reaction behavior" in

CE-Chemistry. To address this point, we further supplemented charge transfer experiments at the FEP solid-liquid interface under various salt-solution conditions, including different salt concentrations (10^{-2} M, 10^{-1} M, and 1 M) and different cation species (K^+, Na^+, Li^+, Ba^{2+}, Ca^{2+}, and Mg^{2+}). The transferred charge was measured using a Faraday cup (**Fig. R38**). The results show that the total transferred charge does not differ significantly under different ion species or salt concentrations (**Fig. R39**). These results indicate that, in the present system, the influence of different salt solutions on CE-Chemistry reaction performance may not mainly originate from substantial changes in the total charge generated during solid-liquid contact electrification, but is more likely associated with the regulation of the interfacial EDL structure, the local triboelectric fields, and the migration and enrichment behavior of reactive species (*Sci. Adv., 2020, 6, eabd2569; Nat. Catal., 2025, 8, 79-91; Energy Environ. Sci., 2019, 12, 3001-3014; Nat. Commun., 2026, 17, 402*).

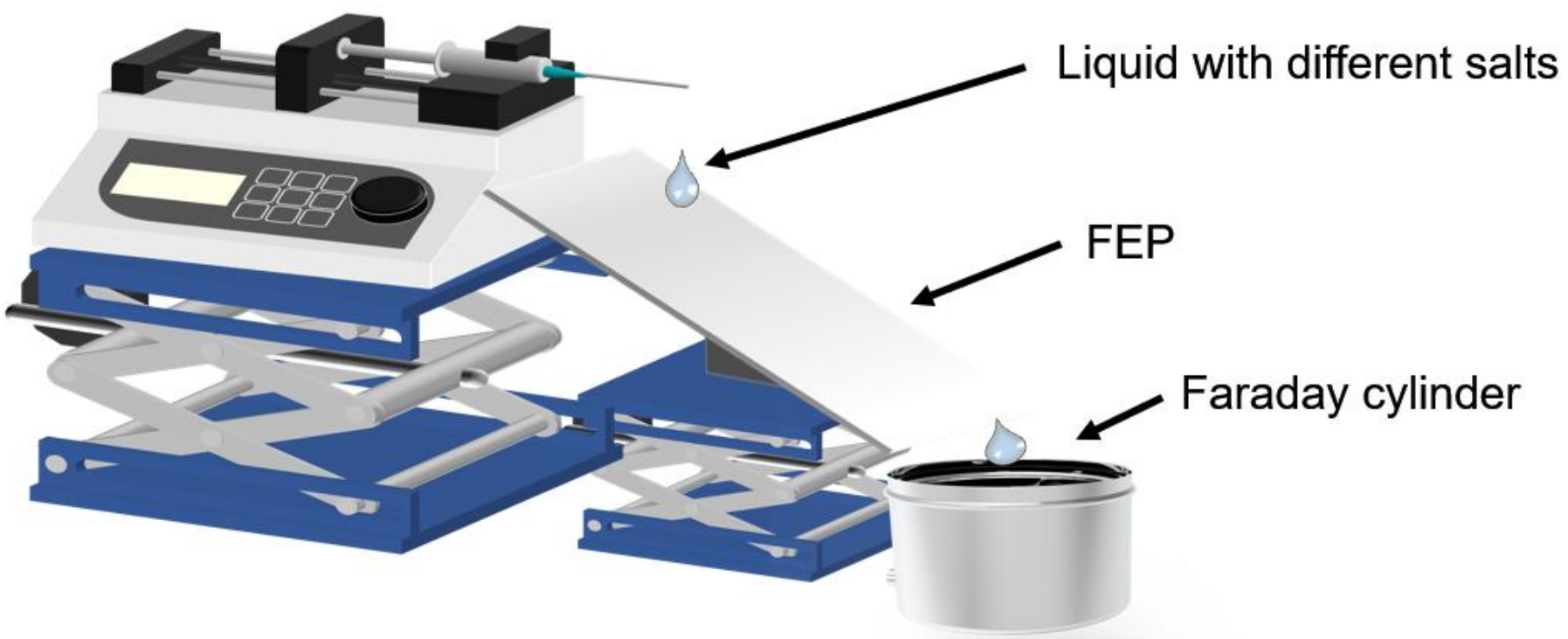

Fig. R38. Illustration of the Faraday cylinder testing apparatus.

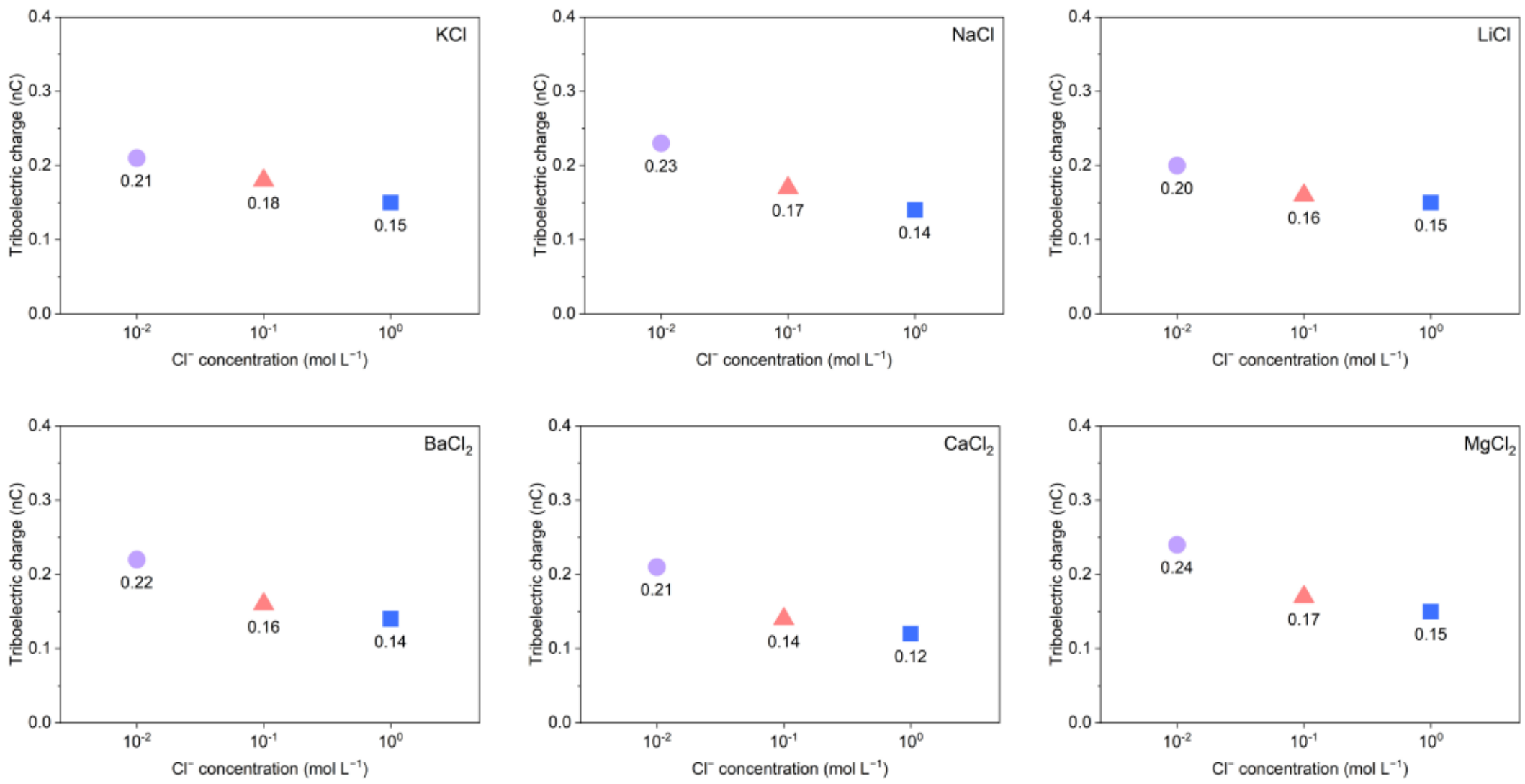

Fig. R39. The transferred charge by droplets of different solution passing over the FEP at different concentrations.
