# Supplementary material for: A triboelectric radical generation route to chlorine disinfectants from brine
Source: Nat Commun. 2026 Jul 7;17:8377. doi: 10.1038/s41467-026-75108-3 (PMC13473123; doi:10.1038/s41467-026-75108-3)
Supplement: Supplementary file 3 [file 41467_2026_75108_MOESM3_ESM.pdf]

Description of Additional Supplementary Files

File Name: Supplementary Data 1

Description: The atomic coordinates of the optimized computational models of FEP + H_2O + Cl^- under normal pressure

File Name: Supplementary Data 2

Description: The atomic coordinates of the optimized computational models of FEP + H_2O + •OH + Cl^- under normal pressure

File Name: Supplementary Data 3

Description: The atomic coordinates of the optimized computational models of FEP + H_2O + $•HOCl^-$ under normal pressure

File Name: Supplementary Data 4

Description: The atomic coordinates of the optimized computational models of FEP + H_2O + $•HOCl^-$ + H^+ under normal pressure

File Name: Supplementary Data 5

Description: The atomic coordinates of the optimized computational models of FEP + H_2O + •Cl + H_2O under normal pressure

File Name: Supplementary Data 6

Description: The atomic coordinates of the optimized computational models of FEP + H_2O + •OH + •Cl under normal pressure

File Name: Supplementary Data 7

Description: The atomic coordinates of the optimized computational models of FEP + H_2O + HOCl under normal pressure

File Name: Supplementary Data 8

Description: The atomic coordinates of the optimized computational models of FEP + H_2O + Cl^- under high pressure

File Name: Supplementary Data 9

Description: The atomic coordinates of the optimized computational models of FEP + H_2O + •OH + Cl^- under high pressure

File Name: Supplementary Data 10

Description: The atomic coordinates of the optimized computational models of FEP + H_2O + •$HOCl^-$ under high pressure

File Name: Supplementary Data 11

Description: The atomic coordinates of the optimized computational models of FEP + H_2O + •$HOCl^-$ + H^+ under high pressure

File Name: Supplementary Data 12

Description: The atomic coordinates of the optimized computational models of FEP + H_2O + •Cl + H_2O under high pressure

File Name: Supplementary Data 13

Description: The atomic coordinates of the optimized computational models of FEP + H_2O + •OH + •Cl under high pressure

File Name: Supplementary Data 14

Description: The atomic coordinates of the optimized computational models of FEP + H_2O + HOCl under high pressure

File Name: Supplementary Data 15

Description: The atomic coordinates of the optimized computational models of FEP + H_2O +

•Cl under normal pressure

File Name: Supplementary Data 16

Description: The atomic coordinates of the optimized computational models of FEP + H_2O + •Cl under high pressure

File Name: Supplementary Data 17

Description: The atomic coordinates of the optimized computational models of FEP + H_2O + K

File Name: Supplementary Data 18

Description: The atomic coordinates of the optimized computational models of FEP + H_2O + Na

File Name: Supplementary Data 19

Description: The atomic coordinates of the optimized computational models of FEP + H_2O + Li

File Name: Supplementary Data 20

Description: The atomic coordinates of the optimized computational models of FEP + H_2O + •Cl + •Cl under high pressure

File Name: Supplementary Data 21

Description: The atomic coordinates of the optimized computational models of FEP + H_2O + Cl_2 under high pressure

File Name: Supplementary Data 22

Description: The atomic coordinates of the optimized computational models of FEP + H_2O + •OH + •Cl under the crystal cell volume of 9*9*18 $Å^3$

File Name: Supplementary Data 23

Description: The atomic coordinates of the optimized computational models of FEP + H_2O + HOCl under the crystal cell volume of 9*9*18 $Å^3$

File Name: Supplementary Data 24

Description: The atomic coordinates of the optimized computational models of FEP + H_2O + •OH + •Cl the crystal cell volume of 10*10*20 $Å^3$

File Name: Supplementary Data 25

Description: The atomic coordinates of the optimized computational models of FEP + H_2O + HOCl the crystal cell volume of 10*10*20 $Å^3$
