# Supplementary material for: A triboelectric radical generation route to chlorine disinfectants from brine
Source: Nat Commun. 2026 Jul 7;17:8377. doi: 10.1038/s41467-026-75108-3 (PMC13473123; doi:10.1038/s41467-026-75108-3)
Supplement: Supplementary file 4 [file 41467_2026_75108_MOESM4_ESM.pdf]

Supplementary Data for

A triboelectric radical generation route to chlorine disinfectants from brine

Han Qian[1,2,†], Jianming Liu[1,2,†], Ning Wu[1,2,†], Shaoxin Li[1,2,3], Xinhong Song[1], Feiyao Yang[1], Morten Willatzen[1,2], Jiajia Shao[1,2], Vishnu Shankar[4], Daping Chu[5], Richard N. Zare[3,*], Zhong Lin Wang[1,*], Di Wei[1,5,*]

Affiliations:

[1]Beijing Institute of Nanoenergy and Nanosystems, Chinese Academy of Sciences, Beijing 101400, China

[2]School of Nanoscience and Engineering, University of Chinese Academy of Sciences, Beijing 100049, China

[3]Department of Chemistry, Stanford University, Stanford, CA, 94305, USA

[4]Program in Immunology, Stanford University School of Medicine, Stanford, CA, 94305, USA

[5]Centre for Photonic Devices and Sensors, University of Cambridge, UK

[†]These authors contributed equally: Han Qian, Jianming Liu, Ning Wu.

*Corresponding author. E-mail: zare@stanford.edu; zlwang@binn.cas.cn; weidi@binn.cas.cn

The PDF file includes:

Supplementary Data 1 | The atomic coordinates of the optimized computational models of FEP + H_2O + Cl^- under normal pressure

Supplementary Data 2 | The atomic coordinates of the optimized computational models of FEP + H_2O + •OH + Cl^- under normal pressure

Supplementary Data 3 | The atomic coordinates of the optimized computational models of FEP + H_2O + •$HOCl^-$ under normal pressure

Supplementary Data 4 | The atomic coordinates of the optimized computational models of FEP + H_2O + •$HOCl^-$ + H^+ under normal pressure

Supplementary Data 5 | The atomic coordinates of the optimized computational models of FEP + H_2O + •Cl + H_2O under normal pressure

Supplementary Data 6 | The atomic coordinates of the optimized computational models of FEP + H_2O + •OH + •Cl under normal pressure

Supplementary Data 7 | The atomic coordinates of the optimized computational models of FEP + H_2O + HOCl under normal pressure

Supplementary Data 8| The atomic coordinates of the optimized computational models of FEP + H_2O + Cl^- under high pressure

Supplementary Data 9 | The atomic coordinates of the optimized computational models of FEP + H_2O + •OH + Cl^- under high pressure

Supplementary Data 10 | The atomic coordinates of the optimized computational models of FEP + H_2O + •$HOCl^-$ under high pressure

Supplementary Data 11 | The atomic coordinates of the optimized computational models of FEP + H_2O + •$HOCl^-$ + H^+ under high pressure

Supplementary Data 12 | The atomic coordinates of the optimized computational models of FEP + H_2O + •Cl + H_2O under high pressure

Supplementary Data 13 | The atomic coordinates of the optimized computational models of FEP + H_2O + •OH + •Cl under high pressure

Supplementary Data 14 | The atomic coordinates of the optimized computational models of FEP + H_2O + HOCl under high pressure

Supplementary Data 15 | The atomic coordinates of the optimized computational models of FEP + H_2O + •Cl under normal pressure

Supplementary Data 16 | The atomic coordinates of the optimized computational models of FEP + H_2O + •Cl under high pressure

Supplementary Data 17 | The atomic coordinates of the optimized computational models of FEP + H_2O + K

Supplementary Data 18 | The atomic coordinates of the optimized computational models of FEP + H_2O + Na

Supplementary Data 19 | The atomic coordinates of the optimized computational models of FEP + H_2O + Li

Supplementary Data 20 | The atomic coordinates of the optimized computational

models of FEP + H_2O + •Cl + •Cl under high pressure

Supplementary Data 21 | The atomic coordinates of the optimized computational models of FEP + H_2O + Cl_2 under high pressure

Supplementary Data 22 | The atomic coordinates of the optimized computational models of FEP + H_2O + •OH + •Cl under the crystal cell volume of 9*9*18 $Å^3$

Supplementary Data 23 | The atomic coordinates of the optimized computational models of FEP + H_2O + HOCl under the crystal cell volume of 9*9*18 $Å^3$

Supplementary Data 24 | The atomic coordinates of the optimized computational models of FEP + H_2O + •OH + •Cl the crystal cell volume of 10*10*20 $Å^3$

Supplementary Data 25 | The atomic coordinates of the optimized computational models of FEP + H_2O + HOCl the crystal cell volume of 10*10*20 $Å^3$

Supplementary Data 1:

FEP+H_2O+Cl^-

```
    1.00000000000000
      15.0000000000000000     0.0000000000000000     0.0000000000000000
       0.0000000000000000    15.0000000000000000     0.0000000000000000
       0.0000000000000000     0.0000000000000000    25.0000000000000000
    C     F     O     H     Cl
      7    16     6    12     1
Direct
  0.4808686177820180  0.4049805420729674  0.2831561691656781
  0.4604235550429369  0.4744659051903412  0.3288555323744666
  0.5099761841880301  0.4641869313120908  0.3845337742546160
  0.4856017718253746  0.5456965189219939  0.4230824301236948
  0.4627017166290051  0.5188497694111299  0.4826297910797155
  0.4119600146227305  0.4065032006684693  0.2354678920540223
  0.6141797729186074  0.4522987350351164  0.3798415482247798
  0.4803597301700120  0.3205928118018721  0.3037196810137014
  0.4788211212857423  0.5575608759243882  0.3092792208364802
  0.3711933610410109  0.4675901099687640  0.3392351482509416
  0.5630103696549074  0.4224705703061833  0.2621665390046632
  0.4780986571992296  0.3868991419596557  0.4081950318423218
  0.5545904784006944  0.6049827570652231  0.4244038988850899
  0.5266350252699081  0.4683372401211436  0.5055271967833890
  0.4545669272921989  0.5950626808372971  0.5114459754654086
  0.3842591559999416  0.4747540554054596  0.4847935955963125
  0.4121319391233241  0.5911439892891600  0.4056241495488499
  0.3332495722767580  0.3717798617459377  0.2504184474035113
  0.4004544421159762  0.4900471046544024  0.2170094832398622
  0.6329825775802084  0.3716167321677262  0.3585193622943311
  0.4458489186613595  0.3553941558258185  0.1953904873115876
  0.6504785839812763  0.5163408296074791  0.3486623086573899
  0.6529216339463139  0.4554167781082989  0.4286391506567026
  0.0165689013993431  0.3979135377906491  0.3781061659168607
  0.1226567486455747  0.5817103992471842  0.2738039140691417
  0.1805495230626361  0.4932805909154686  0.1838434934308829
  0.9501664225268890  0.5333410230596327  0.3111225022924767
  0.8039133696445103  0.5260784192620878  0.2420121910633374
  0.1670935156603200  0.5373388522174202  0.3815324611847190
  0.9999923658241322  0.4466891333695002  0.4032822763303817
```

0.0803785476962967	0.4085541582851489	0.3736073112217521
0.0587555733968502	0.5703902912082087	0.2808931001335708
0.1500635045349753	0.5693336619107585	0.3088952478628499
0.1613866377499193	0.5255637304014358	0.2168452495220462
0.1324807019436638	0.4512181003792634	0.1776202833370147
0.9687553583408242	0.4742262421292963	0.3250650609079799
0.9497882963126081	0.5683261785986518	0.3449035588994097
0.8549057516348562	0.5301329791927941	0.2671846331067177
0.7529673139938057	0.5150707489590044	0.2650093925761742
0.2158974289809787	0.5620007258673965	0.4023206209103638
0.1112091858434483	0.5592832724258425	0.3998203486941878
0.9877765718007927	0.5845466323793009	0.4312353694726173

Supplementary Data 2:

FEP+H_2O+•OH + Cl^-

1.00000000000000		
15.0000000000000000	0.0000000000000000	0.0000000000000000
0.0000000000000000	15.0000000000000000	0.0000000000000000
0.0000000000000000	0.0000000000000000	25.0000000000000000

C F O H Cl

7 16 7 13 1

Direct

0.3640866445487098	0.4420332120872768	0.3449592303150040
0.4265888317894623	0.4932315769208955	0.3853294978926508
0.4435533264026009	0.4509859395783004	0.4426470433544951
0.5150925604970101	0.5091614223048697	0.4749615669006256
0.4900770850831320	0.5280948684518358	0.5352769871652047
0.3352509600121580	0.4981414364585486	0.2945372383574629
0.4716983555738931	0.3497883022483486	0.4410910493952753
0.2885800888806697	0.4147290671391285	0.3699070085457656
0.5080862004422140	0.5032655661427594	0.3607369084184538
0.3890825796502075	0.5745847561993755	0.3930382468188645
0.4088650330144795	0.3689336354466761	0.3256012095481555
0.3638512045818325	0.4539783177882354	0.4701447387466458
0.5958293235350963	0.4666857382775126	0.4742662172269443
0.4715246722602492	0.4523743682939889	0.5626972936580612
0.5615222491593029	0.5673391531558999	0.5588654540582537
0.4201444638758030	0.5838022960603699	0.5387056557735390
0.5270443237302712	0.5910347698935366	0.4526323484431776
0.2806884598484071	0.5652808647354012	0.3069767637634820
0.4084409314679932	0.5310871036701926	0.2688884476753742
0.4016018542080854	0.2990088235431106	0.4256502735944363
0.2926162755728732	0.4423530052689895	0.2601194245702965
0.5401946201602937	0.3352344876630636	0.4069477791764463
0.4962238900218739	0.3207352394025792	0.4903080016349540
0.7729885164616067	0.4651454143099704	0.3285185052887102
0.9061450533249170	0.5801931726112738	0.3501033441234234
0.1685670616107458	0.6728859227364233	0.3864495083504415
0.7678256793595092	0.5977335342647214	0.4290038785866095
0.0653446894299305	0.5401334130967335	0.3755251462673771
0.6495000840480538	0.4558121414872549	0.2518653241941248

0.7405794232282155	0.2970238633672455	0.2702406349134984
0.7922404902584116	0.4052493545740047	0.3181266001626237
0.8273425248140925	0.5032104031428790	0.3328710346652240
0.9116854931033588	0.6304886287068312	0.3259160304649804
0.9715556845667129	0.5634306327206763	0.3613193455789128
0.1372868802160161	0.7190718611182643	0.4065486584051650
0.7368670219445963	0.5471057301783410	0.4132234012029402
0.8220870660464453	0.6009993543037031	0.4068928473675600
0.0706336306601233	0.5172800396938877	0.4118985910786515
0.1066279348737844	0.5973903140689181	0.3781168935560315
0.5908476793543621	0.4669609814662836	0.2669485213529608
0.6928438878250072	0.4743378310164738	0.2803858803870314
0.6979905894330745	0.3445697811117639	0.2595993939090048
0.7064328941926237	0.2575820283385483	0.2932473346567004
0.1860137289317793	0.6117116329548948	0.4765607124544261

Supplementary Data 3:

FEP+H_2O+•$HOCl^{-}$

1.00000000000000					
15.0000000000000000	0.0000000000000000	0.0000000000000000			
0.0000000000000000	15.0000000000000000	0.0000000000000000			
0.0000000000000000	0.0000000000000000	25.0000000000000000			
C F O H Cl					
7 16 7 13 1					
Selective dynamics					
Direct					
0.4486677430225399	0.5448091623511075	0.2670261947022752	T	T	T
0.4339863629314067	0.6061112673452982	0.3175297608332741	T	T	T
0.4403956988874143	0.5629619505041461	0.3751744801649721	T	T	T
0.4310934205526493	0.6381337689888014	0.4201138041370385	T	T	T
0.3667882899123356	0.6138302072838542	0.4679822485449279	T	T	T
0.4178250972245579	0.5882439884447237	0.2126226702701572	T	T	T
0.5269473637185271	0.5046498315794210	0.3841505299616301	T	T	T
0.4018238967789571	0.4673609458859644	0.2724024933053713	T	T	T
0.4944474958166509	0.6742153475246226	0.3145067534567403	T	T	T
0.3496763590404370	0.6401216552019946	0.3124279781507511	T	T	T
0.5370286242615308	0.5259081657263351	0.2618518306688244	T	T	T
0.3681402864454526	0.5058749929206243	0.3803099553311000	T	T	T
0.5128572165526180	0.6574876176899238	0.4410866374474196	T	T	T
0.3875807048539883	0.5354743416874399	0.4913724303048426	T	T	T
0.3746760067573547	0.6791319184173332	0.5054410616153575	T	T	T
0.2812696569443602	0.6114485455697434	0.4514962048851956	T	T	T
0.3974726948554874	0.7159061204842914	0.3998223494921618	T	T	T
0.3284373719629343	0.5931048528063272	0.2089456231821529	T	T	T
0.4526119014192318	0.6710098496469499	0.2064784446377459	T	T	T
0.5208990346586475	0.4298348347018432	0.3542834493292162	T	T	T
0.4480477859026095	0.5363485641379881	0.1719464769332717	T	T	T
0.6004756611923838	0.5503068776906588	0.3705028002962081	T	T	T
0.5327209961567689	0.4793905163404659	0.4360317435557022	T	T	T
0.1153747886617893	0.3615385947700176	0.2103649515108899	T	T	T
0.0942599850358476	0.5029105926025679	0.2841042926465605	T	T	T
0.8193423375998066	0.5109184443808914	0.3769623278595233	F	F	F
0.8827550510448461	0.3187839710308253	0.2918438366400764	T	T	T
0.9114468045663529	0.5018959313584688	0.2942317564348897	T	T	T

0.0557554683776971	0.2650106396294234	0.2919198595348504	T	T	T
0.1429775525937842	0.3883736914131788	0.3613508823788010	T	T	T
0.0917717880999973	0.3200272173504004	0.2393748037330781	T	T	T
0.1772909274139849	0.3442683062994263	0.2057934511078230	T	T	T
0.1023422873701989	0.4605146393705652	0.2540680756531017	T	T	T
0.0283510553524735	0.5077954088277750	0.2884827019591185	T	T	T
0.8545923375998115	0.5034824443808859	0.4094653278595217	F	F	F
0.8436588618337737	0.3165364684814236	0.3235002901875532	T	T	T
0.8920275176318881	0.3838997120588843	0.2871500094530859	T	T	T
0.8736886573186595	0.5051050716044259	0.3312092277014855	T	T	T
0.8794621551208195	0.5393812314957742	0.2690626772581481	T	T	T
0.9906010925973732	0.2793281909582390	0.2949226799965601	T	T	T
0.0846731019117755	0.2991672888157808	0.3209673708165468	T	T	T
0.2076869456749435	0.3873933683274302	0.3620443896576901	T	T	T
0.1269553221879534	0.4364573101446041	0.3346667561468735	T	T	T
0.7520715115382188	0.3636233593564739	0.3925572373728201	F	F	F

Supplementary Data 4:

FEP+H_2O+•$HOCl^{-}$ + H^{+}

```
     1.00000000000000
      15.0000000000000000     0.0000000000000000     0.0000000000000000
       0.0000000000000000    15.0000000000000000     0.0000000000000000
       0.0000000000000000     0.0000000000000000    25.0000000000000000
     C     F     O     H     Cl
       7    16     7    14     1
Selective dynamics
Direct
  0.4098707622028570  0.4295185801962660  0.3474856545614315   T   T   T
  0.4242425734110924  0.4837733018353063  0.4008123598715656   T   T   T
  0.3520992472354365  0.4752161300872970  0.4468947479878338   T   T   T
  0.3835521222817277  0.5292172532365385  0.4988242887384189   T   T   T
  0.3085483054991798  0.5874567361774581  0.5261867928745545   T   T   T
  0.4713968780303901  0.4596166657747958  0.2996452901916129   T   T   T
  0.3273414178717289  0.3760401766933654  0.4615015626900600   T   T   T
  0.3239305118940692  0.4380146617500245  0.3304240686822126   T   T   T
  0.5052331300753750  0.4601442796522548  0.4210856591579528   T   T   T
  0.4258132851166678  0.5716647212852237  0.3859617270900699   T   T   T
  0.4278194318515887  0.3415277128274639  0.3566292195226950   T   T   T
  0.2744721480068577  0.5141587765439795  0.4280374044454973   T   T   T
  0.4156061147963896  0.4707951503454524  0.5360643373359224   T   T   T
  0.2344648134883727  0.5398569753782425  0.5373497717954550   T   T   T
  0.3418730225572233  0.6197770567857077  0.5725545955803781   T   T   T
  0.2863611879019459  0.6569973527205946  0.4944791531503441   T   T   T
  0.4503430498417913  0.5876282395400310  0.4871638898924999   T   T   T
  0.4466821354274870  0.5395744077500524  0.2801380416090069   T   T   T
  0.5578285240070713  0.4627596864992753  0.3143337375918449   T   T   T
  0.2803128964888799  0.3389876048352249  0.4206342260965741   T   T   T
  0.4627194166738349  0.3983590945112212  0.2597544897049531   T   T   T
  0.4008083228455774  0.3261880429299353  0.4702481719701410   T   T   T
  0.2747604057065374  0.3724980872354351  0.5053061930048770   T   T   T
  0.2196482357883980  0.1804234917047438  0.3270753227272110   T   T   T
  0.1396198802820692  0.2778729052159647  0.2464108288658409   T   T   T
  0.8191116983612048  0.6883325295208707  0.4330841944026318   F   F   F
  0.9163559937890184  0.7163511737469790  0.3865735378581334   T   T   T
  0.3140338524326793  0.2246830845452408  0.2361507575038857   T   T   T
```

0.6443008711185314	0.7219088195911709	0.3619851063724724	T	T	T
0.7993707771355005	0.7673413298578708	0.3212805312077975	T	T	T
0.2237267433036010	0.2107458166987470	0.3613210081400808	T	T	T
0.1748605607450742	0.2140480991885028	0.3059645238904096	T	T	T
0.2007113890077790	0.2679063988297798	0.2322419210961328	T	T	T
0.0996145626550415	0.2494424775171566	0.2210659674756172	T	T	T
0.8502432626204595	0.7253616985205866	0.4613972017898078	F	F	F
0.8696025968127543	0.7357142211003165	0.3568454238178873	T	T	T
0.9282523113827708	0.6529581661990349	0.3811182203543027	T	T	T
0.3637200898540129	0.2658200224620586	0.2396198394681406	T	T	T
0.3003527447977774	0.2038587130504297	0.2729185521421584	T	T	T
0.6546494072203757	0.6951578363429576	0.3978851567027846	T	T	T
0.8106713736251763	0.5619463598552912	0.5579149418109353	F	F	F
0.7387560875546768	0.7508731615562141	0.3366722606089994	T	T	T
0.8002001103081255	0.7470065220511160	0.2844167741540033	T	T	T
0.6033655884541105	0.7710193792860593	0.3683588936791509	T	T	T
0.6786049369361891	0.6452909836261824	0.4767310415912434	F	F	F

Supplementary Data 5:

FEP+H_2O+•Cl+ H_2O

```
   1.00000000000000
    15.0000000000000000     0.0000000000000000     0.0000000000000000
     0.0000000000000000    15.0000000000000000     0.0000000000000000
     0.0000000000000000     0.0000000000000000    25.0000000000000000
   C    F    O    H    Cl
     7    16     7    14     1
Direct
  0.4865526639057762  0.4214558442874647  0.3420836845940372
  0.4978206057004186  0.4984938649493387  0.3844511139445075
  0.4969708512669442  0.4737286097863276  0.4460910676034686
  0.5176821126595712  0.5600050658454023  0.4815929638971355
  0.4552506591615213  0.5724504805206223  0.5319696276874808
  0.4652542429161031  0.4548246053234948  0.2836086988945112
  0.5608546672249659  0.3942196149792636  0.4614994392000469
  0.4182762504618154  0.3666414209379454  0.3569690442075881
  0.5758454125901551  0.5416307508102136  0.3735669138898672
  0.4276956689751970  0.5555625939965552  0.3763353498651654
  0.5636005423457249  0.3735570332271934  0.3395157499606682
  0.4111855864122020  0.4455544912118512  0.4582959798068060
  0.6035850567122054  0.5574196527596982  0.4992315263362883
  0.4513967976399158  0.4994774666386448  0.5632677570188065
  0.4868611387035244  0.6411323924698138  0.5610865966076446
  0.3708296197229402  0.5934045322352048  0.5159463796420027
  0.5076945475004422  0.6369994153475105  0.4529269019527931
  0.3814158246423176  0.4869342151070271  0.2800632029341301
  0.5232079253920716  0.5188484326344351  0.2681454669473098
  0.5283765507505009  0.3170924324037188  0.4413156850559972
  0.4728924107362786  0.3845637931417940  0.2500023531037456
  0.6439131740046733  0.4074079793186129  0.4424896465570853
  0.5649899138255989  0.3844573511352323  0.5153157712626495
  0.1763706568218524  0.5101917781606176  0.5352569246206471
  0.0157777089503170  0.7446212616789580  0.4120221159403172
  0.8569825561170390  0.7982648951541123  0.4502293468941332
  0.0434277330161663  0.5972410949185090  0.4787342081122574
  0.7443395506089558  0.7308006489184677  0.3775059920413071
  0.9743842138870349  0.5781162305945031  0.3752179045678474
```

0.8003943188808762	0.5664631979837225	0.4007833204118540
0.1808431395722323	0.4463230391613351	0.5291940233619172
0.2366091484634651	0.5331465114190550	0.5307205577308380
0.0019250326939400	0.6975776270078171	0.3853100617236223
0.0425380450964212	0.7086649320800333	0.4410133338219921
0.8508658935692210	0.7615188488447799	0.4826530369603361
0.9184549565343403	0.7842870702713753	0.4362723574833680
0.0896215413127166	0.5663650149150428	0.5001368038133478
0.9885825402170525	0.5992482444572502	0.5004225962438156
0.6832477328428723	0.7494481862013629	0.3839188026434073
0.7823377642694211	0.7638828862822038	0.4040043227444130
0.9091309688986939	0.5666886792775045	0.3811227835585811
0.0025942399637842	0.5646489525851663	0.4101337123229385
0.7727160969169660	0.6252625670837091	0.3903427263246044
0.7982755194442746	0.5658685928224685	0.4401795532021495
0.8439284186715028	0.6219877011146195	0.5199045945065492

Supplementary Data 6:

FEP+H_2O+•OH + •Cl

```
    1.00000000000000
     15.0000000000000000     0.0000000000000000     0.0000000000000000
      0.0000000000000000    15.0000000000000000     0.0000000000000000
      0.0000000000000000     0.0000000000000000    25.0000000000000000
    C     F     O     H     Cl
      7    16     7    13     1
Selective dynamics
Direct
  0.3706708577602029  0.5385493638883148  0.5759314029430769   T   T   T
  0.3739817693959679  0.5930279759249473  0.6298240344916053   T   T   T
  0.3420867127583733  0.5462062550343063  0.6833167595918326   T   T   T
  0.3591340152762186  0.6102502459242558  0.7334540726327603   T   T   T
  0.2796020886827861  0.6158248183136052  0.7747674238833469   T   T   T
  0.3804054029463231  0.5978371455460234  0.5240989181161808   T   T   T
  0.3844959701767436  0.4512856241096515  0.6927773749279282   T   T   T
  0.2905888017966518  0.4951748081855882  0.5714809194786810   T   T   T
  0.4596253004010507  0.6214108302697383  0.6366941225291143   T   T   T
  0.3194337805537941  0.6652816073246441  0.6222315337985620   T   T   T
  0.4376092593068606  0.4776672207569348  0.5753653474894804   T   T   T
  0.2510095886608497  0.5329312843170015  0.6787482926901447   T   T   T
  0.4334223725896464  0.5827053142823360  0.7603738909386166   T   T   T
  0.2527568996039378  0.5348441203544567  0.7918402364495591   T   T   T
  0.3083174545309717  0.6630409027364479  0.8175935003033103   T   T   T
  0.2094896572380662  0.6590622732901323  0.7529156493255513   T   T   T
  0.3736972287127855  0.6962673876364178  0.7178528497781187   T   T   T
  0.3052380602794145  0.6452183252024639  0.5147646105732164   T   T   T
  0.4499780796048753  0.6537507340044115  0.5272761199400549   T   T   T
  0.3508209677913001  0.3926540621192771  0.6568809077240800   T   T   T
  0.3929476982138951  0.5421702986072958  0.4816104016587346   T   T   T
  0.4740588275524402  0.4534801861870790  0.6875983683837106   T   T   T
  0.3640045763418698  0.4200933960871112  0.7420564264905352   T   T   T
  0.9383470264266904  0.6029025559460308  0.3522304640368787   T   T   T
  0.7982508333934604  0.7078676001925572  0.3609728762392663   T   T   T
  0.6645187781929707  0.5456210352766533  0.4056363848295206   F   F   F
  0.1087053527899897  0.6403342486855266  0.3303264870797804   T   T   T
  0.2696841943220605  0.4050673383499230  0.4224464733866142   T   T   T
```

0.6938002768180098	0.5827762417662470	0.3309148600552234	T	T	T
0.1992075775383597	0.4857972426183466	0.3334751983371689	T	T	T
0.9242349583960561	0.5562818838891346	0.3266299854693671	T	T	T
0.0020047477290817	0.6197599437987691	0.3448122600657726	T	T	T
0.8565843010827123	0.6742667615961295	0.3582076446493758	T	T	T
0.7893981383933237	0.7201351870473260	0.3988032624324447	T	T	T
0.7275487781929755	0.5276110352766565	0.4097863848295162	F	F	F
0.1461254390613789	0.5854146588171147	0.3330450897672179	T	T	T
0.1393353670971180	0.6862213809740041	0.3505976561624635	T	T	T
0.2287679159001712	0.3785209509804375	0.4480408791955028	T	T	T
0.3133358737668743	0.4375903824248439	0.4436790402580755	T	T	T
0.6317635469115856	0.6014552986384558	0.3278941128933468	T	T	T
0.7303538792010684	0.6383117439623405	0.3429992919326139	T	T	T
0.2375832400093572	0.4692300883184188	0.3038560223858298	T	T	T
0.2249951236138799	0.4575141132442155	0.3658342182294732	T	T	T
0.6365887781929729	0.4995810352766554	0.4985663848295161	F	F	F

Supplementary Data 7:

FEP+H_2O+HOCl

1.00000000000000

15.0000000000000000	0.0000000000000000	0.0000000000000000
0.0000000000000000	15.0000000000000000	0.0000000000000000
0.0000000000000000	0.0000000000000000	25.0000000000000000

C	F	O	H	Cl
7	16	6	12	1

Direct

0.4543142832890434	0.4340780833854078	0.3449729491474804
0.4496175231472052	0.5092348725801159	0.3890209632594449
0.4823953607021453	0.4871021879779937	0.4477804694290628
0.4793587664563747	0.5745158692973507	0.4843110530587179
0.4399427933910660	0.5603843421731722	0.5423395199497718
0.3985820675827673	0.4548794436833385	0.2928994801967625
0.5773431060108249	0.4417236425440127	0.4499080213804501
0.4228970350932082	0.3556574744462731	0.3649466080663383
0.4965057058643115	0.5808682765802733	0.3705331577568637
0.3610131960714891	0.5317363798071812	0.3927867528419250
0.5408134302070249	0.4232784426392875	0.3292514500441563
0.4227486031608780	0.4260409586759934	0.4688000237244895
0.5628046998554554	0.6093311572588926	0.4890258885808402
0.4800243316526604	0.4932728034841476	0.5688651278287283
0.4526316779112804	0.6368398072893007	0.5700544459244812
0.3514152237197839	0.5441698432457145	0.5395821495237280
0.4266332222092241	0.6394397921769138	0.4619792230811294
0.3100102834536444	0.4480034964774536	0.3021975107151144
0.4164443632005040	0.5374266256871731	0.2740601543349193
0.5716514531951276	0.3579326675820624	0.4302374125549362
0.4209296193352015	0.3944548772561445	0.2550578253618290
0.6371510011689652	0.4883004620609582	0.4206468576662311
0.6069002393164796	0.4358260745086681	0.5007565799605164
0.7987276737839424	0.3403161281696656	0.4426537680433810
0.1866600852596800	0.6346443328851697	0.4499609645782683
0.9117378165108418	0.5503190839027637	0.3750163720443435
0.1065923759677991	0.4710081521357096	0.4728852127240509
0.8312310359801059	0.4010889428565240	0.3430878972111616
0.0897918605579743	0.5208261359903193	0.3727257529961555
0.8294832314494324	0.3923258595172761	0.4587106701593728

0.8305925804122876	0.2881506769711120	0.4555694985828445
0.1668529534656779	0.6187691273169053	0.4138164420799741
0.2506028449892941	0.6441077330212871	0.4472610005061935
0.9778128778976423	0.5381117585805436	0.3729865248921311
0.8807979280465098	0.4936094811339367	0.3627871796937217
0.0444515943443256	0.4830794531067446	0.4832333133843293
0.1388446277204627	0.5274875933829151	0.4792565643489826
0.8148176466362337	0.3704084747704806	0.3772508818150995
0.7756988695514092	0.4107740857717303	0.3239547691347957
0.1122843396456791	0.4809591798949045	0.3452225832840564
0.0983436830108253	0.4898002471506643	0.4085414162009733
0.8969881497752040	0.5194059226235167	0.4692854979322243

Supplementary Data 8:

FEP+H_2O+Cl^-

```
   1.00000000000000
     8.0000000000000000    0.0000000000000000    0.0000000000000000
     0.0000000000000000    8.0000000000000000    0.0000000000000000
     0.0000000000000000    0.0000000000000000   15.0000000000000000
   C    F    O    H    Cl
     7    16    6    12    1
Direct
  0.2893233729564021  0.3198718031061665  0.3057793783029279
  0.4620139139264982  0.3940736796657996  0.3375321214956824
  0.4831633008098981  0.4487147553669793  0.4383037537250091
  0.6714571978242209  0.5049977746623496  0.4573664564088782
  0.6906127085139030  0.6689634272707882  0.5153476214153028
  0.2664596528994160  0.3139392655188109  0.2012320427991382
  0.4237536310099942  0.3122746637096558  0.5076485270287607
  0.1608425185128879  0.4135244316533878  0.3389353020897518
  0.5829191206172317  0.2789348521546176  0.3177986196323112
  0.4873090956743794  0.5344536053926530  0.2867517425200992
  0.2746755117433858  0.1598345952276958  0.3364211470889431
  0.3808450755855590  0.5865881884890092  0.4505940820477156
  0.7548890253505036  0.3784917833630903  0.4988584143815636
  0.6022182695481777  0.6631514041760235  0.5908174287420251
  0.8544889485600037  0.6871790352079223  0.5347340463639466
  0.6415476599680217  0.8036793236332950  0.4664616342996086
  0.7558324766418796  0.5380705851117263  0.3796869163887286
  0.2418533439241941  0.4686403299820526  0.1682600040729852
  0.4006702234017957  0.2438658225719267  0.1620026131597089
  0.2558408252166408  0.2979989189542385  0.5040282074065319
  0.1295933352492294  0.2200248765232087  0.1838278069149934
  0.4924518118066115  0.1612852988537835  0.4879290886295412
  0.4670688358365831  0.3549739687960790  0.5909076472456295
  0.1474257731099687  0.3609458003971318  0.7144606549017113
  0.6252326024261188  0.1534004652683486  0.8555666831741936
  0.7011334650920322  0.8146780059193962  0.7786204719404911
  0.4058329856737631  0.0182456000079087  0.9781345075758134
  0.9131867014803671  0.3265341514339838  0.8691044219048670
  0.2108050788176871  0.2916376302711559  0.9669679992750970
  0.0623624090943306  0.3394463044349990  0.7601095228154143
```

0.2405784037619941	0.4101341828982196	0.7483875478913199
0.5585101092058269	0.1032372734706587	0.9049916929205388
0.5618322696154382	0.2563844759773273	0.8401773243492915
0.6643601397619924	0.9257775518668551	0.7979615208930828
0.6150445751972123	0.7359035523100241	0.7989382118529822
0.3197178101705429	0.1094512845187918	0.9724464607194460
0.3609747065977807	0.9213516192876102	0.9470867407646810
0.8652636826816207	0.4384864160410250	0.8687932300673240
0.8130401056820942	0.2507457641880569	0.8687664270816241
0.1018478049699999	0.2961230131916242	0.9356807735879150
0.2843311567006834	0.3669691710390139	0.9316393687922825
0.4469703683831757	0.5067254140866462	0.8439317603321477

Supplementary Data 9:

FEP+H_2O+•OH + Cl^{-}

1.00000000000000

8.0000000000000000	0.0000000000000000	0.0000000000000000
0.0000000000000000	8.0000000000000000	0.0000000000000000
0.0000000000000000	0.0000000000000000	15.0000000000000000

C	F	O	H	Cl
7	16	7	13	1

Direct

0.2672444142088512	0.2748483193104390	0.5207538763238718
0.4532863148216502	0.3398033689495358	0.5175131906529005
0.5065342469702960	0.4864057356924406	0.5826366698812949
0.7021887562092757	0.5222843230405514	0.5743586820625274
0.7533569101324262	0.7133020469915464	0.5725642959974758
0.2152335634513065	0.1623115809122632	0.4390269341959461
0.4549732098783886	0.4561469612656265	0.6830121160608328
0.1595541054226857	0.4071400479702896	0.5221979578838482
0.5556479162894377	0.2058287076010272	0.5332190490555386
0.4779551830307468	0.3954480211623280	0.4324428015739203
0.2446975339910019	0.1809453549843678	0.5956666667716866
0.4224620351803276	0.6284281269998055	0.5549344883130438
0.7843878894307521	0.4477792390957988	0.6436566076116339
0.6895565488396639	0.8004506860139087	0.6411909665006524
0.9228462239591629	0.7203492344283527	0.5774673025756376
0.7052222478598511	0.7844901735000651	0.4951837832991861
0.7669999858074538	0.4569867486883966	0.4975315321224503
0.1997254220999135	0.2526271804186459	0.3639108058172922
0.3270621841868383	0.0382522136321954	0.4258276293692976
0.2864670763614447	0.4686842560868565	0.6905865981862491
0.0645110385101444	0.0925553742388986	0.4580481152131109
0.5025621294112111	0.3024963942700936	0.7107281431703593
0.5232591015129585	0.5720494984899943	0.7364679588586774
0.4691675848673815	0.7381538923977133	0.3203091454477948
0.4528527167091522	0.3358308879460141	0.9719499421255036
0.5661711564938582	0.6531319200089409	0.9431840058950404
0.6870578995749889	0.5572576793611131	0.2132095757331984
0.5110534626871578	0.7414817286989493	0.1082337056301301
0.4330791408943789	0.3194295835205080	0.1562957929568828

0.2263102949802319	0.5857394489526732	0.1973187078191759
0.4700445000432910	0.8399795134605406	0.2847143320011201
0.5606934601802563	0.6712707297193287	0.2932593721787997
0.5012232647530860	0.4440005293978136	0.9505008244623565
0.5056920712239643	0.2437908400905704	0.9398147109344188
0.6838399098943821	0.6728059792807110	0.9297869307437779
0.6308552394674992	0.6316179268285891	0.1654265953084238
0.8074287196513693	0.5672048485344385	0.2058055305713339
0.5294402948263707	0.7169185446921150	0.0420339738044710
0.3982250207534714	0.7002235160497461	0.1231508561177505
0.5370879384296330	0.3739101660871579	0.1755387890296742
0.4414430216256882	0.3163565022769186	0.0900280047334242
0.2870704763441032	0.4799234181178209	0.1830581710183250
0.2871189746671949	0.6298935385758431	0.2492550641962423
0.5351608143667350	0.9250152122590583	0.8679097977947227

Supplementary Data 10:

FEP+H_2O+•$HOCl^-$

```
   1.00000000000000
     8.0000000000000000    0.0000000000000000    0.0000000000000000
     0.0000000000000000    8.0000000000000000    0.0000000000000000
     0.0000000000000000    0.0000000000000000   15.0000000000000000
   C    F    O    H    Cl
     7   16     7    13     1
Selective dynamics
Direct
  0.4673716999657458  0.4526578571263968  0.4195439452463390   T   T   T
  0.6172551282461498  0.5736514661467140  0.4417171588069024   T   T   T
  0.6896858249170492  0.5753520557967562  0.5402226247920865   T   T   T
  0.8495194123664831  0.6949463373884583  0.5459446898611660   T   T   T
  0.8557479230435021  0.8123188140699185  0.6307061544985970   T   T   T
  0.3764123917322593  0.4893714275744969  0.3282278033702823   T   T   T
  0.7308181837493422  0.3963850170390018  0.5783319994601399   T   T   T
  0.3491021636156972  0.4627836008634105  0.4851665841826147   T   T   T
  0.7444037474337364  0.5379195205910250  0.3846318040465349   T   T   T
  0.5589814164487166  0.7320990230211564  0.4256140109757212   T   T   T
  0.5258723794197238  0.2923196105352162  0.4155979001364400   T   T   T
  0.5669498877276951  0.6414635193389202  0.5948579503786604   T   T   T
  0.9919235504815944  0.6002651039298357  0.5452161553858090   T   T   T
  0.8385796263455987  0.7285141610767027  0.7074652232964809   T   T   T
  0.0062227812875832  0.8904084601031954  0.6304206225197575   T   T   T
  0.7351294163488120  0.9308407544877468  0.6248910992490337   T   T   T
  0.8581819898769538  0.8006186895777603  0.4747251697122650   T   T   T
  0.2875491016845899  0.6322794476752875  0.3329962242334901   T   T   T
  0.4875283328917206  0.4990048644419147  0.2612947622712909   T   T   T
  0.5867237540005306  0.3123995853617103  0.5943204115225242   T   T   T
  0.2697301698462766  0.3612113644532187  0.3126303550301373   T   T   T
  0.8237419739491045  0.3064426060704548  0.5200126657330699   T   T   T
  0.8144378923906501  0.4062352132913958  0.6562008937070303   T   T   T
  0.7276307378785276  0.5802772314595899  0.9275225717770255   T   T   T
  0.5811859719639259  0.7008678870595304  0.0748488112920924   T   T   T
  0.0678950769132456  0.4806394419687265  0.9457568383036801   T   T   T
  0.8882611344500404  0.7222436500204168  0.1976495283567772   T   T   T
  0.2028444830963778  0.6079965141320471  0.1089234515687636   T   T   T
```

0.4800488029623153	0.4292663644514505	0.8213992120761731	F	F	F
0.2938569161358018	0.2287829530091940	0.9227883934677479	T	T	T
0.8380838434230427	0.5256654957839828	0.9296492848237605	T	T	T
0.6561028967550158	0.5229551752536210	0.8834898221842309	T	T	T
0.5639904869379464	0.8166313612208995	0.0574160990636372	T	T	T
0.6316551588545281	0.6483553104615041	0.0200054098354272	T	T	T
0.1298222382601963	0.3732178560092692	0.9389190901845765	T	T	T
0.9916633382481450	0.6928718118043236	0.1659902272773337	T	T	T
0.7973014142719137	0.7088411468783420	0.1543808504842161	T	T	T
0.1593665816106622	0.5599190528980955	0.0527867541435327	T	T	T
0.3201412078124832	0.6352307995626560	0.0966484058661834	T	T	T
0.1314422762051919	0.5572591296823148	0.9066040471993944	T	T	T
0.4338348029623162	0.4110113644514470	0.7621932120761770	F	F	F
0.3696623312499838	0.2998884498232144	0.8838591680543075	T	T	T
0.2905270457397506	0.1180695690855202	0.8962606755688540	T	T	T
0.3232518029623179	0.6934133644514517	0.8293672120761784	F	F	F

Supplementary Data 11:

FEP+H_2O+•$HOCl^{-}$ + H^{+}

```
   1.00000000000000
     8.0000000000000000    0.0000000000000000    0.0000000000000000
     0.0000000000000000    8.0000000000000000    0.0000000000000000
     0.0000000000000000    0.0000000000000000   15.0000000000000000
   C    F    O    H    Cl
     7    16    7    14    1
Selective dynamics
Direct
  0.6212125669497147  0.5810411379145932  0.3439361430421368   T   T   T
  0.6026173696031139  0.6113483630123658  0.4473606137285779   T   T   T
  0.4250953079984790  0.5843960177584810  0.4921957645478174   T   T   T
  0.4379885584073080  0.6066537809702218  0.5979857663799912   T   T   T
  0.2929219453251963  0.7128987910908082  0.6417693498621354   T   T   T
  0.7887579872293539  0.6505208432848130  0.3024458278619959   T   T   T
  0.3398540662825790  0.4127057756840919  0.4672602139894401   T   T   T
  0.4927615676391678  0.6556679433803541  0.2996338765264352   T   T   T
  0.7152251587284004  0.5100825586968230  0.4897162555880108   T   T   T
  0.6453876235400540  0.7749924814385908  0.4613746305274615   T   T   T
  0.6187617392471053  0.4132198305862574  0.3276270257714848   T   T   T
  0.3209578040343734  0.7084969648894428  0.4597545447993033   T   T   T
  0.4397110430483693  0.4528354470461108  0.6378107705758849   T   T   T
  0.1396048542400165  0.6566201069620310  0.6191805608791013   T   T   T
  0.3095816731230328  0.7025399212766377  0.7312039295750851   T   T   T
  0.3065731455122508  0.8752316032950003  0.6175619754715278   T   T   T
  0.5820251305604129  0.6861295465359606  0.6221508742549873   T   T   T
  0.7880839653566174  0.8197166231514446  0.2987672734380385   T   T   T
  0.9230953995620306  0.6004322279773295  0.3503567323505634   T   T   T
  0.2923511396323560  0.4147010275775520  0.3808041368289937   T   T   T
  0.8031325359115540  0.5910310242620057  0.2186671374182528   T   T   T
  0.4453587865175524  0.2828827013970582  0.4803477463831031   T   T   T
  0.2006061305652154  0.3871032460938700  0.5166772937155463   T   T   T
  0.5102559117268382  0.2491413295342301  0.0464758155963370   T   T   T
  0.4653384895997160  0.3271117327642122  0.8741310664756460   T   T   T
  0.3663046042301511  0.9861285818908010  0.1119028998401574   T   T   T
  0.6140710000000027  0.6195099999999982  0.9047270000000012   F   F   F
  0.0296629997557085  0.0062060181298455  0.1134095105009651   T   T   T
```

0.4217412723538511	0.6622973504231181	0.0395566153848357	T	T	T
0.8541698864467023	0.2673432249973627	0.0503997921401258	T	T	T
0.4662729090412220	0.3473539621461712	0.0770893447814106	T	T	T
0.4528981706967384	0.1455854192355787	0.0729797172285828	T	T	T
0.5112345997110329	0.2430344187095957	0.8343103865910232	T	T	T
0.4768602916862634	0.2813472253849936	0.9361968354890065	T	T	T
0.3942260664849861	0.8755269493518696	0.0863807890209530	T	T	T
0.9748233012164331	0.9804624576434022	0.1695200595122497	T	T	T
0.5724879999999999	0.5028470000000027	0.8841759999999965	F	F	F
0.2415595773954968	0.9939557252714971	0.1124464602930695	T	T	T
0.9668524287636578	0.1033661591437656	0.0878133576398784	T	T	T
0.3193285528591161	0.6451403281313672	0.0056483606195904	T	T	T
0.5163183563188940	0.6483182890735996	0.9949644588884609	T	T	T
0.7302790377036185	0.2570885381558649	0.0501560976793259	T	T	T
0.8825589845402795	0.3313968973214418	0.9971460348954558	T	T	T
0.8283249999999995	0.3651249999999990	0.6971640000000008	F	F	F
0.9020370521546326	0.5358059480251711	0.8683627964691922	F	F	F

Supplementary Data 12:

FEP+H_2O+•Cl+ H_2O

```
   1.00000000000000
     8.0000000000000000    0.0000000000000000    0.0000000000000000
     0.0000000000000000    8.0000000000000000    0.0000000000000000
     0.0000000000000000    0.0000000000000000   15.0000000000000000
   C     F     O     H     Cl
     7    16     7    14     1
Direct
  0.5534955104338902  0.4642061477391750  0.2444832699890177
  0.5974626683092658  0.5740373486908266  0.3286059780080565
  0.5368388086480492  0.5130236419535346  0.4238586517928392
  0.6076086463347402  0.6333312222783994  0.5003578204863557
  0.4754941194204037  0.6858823083608184  0.5735432880150639
  0.5782061039215177  0.5557778634591941  0.1521788084558919
  0.5805662000400053  0.3248083496124212  0.4446769499640434
  0.3907267719008903  0.4144294737333240  0.2484739552691549
  0.7676048736833518  0.5925781522721423  0.3309948346732664
  0.5250756864816579  0.7267525874321592  0.3142681598176040
  0.6544448131648556  0.3263954065084527  0.2438954178392124
  0.3644164473625101  0.5239875436382112  0.4243552484489846
  0.7397588185483940  0.5594555642448723  0.5416100542626446
  0.4000012556792285  0.5526152061976252  0.6105084504659134
  0.5575102701826221  0.7711407829411662  0.6379352874981039
  0.3590170468922412  0.7879666734126828  0.5373378700585110
  0.6647984334034324  0.7819412860512474  0.4655765239438623
  0.4596908886931819  0.6729450403147000  0.1393274162956724
  0.7307812810118617  0.6285927540271325  0.1478070360200922
  0.4868890152062684  0.2225080449710797  0.3930833549325775
  0.5663488873977950  0.4403488855541593  0.0868131951262237
  0.7440876111801045  0.2923078898959031  0.4291047712364605
  0.5459656056292774  0.2864579288396667  0.5305804093740668
  0.9082597437512994  0.1264051262158541  0.6969682342225115
  0.5411378685106751  0.3547375600272297  0.8315709608685996
  0.0309901540402902  0.6266490959243958  0.8665814541596231
  0.0169076005912168  0.9653225988317813  0.4192338748942606
  0.8847867318678787  0.3241020458005243  0.8331653898325637
  0.1219518276753875  0.1734031898612470  0.5563688453614076
```

0.3597986847361955	0.6250232766820188	0.8772898617824924
0.9362668291034293	0.0105840575161460	0.7141492387569544
0.9862442482160154	0.1520369746204503	0.6469013942834293
0.6654714583224093	0.3500647401432143	0.8336207700317126
0.5103446756338564	0.2997737155385614	0.7762555188006111
0.9848248021600299	0.5173996625896659	0.8450969521653698
0.1579744194222525	0.6155311115957619	0.8689639912309708
0.9645458602116234	0.0209444654868842	0.3687930420904011
0.9360422071525284	0.8822840061801325	0.4390289944089906
0.9154404797601340	0.2561112929659008	0.8843832282083073
0.9035542248303855	0.2486638292661283	0.7785785885713682
0.0851013281131423	0.0998858407142409	0.5069305337159974
0.2334265037756180	0.1343527313810895	0.5715222333257208
0.3930663739482776	0.7140220045972626	0.8368774554093948
0.4291650066155382	0.5261002294092804	0.8599105868655118
0.0442892080362442	0.7568923425233177	0.7236420990401200

Supplementary Data 13:

FEP+H_2O+•OH + •Cl

1.00000000000000

8.0000000000000000	0.0000000000000000	0.0000000000000000
0.0000000000000000	8.0000000000000000	0.0000000000000000
0.0000000000000000	0.0000000000000000	15.0000000000000000

C	F	O	H	Cl
7	16	7	13	1

Selective dynamics

Direct

0.4345028682467003	0.3336942859442941	0.3496751938334126	T	T	T
0.5868441369952457	0.3663526090387191	0.4141751467936002	T	T	T
0.5945270240788249	0.5377640888180836	0.4670700812561514	T	T	T
0.7506328893217556	0.5391001030213159	0.5336872162224273	T	T	T
0.8554247892164634	0.7067060491989269	0.5339672548012024	T	T	T
0.4592744573156170	0.1810145602737996	0.2843331520201577	T	T	T
0.4276155888538726	0.5831218572323514	0.5175008222425549	T	T	T
0.4067469524864691	0.4711128498881568	0.2979926212411902	T	T	T
0.5915348763041532	0.2384880686248644	0.4745032364516591	T	T	T
0.7267080065363334	0.3599947613163517	0.3615428737287640	T	T	T
0.2952684433454946	0.3003370451738760	0.3989774507071718	T	T	T
0.6192012285048896	0.6627897295949912	0.4044906908702648	T	T	T
0.6968447880313086	0.5100139373988076	0.6187216109764357	T	T	T
0.7599820237250295	0.8426523866265277	0.5494065391058220	T	T	T
0.9706958149686086	0.6948987248317646	0.5997202073344055	T	T	T
0.9362319573377279	0.7263373123987467	0.4555526169801781	T	T	T
0.8630693961307665	0.4165847769004621	0.5132306663219902	T	T	T
0.5739155303830096	0.2158308654343107	0.2204940923538792	T	T	T
0.5088067235191164	0.0427347756547080	0.3290003055652477	T	T	T
0.3075942575284253	0.6222681672387955	0.4573399804909862	T	T	T
0.3118284243824844	0.1486158682937356	0.2440877320761600	T	T	T
0.3736369479731995	0.4540160973246869	0.5678984600017282	T	T	T
0.4491232787744865	0.7182328514137993	0.5702608048460197	T	T	T
0.2789584888471701	0.4123130217041391	0.0641717833926170	T	T	T
0.9924737180286848	0.5185011327310396	0.1449751324906738	T	T	T
0.4033257767675096	0.5365828845223817	0.7928902667591018	F	F	F
0.7604671970166785	0.3589700374191158	0.0374410471707855	T	T	T
0.0244010063169750	0.3543886508671756	0.8205682803208407	T	T	T

0.0703522175475877	0.1979970025156678	0.9774079738402934	T	T	T
0.7070014382787914	0.4119939705149421	0.8581092248333144	T	T	T
0.1858113363889178	0.4601319961064392	0.1005188115227118	T	T	T
0.3004696549135534	0.4988214310854969	0.0192278336804926	T	T	T
0.9000608863221445	0.4778527876862780	0.1065291078111307	T	T	T
0.9688405725155348	0.6351588676046394	0.1576058455879167	T	T	T
0.5142757767675050	0.5426028845223811	0.8219502667591030	F	F	F
0.7303821453401975	0.3883779652696462	0.9747801671935979	T	T	T
0.6561948058044151	0.3294402008648614	0.0670280903642775	T	T	T
0.1182205292181275	0.4287243465086245	0.8081459199370167	T	T	T
0.0551804409437696	0.2893009390719646	0.8754119410656445	T	T	T
0.9635848823990246	0.2236861788119935	0.0069837289129599	T	T	T
0.1561023992520894	0.2635861975294593	0.0113613082497823	T	T	T
0.8276734121850743	0.4089060260497396	0.8372813562059763	T	T	T
0.6591862242450099	0.3082094252034489	0.8356110339401118	T	T	T
0.2881507767675089	0.7125688845223834	0.9125622667591031	F	F	F

Supplementary Data 14:

FEP+H_2O+HOCl

1.00000000000000

8.0000000000000000	0.0000000000000000	0.0000000000000000
0.0000000000000000	8.0000000000000000	0.0000000000000000
0.0000000000000000	0.0000000000000000	15.0000000000000000

C	F	O	H	Cl
7	16	7	13	1

Direct

0.4297900406008077	0.4209150902740228	0.3856354009603982
0.5181113522690967	0.5421643319526136	0.4538236323279691
0.4522784114869411	0.5473065530596204	0.5534985041832234
0.5679600193955429	0.6665392557266613	0.6129856195509681
0.4700563349321556	0.7900330304207225	0.6765933246641295
0.4726886378562127	0.4564475636444874	0.2847712547061408
0.4330304426857949	0.3694467186626136	0.5975178811269585
0.2603267656397978	0.4333314684230790	0.3938640180154981
0.6841606673714955	0.5023958822061991	0.4551402722797355
0.4983550520224937	0.7000530362990519	0.4201307072880470
0.4777479379474868	0.2605744129813749	0.4032691038405288
0.2934960415712488	0.6160855137311012	0.5512466797379965
0.6718654010582017	0.5714032082668991	0.6645549592424936
0.3591931550239576	0.7099034963073539	0.7292315209999741
0.5835217823644004	0.8673431976151282	0.7291492485700545
0.3888550569794528	0.9074387410757864	0.6283392218716545
0.6665920190033533	0.7667994392438244	0.5615288791705816
0.4012941296559985	0.5994716821519883	0.2564789050672639
0.6390954872681969	0.4641005493880709	0.2716406046188785
0.3045902610547579	0.2862592216057542	0.5593578190500995
0.4098223423876135	0.3282596887596032	0.2355989599626835
0.5737626967651313	0.2779920791823510	0.5887950225797209
0.3967303825762197	0.3838011664382375	0.6856629864160503
0.8267260391097384	0.0736886619314578	0.0369703338806082
0.6645953536090988	0.2748399657882954	0.9225071621654022
0.9744006197392830	0.7596215310014490	0.9694982208226789
0.2077578431623736	0.5500860237609878	0.0086764769164168
0.0371994141883536	0.3098329211281621	0.1145574030482849
0.3383918763812543	0.3224562581534555	0.9040545372942546
0.5022559863694256	0.7253045620901980	0.0448019041495641

0.8918309836146525	0.9795396531116370	0.0136774507478286
0.9081931344691415	0.1509493448564166	0.0652780312173976
0.7216560247820945	0.1933720391483132	0.9634970217426920
0.7317908235929304	0.3762210822979168	0.9255137932043045
0.0670518240499249	0.6709940217544900	0.9812019154831254
0.3057973526707315	0.6180560080623160	0.0269938229515190
0.2541318975066050	0.4683423845313385	0.9631278234217728
0.1018345361108707	0.3949464389935970	0.0812191341274814
0.1098767493997622	0.2751251850444685	0.1629769690690612
0.3176063468672237	0.3152390906776903	0.8404575836458873
0.4617229898145239	0.3018138055867796	0.9124849639037901
0.5222164731137837	0.8404454120285143	0.0628984096585082
0.5930435029808314	0.6999853341004731	0.0033679985542846
0.8408259765510554	0.6684651265354998	0.8974545217640792

Supplementary Data 15:

FEP+H_2O+•Cl

```
   1.00000000000000
    15.0000000000000000     0.0000000000000000     0.0000000000000000
     0.0000000000000000    15.0000000000000000     0.0000000000000000
     0.0000000000000000     0.0000000000000000    25.0000000000000000
   C     F     O     H     Cl
     7    16     6    12     1
Direct
  0.4543142832890434  0.4340780833854078  0.3449729491474804
  0.4496175231472052  0.5092348725801159  0.3890209632594449
  0.4823953607021453  0.4871021879779937  0.4477804694290628
  0.4793587664563747  0.5745158692973507  0.4843110530587179
  0.4399427933910660  0.5603843421731722  0.5423395199497718
  0.3985820675827673  0.4548794436833385  0.2928994801967625
  0.5773431060108249  0.4417236425440127  0.4499080213804501
  0.4228970350932082  0.3556574744462731  0.3649466080663383
  0.4965057058643115  0.5808682765802733  0.3705331577568637
  0.3610131960714891  0.5317363798071812  0.3927867528419250
  0.5408134302070249  0.4232784426392875  0.3292514500441563
  0.4227486031608780  0.4260409586759934  0.4688000237244895
  0.5628046998554554  0.6093311572588926  0.4890258885808402
  0.4800243316526604  0.4932728034841476  0.5688651278287283
  0.4526316779112804  0.6368398072893007  0.5700544459244812
  0.3514152237197839  0.5441698432457145  0.5395821495237280
  0.4266332222092241  0.6394397921769138  0.4619792230811294
  0.3100102834536444  0.4480034964774536  0.3021975107151144
  0.4164443632005040  0.5374266256871731  0.2740601543349193
  0.5716514531951276  0.3579326675820624  0.4302374125549362
  0.4209296193352015  0.3944548772561445  0.2550578253618290
  0.6371510011689652  0.4883004620609582  0.4206468576662311
  0.6069002393164796  0.4358260745086681  0.5007565799605164
  0.7987276737839424  0.3403161281696656  0.4426537680433810
  0.1866600852596800  0.6346443328851697  0.4499609645782683
  0.9117378165108418  0.5503190839027637  0.3750163720443435
  0.1065923759677991  0.4710081521357096  0.4728852127240509
  0.8312310359801059  0.4010889428565240  0.3430878972111616
  0.0897918605579743  0.5208261359903193  0.3727257529961555
```

0.8294832314494324	0.3923258595172761	0.4587106701593728
0.8305925804122876	0.2881506769711120	0.4555694985828445
0.1668529534656779	0.6187691273169053	0.4138164420799741
0.2506028449892941	0.6441077330212871	0.4472610005061935
0.9778128778976423	0.5381117585805436	0.3729865248921311
0.8807979280465098	0.4936094811339367	0.3627871796937217
0.0444515943443256	0.4830794531067446	0.4832333133843293
0.1388446277204627	0.5274875933829151	0.4792565643489826
0.8148176466362337	0.3704084747704806	0.3772508818150995
0.7756988695514092	0.4107740857717303	0.3239547691347957
0.1122843396456791	0.4809591798949045	0.3452225832840564
0.0983436830108253	0.4898002471506643	0.4085414162009733
0.8969881497752040	0.5194059226235167	0.4692854979322243

Supplementary Data 16:

FEP+H_2O+•Cl

```
     1.00000000000000
       8.0000000000000000         0.0000000000000000         0.0000000000000000
       0.0000000000000000         8.0000000000000000         0.0000000000000000
       0.0000000000000000         0.0000000000000000        15.0000000000000000
     C     F     O     H     Cl
       7    16     6    12     1
Direct
  0.4199524933271977  0.4413353288215868  0.3795239327092618
  0.5114804711823830  0.5577093142463927  0.4488632857227629
  0.4614743982903845  0.5441172587698910  0.5508062787420792
  0.5791642742864065  0.6615953182791910  0.6102496413142982
  0.4843912546649889  0.7703978698168885  0.6822452682844052
  0.4437122995570109  0.4962020245653015  0.2793121324448221
  0.4598022662892016  0.3594499162675013  0.5873455801478671
  0.2521907852065245  0.4398830950102511  0.3959411164961386
  0.6791736348613818  0.5285383601200115  0.4416140845107630
  0.4774746523323847  0.7185188153167443  0.4232624646275568
  0.4811000702924086  0.2827348767311830  0.3874792265243224
  0.2991329267361509  0.6023758220188747  0.5585147927676064
  0.6950350420377723  0.5661152534475757  0.6536340556441136
  0.3854469732127503  0.6772039705725564  0.7356351537336381
  0.6009392526370361  0.8465104022932615  0.7334888121708258
  0.3909299606928486  0.8893316303871178  0.6422025623344165
  0.6645522217409082  0.7741872199377722  0.5593101555131589
  0.3539909503452887  0.6346512344190862  0.2613569669383525
  0.6061578060153240  0.5240696324956279  0.2606653060047129
  0.3309917520304180  0.2737340380748469  0.5514047649705704
  0.3874945211780587  0.3700085540873920  0.2268068474023762
  0.6037363681934185  0.2787808420833645  0.5688767433200249
  0.4369737919587246  0.3583207987771496  0.6772357076117907
  0.7754525516480815  0.1396876714947325  0.0380232627287075
  0.6655869522361029  0.4195733408267340  0.9620336857448699
  0.1927888063447929  0.4630786580155107  0.0072045387541757
  0.0276619491034674  0.2703876106040918  0.1312222174859896
  0.3752735438758668  0.3186058347072909  0.8849117475730829
  0.4611718530362752  0.6283985170293227  0.0564733914158235
```

0.8210009338338681	0.0577781197630203	0.9967439490404930
0.8722215584808397	0.1756003067412285	0.0760692662877160
0.7079917203371853	0.3134802839085351	0.9910797809226618
0.7664363835294858	0.4782604119592997	0.9407647862394304
0.2805316237214969	0.5477549263230990	0.0267987444343428
0.2503768132176831	0.4040042129985082	0.9549140614184892
0.0927572272945650	0.3417542317291249	0.0896553326327988
0.1089868480525222	0.2089484746026310	0.1663259233060612
0.3563523897566179	0.3564826264265847	0.8242137080588089
0.4899815771658478	0.3567501915696945	0.9018034463573876
0.4859104172025543	0.7444255986309248	0.0425799020371481
0.5481916934316755	0.5591479149757246	0.0259004540352804
0.0068470246621208	0.6269095481543733	0.9168668705908765

Supplementary Data 17:

FEP+H_2O+K

1.00000000000000

15.0000000000000000	0.0000000000000000	0.0000000000000000
0.0000000000000000	15.0000000000000000	0.0000000000000000
0.0000000000000000	0.0000000000000000	25.0000000000000000

O	H	C	F	K
10	20	7	16	1

Direct

0.2513689542183900	0.2916392281045184	0.1612941037729657
0.6707810175155569	0.7615971739591089	0.2608596281679564
0.4044722436267657	0.4744170850453218	0.0413619275846750
0.9446096309323780	0.3624195047402365	0.1182936363331837
0.2017085490574951	0.6624298090900085	0.2615707676936577
0.5311951371240061	0.7208435267533521	0.3276341920772721
0.2433048418951802	0.3800851056741791	0.0672906342479684
0.1043392356996577	0.5255342096651804	0.2301643183333008
0.1143289651334735	0.4129559793826411	0.1463678136478908
0.2326881148660059	0.6710846961402149	0.3684943044243979
0.1931865765306578	0.3211855266942082	0.1669313630477999
0.2424903827341526	0.2287116692109915	0.1687195877415276
0.7056358465053697	0.7130402572731156	0.2457835734047871
0.6657241690871553	0.8059691636741086	0.2326864234261024
0.4052350669786391	0.4897530674527536	0.0036357304776612
0.3476918084693915	0.4430544520232870	0.0470833304352030
0.8942826813091185	0.3966563154548048	0.1317481631361945
0.9289283078728464	0.3464058435723470	0.0817956336881754
0.1668270736220734	0.6094809111420575	0.2480087129300950
0.1792970959265434	0.7128890143197486	0.2412608350013585
0.5805042853299810	0.7352898196071864	0.3026729007951959
0.5526020488043121	0.7400133177484327	0.3624606177910397
0.2589721126064040	0.3363708172850839	0.0958817259282536
0.2254693793640138	0.3447977119865394	0.0364599409134417
0.0879824863266240	0.4848008963286137	0.2587014100403471
0.1120932172464733	0.4878158323958924	0.1974841824212425
0.1505477369265051	0.4194822532562432	0.1135351120316463
0.0533169813441918	0.3965178491056405	0.1345542033627617
0.1959321562438812	0.6728184141097396	0.3357451327588792
0.1937361148232671	0.6814804064693235	0.3988186553757579

0.4500312624551329	0.5243888405783577	0.1774656861167851
0.5540082326027123	0.5334186271131607	0.1711801520316422
0.6163334772582144	0.4721366734667874	0.2065384203417520
0.7180417630088785	0.4869801380422135	0.1904854557548813
0.7849447300133346	0.4943393919415736	0.2390518941583573
0.3967416562294185	0.6064108717470746	0.1535338650182662
0.5906614372873360	0.3702813154921784	0.2048761671297087
0.4290969093882612	0.5213851131992490	0.2314344235201216
0.5748069924286038	0.5205001679234358	0.1189814045950964
0.5732039371185836	0.6201028978179690	0.1855538323728748
0.4210619792661032	0.4487261275530879	0.1545024614717573
0.6065919414375684	0.4995897267288889	0.2591776046671673
0.7463981629939260	0.4186090503840399	0.1583125593380194
0.7771051202479086	0.4273976753365219	0.2742732529193396
0.8688654121167514	0.4934666281090067	0.2187348929715388
0.7728531072164249	0.5730988377506778	0.2648092580132227
0.7300246466795788	0.5641407590848682	0.1623328106629983
0.3967034072510167	0.6747581762101675	0.1901339231170654
0.4321613700163968	0.6370978265912123	0.1081077057478773
0.5133770463980616	0.3574670603217991	0.2315450299792332
0.3115083865511279	0.5818580616672849	0.1454554556727000
0.5811751347991910	0.3417198671137792	0.1540623349762085
0.6538733043189614	0.3204333044262354	0.2293358144987731
0.3731285647959873	0.6533230297354945	0.3077971329338787

Supplementary Data 18:

FEP+H_2O+Na

```
   1.00000000000000
     15.0000000000000000    0.0000000000000000    0.0000000000000000
      0.0000000000000000   15.0000000000000000    0.0000000000000000
      0.0000000000000000    0.0000000000000000   25.0000000000000000
   O    H    C    F    Na
    10   20    7   16    1
Direct
  0.2067302474984570  0.2790242638242786  0.1873796270257478
  0.6735275648736356  0.7696079862624148  0.2657839545929460
  0.3961257644805236  0.4405588706056712  0.0710542897725433
  0.9231744336894879  0.3555314280131766  0.0999102289833154
  0.1634912611180043  0.6594515944001920  0.2483864499309529
  0.5373705664975339  0.7255845192628344  0.3313313039606609
  0.2330105368073193  0.3479580973459991  0.0890292302917095
  0.0659076424579288  0.5148065742024280  0.2306474026533297
  0.0816844097742415  0.3976944913758995  0.1487489065916887
  0.2703222301412367  0.6504943673436490  0.3307970498016732
  0.1482627038043232  0.3087584331932187  0.1834662567104184
  0.1949641883337621  0.2183594005508336  0.1989294760805240
  0.7121751321661632  0.7229041580138161  0.2519496868560162
  0.6655960982746245  0.8118262882763705  0.2366888801711707
  0.3972937852571333  0.4596732999725981  0.0339627965999328
  0.3389697839568971  0.4091416155322490  0.0753631402176033
  0.8794870314518333  0.4025447822512636  0.0943531654964548
  0.9215671665119592  0.3194593374122557  0.0676177531441425
  0.1271937685512441  0.6047257906945602  0.2401412337651373
  0.1234963323557358  0.7099038141948932  0.2446028280233578
  0.5873158429296171  0.7415153934678024  0.3069119108812839
  0.5532710597382934  0.7509127922823045  0.3657605272053515
  0.2375780586404392  0.3105173960269367  0.1219083930224139
  0.2245014237323519  0.3066051077261726  0.0595232914518542
  0.0666436926778381  0.4746632064543202  0.2611183665701252
  0.0744697503742013  0.4759922107815055  0.1986084643321229
  0.1280153342717136  0.3981815445058866  0.1206775192640278
  0.0246985776313200  0.3835842756860454  0.1304601322985051
  0.2243684619380437  0.6546241345169936  0.3012322052982735
  0.2377037521233029  0.6489942454695847  0.3642442111962867
```

0.4626012400498263	0.5190638677046940	0.1972215745125116
0.5573313282817779	0.5433241174391369	0.1746101213270560
0.6424835774662875	0.4967914999505760	0.1995004605200756
0.7286639220293740	0.5234803154799625	0.1660657118956448
0.8158337189446578	0.5418845328075005	0.1998391248777320
0.3864447145483681	0.5858007023905991	0.1787307282433054
0.6332250461282691	0.3924781961020235	0.2048700390962840
0.4656925797265555	0.5245747775799567	0.2527807216851360
0.5571264636186712	0.5280406845483803	0.1212710513641130
0.5664783994470451	0.6333889158871870	0.1843074252033253
0.4391850369841194	0.4357717664106137	0.1838174747166730
0.6533286447646179	0.5300763805758641	0.2508173030856426
0.7476042589562586	0.4573049185354824	0.1300964334228085
0.8349725727349692	0.4770695261558398	0.2348509658095215
0.8839596245670096	0.5504306736343144	0.1649563354420122
0.8074142842312132	0.6202656348433101	0.2269614262782070
0.7155630894084672	0.5999655601843822	0.1376289479977701
0.3858441553070276	0.6581719328209719	0.2129312991097419
0.3979968953512665	0.6167201352029119	0.1292327093866124
0.5754847136137378	0.3734691901635601	0.2447860604815101
0.3074030730584555	0.5448465458930910	0.1829687782905240
0.6034417181068737	0.3560983212083988	0.1589689134509929
0.7122591542448391	0.3547729284192945	0.2176003815951115
0.4126953863711714	0.6537794824158021	0.3095774290181228

Supplementary Data 19:

FEP+H_2O+Li

1.00000000000000

15.0000000000000000	0.0000000000000000	0.0000000000000000
0.0000000000000000	15.0000000000000000	0.0000000000000000
0.0000000000000000	0.0000000000000000	25.0000000000000000

O	H	C	F	Li
10	20	7	16	1

Direct

0.2062847412768015	0.4376226091698812	0.1448687733227715
0.8586032409153377	0.7291979530802669	0.3227512242727163
0.3153835719763465	0.4747698247010235	0.0634379468423979
0.9256608504168397	0.3809990958812521	0.0646796135667624
0.8547269991580938	0.6159894516459710	0.2312029199227142
0.6968822893754975	0.6577592031948087	0.3074566244385077
0.3312288071446257	0.3088043682666484	0.1063776577954607
0.9609314519786908	0.4846795881766336	0.2395463730017341
0.0246896043958030	0.4662579906819378	0.1447021771046963
0.5598672797399193	0.6583778286471650	0.3795323983072940
0.2396453678068635	0.4648150054724235	0.1141185303713963
0.2293522750494502	0.4664423500639033	0.1768615363268516
0.9010832965492478	0.7220456094675344	0.3517612757927632
0.8793172439595295	0.6909802189395976	0.2932543279983831
0.2992932968973029	0.4865660404202613	0.0264844613625956
0.3327361236650591	0.4111535146010936	0.0656350865171138
0.9280272613876848	0.4100688331669005	0.0299259864529886
0.9283905785434823	0.3174700529551697	0.0571157782081659
0.8959835377871908	0.5605551954879736	0.2353023104975372
0.8772837355355175	0.6471541437838642	0.1999015573772585
0.7489228877411880	0.6896901863066891	0.3230056515423180
0.6474817776932696	0.6592357271010685	0.3336575665971072
0.2800629379965650	0.3299649376139600	0.1274786973847930
0.3164647552702341	0.2489528810669727	0.0944931591210292
0.9428153548802607	0.4286585674720619	0.2556178428723910
0.9860789116469322	0.4708698440216735	0.2023752984910397
0.0895010817582751	0.4537279584232329	0.1421601186904495
0.9939866820465836	0.4317647258275067	0.1166791771815482
0.5277226935216199	0.7137526424161182	0.3856545222339933
0.5702353901835630	0.6332029939456055	0.4148405727071846

0.4383639255674326	0.5276001897311448	0.2448545315349900
0.4974278007522746	0.5435666675820710	0.1929067971105665
0.5865732644944789	0.4874610007914355	0.1853788634665557
0.6453651325384784	0.5307361423810558	0.1392222856609366
0.7280465708644739	0.4782945556519703	0.1162061358498612
0.3808445756406342	0.6111934887981012	0.2611463987254298
0.5687825583787098	0.3851326168053819	0.1780483094846330
0.4896515254868702	0.5050074397536289	0.2875233527775471
0.4443173292696165	0.5293384982944251	0.1498136631928812
0.5219468407442122	0.6317645497600513	0.1937438354833139
0.3790262340159568	0.4606097179372252	0.2348480184165646
0.6377850012393197	0.4965376437685394	0.2319473781818795
0.5939748600595854	0.5549262836716057	0.0974291170403749
0.7023137580022628	0.4073469671756081	0.0872783828661305
0.7733766723497700	0.5347570827895438	0.0840871921191620
0.7823709045108025	0.4521307539776864	0.1564422082825093
0.6826651829617266	0.6074786580617326	0.1614383080383914
0.4316361794694894	0.6780823347990244	0.2802458613681372
0.3337591280559737	0.6406790185573138	0.2183820534672090
0.5138253062463966	0.3550038495262282	0.2167502032238231
0.3230737424936637	0.5859750591300940	0.2997234840490413
0.5319683286800756	0.3694157375884050	0.1298875521261863
0.6462120844215051	0.3394106780299934	0.1823132731088963
0.7299992674585131	0.6071917494384786	0.2444837271230211

Supplementary Data 20:

FEP+H_2O+ •Cl + •Cl

1.00000000000000

8.0000000000000000	0.0000000000000000	0.0000000000000000
0.0000000000000000	8.0000000000000000	0.0000000000000000
0.0000000000000000	0.0000000000000000	15.0000000000000000

C F O Cl H

7 16 6 2 12

Selective dynamics

Direct

0.6596365780371558	0.1542743665513659	0.3375746813650176	T	T	T
0.6691188508002122	0.3455061848171556	0.3630135996627401	T	T	T
0.7203182673559378	0.3953672835023938	0.4613686530613825	T	T	T
0.7327946563495917	0.5942079695567644	0.4714213183703275	T	T	T
0.6425928555551820	0.6678762502221013	0.5564579589086041	T	T	T
0.5612609604644572	0.1177634448759226	0.2484897201359671	T	T	T
0.8851210643775684	0.3077762783799367	0.4952895028964996	T	T	T
0.5813319004618209	0.0666335863293254	0.4029787313409867	T	T	T
0.7788210908944198	0.4201371853757728	0.3049473082708261	T	T	T
0.5124503101626317	0.4082117258978146	0.3485436765847724	T	T	T
0.8178375986657018	0.0941659716690011	0.3260384170626385	T	T	T
0.5929541409281783	0.3399385722860906	0.5164202266028624	T	T	T
0.8963315471787505	0.6416367580599828	0.4747625310359228	T	T	T
0.4757039768381283	0.6502403214929116	0.5505435773288088	T	T	T
0.6965732165161981	0.5931412331543625	0.6327440228257334	T	T	T
0.6796537658918781	0.8321103882248274	0.5617642048421534	T	T	T
0.6617372374775745	0.6747719631494331	0.4007188872134477	T	T	T
0.3957588193962995	0.1407548377692557	0.2583202353198039	T	T	T
0.6161538595034450	0.2184153063212922	0.1810872620543572	T	T	T
0.8573707626614805	0.1422760005718912	0.5055421236806864	T	T	T
0.5890453693390271	0.9574816422563466	0.2244974714544669	T	T	T
0.0119694181476493	0.3297815862995377	0.4370231238544138	T	T	T
0.9305385605505978	0.3693537631116743	0.5756458798123611	T	T	T
0.6350142151727731	0.0459585679336870	0.8688388677464457	T	T	T
0.8782848319241704	0.4506370120541682	0.0869845749969843	T	T	T
0.1605460860200364	0.6124258215987107	0.1398487001184164	T	T	T
0.5792659218111140	0.5151035057857311	0.8299504933973930	T	T	T
0.4169043222778021	0.2393764798598178	0.7780597153809022	T	T	T

0.7832693771264585	0.3186723570350248	0.9292493744267909	T	T	T
0.2701135986075514	0.0996608766645082	0.5944478748391475	F	F	F
0.2290810513654264	0.7137354138424996	0.3407918796307001	F	F	F
0.6056707796723808	0.9636367417027073	0.9138642042429926	T	T	T
0.7040668452493001	0.1345237749812007	0.8988923993096838	T	T	T
0.8389754885915779	0.3924099089114541	0.1398378357064984	T	T	T
0.9821810790557730	0.5103831023898940	0.1053584176479502	T	T	T
0.1759170134827411	0.6459500097362896	0.2028442307689788	T	T	T
0.2672131013271590	0.5653034045185678	0.1215413689235353	T	T	T
0.5056914878495727	0.4261111117748835	0.8040839658298444	T	T	T
0.6245341481434151	0.5762187510650406	0.7793010249046251	T	T	T
0.3763167990273544	0.1969498788882979	0.7201814372624217	T	T	T
0.4876650531936714	0.1507405664620319	0.8060859973491785	T	T	T
0.7184962663845208	0.4060287584225727	0.8963396326652803	T	T	T
0.8224871357542917	0.3676952377832838	0.9869119266901045	T	T	T

Supplementary Data 21:

FEP+H_2O+Cl_2

1.00000000000000

8.0000000000000000	0.0000000000000000	0.0000000000000000
0.0000000000000000	8.0000000000000000	0.0000000000000000
0.0000000000000000	0.0000000000000000	15.0000000000000000

C	F	O	Cl	H
7	16	6	2	12

Direct

0.4611004229981536	0.2615344826704181	0.2923675183028001
0.4803160795191059	0.4390897429970659	0.3369765295046542
0.5654998875941267	0.4518400487921496	0.4320964714320025
0.5840142352052740	0.6433618390356520	0.4613352865697509
0.5307434108404139	0.6820808070409207	0.5606306969890708
0.3355434464306635	0.2568987693824969	0.2113018068732395
0.7380073486464442	0.3571936733135795	0.4391414711719624
0.4048957260116770	0.1483850919876237	0.3533175426334268
0.5674112158459708	0.5380168367216708	0.2790219450736547
0.3212252725059386	0.5001149419551234	0.3461117464137234
0.6129584965450966	0.2111002915424859	0.2606948875600387
0.4590916901004473	0.3741023165138009	0.4919060804453111
0.7461184371176526	0.6932629374729945	0.4505115433603764
0.3640437658401763	0.6642053400483506	0.5704846308408780
0.6101071591060920	0.5836902687212240	0.6201376611557680
0.5713125386575078	0.8433050387257849	0.5780347507153220
0.4874200768178030	0.7462276340883379	0.4101094056039550
0.1753588494263995	0.2715741814239048	0.2391353790281907
0.3687353046830604	0.3801852979741977	0.1523685962966042
0.7122531012155963	0.1902163296188804	0.4355260366503392
0.3523756405595209	0.1077204460882897	0.1698401458628814
0.8420406628351655	0.4010915140023129	0.3719008518872495
0.8140668570464653	0.3902716839948904	0.5176105338547606
0.8075033240787204	0.2013776329434371	0.9170918445586645
0.9807023284745860	0.6791224534479092	0.0423128047041542
0.2924073918719579	0.7976654284805290	0.0271314127278916
0.4915463000041086	0.6847060225243320	0.8837932470473975
0.2123492959300431	0.5328837233930811	0.8152367954231549
0.8292956974960080	0.5251714760905261	0.9063836140158162
0.2491883504973234	0.1774739821291182	0.9230101147664870

0.5182451842947794	0.1874683209396578	0.9210043292715134
0.8310333430709772	0.1663475188943331	0.9781623962580773
0.8180267614333226	0.3305215982365527	0.9159663731359707
0.9721317752310016	0.6341005702964490	0.1022084029290773
0.0997819683787150	0.7172175352318749	0.0352855653752909
0.2821137471601711	0.9164894318739455	0.0134657483553528
0.3696210451118454	0.7538516666261518	0.9809321994003202
0.3933524952928489	0.6297438364891178	0.8544999893265436
0.5121531537510019	0.7859195937548132	0.8494999883250507
0.0999022033404258	0.5698693136738462	0.8314582772314035
0.2158361953895776	0.4162073302501241	0.8349791636378247
0.7177775075393640	0.5748474465835157	0.8975606958877828
0.8804423911044515	0.5828255640285189	0.9592852993963025

Supplementary Data 22:

FEP+H_2O+•OH + •Cl

1.00000000000000
9.0000000000000000 0.0000000000000000 0.0000000000000000
0.0000000000000000 9.0000000000000000 0.0000000000000000
0.0000000000000000 0.0000000000000000 18.0000000000000000
C F O H Cl
7 16 7 13 1
Direct
0.3884459447061149 0.6897022924401662 0.6090290359026992
0.4187412762234902 0.5197055976400904 0.6239550956443087
0.5850763863067970 0.4629607148073917 0.6285161776197672
0.5895320132530483 0.2908187162388930 0.6508629191360943
0.7012145953676739 0.1928664419398000 0.6040152570469781
0.2232780815598877 0.7268810450804013 0.5861908547395007
0.6865933623469976 0.5585840743412481 0.6815992610247039
0.4767681779737241 0.7389914404599028 0.5523432023989165
0.3471427686023831 0.4821628621613697 0.6877245845827463
0.3541936454506879 0.4453308559782737 0.5650721875071429
0.4186832431626480 0.7686391443737289 0.6716656627714456
0.6446076890263793 0.4769694950656778 0.5578540169212387
0.6258420764282115 0.2771469343226657 0.7239998368773348
0.8401904246746216 0.2488077058338459 0.6036118701111207
0.7051899912339842 0.0559338773833663 0.6350836200771526
0.6527552590558156 0.1792358323256324 0.5332130227871918
0.4541150950759618 0.2247204292329296 0.6410701945254537
0.1934416279619667 0.6788143235575295 0.5164274956194396
0.1246827932191522 0.6658734543098567 0.6335895873724541
0.7101408093548364 0.6939852906779019 0.6508896036663848
0.2068252500308165 0.8764161613611771 0.5872342642423516
0.6228707770813040 0.5765926393718229 0.7488350317949294
0.8209874202938823 0.4939074686006749 0.6905690076168474
0.7983231982462261 0.2853951942149063 0.2930647510302482
0.4847573261976938 0.9500771181086222 0.3761864869447200
0.6026388172352879 0.6908274856738069 0.3528619900417750
0.3852820211333892 0.4710670469628323 0.3767639730272244
0.8681840779881288 0.5574512804734031 0.3374037588317965
0.1538690550744733 0.6003816653087675 0.3110032210501841

0.5203995978145358	0.2098851944213433	0.3188505851112803
0.6926536328235980	0.2523907885529892	0.3025249565478824
0.5235873927251788	0.6162156559345177	0.3628485387587737
0.5584542403174835	0.7940212838211111	0.3602470325020801
0.4110176452070178	-0.0560223491368713	0.4159886658028221
0.8172445467604929	0.2672724511889693	0.2406656109335107
0.2939580017438052	0.5135007197624623	0.3523290121888765
0.7754929318234478	0.6173807778198684	0.3435369306172864
0.8377417756007847	0.4538698039186863	0.3224998466718338
0.4998574163394857	0.1097498352547318	0.3434012536430328
0.4604290472266722	0.2897141222181716	0.3419392322946168
0.3616382259338695	0.4659417319490865	0.4294557461577314
0.1701132786307703	0.6944951562297503	0.2859273608610714
0.0445064554695074	0.5929202110445994	0.3217397687539454
0.3391724413177687	0.3630880607738626	0.8784993212430923

Supplementary Data 23:

FEP+H_2O+HOCl

1.00000000000000

9.0000000000000000	0.0000000000000000	0.0000000000000000
0.0000000000000000	9.0000000000000000	0.0000000000000000
0.0000000000000000	0.0000000000000000	18.0000000000000000

C	F	O	H	Cl
7	16	7	13	1

Direct

0.6440832598726217	0.7000206575786054	0.5864899258530388
0.5671892458648654	0.5424812508798682	0.5855904434587815
0.4777798737041618	0.4905429434049640	0.6569094618235899
0.3993237553335919	0.3336578568157979	0.6419934627602556
0.4162085044583315	0.2164763650445204	0.7068147372242859
0.7621150860875282	0.7245779358779718	0.5228363762903850
0.3622109444564794	0.6085617640038311	0.6861353465924752
0.7165013010196020	0.7210464605479457	0.6522502460648820
0.4737046216159259	0.5382539531798487	0.5260133874782774
0.6794860844167963	0.4414930613522304	0.5756387370873925
0.5380751028169207	0.8071506168889671	0.5782533066034538
0.5804020769397390	0.4712464852366210	0.7134512452228400
0.2509445086234161	0.3546379946769319	0.6295114876873208
0.3714317834132680	0.2705680336093219	0.7730119687404979
0.3304907952457975	0.0975627649158237	0.6897102769366434
0.5586388236185077	0.1705002733236438	0.7123479741073623
0.4558174911502481	0.2648208558099869	0.5805829813277058
0.8860824749285861	0.6440163592774717	0.5349613834856681
0.7054987006582661	0.6890566798014861	0.4556556382762538
0.4349396778081507	0.7267533299516433	0.7150794933533484
0.7999168694705335	0.8704921425745036	0.5223628759916338
0.2721843704161101	0.6564132526152107	0.6310145517290560
0.2774708113164913	0.5505443219335756	0.7410671200790109
0.1280608609153185	0.1202186238600444	0.3539486960673058
0.8794107099961869	0.2342807979979617	0.2796267556745186
0.4022052948122329	0.2085732758483570	0.3098959646355415
0.3551931875872109	0.7039676320845873	0.2965072289422642
0.5053521807057711	0.4583186918479663	0.3338895495613166
0.0926919488309750	0.8064345070845125	0.3519408070297279
0.7784240047611282	0.5141809043511723	0.2746864256474400

0.2285677194463008	0.1547068510193579	0.3365813947472168
0.1244872463576065	0.1468553991926897	0.4063138468192996
0.7957338870231254	0.1747014552983336	0.2968671490322609
0.9685385434673257	0.1972412328015660	0.3060858156542052
0.4490494593986962	0.3207540792224964	0.3201924737905062
0.4179268924483452	0.7917140526716878	0.3002290456294690
0.4411773463537731	0.5463642547846820	0.3215989264316938
0.6048314424252782	0.4786695940192071	0.3110646065556909
0.7714886184175239	0.5359760448708091	0.2219001319095324
0.8275672507917935	0.4141815664358449	0.2772695677433099
0.2571739018864362	0.7355412328763389	0.3171486938930708
0.0957432986755298	0.9160420217379595	0.3482777060041616
0.0035441532452702	0.7733336499131411	0.3262461230286626
0.5295960722182389	0.0872188957805049	0.3425666050286544

Supplementary Data 24:

FEP+H_2O+•OH + •Cl

```
   1.00000000000000
    10.0000000000000000     0.0000000000000000     0.0000000000000000
     0.0000000000000000    10.0000000000000000     0.0000000000000000
     0.0000000000000000     0.0000000000000000    20.0000000000000000
   C    F    O    H    Cl
     7    16     7    13     1
Direct
  0.6285186485650964  0.3435213336404447  0.4081007144395826
  0.5250303153453211  0.4528149037746672  0.4317317802753036
  0.4199110581589969  0.5083993316113791  0.3792679906072229
  0.3176730944309873  0.6084343609702690  0.4149620485890743
  0.2866798838122225  0.7413216249822309  0.3750546596028079
  0.7488576686929315  0.3204154364193483  0.4577479615204826
  0.3429711319799661  0.3957830481220659  0.3395848336053187
  0.6820995242589541  0.3790301671542488  0.3480776369596864
  0.4562722595400376  0.4016061943749498  0.4850218577167039
  0.5994586215011264  0.5599735006906512  0.4525569214482681
  0.5641881441279200  0.2237607705114162  0.4018325499608371
  0.4910196940631434  0.5808146654453301  0.3322439401532687
  0.1987558935398412  0.5441362036303303  0.4264542686359351
  0.2474980927524237  0.7180094588143056  0.3117532028224492
  0.1865712326987581  0.8055652882243098  0.4075703161021163
  0.3953246963278820  0.8215829616349223  0.3745877691339624
  0.3639373629780925  0.6502360382256982  0.4757538546507165
  0.8331431039654803  0.4259077553579938  0.4582073991230042
  0.7044569364016429  0.2980123699021212  0.5206101162849470
  0.4278642169495359  0.3338846605757417  0.2969953973329579
  0.8175864432302951  0.2115045002058525  0.4369254608097415
  0.2906067906196846  0.3036652498720920  0.3816447147341729
  0.2430837020942487  0.4486306988965237  0.3025443061132962
  0.1227682077473596  0.5109082516477185  0.7842680380863932
  0.8224686029067255  0.5286449703926034  0.6308737018074673
  0.0654443127649643  0.5515775135942997  0.5867705890298737
  0.6321067118830914  0.5171651119972295  0.7893659828446691
  0.5326230419715028  0.5469460245623806  0.6635400667309604
  0.8818043114476427  0.4213882055115911  0.7561049038655457
```

0.2589425599011481	0.5332748986176431	0.6704801165802357
0.1774600664599744	0.4724727934753380	0.8194625792900305
0.5660207794216627	0.5373613486526273	0.7105245309888946
0.8454258683497103	0.4899020241020262	0.6752988513127305
0.9132085995890420	0.5457941344433326	0.6093859185702050
0.7258960533915385	0.4859436790005388	0.7845896388838349
0.1816577850038034	0.5193445498044588	0.7438474669577579
0.0785665374170624	0.5702766039231032	0.5391708375300741
0.6137767369322247	0.5447533598955996	0.6358938113246952
0.1946806437754177	0.5391737781897222	0.6311271420379215
0.3543561197553730	0.5418512114341250	0.6573659456989469
0.6363004562674562	0.5977512267577936	0.8164038605044239
0.9746374992273907	0.4526334268242402	0.7693176098769228
0.8858736968681293	0.3243572526521101	0.7543937767712660
0.9112131138841940	0.5904593604846433	0.2557347766852877

Supplementary Data 25:

FEP+H_2O+HOCl

1.00000000000000

10.0000000000000000	0.0000000000000000	0.0000000000000000
0.0000000000000000	10.0000000000000000	0.0000000000000000
0.0000000000000000	0.0000000000000000	20.0000000000000000

C	F	O	H	Cl
7	16	7	13	1

Direct

0.6132843071517053	0.6408488751344082	0.5545846186870311
0.4924394344585744	0.6214310663378344	0.6043289718285504
0.4813594839354076	0.4853633850097139	0.6446299326044518
0.3590830700139607	0.4915073215400143	0.6961684958803933
0.2683993284581155	0.3621323684895134	0.6980268106696255
0.5957000179299994	0.7608230776410615	0.5041258182707007
0.6146653987061168	0.4434561483171574	0.6811790568576381
0.6309926577159559	0.5285094032016614	0.5171027248423414
0.4957932835753658	0.7244478196313785	0.6492031459449741
0.3791115340560285	0.6314774778158128	0.5661775235627960
0.7266169377062440	0.6655739945721337	0.5905422346173936
0.4547609424980527	0.3856469576670062	0.5985844395736732
0.4066803056311877	0.5135623398197415	0.7592393634973099
0.3397974566319617	0.2495763963079139	0.7083428883049414
0.1806254296965413	0.3764146213250398	0.7491230115696322
0.1985874183333014	0.3501266492719073	0.6405697119693966
0.2735447344275612	0.5938470491162957	0.6815125588707290
0.5039719553799024	0.7322919819479514	0.4565797857047976
0.5576212887835879	0.8737604143458632	0.5363758502078996
0.7075875356255321	0.4065913134360082	0.6351660682785484
0.7142469471042978	0.7833582978401558	0.4738266922893587
0.6649671880534587	0.5447461919729600	0.7180306377734412
0.5944741330962433	0.3367700289879713	0.7214080454190993
0.2124328311879264	0.6171254698596290	0.2725615590931155
0.7482798893065905	0.3610223638143168	0.3787256452952102
0.3272153664871917	0.3033903712516766	0.3786131917176427
0.6990300616473651	0.2011588609930042	0.4833118908496116
0.4337230898422930	0.1575975735345227	0.4806389875306100
0.5198609814577317	0.3711326058343727	0.3095381660514164
0.4797149764543946	0.6036001194876948	0.2429708864054272

0.1548391396287333	0.6946595154426352	0.2777150622815695
0.2057942556755896	0.5668975039014853	0.3146388160431839
0.7344189946652951	0.3017150280513163	0.4187841898296321
0.8231635819559661	0.3226553580583976	0.3544721267042352
0.4121443779962080	0.3401766875836355	0.3485489288329551
0.6007487971262655	0.1813956066599009	0.4836136539702550
0.4015516319223936	0.0662300057063234	0.4776000149647687
0.3895636000291175	0.2080946191872301	0.4436604681941155
0.5274055555085636	0.6819214772375976	0.2589651991744555
0.3833372716685768	0.6222992266461499	0.2505950887302439
0.7182897447394797	0.2408285182380463	0.5265869628876332
0.5117948277814929	0.4581961111467990	0.2852559192419415
0.6071805596271295	0.3700642545377409	0.3345188797709011
0.2503899403225881	0.4400253900980066	0.4098560052063344
